# In-solution direct oxidative coupling for the integration of sulfur/selenium into DNA-encoded chemical libraries†

**DOI:** 10.1039/d1sc06268a

**Published:** 2022-02-01

**Authors:** Shilian Yang, Guixian Zhao, Yuting Gao, Yang Sun, Gong Zhang, Xiaohong Fan, Yangfeng Li, Yizhou Li

**Affiliations:** Chongqing Key Laboratory of Natural Product Synthesis and Drug Research, Innovative Drug Research Center, School of Pharmaceutical Sciences, Chongqing University Chongqing 401331 P. R. China yizhouli@cqu.edu.cn yfli3@cqu.edu.cn; Pharmaceutical Department of Chongqing Three Gorges Central Hospital, Chongqing University Chongqing 404100 P. R. China; Chemical Biology Research Center, School of Pharmaceutical Sciences, Chongqing University Chongqing 401331 P. R. China; Key Laboratory of Biorheological Science and Technology, Ministry of Education, College of Bioengineering, Chongqing University Chongqing 400044 P. R. China

## Abstract

Sulfur/selenium-containing electron-rich arenes (ERAs) exist in a wide range of both approved and investigational drugs with diverse pharmacological activities. These unique chemical structures and bioactive properties, if combined with the emerging DNA-encoded chemical library (DEL) technique, would facilitate drug and chemical probe discovery. However, it remains challenging, as there is no general DNA-compatible synthetic methodology available for the formation of C–S and C–Se bonds in aqueous solution. Herein, an in-solution direct oxidative coupling procedure that could efficiently integrate sulfur/selenium into the ERA under mild conditions is presented. This method features simple DNA-conjugated electron-rich arenes with a broad substrate scope and a transition-metal free process. Furthermore, this synthetic methodology, examined by a scale-up reaction test and late-stage precise modification in a mock peptide-like DEL synthesis, will enable its utility for the synthesis of sulfur/selenium-containing DNA-encoded libraries and the discovery of bioactive agents.

## Abstract 2

DNA-compatible direct oxidative coupling using various sulfur/selenium sources has been achieved, featuring pre-functionalization-free substrates and transition metal-free condition.

## Graphical abstract

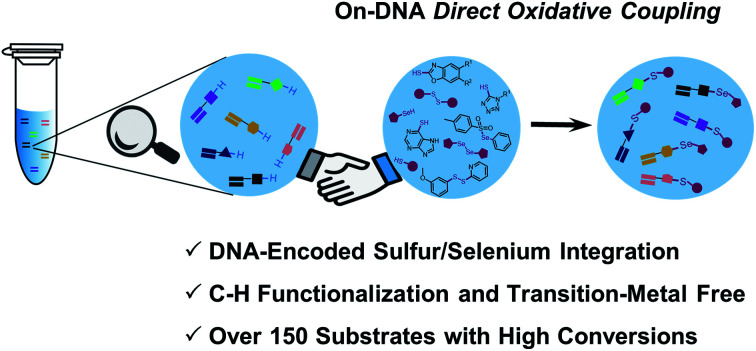

## Introduction

**Fig. 1:**
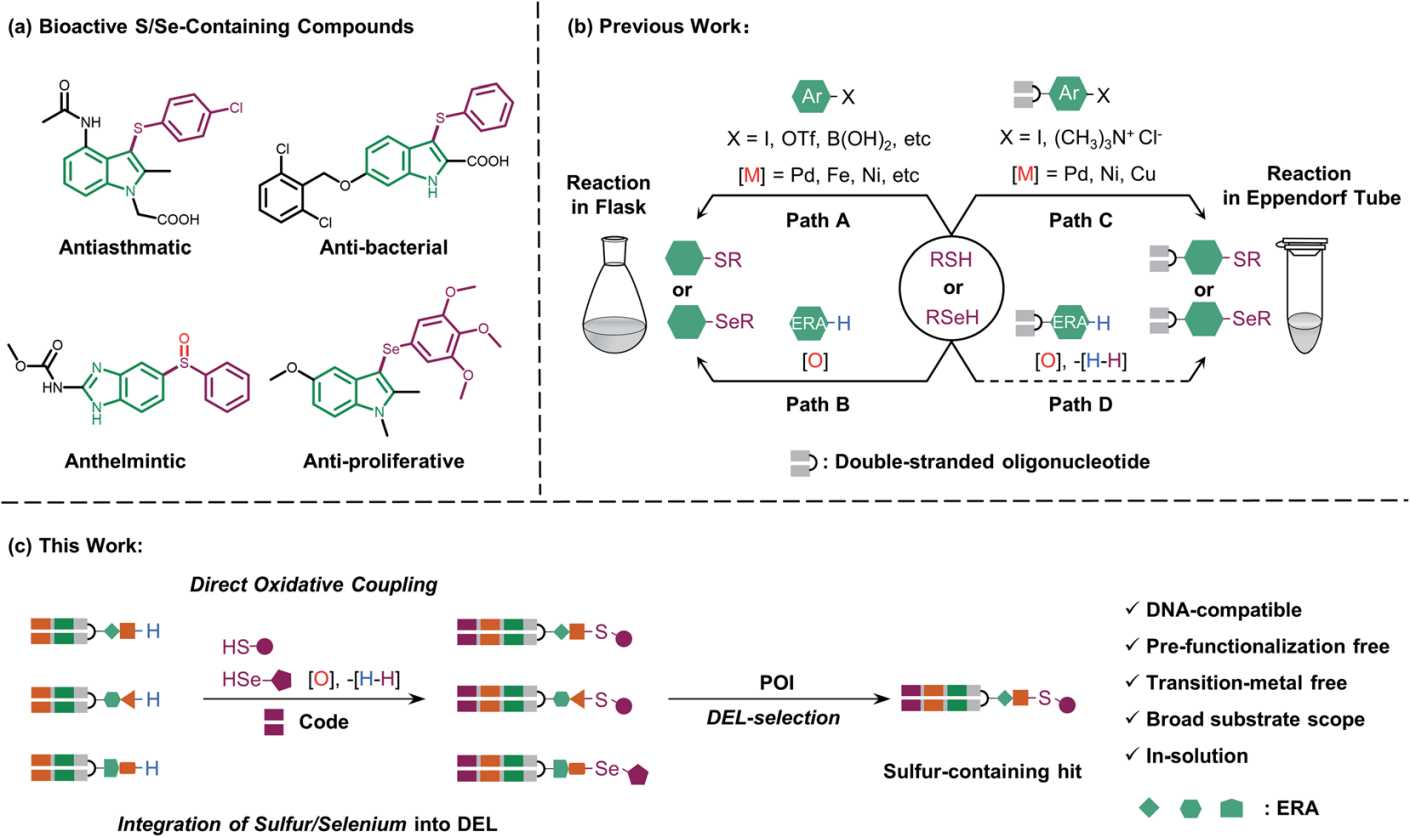
(a) Representative S/Se-containing compounds are shown with different pharmacological activities. (b) Previous methods of C–S/Se bond formation are illustrated and classified into traditional organic synthesis and on-DNA library construction. (c) In-solution direct oxidative coupling in this work is illustrated by the construction of a S/Se-containing DEL, and a proposed S-containing hit could be isolated from DEL-selection against POI.

Sulfur-containing organic moieties are present in a variety of biologically and pharmacologically active molecules.^1^ Many drugs with aryl sulfides have been applied for cancer,^2^ anti-inflammatory,^3^ antiparasitic,^4^ and antibacterial^5^ treatments (Fig. 1a). Located in the same main group of sulfur, selenium is essential for human metabolism, and over 25 selenoproteins are involved in diverse molecular pathways and biological functions.^6^ Besides, organoselenium arenes have received great attention since they are widely used as therapeutics (Fig. 1a).^7^ Consequently, sulfur/selenium-containing arenes have gained great attention in drug discovery campaigns. The development of synthetic methodologies to produce sulfur/selenium-containing arenes through simple C–S and C–Se bond forming reactions is hence considered highly important.^8^ Much of the efforts on C–S and C–Se bond formation have been devoted to the direct coupling of aryl halides, triflates, and boronic acids with aryl thiols using transition metals like palladium, nickel, iron, copper, rhodium, *etc.* (see path A in Fig. 1b).^9^ However, these coupling methods have obvious limitations, including the air-sensitive metal–ligand combinations and harsh conditions like high-temperature treatment. Moreover, highly pre-functionalized substrates are required in such procedures.

More recently, direct oxidative coupling provides an avenue for C–S and C–Se bond formation by C–H functionalization, which offers benefits for bioactive compound production without specialized, toxic, or high-cost reagents (see path B in Fig. 1b).^10^ This strategy presents a truly efficient and convenient protocol for the construction of a desirable sulfur/selenium-containing combinatorial library by using thiols/selenols as building blocks (BBs) followed by the isolation of bioactive compounds from the high throughput screening (HTS) procedure.^11^

Beyond the traditional HTS approach of target-based screening of individual compounds with the support of expensive and sophisticated facilities, genetic barcoding of compounds with amplifiable DNA tags allows for the construction of DNA-encoded chemical libraries in which the structural information is encoded for each compound of the library.^12^ With these characteristics, DEL technology enables generation^13^ and selection^14^ of synthetic molecules with large chemical diversity.^15^ Collectively, incorporating sulfur and selenium into DNA-encoded compounds would increase the diversity of DELs and build the foundation for discovering sulfur/selenium-containing drugs.

In general, the development of chemistries that integrate C–S and C–Se bond formation with DEL synthesis expands the chemical space of DELs and is of great interest. However, the distinctive nature of the DNA barcode (which is soluble in the aqueous phase and rich in π-electrons on the nucleobases) limits the use of both metal–ligand combinations and strong oxidants, which are typically applied for C–S and C–Se bond formation in the reaction flask. Recently, a few conditions have been developed for the creation of C–S bonds to allow for more chemical diversity for DEL design and synthesis.^16^ Besides, these conditions employ transition metal promoters (palladium, nickel, and copper) which are not only accompanied by the need for special ligands but also might potentially inhibit enzymatic DNA-tagging efficiency during a library construction or disrupt a further selection campaign against the protein-of-interest (POI, like kinase or phosphatase).^17^ Moreover, Zhang and Lu reported an on-DNA transition-metal free aryl-S bond formation by using novel aryl ammonium building blocks through the S_N_Ar process.^18^ For on-DNA selenylation, to the best of our knowledge, there are only two seminal reports from the groups Lerner and Yang^19^ and Zhang,^20^ respectively, where the former achieved Rh-catalyzed C–H functionalization *via* bi-functional benzoselenazolone and the latter achieved radical selenylation through a heterogeneous solid support-mediated on-DNA strategy (see path C in Fig. 1b). Therefore, to investigate larger and deeper chemical spaces, it is crucial yet challenging to develop new, efficient, and robust methodologies, which could incorporate sulfur/selenium into DEL synthesis with commonly available diversified BBs.

Considering the high demand for a transition-metal free method for the introduction of sulfur/selenium into DELs under mild conditions (low temperature and in solution) and impelled by our research interest in DEL design and selection,^21^ we were motivated to develop a new and versatile synthetic methodology, especially one that would allow on-DNA C–S and C–Se bond formation, together with various sulfur/selenium sources. We envisioned that on-DNA direct oxidative coupling could fulfill these demands. A simple dehydrogenative C–H/S–H or C–H/Se–H cross-coupling reaction would result in C–S and C–Se bond formation. Moreover, this strategy avoids the preparation of DNA-conjugates with additional functional groups (like aryl halides or ammonium) to render more BBs applicable and efficient for DEL synthesis (see path D in Fig. 1b). We recognized that the complexity of selective on-DNA C–H oxidative functionalization^22^ and oxidants might cause a serious issue of DNA damage, but we reasoned that these barriers could be overcome by oxidative coupling to electron-rich arenes (ERAs) and fine-tuning the oxidative systems.

Herein, we present our study of on-DNA sulfenylation and selenylation with electron-rich arenes representing pharmacologically interesting structural moieties *via* direct oxidative coupling (Fig. 1c). Remarkably, this methodology using wide-scope S/Se-containing sources and pre-functionalization-free DNA-conjugated aryl substrates, could expand the chemical space of DELs and facilitate the isolation of sulfur-/selenium-containing bioactive compounds. The reaction process works efficiently in a transition-metal and ligand-free fashion, compared with the recently developed C–S and C–Se bond-forming reactions relying on transition-metal promoter/ligand combinations. Besides, we demonstrated that further oxidation of sulfide and selenide DNA conjugates to afford the corresponding sulfoxide and selenoxide derivatives conveniently widens the chemical diversity during encoded library synthesis. Scale-up synthesis and the possibility of late-stage precise functionalization provide a suitable avenue to integrate sulfur/selenium into DEL design and synthesis.

## Results and discussion

In both naturally occurring and synthetic molecules, indole derivatives are recognized as a privileged structural motif with significant biological activities.^23^ As shown in Fig. 1a, the 3-indolyl sulfide moiety has been widely tested in the drug discovery process. Moreover, incorporating sulfur into aryls by oxidation coupling has proved to be an effective off-DNA synthetic methodology.^24^ The C3-nucleophilicity of electron-rich indole heterocyclic rings enables C–S bond formation with high regioselectivity.^25^ Therefore, we initiated our study with a single-stranded oligonucleotide (Dol-A) conjugated indole-7-carboxylic acid 1a as the model substrate to furnish a sulfide bond with 4-methylbenzenethiol 2a under different oxidative coupling conditions (Table 1). In the beginning, representative oxidant combinations like copper,^26^ vanadium,^27^ and iron,^28^ combined with oxygen were explored, but no desired product was detected (Fig. S2†). Considering that metallic oxidants might inhibit enzymatic procedures in DEL manipulation and lead to DNA damage,^29^ we further examined various non-metallic oxidants including TBHP, potassium persulfate, hydrogen peroxide, iodine, and iodine/BSA.^30^ Iodine alone (entry 6) gave a conversion of 35% with detectable DNA damage (Fig. S5a†). Pleasingly, over 95% conversion was achieved when switching to the iodine/BSA (bovine serum albumin) system (entry 7). To verify the on-DNA C–H oxidative functionalization on the indole ring instead of the heterocycles of DNA bases, we prepared an acetylated Dol-A which was investigated under the same condition as entry 7. No sulfenylation was observed on the acetylated Dol-A (Fig. S5b†). Moreover, the C3 selectivity on the indole ring was elucidated using substituted indoles as well as co-injection validation (Fig. S7†).

**Table 1:** Optimization of sulfenylation using DNA conjugate 1a and thiol 2a

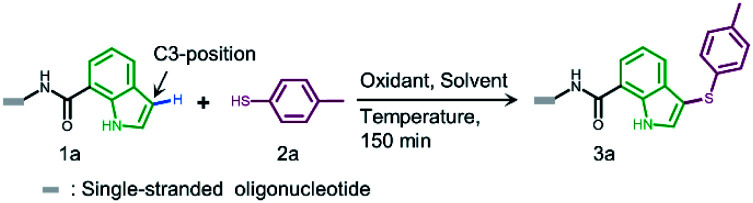				
Entry^a^	Oxidant	Solvent	Temperature (°C)	Conversion^b^ (%)
1	O_2_, Cu(OAc)_2_	MeOH/H_2_O	40	0
2	O_2_, VO(acac)_2_	MeOH/H_2_O	40	0
3	O_2_, FeCl_3_	MeOH/H_2_O	40	0
4	THBP	MeOH/H_2_O	40	0
5	K_2_S_2_O_8_	MeOH/H_2_O	40	0
6	I_2_	MeOH/H_2_O	40	35^c^
7	I_2_/BSA	MeOH/H_2_O	40	>95^d^
8	No oxidant/BSA	MeOH/H_2_O	40	0
9	I_2_/PSA	MeOH/H_2_O	40	95
10	I_2_/HSA	MeOH/H_2_O	40	95
11	I_2_/BSA	MeOH/H_2_O	60	50^c^
12	I_2_/BSA	MeOH/H_2_O	25	85
13	I_2_/BSA	DMA/H_2_O	40	>95
14	I_2_/BSA	DMF/H_2_O	40	>95
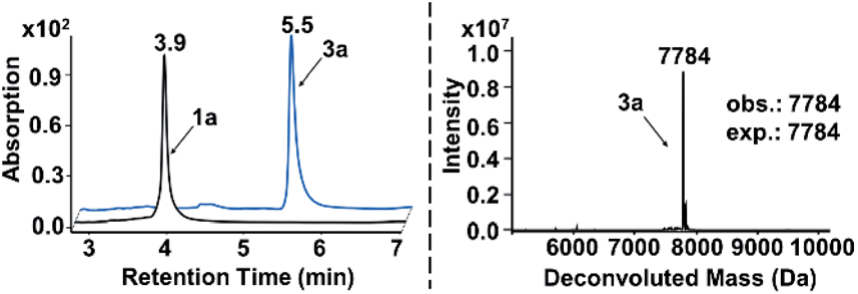				

a The reaction was carried out under the standard conditions in an Eppendorf tube using 1a (0.2 nmol, 6.7 μM), 2a (400 nmol, 13.3 mM), and an oxidant (200 nmol, 6.7 mM) in the respective solvent (30 μL) for 150 minutes.

b Conversion is determined by the method given in ESI Section 2.6.

c DNA damage (depurination) was observed (Fig. S3 and S5a).

d UPLC chromatograms of starting DNA-conjugate 1a and the on-DNA sulfenylation reaction (entry 7) are shown on the left below. The retention time of 1a and 3a is 3.9 min and 5.5 min, respectively; on the right below is the mass spectrum of 3a from on-DNA sulfenylation. (Deconvolution expected mass: 7784 Da; observed mass: 7784 Da).

BSA alone in the absence of iodine gave no desired product (entry 8). We obtained comparable high conversion results by replacing BSA with HSA (human serum albumin) or PSA (porcine serum albumin) (entries 9–10) and we decided to apply BSA as an additive on commercial availability considerations. Higher reaction temperatures led to severe DNA damage (entry 11, Fig. S3†), while lower temperatures yielded slightly lower conversion with iodization by-products (entry 12, Fig. S3†). Next, we examined the effects of cosolvents (DMA/H_2_O and DMF/H_2_O) and found that both cosolvent systems were suitable (entries 13–14). These results would expand applicability since both DMA and DMF could provide good solubility for a wide range of organic substrates which are commonly used as stock solvents for BBs in DEL synthesis. Collectively, we solidified the proof-of-concept study on a novel direct oxidative coupling procedure that could efficiently integrate sulfur into DNA-conjugated indole in the aqueous solution. The recently advanced heterogeneous solid support-mediated on-DNA strategy (like reversible adsorption to solid support, RASS) benefited from high conversion and near anhydrous chemistry.^16*a*,31^ However, they typically consume several milligrams of each BB (typical concentration 100 mM or higher) for one step, which result in expensive DEL synthesis and limitation of valuable BB usage. By contrast, our in-solution homogeneous on-DNA sulfenylation using one order of magnitude lower BB concentration (13 mM under the optimized conditions) may save the cost for large DEL construction. To further explore the feasibility of the optimized condition, scale-up on-DNA sulfenylation was conducted. Gratifyingly, a conversion of 85% at the 5 nmol scale was obtained (Fig. S6†). The robustness of DNA-compatible sulfenylation with a scale-up test would therefore be practicable during the DEL construction.

**Fig. 2:**
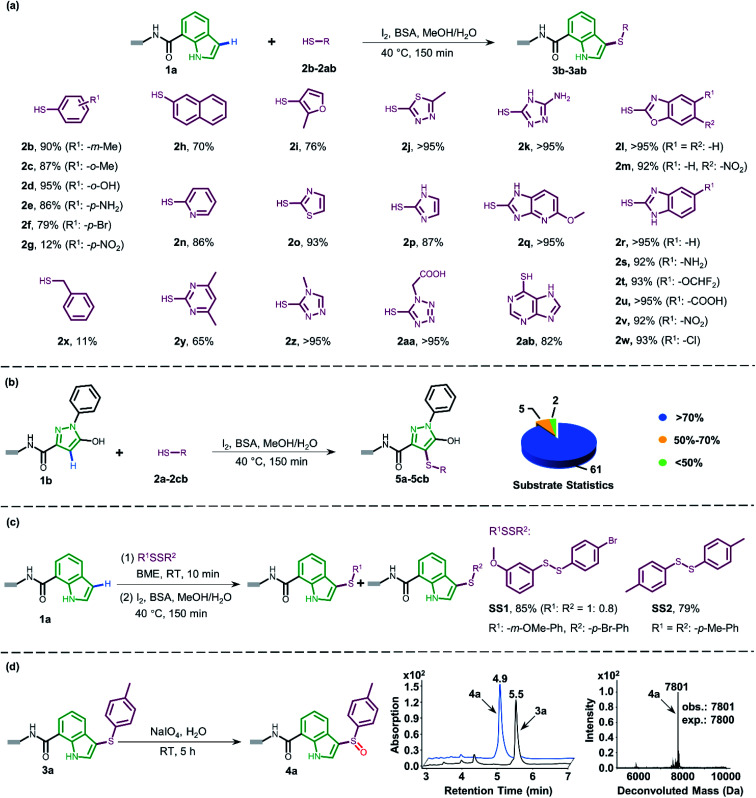
(a) Substrate scope of the thiols in on-DNA indole conjugate sulfenylation and corresponding conversions. (b) On-DNA pyrazole conjugate sulfenylation and statistics of the substrate scope. (c) The substrate scope of disulfides in on-DNA indole conjugate sulfenylation and corresponding conversions. (d) On-DNA sulfoxide formation *via* a sulfide precursor. UPLC chromatograms of starting DNA-conjugate 3a and on-DNA oxidative product 4a are shown on the right. The retention time of 3a and 4a is 5.5 min and 4.9 min, respectively, the latter of which was validated in the mass spectrum (deconvolution expected mass: 7800 Da; observed mass: 7801 Da).

Attracted by the great diversity of thiol derivatives, we then explored the substrate scope and evaluated the generality of our method under the optimized conditions (Fig. 2a). In general, phenyl thiols bearing mono electron-donating substituents (2b–2e) provided the corresponding products with excellent conversion (86–95%), while the introduction of strong electron-withdrawing groups such as nitro (2g) led to poor reactivity. In addition, the reaction went smoothly with naphthalene-(2h) and pyridine-derived (2n) thiols. Also, a variety of 5-membered heterocyclic derivatives including furan (2i), imidazole (2p), thiazole (2o), 1,2,4-triazole (2k and 2z), tetrazole (2aa), and thiadiazole (2j) proved to be compatible with high conversion (76–95%). Six-membered pyrimidine-containing substrate 2y generated a moderate conversion rate which might be attributed to the rather electron-poor pyrimidine ring. To our delight, all tested benzimidazole thiols with electron-donating (2s) as well as electron-withdrawing groups (2t–2w) could be converted to the corresponding final products with high conversion. When benzoxazole derivatives (2l and 2m) were employed, the reaction also proceeded with excellent conversions. Next, we turned to survey alkyl thiols under the optimized conditions, however, a poor conversion yield was observed (benzyl mercaptan 2x with 11% conversion and 2-aminoethanethiol with no detectable product). In summary, of a total of 67 thiols (see ESI Section 6.1†), 56 substrates exhibited a conversion rate >70% and 4 thiols gave a synthetically useful conversion (50–70%). Importantly, the functional group compatibility of halogens (Cl: 2w and Br: 2f),^32^ nitro compounds (2g),^33^ amines (2e and 2k),^34^ and carboxylic acids (2u and 2aa)^35^ provides an opportunity for further on-DNA transformations to expand the chemical diversity of DELs.

Pyrazoles represent another crucial class of heterocyclic moieties in a variety of pharmacologically active compounds. Likewise, we chose DNA-conjugated 1-phenyl-1*H*-pyrazol derivative 1b as the standard substrate for sulfenylation with different thiols (Fig. 2b). In the direct oxidative coupling procedure, C–H bonds are less reactive on the phenyl ring than on electron-rich pyrazolone.^36^ The C4 selectivity on pyrazolone was validated by co-injection experiments (Fig. S8†). Collectively, 61 of the evaluated 68 substrates gained a conversion rate >70%, while only 2 thiols gave conversion yields lower than 50% (see ESI Section 6.2†).

Since thiols are prone to be gradually oxidized into the corresponding disulfides even under ambient storage conditions, it is interesting to investigate whether the disulfide derivative could be employed as a sulfur source for on-DNA C–S bond formation. In an optimized one-pot procedure, sulfur was successfully introduced into DNA-conjugated indole 1a by the reduction of disulfides into *in situ* aryl thiols with BME (β-mercaptoethanol) (Fig. 2c). Representative unsymmetrically substituted thiol SS1 and symmetrical disulfide SS2 achieved conversions of 85% and 79%, respectively, with this protocol (see Scheme S3 in the ESI†). Interestingly, the conversion rates of the two sulfides from the unsymmetrical source correlated with the transformation rates in the corresponding aryl thiols. Moreover, using unsymmetrical disulfide as the sulfur source provides a “one stone, two birds” strategy, where one identical DNA barcode could encode two structural BBs through one reaction, which could further expand DEL diversity.

The sulfoxide group, a typical bioisostere that has been adopted in many bioactive compounds, displays unique interest in drug discovery (Fig. 1a).^37^ DNA-compatible oxidation of sulfide to sulfoxide has been exclusively demonstrated *via* RASS technology by Dawson and Baran.^16*a*^ These results inspired us to pursue the introduction of sulfoxide indirectly through sulfenylation and cascade oxidation in solution. Thereby, we examined different oxidants and found that sodium periodate-mediated oxidation gave the corresponding sulfoxide 4a in high conversion (Fig. 2d, also see Scheme S4 in the ESI† for optimization conditions).

**Fig. 3:**
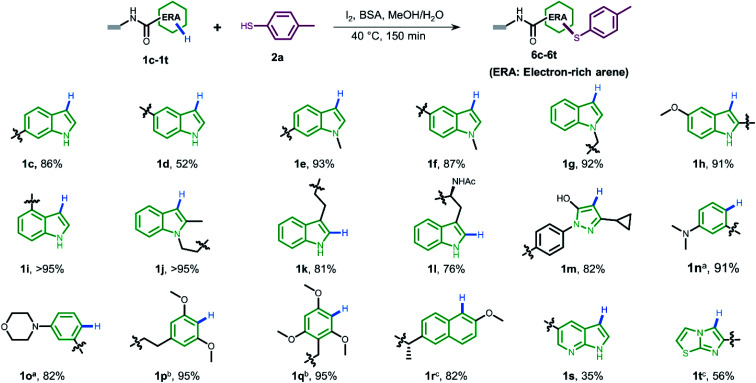
The substrate scope of different DNA-conjugated ERAs in the sulfenylation reaction and the corresponding conversions. Deviation from above reaction condition ^a^ reaction time is 6 h. ^b^ I_2_ (400 nmol, 13.3 mM). ^c^ I_2_ (800 nmol, 26.7 mM).

To gain more understanding about the generality for DEL synthesis, we conducted sulfenylation between 4-methylbenzenethiol 2a and different on-DNA ERAs. As shown in Fig. 3, various indole-based DNA conjugates provided corresponding products with good-to-excellent conversions (1c–1j). It is interesting that C3-substituted indole substrates (1k, 1l) also performed well in the reaction, but the 3-methylindole-2-positioned DNA conjugate gave no product, indicating that these two sites were solely preferred. Additionally, the pyrazolone-based DNA conjugate (1m) displayed good conversions. When applying to more electron-donating aryl rings (1n–1r), the optimal condition was slightly altered, either by increasing the total amount of iodine or extending the reaction time, to reach excellent conversions. Heterocyclic rings such as 1s and 1t resulted in comparatively lower conversion. In summary, 24 out of the total 31 DNA-conjugated ERAs tested in the protocol showed conversions over 60% (see ESI Section 6.3†).

**Fig. 4:**
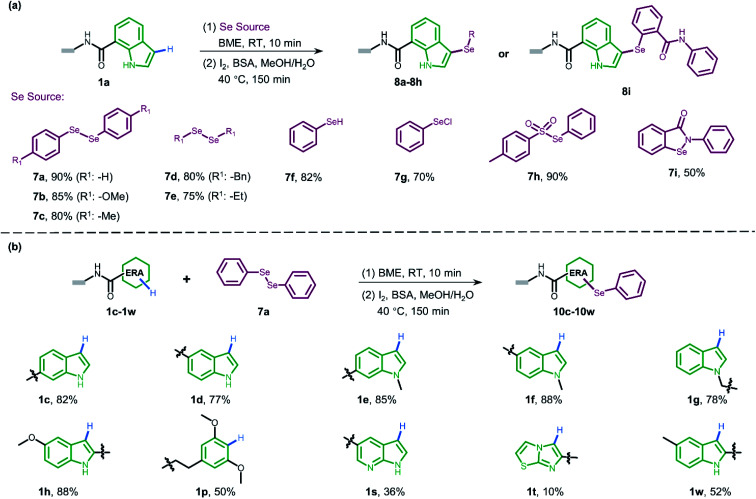
(a) Substrate scope of the selenium sources in the on-DNA indole conjugate sulfenylation reaction and the corresponding conversions. (b) The substrate scope of different DNA-conjugated ERAs in selenylation and corresponding conversions.

Since selenium is in the same main group of sulfur and shares many chemical properties with sulfur, we envisioned that the direct oxidative coupling procedure could work compatibly with selenide reagents. As presented in Fig. 4a, various selenide reagents were studied. Selenols generated from the reduction of diselenides (7a–7e) underwent selenylation with indole DNA-conjugate 1a to form corresponding diaryl (7a–7c) and dialkyl (7d and 7e) selenides in good conversions. Simple phenylselenol (7f), phenylselenyl chloride (7g), and sulfonyl selenol (7h) worked well to afford the desired products efficiently. Ebselen (7i) provided moderate transformation. Moreover, we employed diphenylselenol 7a to explore the scope of various DNA-conjugated ERAs by following the same protocol (Fig. 4b, also see ESI Section 6.4†). In brief, different regio-substituted DNA-indole conjugates (1c–1h, 1w) afforded good-to-moderate conversions. The dimethoxyphenyl ring (1p) also bore selenylation. Like on-DNA sulfenylation, pyrrolopyridine (1s) and pyrrolothiazole (1t) gave poor results. In contrast to the straightforward pyrazolone-derived C–S bond formation, no conversion was observed when pyrazolone derivatives were examined in C–Se formation. In pursuit of wider functionalization, oxidization of on-DNA selenol 8a into selenoxide 9a with sodium periodate was achieved (see Scheme S6†).

Peptidic and peptidomimetic DELs are currently produced in large numbers both by academic laboratories and pharmaceutical companies and are used in selection against pharmacologically interesting protein targets.^21*a*,38*a–i*^ Considering the easy access to carboxylic acids/amines as BBs and well-established on-DNA amide bond formation methods,^39^ it is significant to develop late-stage precise sulfenylation/selenylation on DNA-encoded peptide and peptidomimetic libraries thereby benefitting from high atom economy chemical procedures and broad structural diversity for pharmacological screening.

**Fig. 5:**
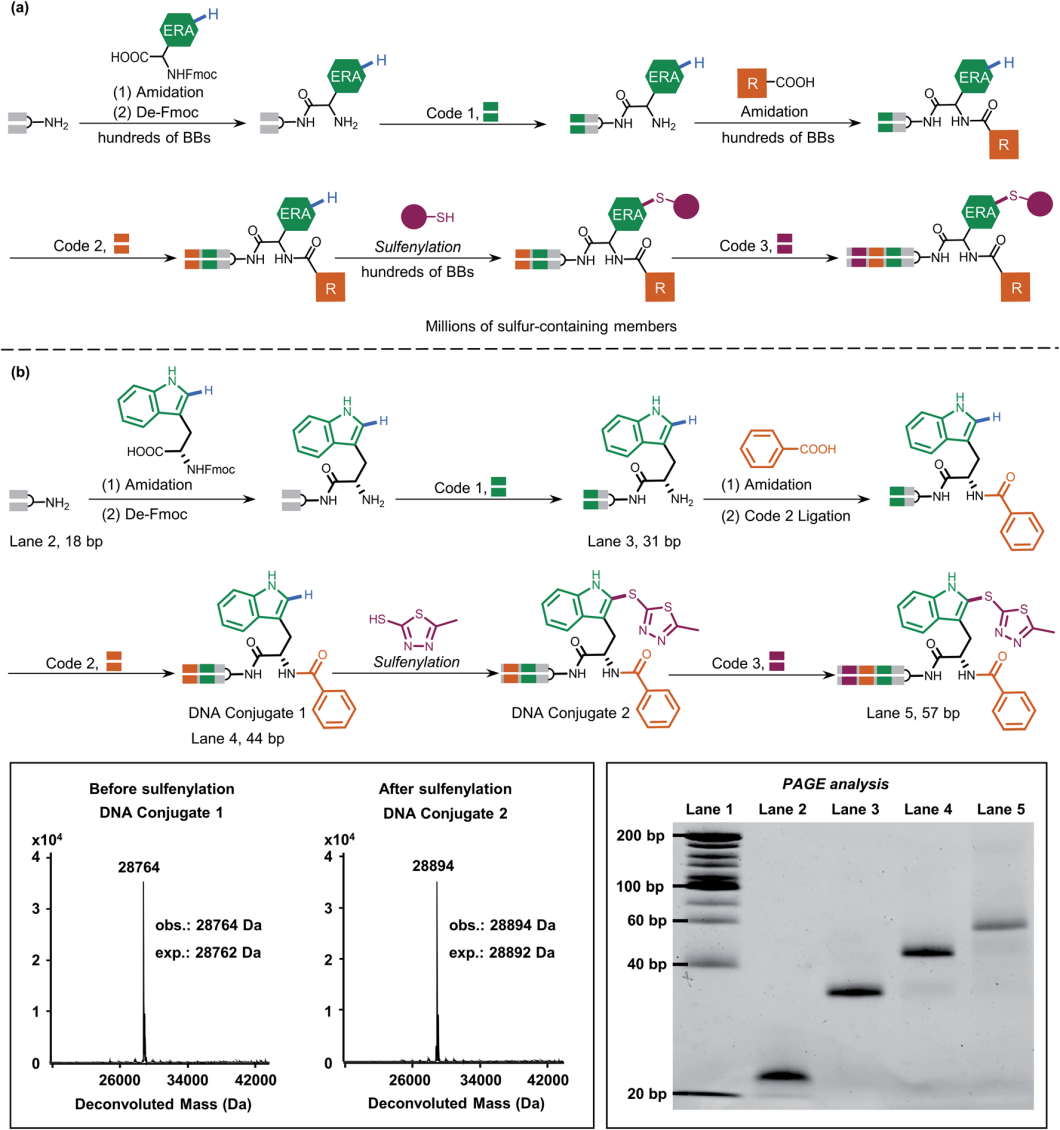
(a) Proposed DEL synthesis by the sulfenylation method in this work would afford sulfur-containing peptide members, highlighting late-stage precise modification on the ERAs. Three rounds are involved in the concept synthesis, where a million members would be generated (round 1 containing hundreds of Fmoc-protected amino acid BBs, round 2 containing hundreds of carboxylic acid BBs, and round 3 containing hundreds of thiol BBs). (b) The mock peptide-like DEL synthesis contains three rounds involving three BBs. The mass data indicate the high efficiency of sulfenylation. PAGE analysis of the demonstrated on-DNA linear synthesis in each round. Lane 1 (DNA ladder), lane 2 (18 bp), lane 3 (31 bp), lane 4 (44 bp), and lane 5 (57 bp).

Inspired by this, we next applied our newly developed methodology to produce a sulfide-containing scaffold for the formal synthesis of a peptide-based focused DEL. The synthesis concept of the DEL is illustrated in Fig. 5a and consisted of three rounds, where the 1st round introduced BBs that contained ERAs and the 2nd round brought electron-neutral or electron-poor aryl BBs. The amide bond is supposed to conjugate each BB for consideration of peptide-based libraries. Late-stage precise sulfenylation constitutes the 3rd round in the synthesis. Notably, only electron-rich arene moieties would be selectively sulfenylated if other electron-deficient arenes or alkyl groups exist (for regioselectivity illustration, Fig. S10 and S14†). Therefore, three rounds of diversity would end with a library collection of millions of sulfur-containing focused dipeptides.

As a proof-of-concept, an 18-bp double-stranded headpiece-primer DNA was initially coupled with tryptophan having an electron-rich indole ring and was then tagged with bar-coding DNA (Fig. 5b). The product was then converted to an amide derivative using benzoic acid, followed by enzymatic ligation to achieve a 44-bp long DNA conjugate. Round 3 included the installation of 5-methyl thiadiazole sulfide at the 2-position of the indole core under the optimized condition, gaining an affordable conversion of 80% (Fig. S13†). The practical conversion (from DNA-conjugate 1 to DNA-conjugate 2 in Fig. 5b) was identified by mass spectrometric analysis in the sequential synthesis. Additionally, all the ligation products after DNA-tagging were confirmed by PAGE analysis. Altogether, the feasibility of our on-DNA sulfenylation for DEL synthesis had been fully demonstrated based on the following aspects: (1) good conversion yields in selective C–H functionalization; (2) high compatibility with enzymatic DNA encoding protocols.

## Conclusion

In summary, we have successfully developed a DNA-compatible in-solution methodology for sulfenylation and selenylation of electron-rich arenes exemplified by excellent conversions of indoles and pyrazoles. Significantly, this transition-metal free method enables C–S and C–Se bond formation without substrate pre-functionalization, affording good-to-excellent conversions. Mild reaction conditions have been applied to over 150 substrates, making this protocol applicable for a wide range of substrates with steric and functional group tolerance. Besides, DNA-compatible sulfides and selenides could be functionalized and turned into the corresponding S- or Se-oxides, which would amplify the structural diversity. These features therefore potentially enable this method to be a powerful tool to rapidly construct sulfur/selenium-containing DNA-encoded chemical libraries, as demonstrated by the efficient synthesis of a mock library. Considering the pharmacological properties of sulfur/selenium-containing electron-rich arenes, this method reported by us will facilitate drug-like DELs featuring sulfur/selenium to fulfill the potential of DEL technology in drug discovery.

## Data availability

We have provided all the experimental data in the ESI.† No computational data is involved in this article.

## Author contributions

S. Yang, G. Zhao, Y. Li, and Y. Li conceived and designed the experiments. S. Yang, G. Zhao, and Y. Sun performed the experiments and analyzed the data. S. Yang, G. Zhao, Y. Gao, G. Zhang, X. Fan, Y. Li, and Y. Li prepared the manuscript.

## Conflicts of interest

There are no conflicts to declare.

## Supplementary Material

SC-013-D1SC06268A-s001
