# Supplementary material for: In-solution direct oxidative coupling for the integration of sulfur/selenium into DNA-encoded chemical libraries
Source: Chem Sci. 2022 Feb 1;13(9):2604–13. doi: 10.1039/d1sc06268a (PMC8890091; doi:10.1039/d1sc06268a)
Supplement: SC-013-D1SC06268A-s001 [file SC-013-D1SC06268A-s001.pdf]

Electronic Supplementary Material (ESI) for Chemical Science.
This journal is © The Royal Society of Chemistry 2022

# Supporting Information:

# In-Solution Direct Oxidative Coupling for the Integration of Sulfur/Selenium into DNA-Encoded Chemical Libraries

Shilian Yang,‡[a] Guixian Zhao,‡[a] Yuting Gao,[a] Yang Sun,[a] Gong Zhang,[ac] Xiaohong Fan,[ab] Yangfeng Li,*[ac] and Yizhou Li*[acd]

Affiliation

[a] *Chongqing Key Laboratory of Natural Product Synthesis and Drug Research, Innovative Drug Research Center, School of Pharmaceutical Sciences, Chongqing University, Chongqing, 401331, P. R. China.*
[b] *Pharmaceutical Department of Chongqing Three Gorges Central Hospital, Chongqing University Three Gorges Hospital, Chongqing, 404100, P. R. China.*
[c] *Chemical Biology Research Center, School of Pharmaceutical Sciences, Chongqing University, Chongqing, 401331, P. R. China.*
[d] *Key Laboratory of Biorheological Science and Technology, Ministry of Education, College of Bioengineering, Chongqing University, Chongqing, 401331, P. R. China.*

∗ Corresponding authors:

*E-mail address*: yizhouli@cqu.edu.cn (Yizhou Li); yfli3@cqu.edu.cn (Yangfeng Li)

## Table of Contents


**1. Abbreviations** **S3**

**2. Materials and General Methods** **S4**

**3. Experimental Procedure for on-DNA Reactions** **S7**

**4. Synthesis of Substrates** **S13**

**5. Structure Confirmation** **S16**

**6. UPLC-MS Spectrum** **S18**

**7. Application of on-DNA Sulfenylation in Mock Peptide-like DEL Synthesis** **S110**

**8. References** **S121**

**1. Abbreviations**

AMA: methylamine (40% wt.)/ ammonium hydroxide (30% wt.), 1:1

BB: boric acid buffer

BBs: building blocks

BME: β-mercaptoethanol

BSA: bovine serum albumin

CPG: controlled-pore glass

DCM: dichloromethane

DIPEA: *N, N*-diisopropylethylamine

DMA: *N, N*-dimethylacetamide

DMT-MM: 4-(4, 6-dimethoxy-1, 3, 5-triazin-2-yl)-4-methylmorpholinium chloride

HFIP: 1, 1, 1, 3, 3, 3-hexafluoro-2-propanol

HP-P: headpiece primer

HSA: human serum albumin

MW: molecular weight

NHS: *N*-hydroxysuccinimide

PAGE: polyacrylamide gel electrophoresis

PB: phosphate buffer

PMDETA: 2, 5, 8-trimethyl-2, 5, 8-triazanonane

PSA: porcine serum albumin

TBE: tris-borate-EDTA

TEAA: triethylammonium acetate

TEA: trimethylamine

TLC: thin layer chromatography

TIC: total ion chromatogram

UPLC-MS: Ultra performance liquid chromatography-mass spectrum

UV: ultraviolet

## 2. Materials and General Methods

### 2.1 Chemical and biological materials

DMA (Cas: 127-19-5, Catalog: 271012) was purchased from Sigma-Aldrich. DMSO (Cas: 67-68-5, Catalog: JJHG000170), DCM (Cas: 75-09-2, Catalog: JJHG000282) MeCN (Cas: 75-05-8, Catalog: JJSM000738), MeOH (Cas: 67-56-1, Catalog: JJHG000015), and iodine (Cas: 7553-56-2, Catalog: I0604) were purchased from Kelong. BSA (Cas: 9048-46-8, Catalog: A6020), HSA (Cas: 70024-90-7, Catalog: A6230) were purchased from Biotopped. PSA (Catalog: L3530-00) was purchased from Guangzhou Ruite biotechnology. Cestbon water was used in all the reactions unless otherwise stated. Chemical building blocks and reagents were purchased from a variety of commercial suppliers.

### 2.2 DNA materials

Two types of DNA were used in this work and their structures are shown below. ssDNA **Dol-A** (5′/5Amino-modifier/- GGAGCG CCACATTACTGTGACTTA -3′, MW: 7519) was synthesized via DNA synthesizer; headpiece dsDNA (5′/5Phos/GAGTCA/iSp9/iUniAmM/iSp9/TGACTCCC -3′) and code sequence were received from HitGen Ltd. **Dol-A** was stored in aqueous solution (1 mM), and headpiece DNA was stored in aqueous solution (0.5 mM).

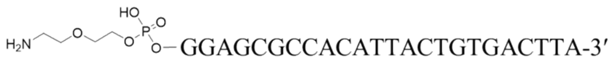


(a)

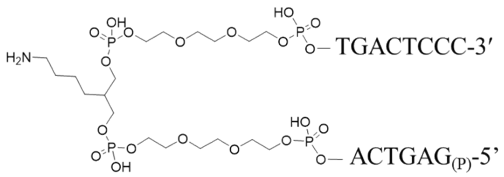


(b)

**Fig. S1.** Structure of **Dol-A** (a) and headpiece DNA (b).

## 2.3 General methods for monitoring and characterization of small molecules

Reactions were monitored by TLC or UPLC-MS. TLC analysis was visualized by using general staining reagents or UV light (λ = 254 nm). Novel synthetic compounds were characterized by $^1$H-NMR, $^{13}$C-NMR, and HRMS. NMR spectrum were recorded on Agilent 400 MHz spectrometer using residual non-deuterated solvent (DMSO-$d_6$) as the internal standard. Multiplicity abbreviations are as follows: s = singlet, brs = broad singlet, d = doublet (dd = doublet of doublets), t = triplet, q = quartet, m = multiplet. Unless otherwise noted, all deuterated solvents were purchased from Adamas.

## 2.4 Preparation of Dol-A

Dol-A was synthesized on Applied Biosystems 3400 DNA synthesizer using standard phosphoramidite protocols and purified by reversed-phase HPLC with aqueous 0.1 M TEAA/$CH_3CN$ gradient on Waters 1575EF systems (Eclipse-XDB C18, 5 μM, 9.4 × 250 mm). After solid-phase synthesis, oligonucleotides were cleaved by an AMA cleavage solution (55 ºC, 1 h) over dry bath. The cleaved oligonucleotide was then concentrated and neutralized with TEAA (2 M) followed by HPLC purification using a gradient of acetonitrile (5-80%) from 5 min to 25 min. For all the non-standard phosphoramidites (dA-, dG-, dC- and dT- phosphoramidites), coupling time was modified to 600 sec. 5′-Amino-modifier was prepared in our laboratory. Oligonucleotides were quantified by BioTek Epoch UV-Vis spectrometer based on extinction coefficients at 260 nm and characterized by Agilent UPLC-MS. All DNA sequences are written in 5′- to 3′- orientation unless otherwise noted.

## 2.5 General methods for DNA conjugate purification

General method for ethanol precipitation: to the DNA reaction mixture was added 10% (v/v) NaOAc (3 M, pH 5.0) solution and 3 times the volume of cold (-20 °C) ethanol subsequently. The solution was maintained at -80 °C for over 2 h and then centrifuged at 13,500 rpm for 30 min at 4 °C. The supernatant was discarded and the pellet was rinsed once with cold 70% ethanol. After centrifuging at 13,500 rpm for

another 10 min at 4 °C, the supernatant was discarded and the pellet was dried by a speedvac. The recovered sample was then dissolved in an appropriate buffer for subsequent analysis or experiments. Eppendorf 5424R centrifuge is used for ethanol precipitation of all the DNA samples.

General method for HPLC purification: preparative reversed-phase high-pressure liquid chromatography (RP-HPLC) for the DNA conjugate was performed on a Waters 1575EF Series with a reversed-phase HPLC column (Eclipse-XDB C18, 5 µM, 9.4×250 mm) using eluent A (100 mM TEAA in $H_2O$) and eluent B (100 mM TEAA in 80% MeCN) with gradient: 10% B (0 to 1 min), 10% to 30% B (1 to 11min), 30% to 100% B (11 to 11.1 min), 100% B (11.1 to 12 min), 100% to 10% B (12 to 12.1 min), 10% B (12.1 to 16 min). The fractions containing the product were combined and lyophilized overnight.

**2.6 General methods for DNA analysis**

Analysis of on-DNA reactions by UPLC-MS: 10-20 pmol of the samples was injected into a reversed-phase UPLC column (Agilent, AdvanceBio Oligonucleotide, C18, 2.1×50 mm, 2.7 µm) at flow rate of 0.3 mL/min with gradient: 5% to 17% B (0 to 1 min), 17% to 30% B (1 to 8 min), 30% to 90% B (8 to 8.1 min), 90% B (8.1 to 9 min), 90% to 5% B (9 to 9.1 min), 5% B (9.1 to 10 min) (Solvent A: 200 mM HFIP and 8 mM TEA in $H_2O$; Solvent B: MeOH). The effluent was detected by absorbance at 260 nm, and analyzed on Agilent TOF (6230 B) in negative ion mode.

Conversion calculation: the conversion of on-DNA product was determined from UV absorbance trace (260 nm) peak area by using the equation: conversion% = UV (product)/UV (total DNA recovered), ignoring the UV coefficient difference among all the DNA products and assuming 100% DNA recovery. Any non-oligo material that had an absorbance at UV 260 nm was subtracted from the conversion calculation.[1]

Analysis of molecular mass: observed m/z could be calculated as $m/z = (m - z)/z$. BioConfirm software (Agilent, v10.0) was used to deconvolute the multiple charge states.

## 3. Experimental Procedure for on-DNA Reactions

### 3.1 On-DNA amidation

**Scheme S1.** Synthesis of compound **1a**.

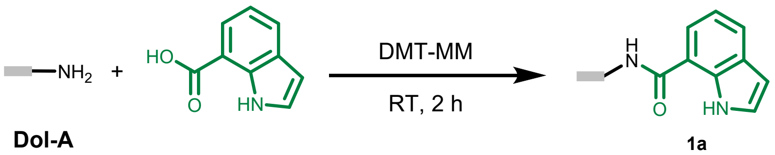


To the solution of amino-modified oligonucleotide **Dol-A** (10 µL, 1 mM) in sodium borate buffer (250 mM, pH 9.4) was added indole-7-carboxylic acid (10 µL, 200 mM in DMA) and DMT-MM (10 µL, 200 mM in dd$H_2O$), and then mixed by vortex. The reaction was agitated at 25 ºC for 2 h, followed by ethanol precipitation. The product was purified by preparative HPLC and quantified by UV absorption at 260 nm to give a conversion of 90%. The conjugate was characterized by UPLC-MS. Deconvoluted molecular mass: predicted: 7662; found: 7662.

### 3.2 On-DNA sulfenylation

#### 3.2.1 General methods for on-DNA sulfenylation by using thiols

**Scheme S2.** Synthesis of compound **3a**.

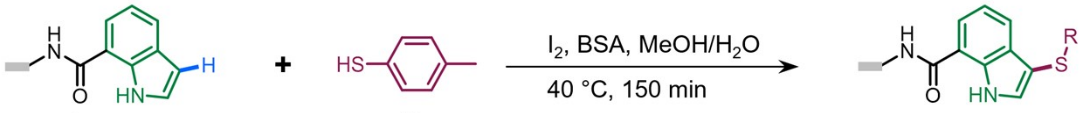


DNA conjugate **1a** (4 µL, 0.05 mM in $H_2O$, 0.2 nmol) and 14 µL $H_2O$ in a 1.5 mL Eppendorf tube were equipped and quickly added with 4-methylbenzenethiol (4 µL, 100 mM in MeOH, 400 nmol) and iodine (4 µL, 50 mM in MeOH, 200 nmol). Then the solution was mixed by vortex and added with BSA (4 µL, 0.75 mM in $H_2O$, 0.3 nmol). The mixture was vortexed again, centrifuged, and heated at 40 °C for 150 min. The product was obtained by ethanol precipitation and characterized by UPLC-MS. (conversion: 98%)

All the DNA-indole conjugates and thiol reactions were performed using the

method above. During the reactions, attention must be paid to: sequence of adding reagents should not be reversed, and the time of adding reagents should be controlled within one min. DMA is suggested to help dissolve the thiols with poor solubility in methanol.

### 3.2.2 Optimization of the model reaction

(1) Optimization of sulfenylation by using different oxidants

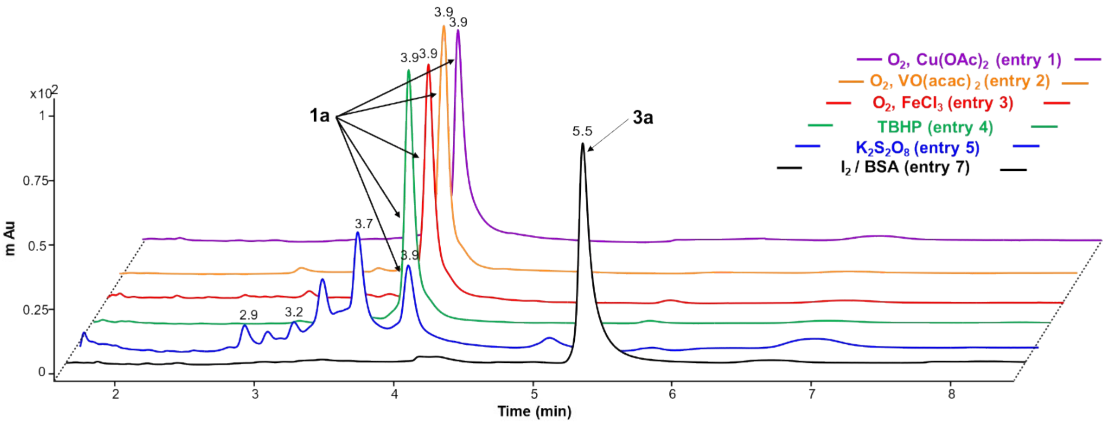


**Fig. S2.** The HPLC chromatography showed that no desired product was detected in the conditions that contained transition metals (entries 1-3), TBHP (entry 4) and $K_2S_2O_8$ (entry 5).

(2) Optimization of sulfenylation under different temperatures

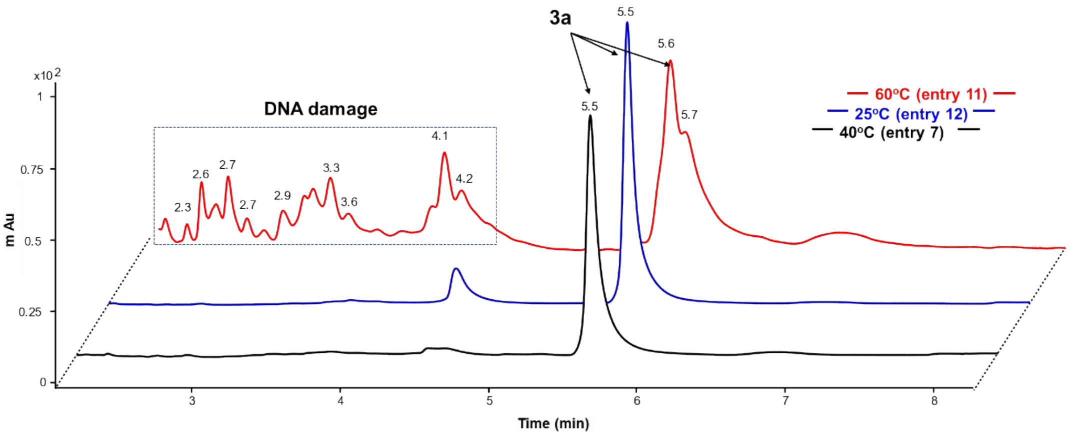


**Fig. S3.** The HPLC chromatography showed that high temperature led to obvious DNA damage (entry 12) and low temperature gave a lower conversion (entry 11).

(3) Optimization of sulfenylation by using different serum albumins.

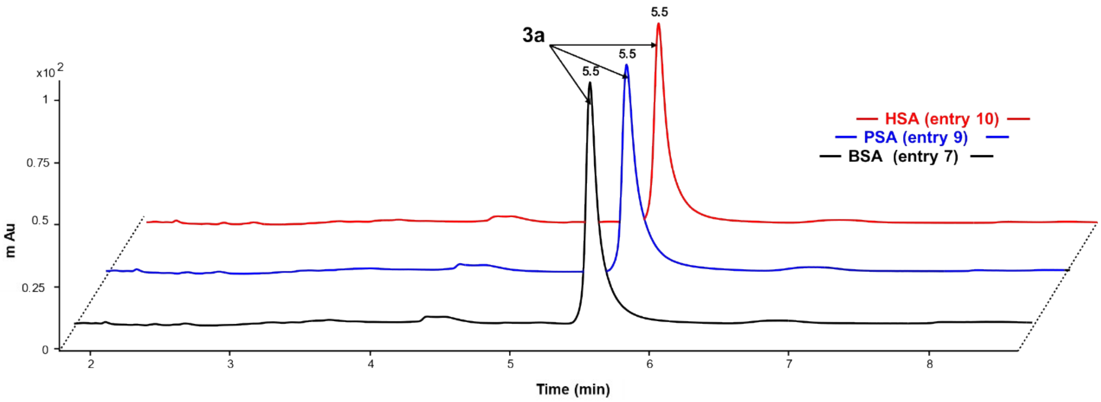


**Fig. S4.** The HPLC chromatography showed that similar high conversions were obtained in the three conditions (HSA, entry 10; PSA, entry 9; BSA, entry 7).

(4) Control experiments

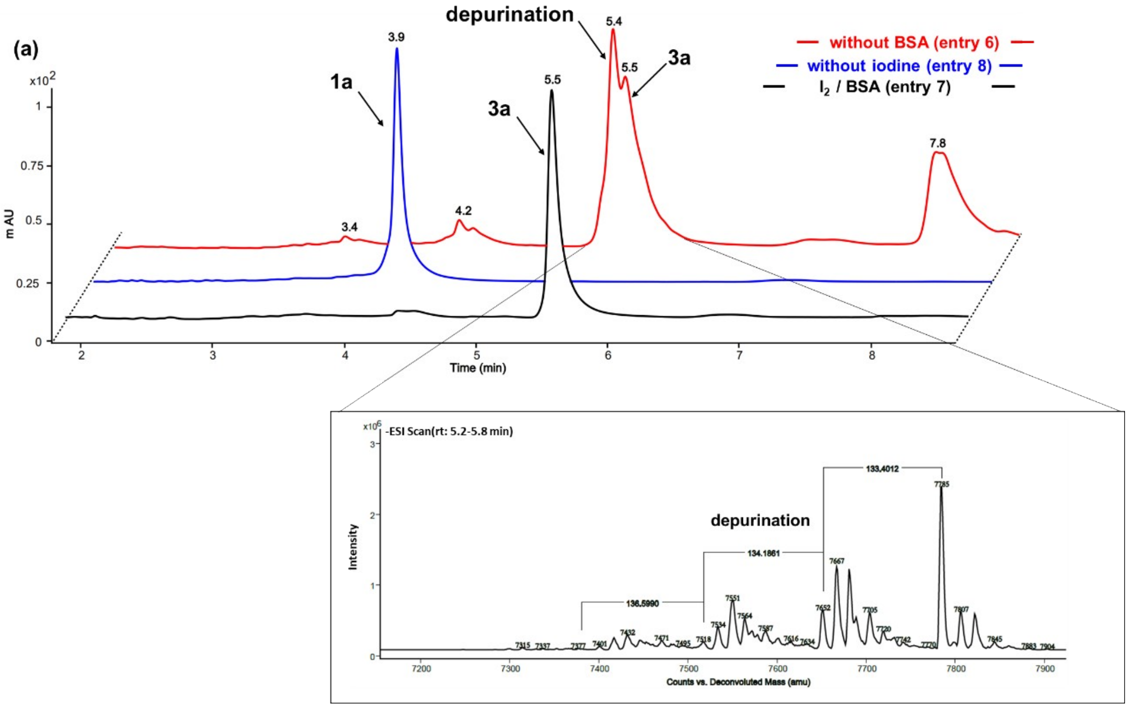

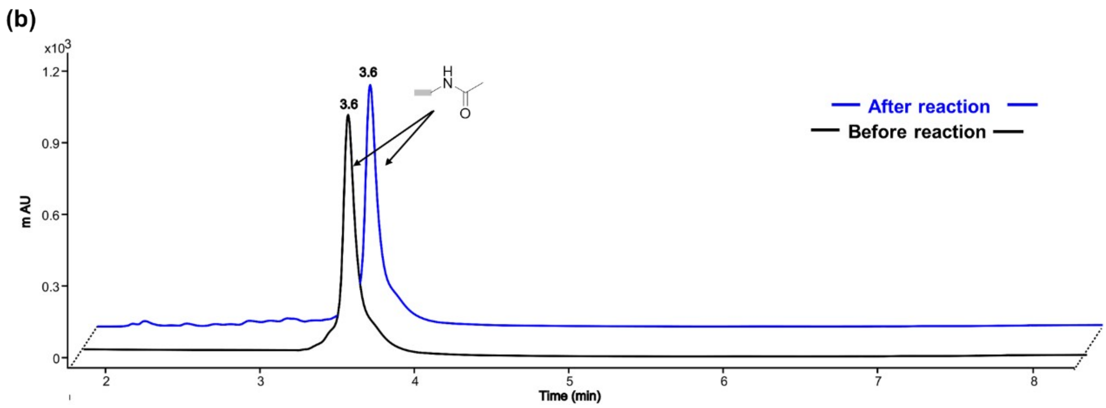


**Fig. S5.** (a) Compared to the standard condition (entry 7), deprivation of BSA afforded DNA damage (entry 6, depurination shown in the box), and deprivation of iodine gave no desired product (entry 8). (b) Compound **1a** was replaced with acetylated Dol-A conjugate to undergo the condition (entry 7), and no reaction occurred. The result indicated no sulfenylation onto the heterocyclics of the DNA base pairs.

### 3.2.3 Scale-up reaction for sulfenylation

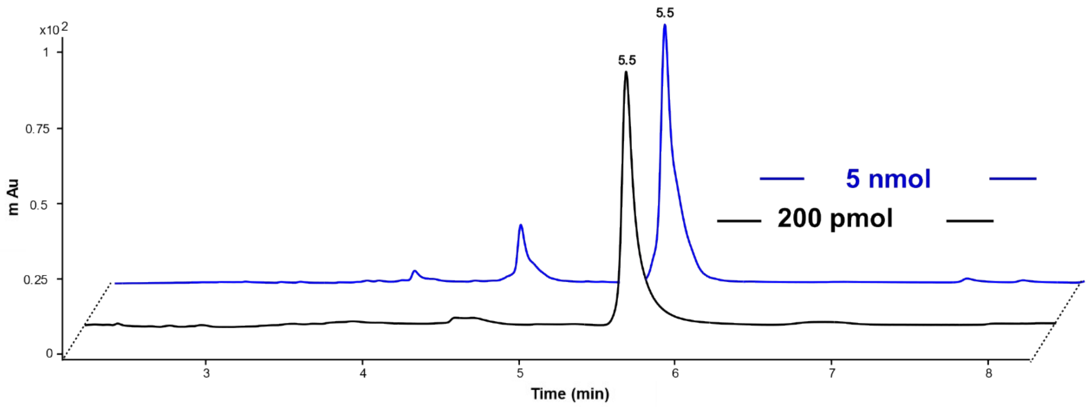


**Fig. S6.** DNA conjugate **1a** (5 µL, 1 mM in $H_2O$, 5 nmol) and $H_2O$ (85 µL) in the Eppendorf tube were equipped and quickly added 4-methylbenzenethiol (20 µL, 100 mM in MeOH, 2 µmol) and iodine (20 µL, 50 mM in MeOH, 1 µmol). Then the solution was mixed by vortex and added with BSA (10 µL, 0.5 mM in $H_2O$, 5 nmol). The mixture was vortexed again, centrifuged, and heated at 40 °C for 150 min. The product was obtained by ethanol precipitation and characterized by UPLC-MS. **3a** conversion: 85%. The UPLC chromatography of 200 pmol scale reaction was used as the comparison.

### 3.2.4 General methods for sulfenylation by using disulfides

**Scheme S3.** Sulfenylation of **1a** with disulfides.

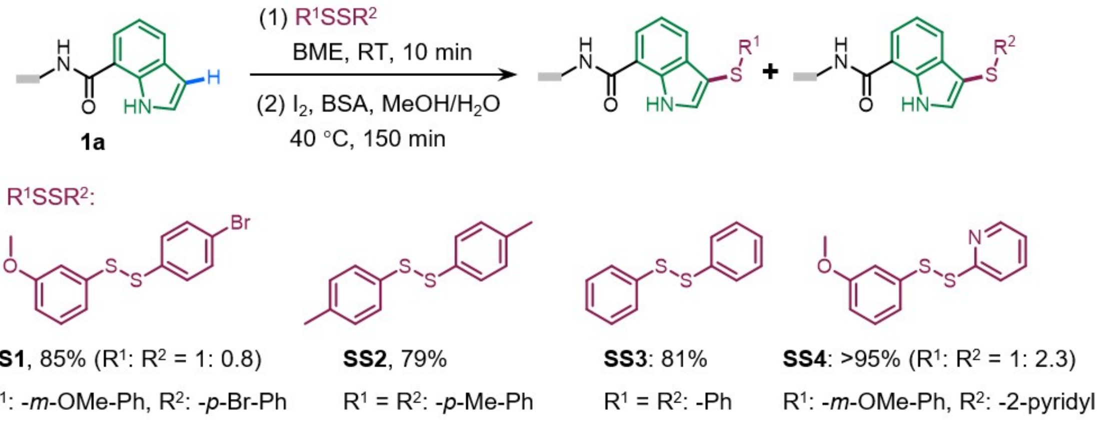


Disulfide (10 µL, 200 mM in DMA) and BME (10 µL, 200 mM in DMA) were mixed at 25 °C for 10 min. Then, to a solution of DNA conjugate **1a** (4 µL, 0.5 mM in $H_2O$, 0.2 nmol) in 12 µL $H_2O$ was added reaction mixture (4 µL) from premixed solution and iodine (4 µL, 50 mM in MeOH, 200 nmol), and mixed by vortex, followed by adding BSA aqueous solution (4 µL, 0.75 mM in $H_2O$, 0.3 nmol). The mixture was vortexed, centrifuged, and heated at 40 °C for 150 min. The product was obtained by ethanol precipitation as described above and characterized by UPLC-MS.

### 3.2.5 On-DNA sulfoxide formation

**Scheme S4.** Optimization of on-DNA sulfoxide formation.

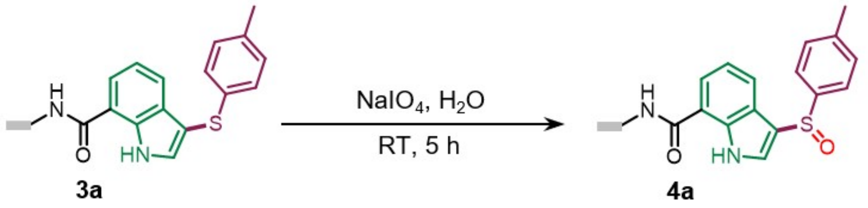


| Entry | Oxidant | Conversion (%) |
|---|---|---|
| 1 | $H_2O_2$ | 0 |
| 2 | TBHP | 0 |
| 3 | $NaIO_4$ | 95 |
| 4 | $K_3Fe(CN)_6$ | 0 |

To the solution of DNA conjugate **3a** (1 µL, 2 mM in $H_2O$, 0.2 nmol) in 45 µL $H_2O$ was added $NaIO_4$ (5 µL, 100 mM in $H_2O$, 500 nmol). The mixture was vortexed, centrifuged, and allowed to incubate at 25 °C for 5 h. The product was obtained by ethanol precipitation and characterized by UPLC-MS.

### 3.3 On-DNA selenylation

**Scheme S5.** Synthesis of compound **8a**.

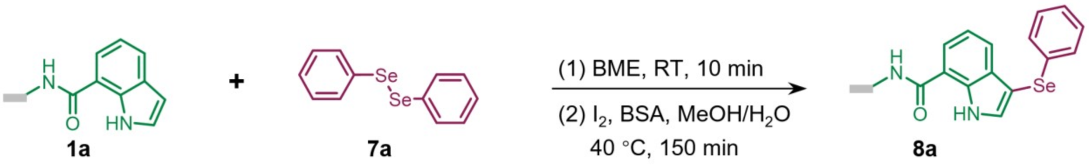


Diselenide (10 µL, 200 mM in DMA) and BME (10 µL, 200 mM in DMA) were mixed at 25 °C for 10 min. To a solution of **1a** (4 µL, 0.5 mM in $H_2O$, 0.2 nmol) in 12 µL $H_2O$, was added crude solution (4 µL) from premixed solution and iodine (4 µL, 50 mM in MeOH, 200 nmol), and mixed by vortex. To a DNA reaction mixture was added BSA aqueous solution (4 µL, 0.75 mM in $H_2O$, 0.3 nmol). The mixture was vortexed, centrifuged, and heated at 40 °C for 150 min. The product was obtained by ethanol precipitation and characterized by UPLC-MS.

### 3.4 On-DNA selenoxide formation

**Scheme S6.** Synthesis of compound **9a**.

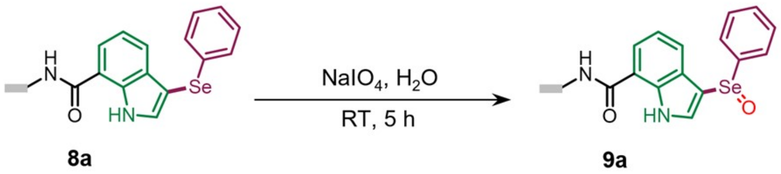


To the solution of DNA conjugate **8a** (1 µL, 2 mM in $H_2O$, 0.2 nmol) in 45 µL $H_2O$ was added $NaIO_4$ (5 µL, 100 mM in $H_2O$, 500 nmol). The mixture was vortexed, centrifuged, and allowed to incubate at 25 °C for 5 h. The product was obtained by ethanol precipitation and characterized by UPLC-MS.

## 4. Synthesis of Substrates

**Scheme S7.** Synthesis of **P1** and **P2.**

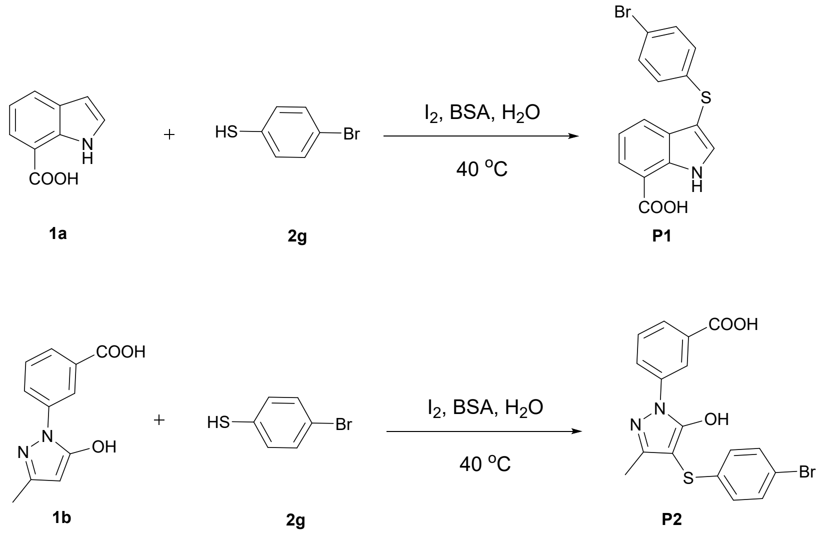


To the solution of **1a** (0.5 mmol, 80 mg), 4-bromothiophenol (0.6 mmol, 112 mg) in water (1 mL) were added BSA (25 mg) and iodine (0.3 mmol, 76 mg). The reaction was stirred at 40 °C and monitored by TLC. Upon completion, saturated sodium thiosulfate was added to the reaction and the mixture was extracted with ethyl acetate. Organic layer was combined, dried over $Na_2SO_4$, filtered and concentrated in vacuo to give a residue, which was purified by column chromatography on silica gel to afford product **P1** (89 mg, yield: 52%).[2] $^1$H NMR (400 MHz, DMSO-$d_6$) δ 13.24 (s, 1H), 11.67 (s, 1H), 7.84 (d, $J$ = 7.4 Hz, 1H), 7.75 (d, $J$ = 2.3 Hz, 1H), 7.65 (d, $J$ = 7.8 Hz, 1H), 7.39 (d, $J$ = 8.4 Hz, 2H), 7.20 (t, $J$ = 7.6 Hz, 1H), 6.95 (d, $J$ = 8.4 Hz, 2H). $^{13}$C NMR (101 MHz, DMSO-$d_6$) δ 167.5, 138.5, 135.4, 134.1, 131.8, 129.9, 127.4, 125.1, 123.7, 120.0, 117.8, 114.9, 99.6. HRMS (ESI) exact mass predicted for $C_{15}H_{10}BrNO_2S$ ($[M-H]^-$): 345.9543; found: 345.9536.

By following the reaction, product **P2** was obtained (108 mg, yield: 54%). $^1$H NMR (400 MHz, DMSO-$d_6$) δ 13.20 (s, 1H), 12.58 (s, 1H), 8.34 (s, 1H), 8.03 (d, $J$ = 8.0 Hz, 1H), 7.84 (d, $J$ = 7.5 Hz, 1H), 7.61 (s, 1H), 7.47 (d, $J$ = 8.3 Hz, 2H), 7.04 (d, $J$ = 8.0 Hz, 2H), 2.13 (s, 3H). $^{13}$C NMR (101 MHz, DMSO-$d_6$) δ 166.9, 138.0, 131.9, 131.7, 129.5, 127.0, 126.4, 124.4, 121.0, 117.8, 12.4. HRMS (ESI) exact mass predicted for $C_{17}H_{13}BrN_2O_3S$ ($[M-H]^-$): 404.9757; found: 404.9744.

$^{1}$H-NMR of compound **P1**

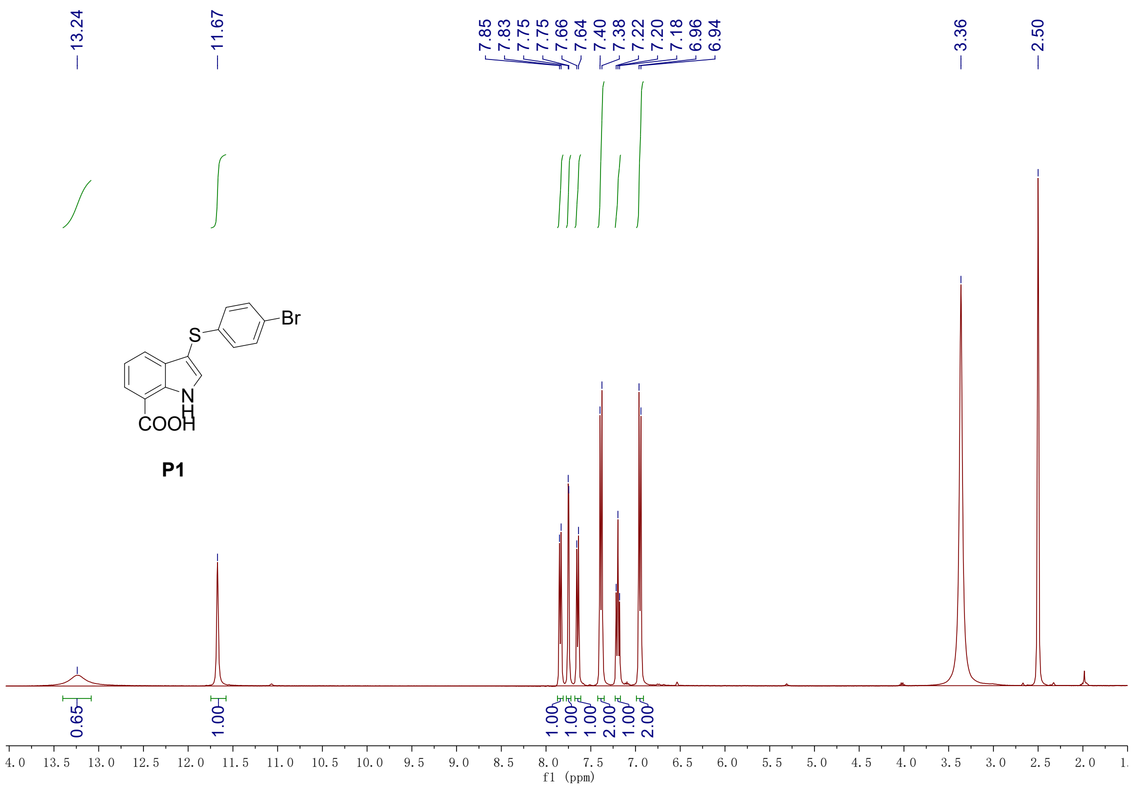


$^{13}$C-NMR of compound **P1**

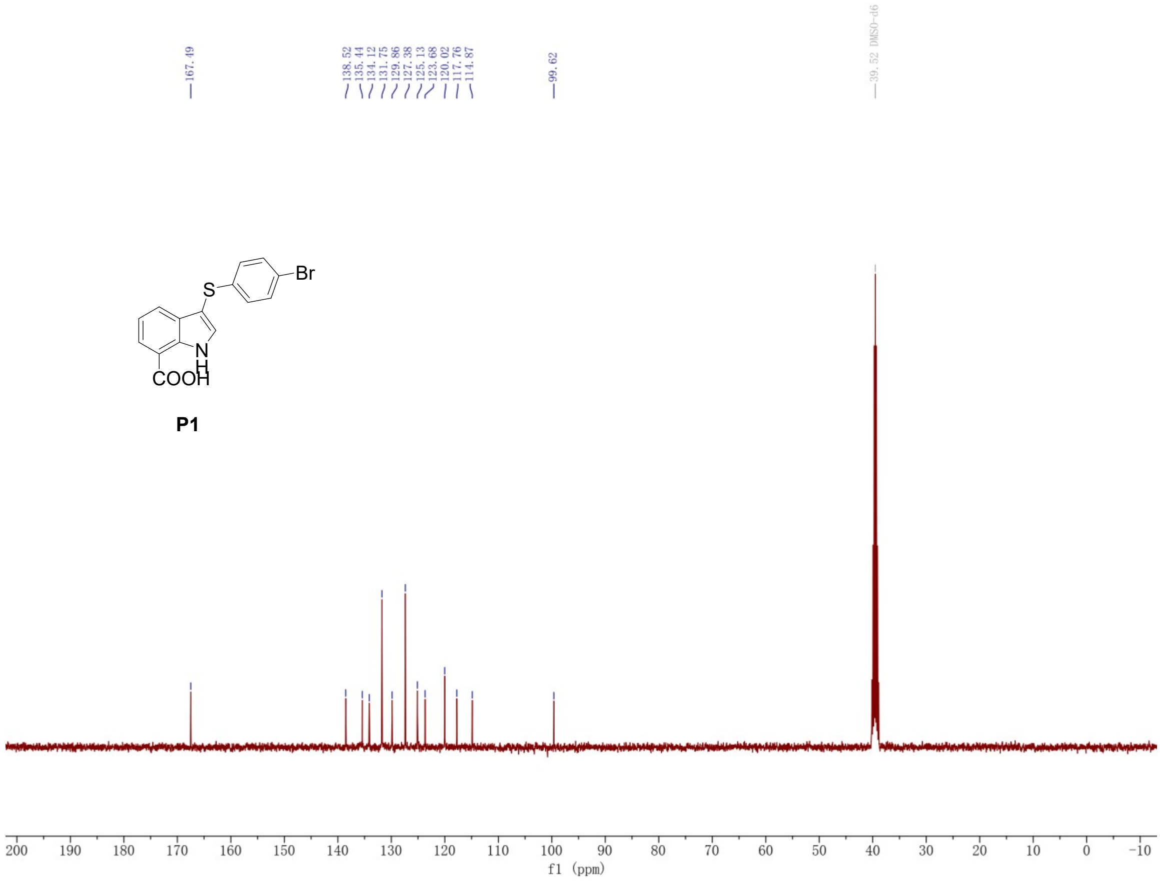


$^1$H-NMR of compound **P2**

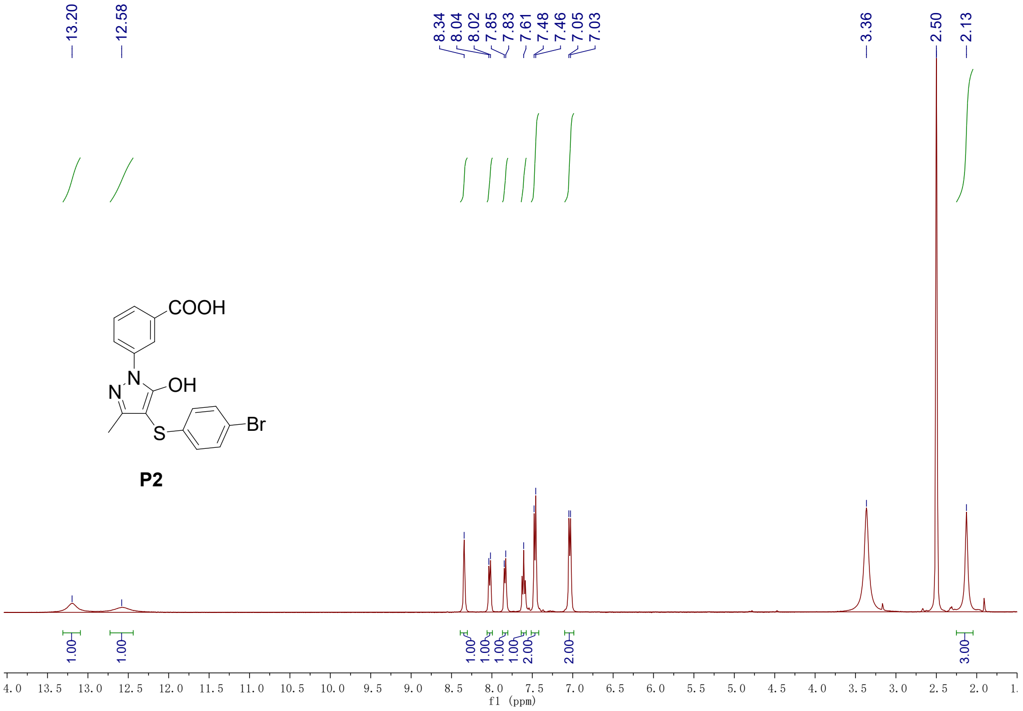


$^{13}$C-NMR of compound **P2**

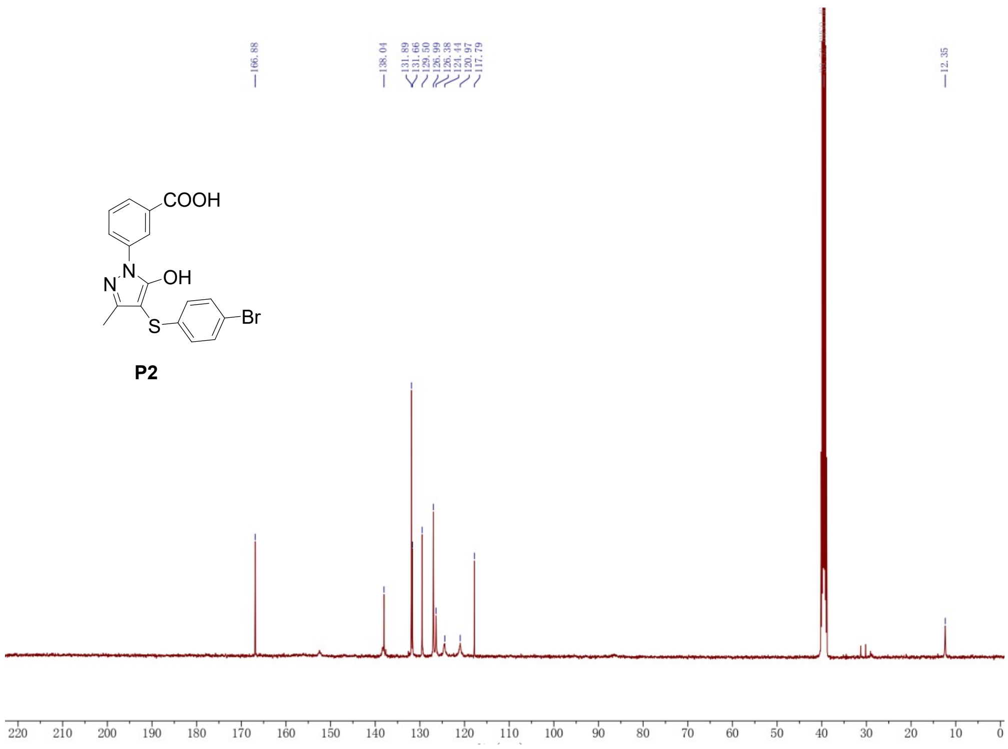
166.88
138.04
131.89
131.66
129.50
126.99
126.38
124.44
120.97
117.79
12.35
COOH
N
N
OH
S
Br
P2
220 210 200 190 180 170 160 150 140 130 120 110 100 90 80 70 60 50 40 30 20 10 0

## 5. Structure Confirmation by Co-injection Experiment

### 5.1 Sulfenylation of DNA-conjugate indole

**Scheme S8.** The compound **3g** was synthesized via two routes.

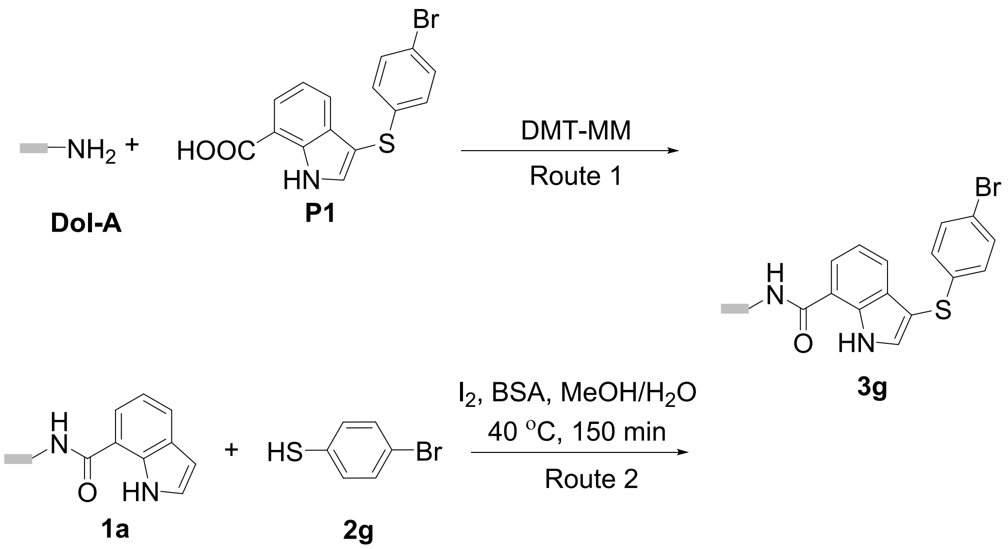


Above was route 1 where amide coupling between **Dol-A** and **P1** afforded **3g**, and below was route 2 where the on-DNA sulfenylation of **1a** with **2g** generated **3g**.

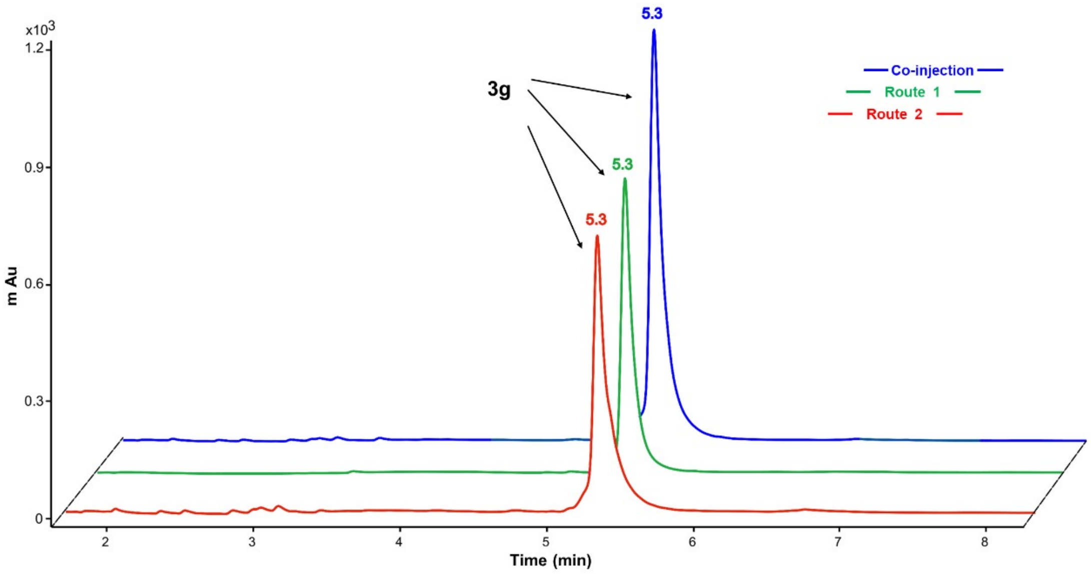


**Fig. S7.** Co-injection experiment of **3g** from two independent synthetic routes in Scheme S8. HPLC chromatography showed that the peak from the co-injection (curve in blue) shared the same retention time with the other two peaks (**3g** from route 1, curve in green; **3g** from route 2, curve in red).

### 5.2 Sulfenylation of DNA-conjugate pyrazole

**Scheme S9.** The compound **5g** was synthesized via two routes.

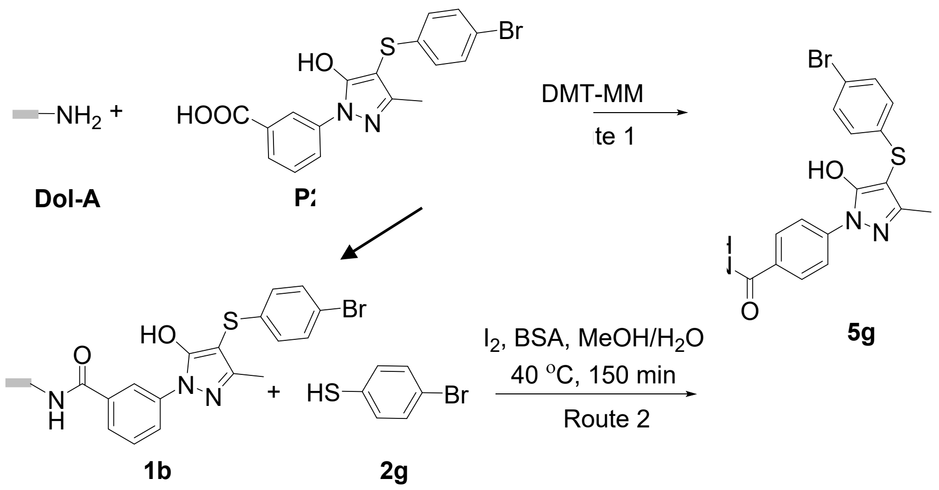


Above was route 1 where amide coupling between **Dol-A** and **P2** afforded **5g**, and below was route 2 where the on-DNA sulfenylation of **1b** with **2g** generated **5g**.

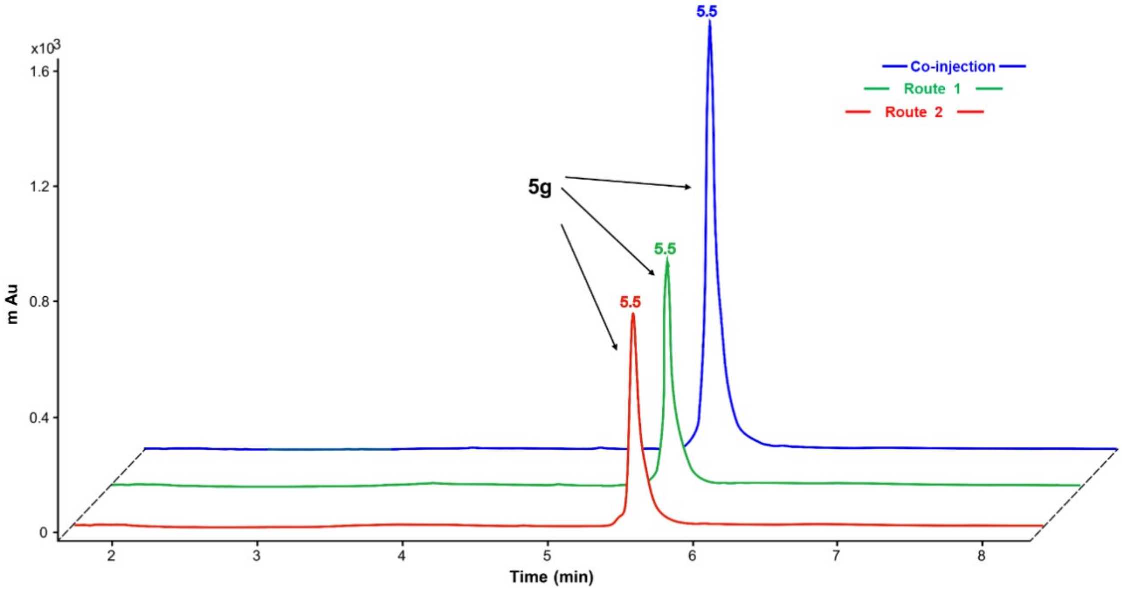


**Fig. S8.** Co-injection experiment of **5g** from two independent synthetic routes in Scheme S9. HPLC chromatography showed that the peak from the co-injection (curve in blue) shared the same retention time with the other two peaks (**5g** from route 1, curve in green; **5g** from route 2, curve in red).

## 6. UPLC-MS Spectrum

### 6.1 HPLC spectrum of DNA-conjugated indole 3a with thiols

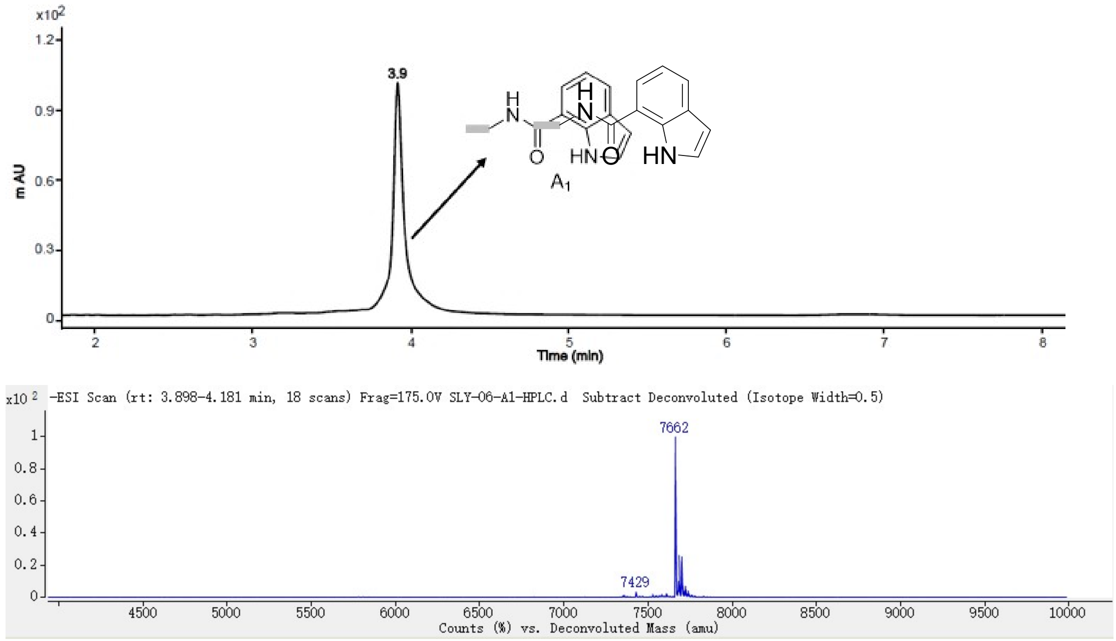


**Fig. S9.** Spectrum of on-DNA Product **3a**.

UPLC chromatograph and deconvoluted MS of **3b**.

**Conversion: 90%**

**Calculated Mass: 7784 Da; Found Mass: 7784 Da**

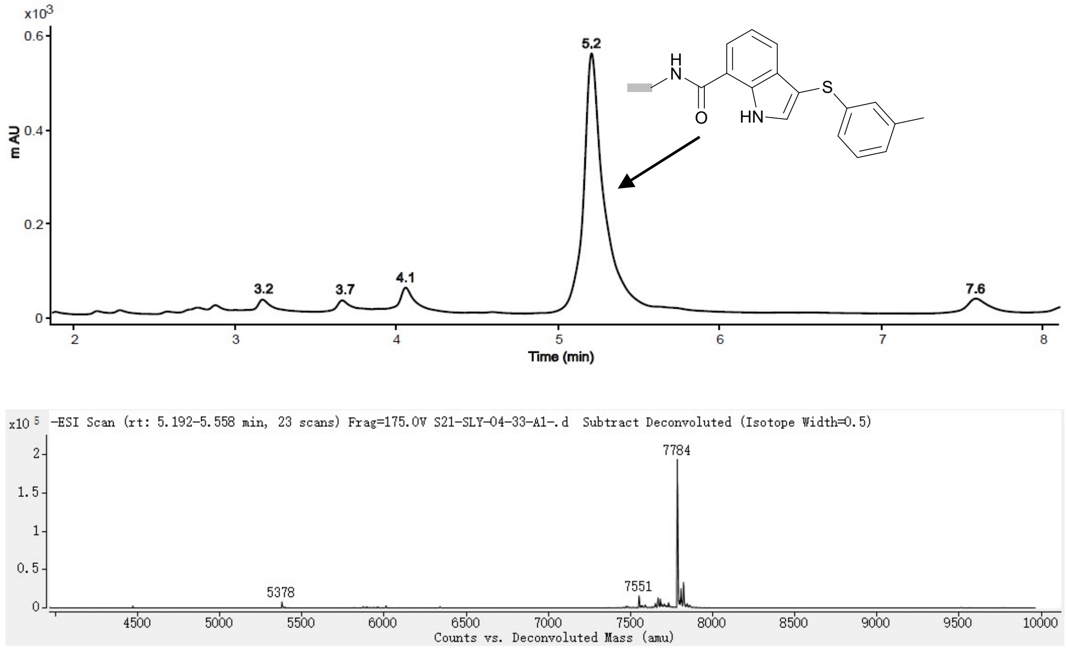


UPLC chromatograph and deconvoluted MS of **3c**.

**Conversion: 87%**

**Calculated Mass: 7784 Da; Found Mass: 7784 Da**

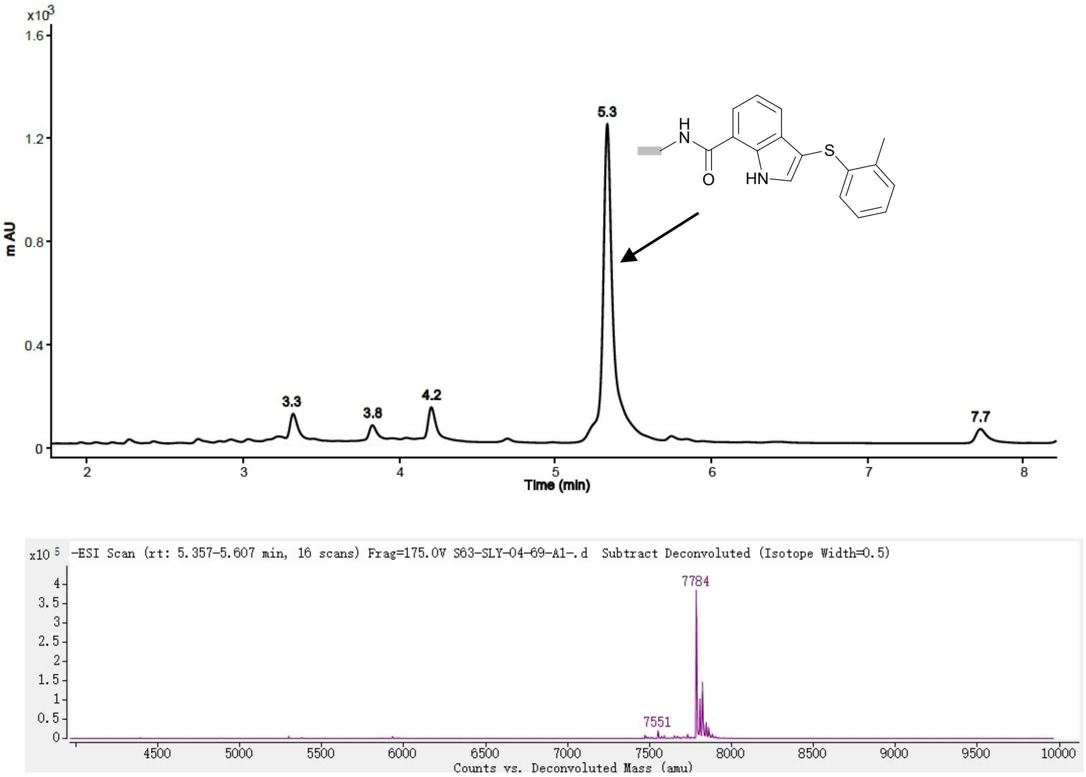


UPLC chromatograph and deconvoluted MS of **3d**.

**Conversion: 95%**

**Calculated Mass: 7786 Da; Found Mass: 7788Da**

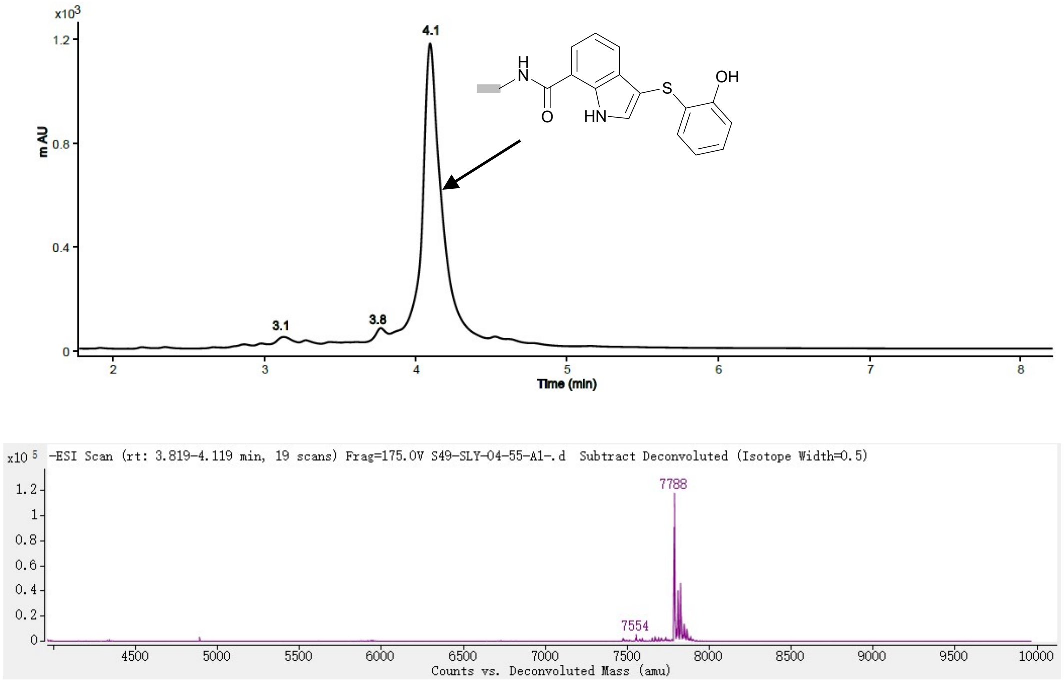


UPLC chromatograph and deconvoluted MS of **3e**.

**Conversion: 86%**

**Calculated Mass: 7785 Da; Found Mass: 7785 Da**

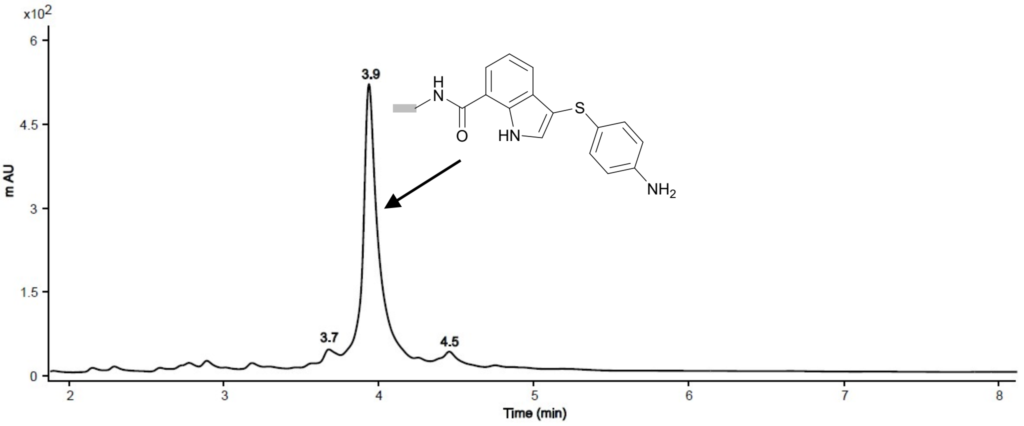


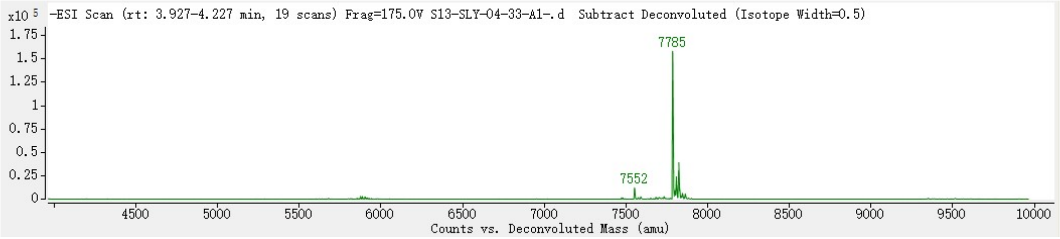


UPLC chromatograph and deconvoluted MS of **3f**.

**Conversion: 79%**

**Calculated Mass: 7848 Da; Found Mass: 7850 Da**

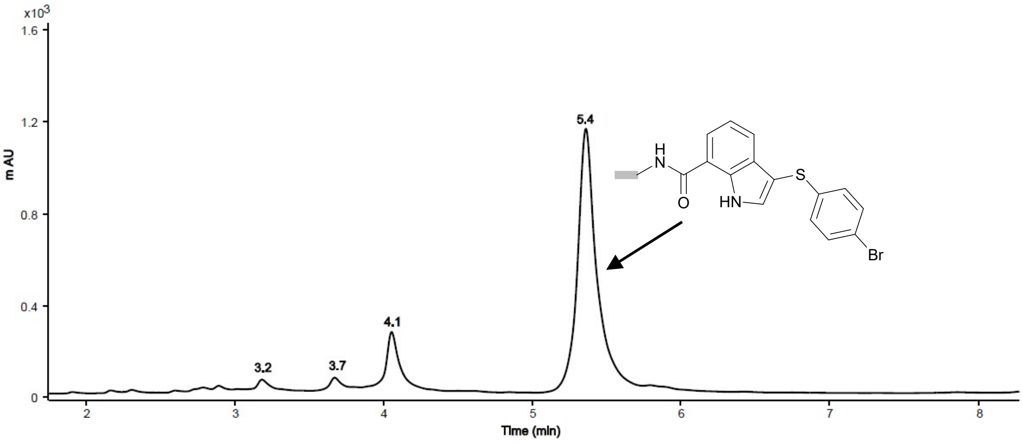


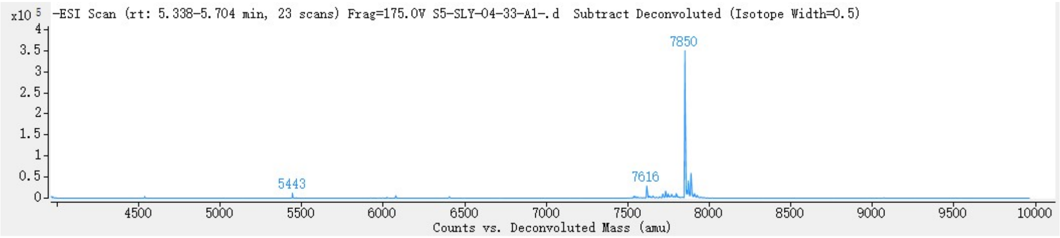


UPLC chromatograph and deconvoluted MS of **3g**.

**Conversion: 12%**

**Calculated Mass: 7815 Da; Found Mass: 7815 Da**

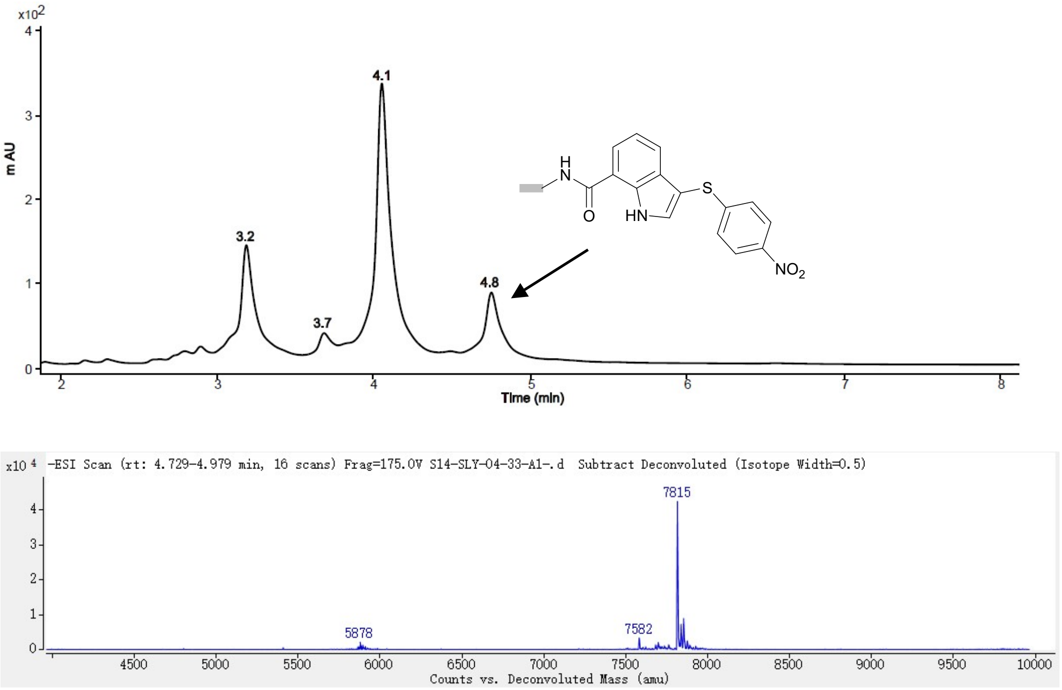


UPLC chromatograph and deconvoluted MS of **3h**.

**Conversion: 70%**

**Calculated Mass: 7820 Da; Found Mass: 7821 Da**

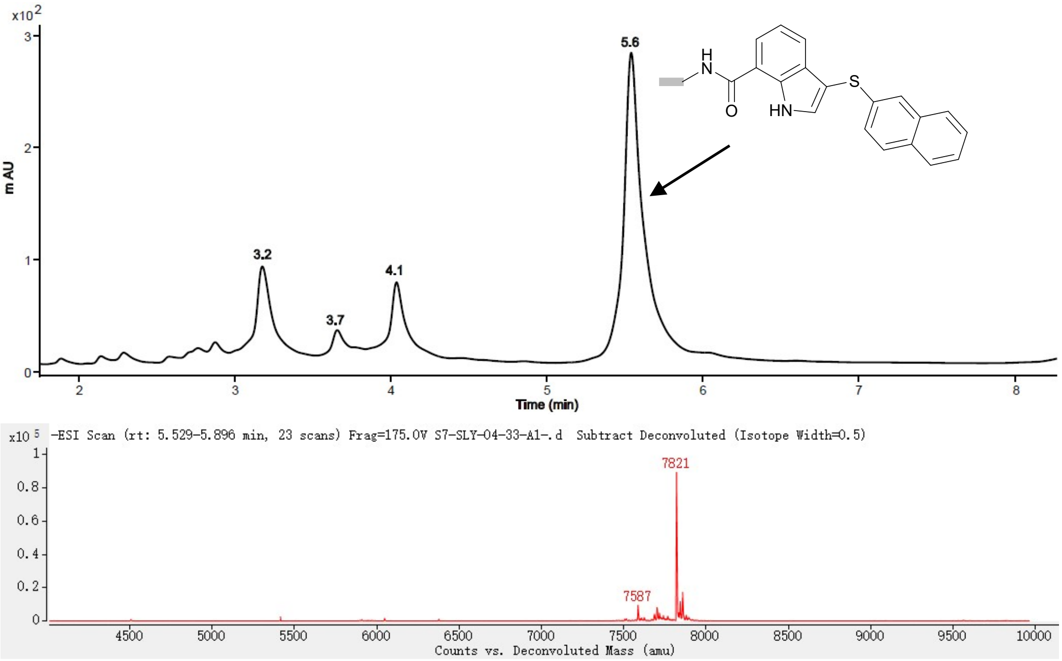


UPLC chromatograph and deconvoluted MS of **3i**.

**Conversion: 76%**

**Calculated Mass: 7774 Da; Found Mass: 7775 Da**

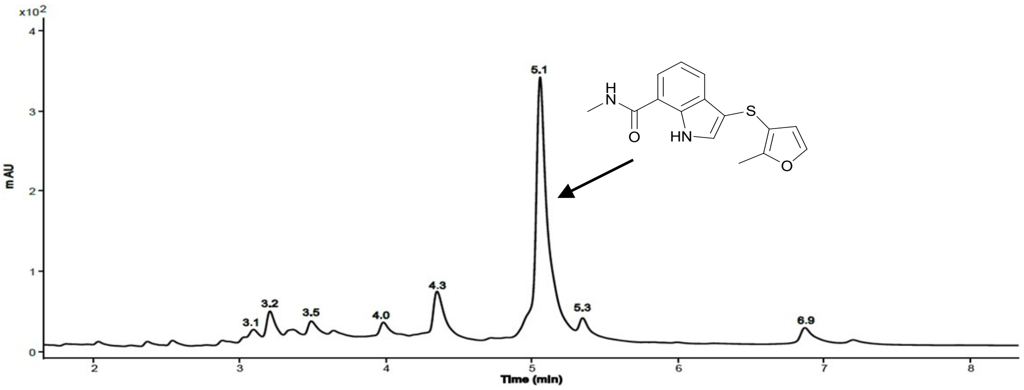


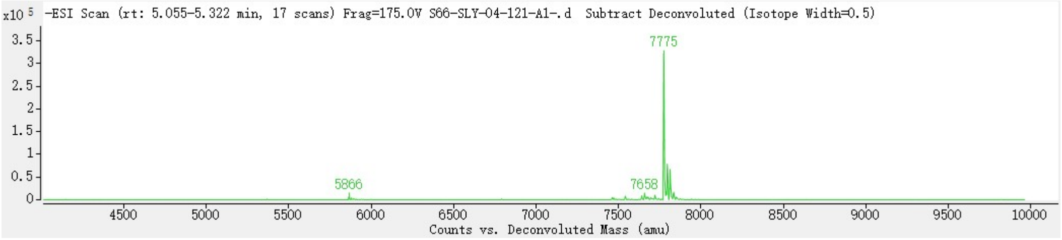


UPLC chromatograph and deconvoluted MS of **3j**.

**Conversion: >95%**

**Calculated Mass: 7792 Da; Found Mass: 7793 Da**

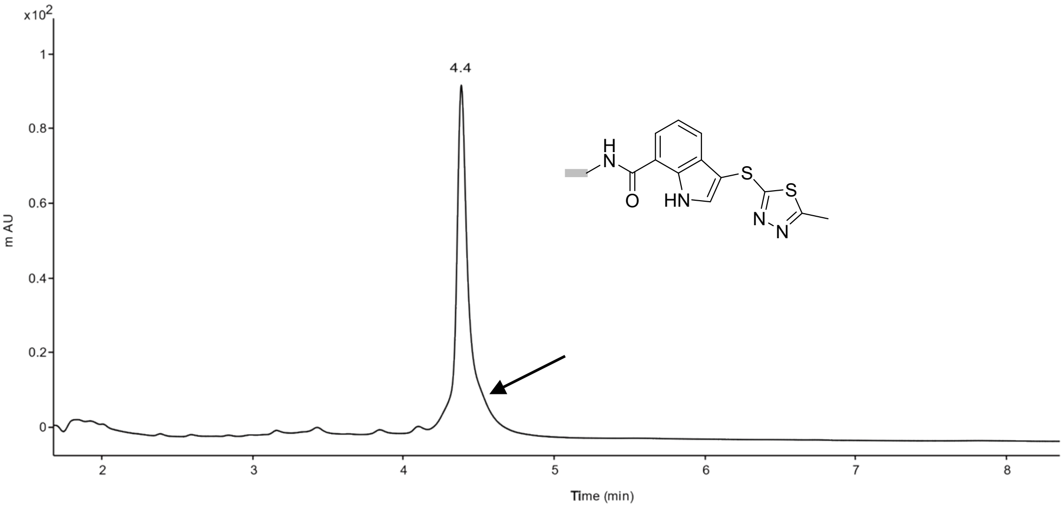


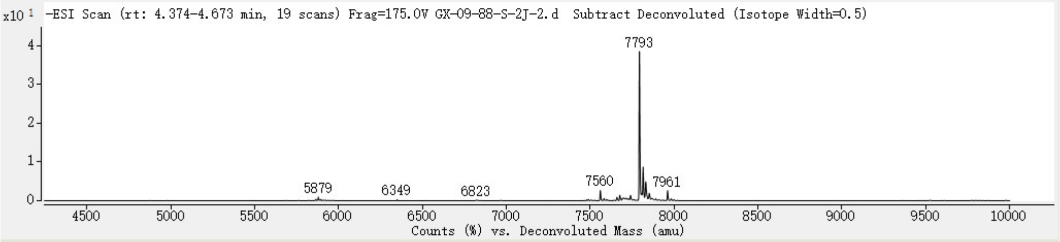


UPLC chromatograph and deconvoluted MS of **3k**.

**Conversion: >95%**

**Calculated Mass: 7776 Da; Found Mass: 7776 Da**

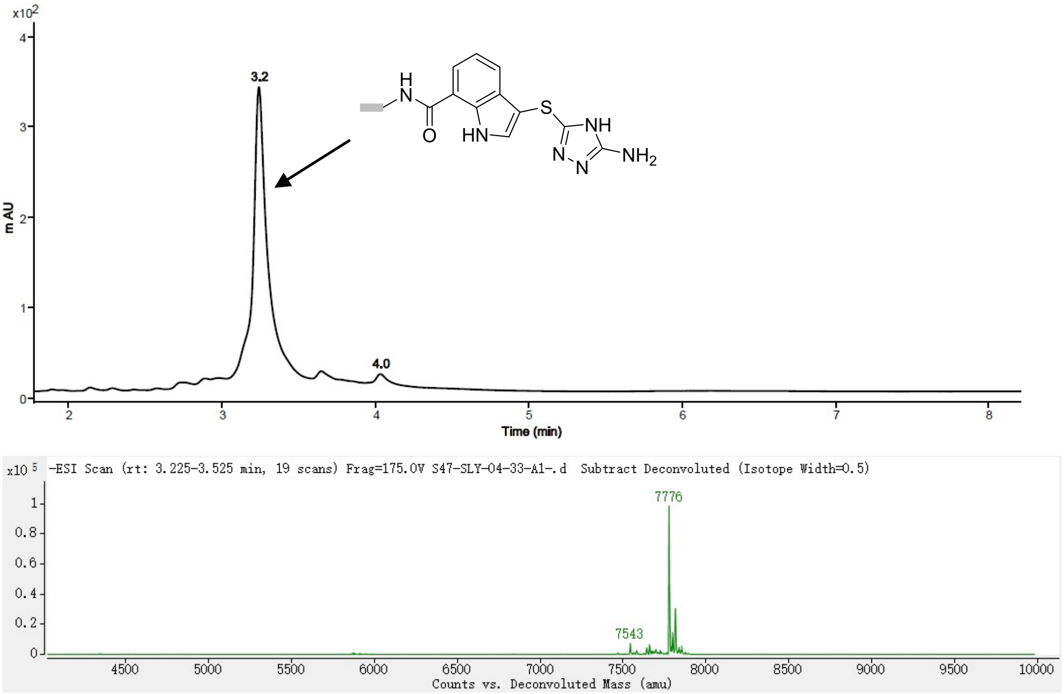


UPLC chromatograph and deconvoluted MS of **3l**.

**Conversion: >95%**

**Calculated Mass: 7811 Da; Found Mass: 7812 Da**

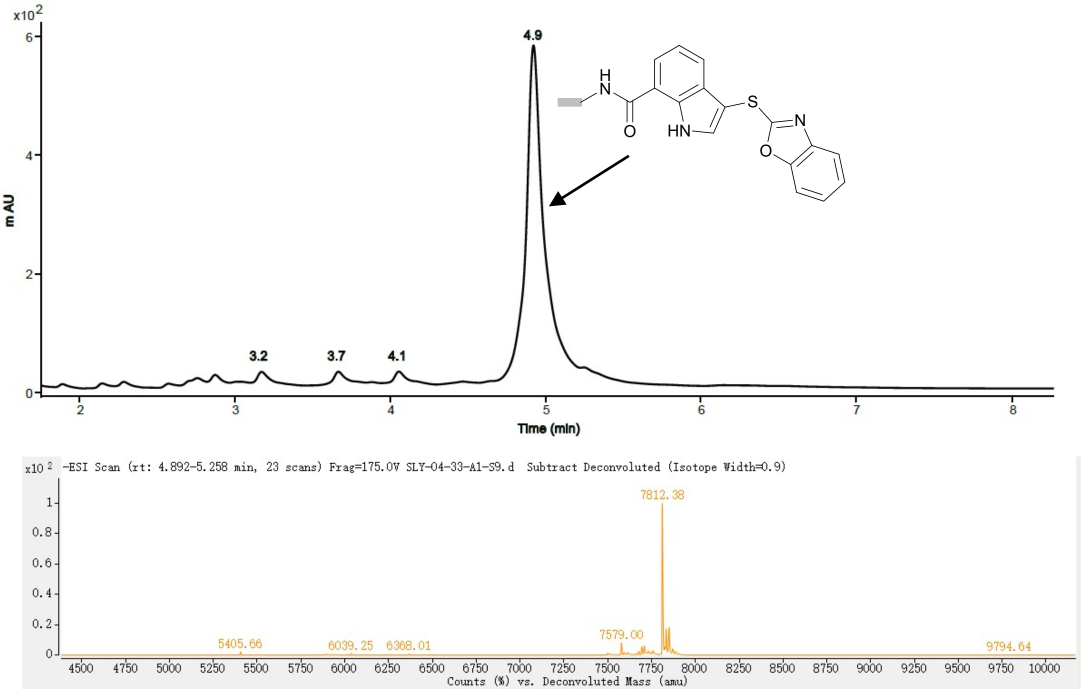


UPLC chromatograph and deconvoluted MS of **3m**.

**Conversion: 92%**

**Calculated Mass: 7856 Da; Found Mass: 7857 Da**

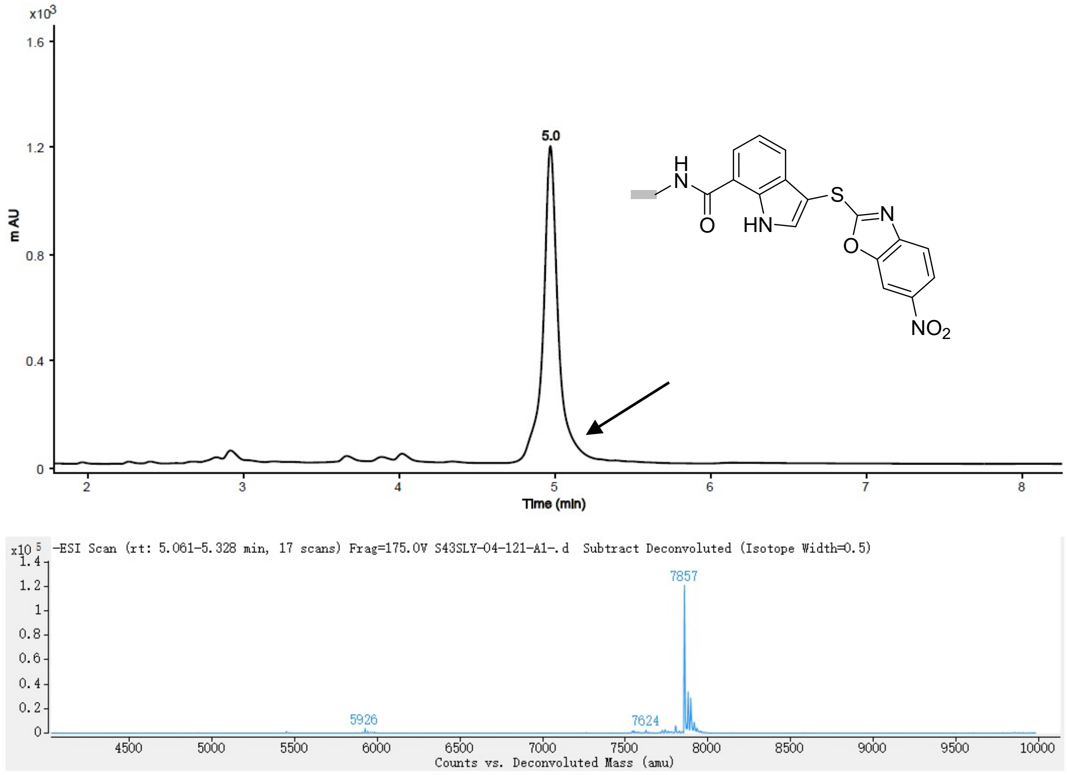


UPLC chromatograph and deconvoluted MS of**3n**.

**Conversion: 86%**

**Calculated Mass: 7771 Da; Found Mass: 7771 Da**

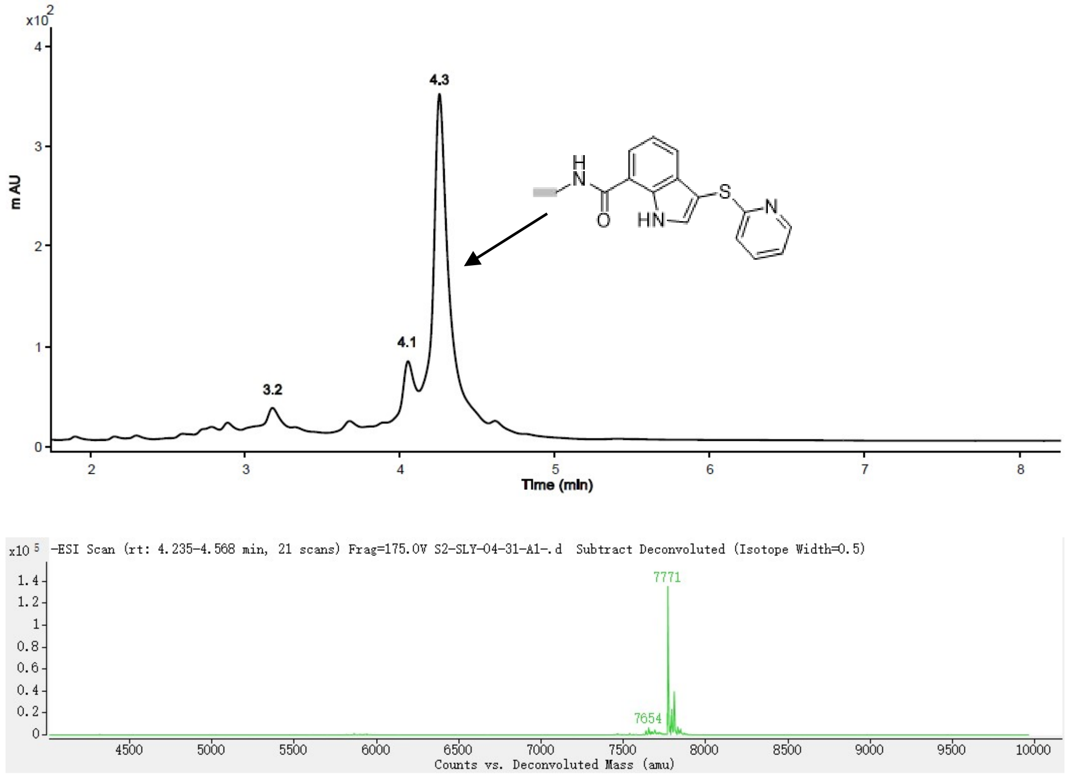


UPLC chromatograph and deconvoluted MS of **3o**.

**Conversion: 93%**

**Calculated Mass: 7777 Da; Found Mass: 7778 Da**

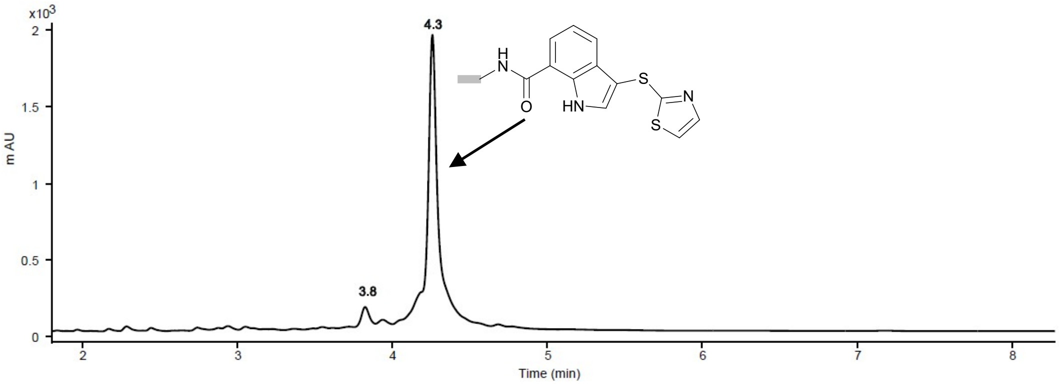


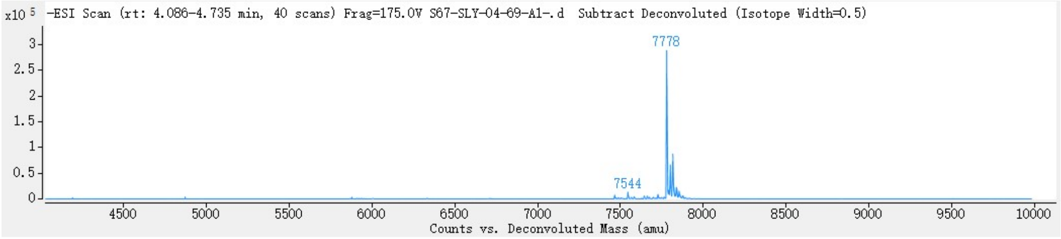


UPLC chromatograph and deconvoluted MS of **3p**.
**Conversion: 87%**
**Calculated Mass: 7760 Da; Found Mass: 7760 Da**

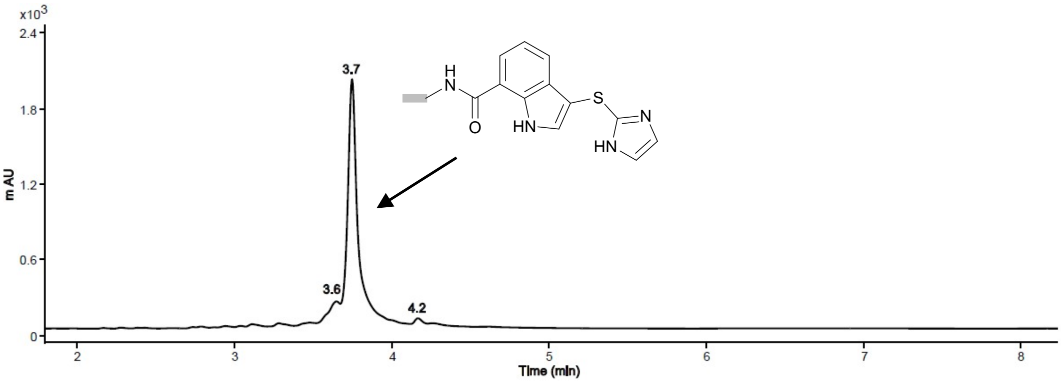


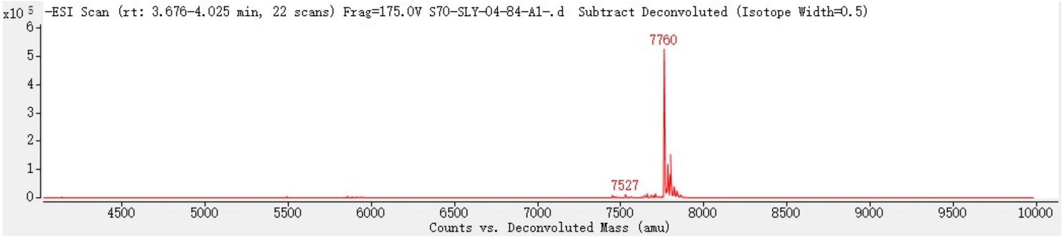


UPLC chromatograph and deconvoluted MS of **3q**.
**Conversion: >95%**

**Calculated Mass: 7841 Da; Found Mass: 7841 Da**

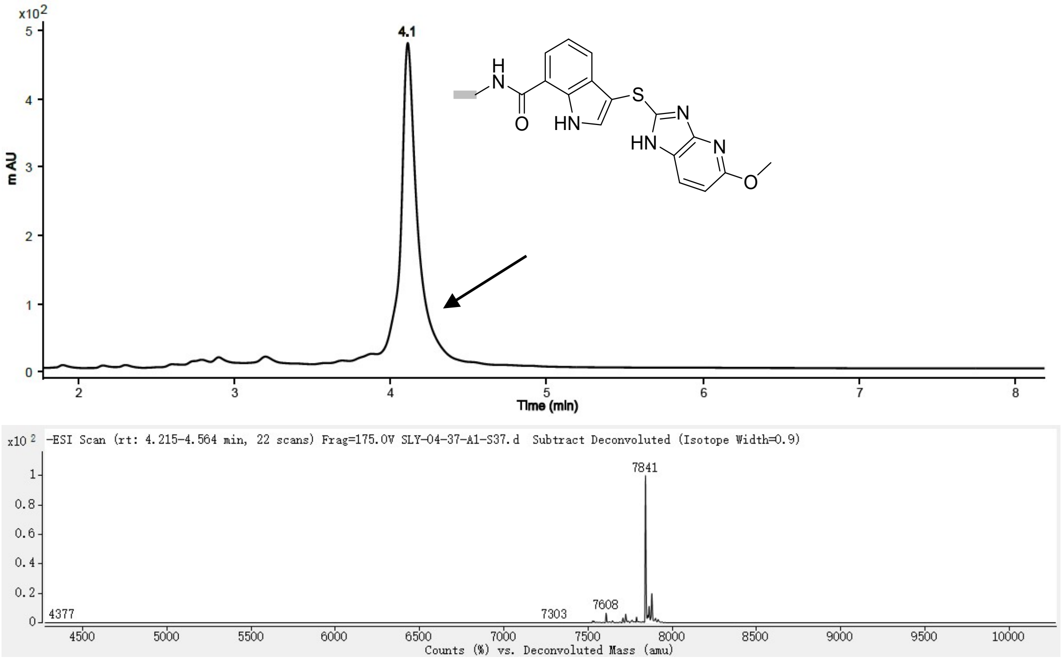

UPLC chromatograph and deconvoluted MS of **3r**.

**Conversion: >95%**

**Calculated Mass: 7810 Da; Found Mass: 7810 Da**

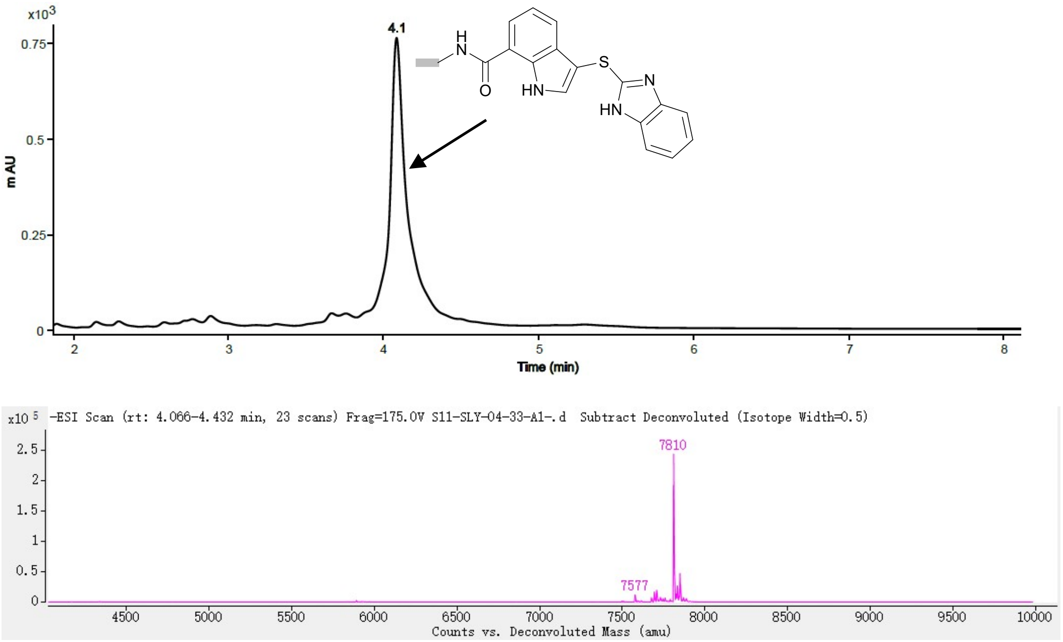

UPLC chromatograph and deconvoluted MS of **3s**.

**Conversion: 92%**

**Calculated Mass: 7825 Da; Found Mass: 7825 Da**

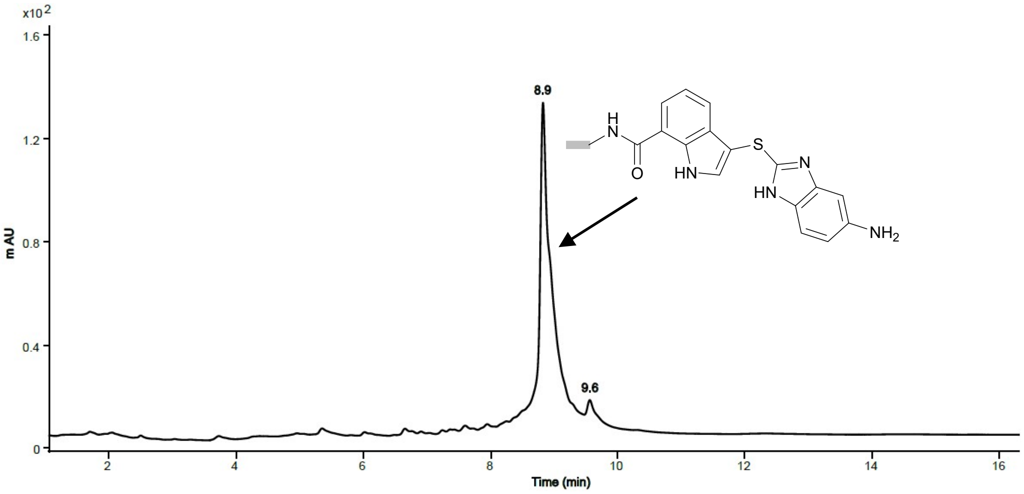


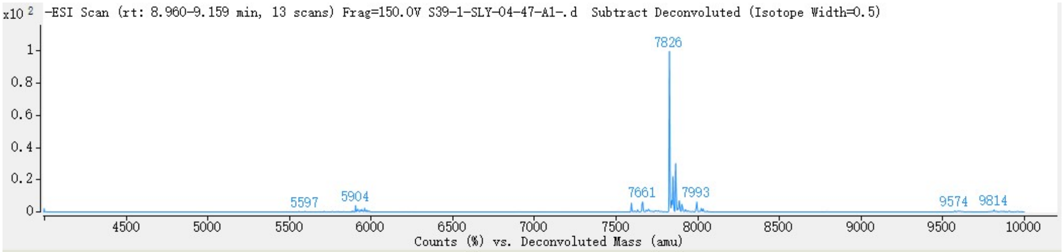


UPLC chromatograph and deconvoluted MS of **3t**.

**Conversion: 93%**

**Calculated Mass: 7876 Da; Found Mass: 7877 Da**

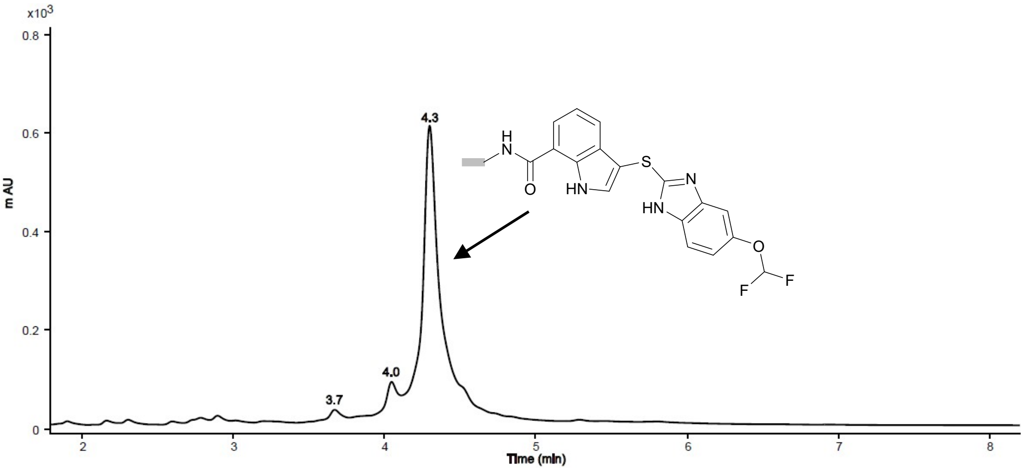


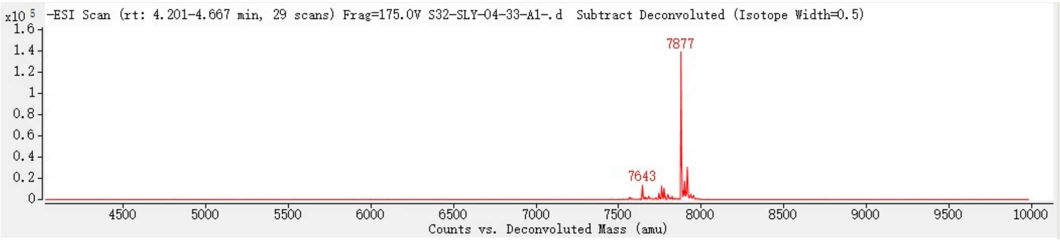


UPLC chromatograph and deconvoluted MS of **3u**.

**Conversion: >95%**

**Calculated Mass: 7854 Da; Found Mass: 7854 Da**

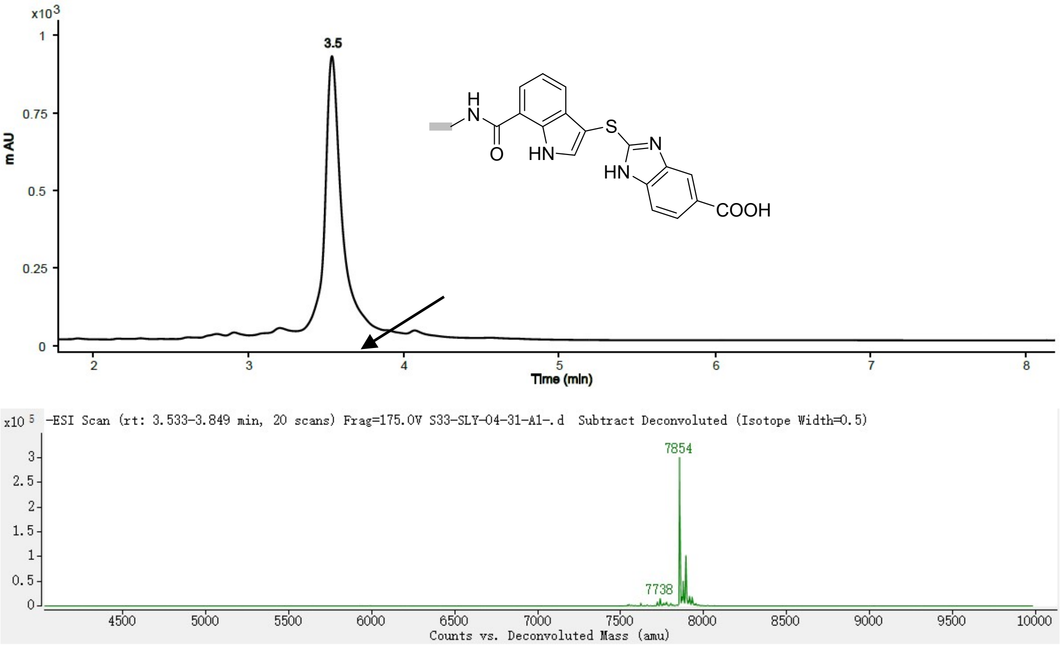


UPLC chromatograph and deconvoluted MS of **3v**.

**Conversion: 92%**

**Calculated Mass: 7855 Da; Found Mass: 7855 Da**

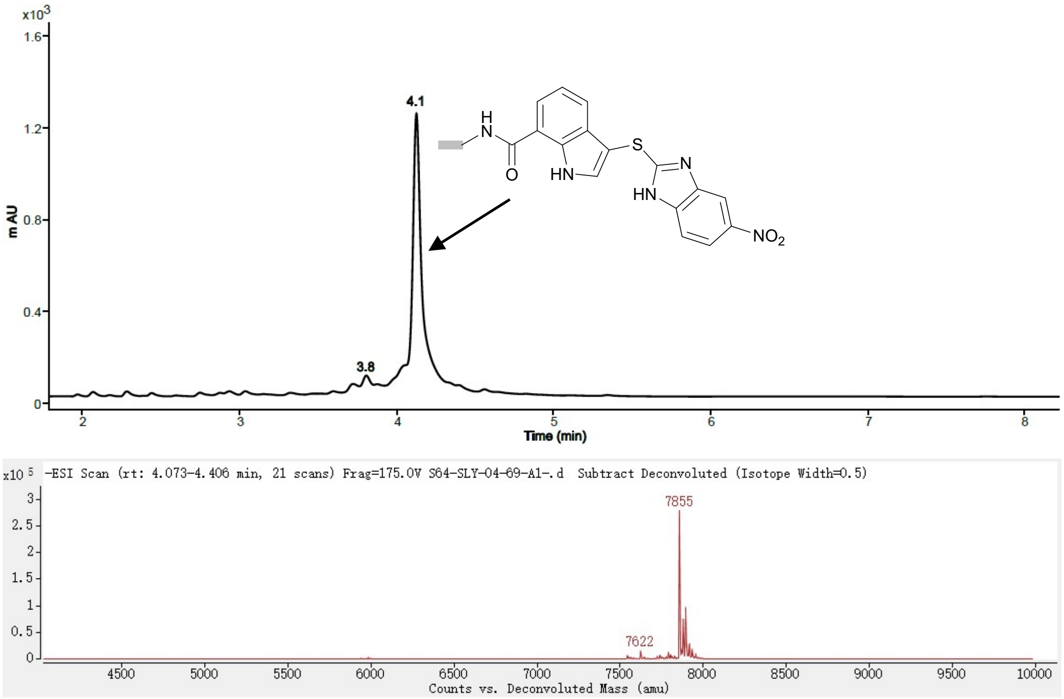


UPLC chromatograph and deconvoluted MS of **3w**.

**Conversion: 93%**

**Calculated Mass: 7845 Da; Found Mass: 7845 Da**

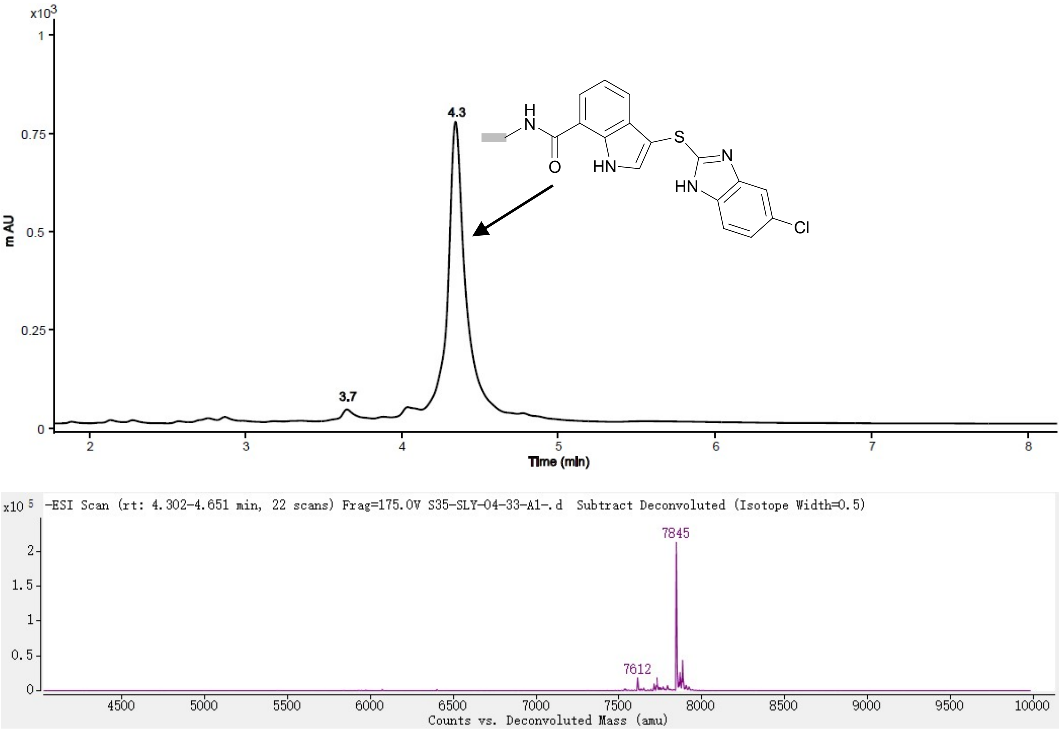


UPLC chromatograph and deconvoluted MS of **3x**.

**Conversion: 11%**

**Calculated Mass: 7784 Da; Found Mass: 7785 Da**

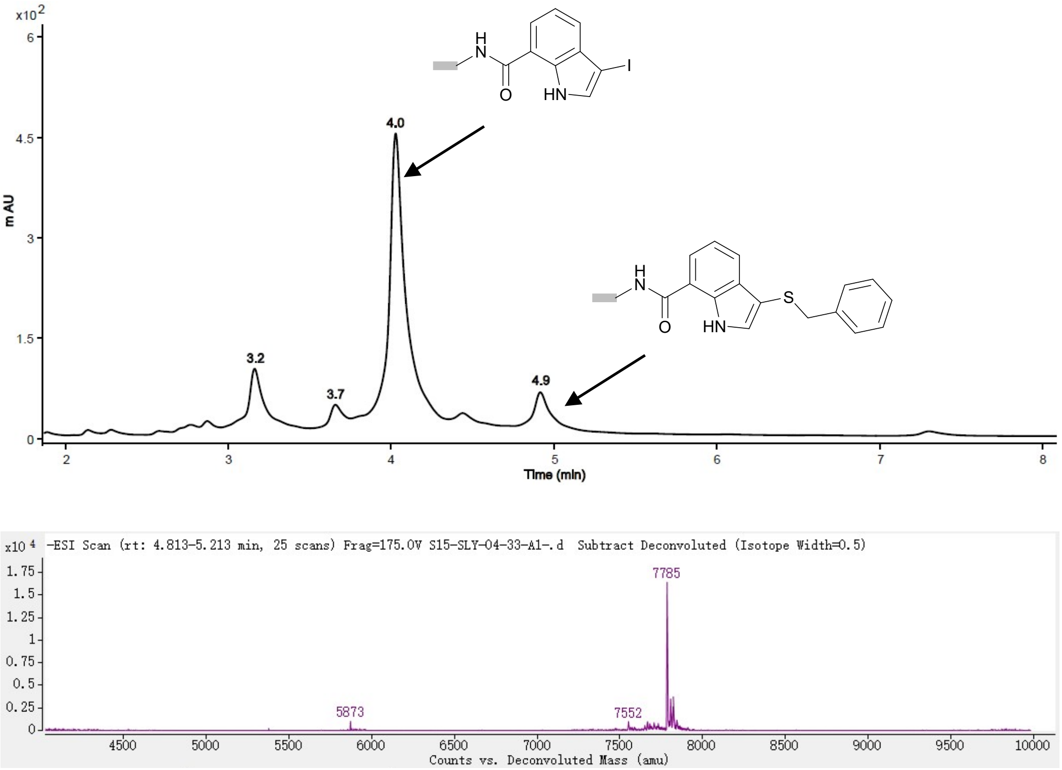


UPLC chromatograph and deconvoluted MS of **3y**.

**Conversion: 65%**

**Calculated Mass: 7800 Da; Found Mass: 7802 Da**

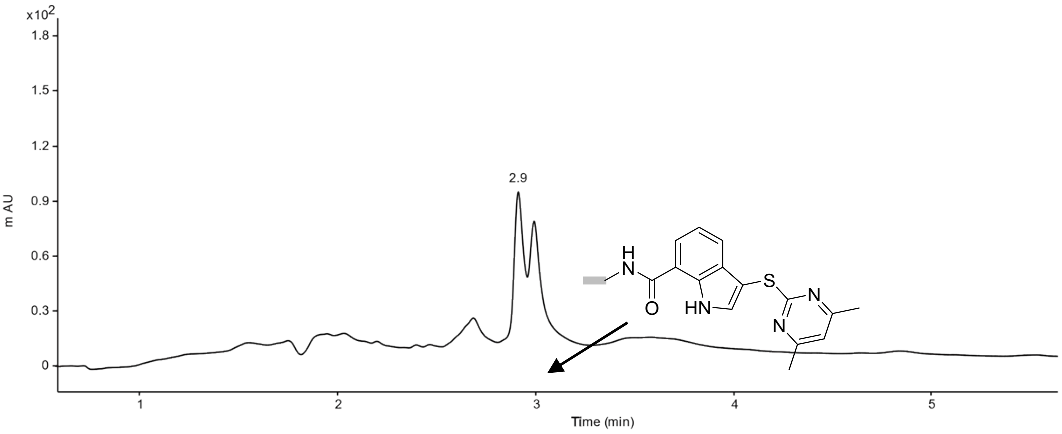


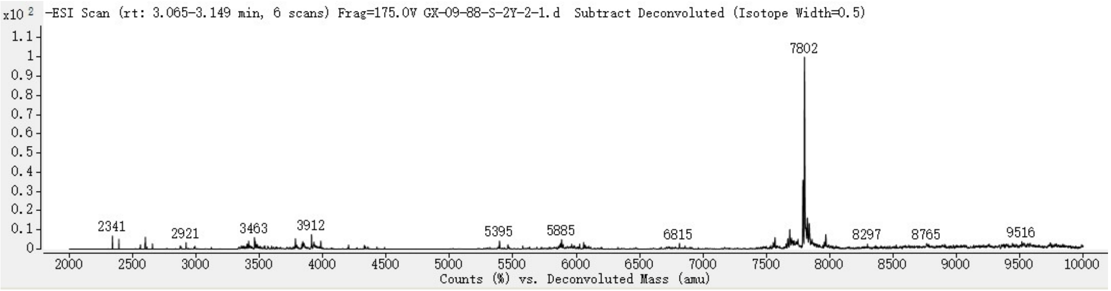


UPLC chromatograph and deconvoluted MS of **3z**.

**Conversion: >95%**

**Calculated Mass: 7775 Da; Found Mass: 7775 Da**

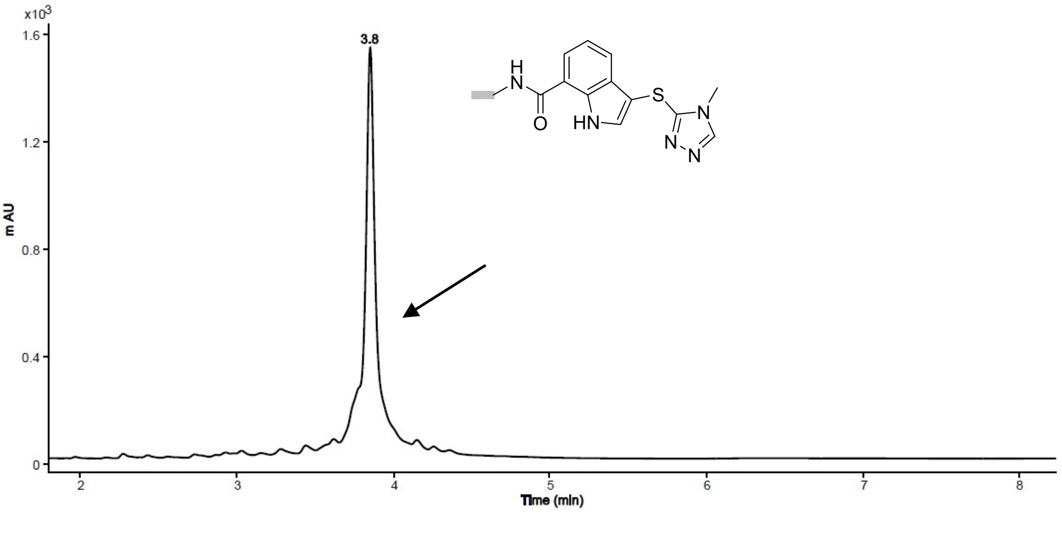


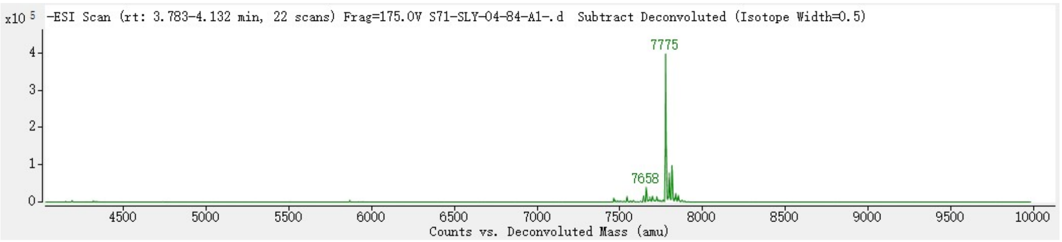


UPLC chromatograph and deconvoluted MS of **3aa**.

**Conversion: >95%**

**Calculated Mass: 7820 Da; Found Mass: 7821 Da**

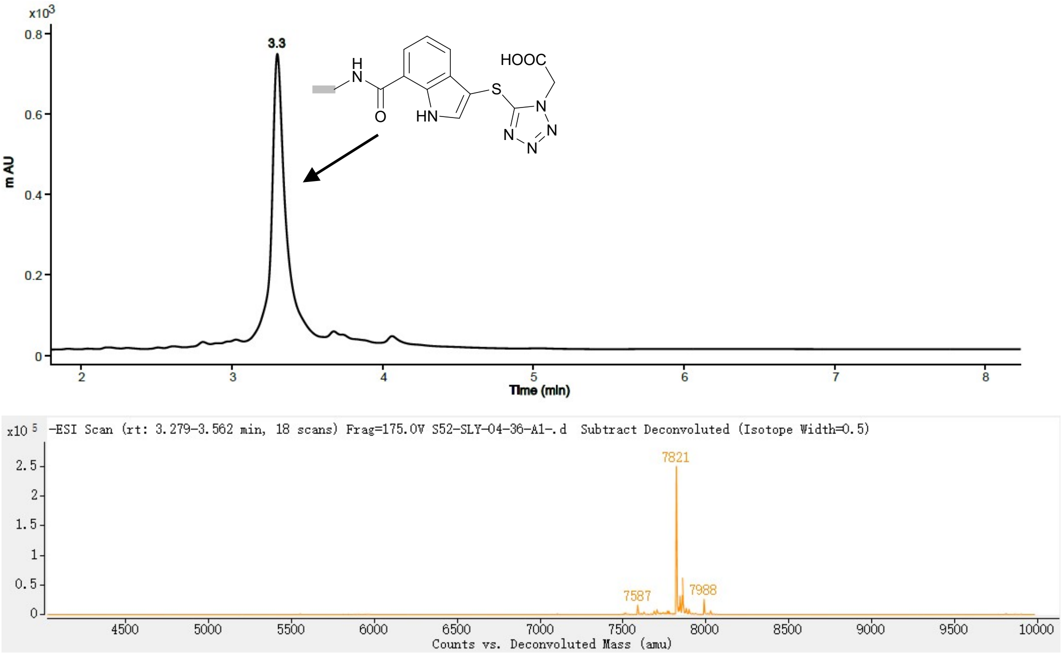


UPLC chromatograph and deconvoluted MS of **3ab**.

**Conversion: 82%**

**Calculated Mass: 7812 Da; Found Mass: 7813 Da**

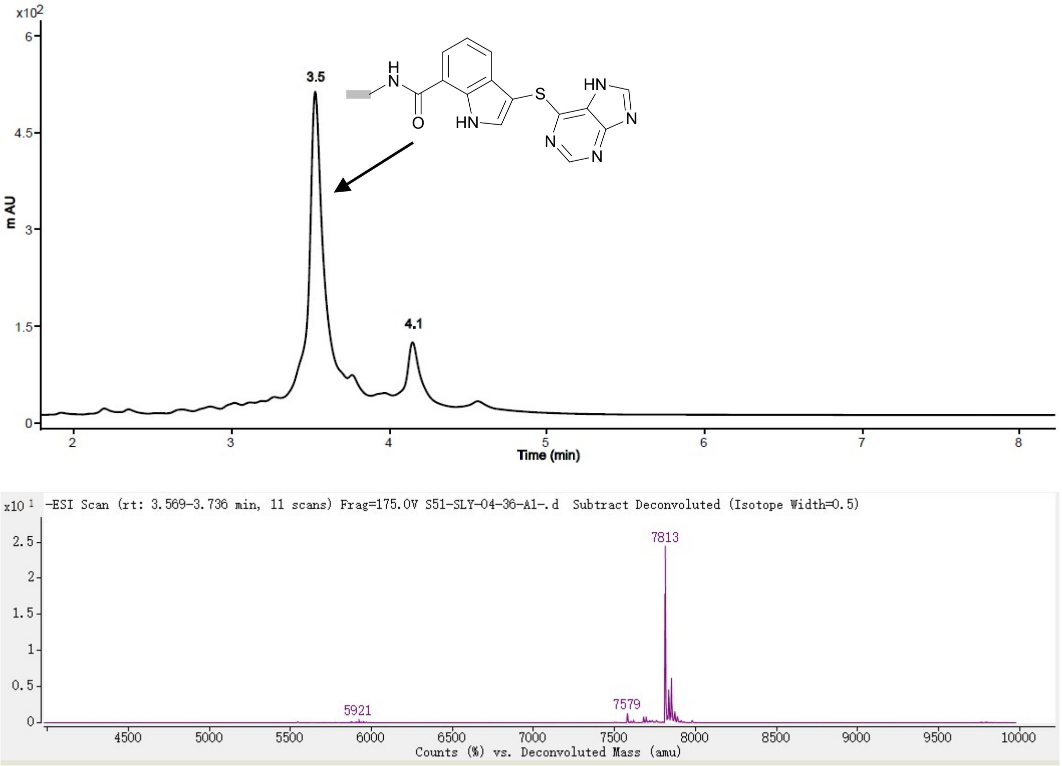


UPLC chromatograph and deconvoluted MS of **3ac**.

**Conversion: 88%**

**Calculated Mass: 7812 Da; Found Mass: 7813 Da**

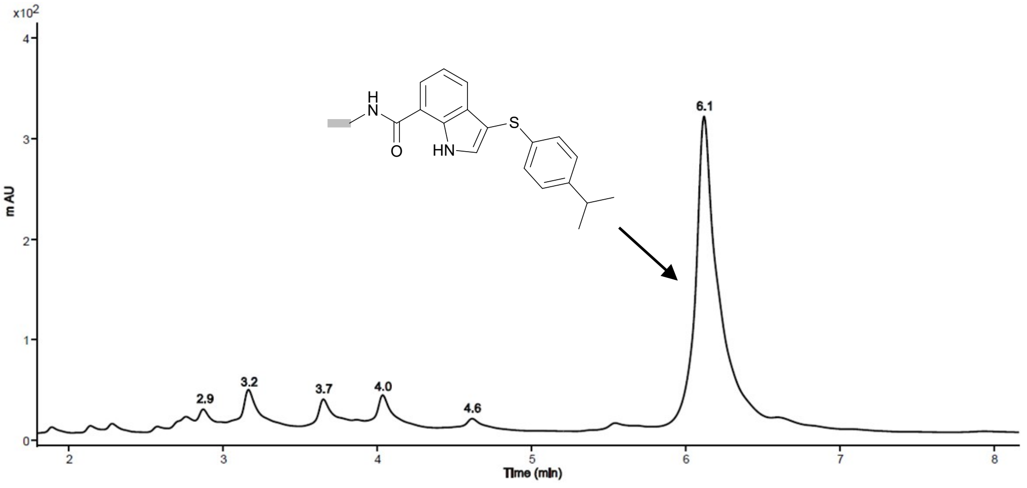


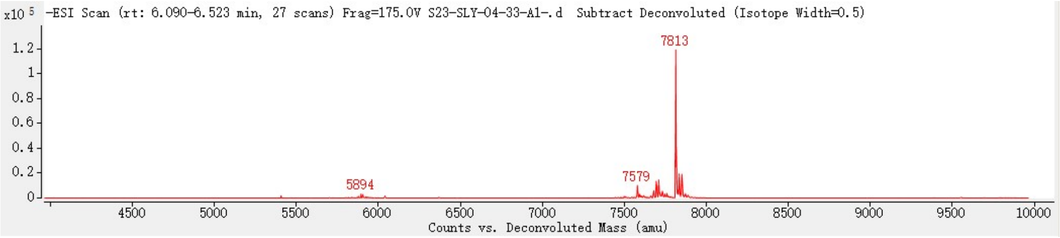


UPLC chromatograph and deconvoluted MS of **3ad**.

**Conversion: 36%**

**Calculated Mass: 7906 Da; Found Mass: 7907 Da**

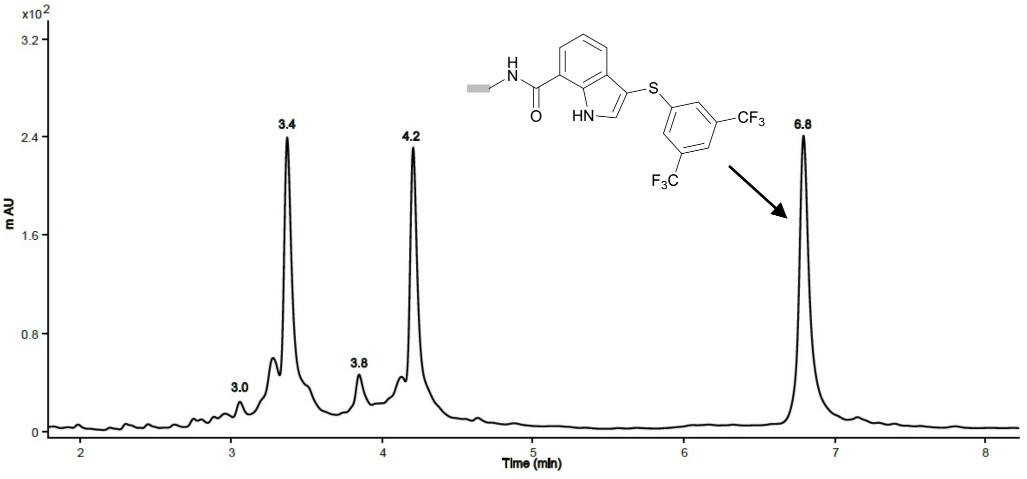


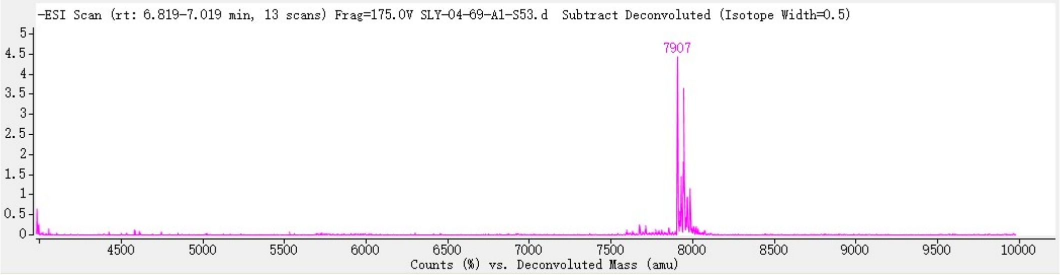


UPLC chromatograph and deconvoluted MS of **3ae**.

**Conversion: 93%**

**Calculated Mass: 7774 Da; Found Mass: 7774 Da**

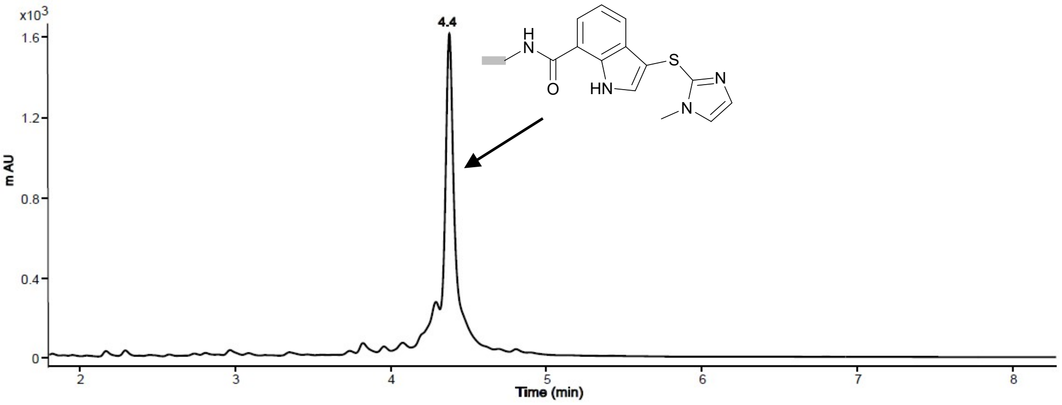


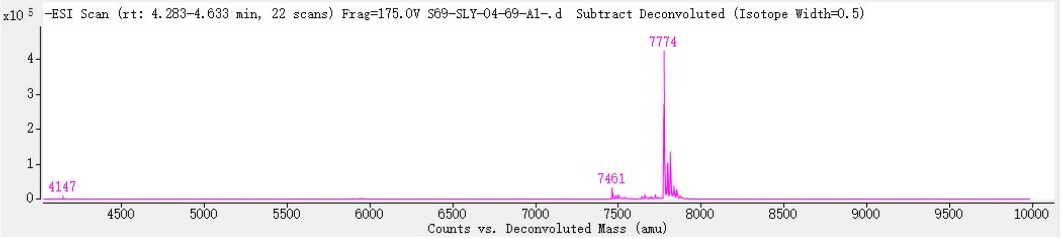


UPLC chromatograph and deconvoluted MS of **3af**.

**Conversion: 82%**

**Calculated Mass: 7854 Da; Found Mass: 7855 Da**

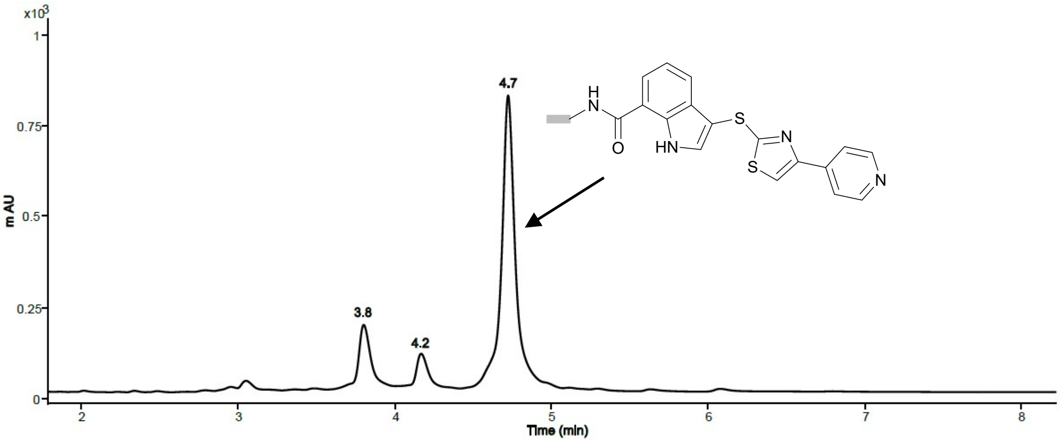


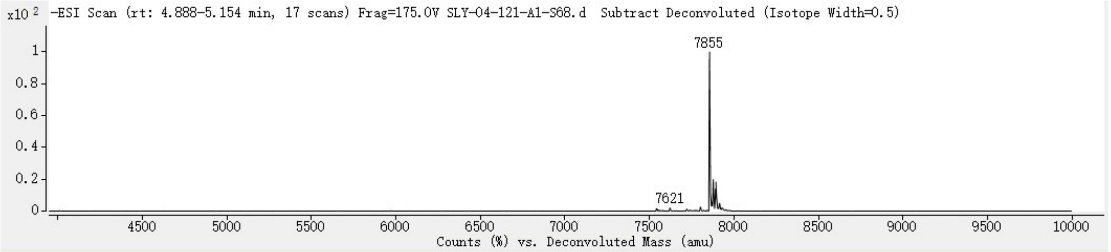


UPLC chromatograph and deconvoluted MS of **3ag**.

**Conversion: 94%**

**Calculated Mass: 7761 Da; Found Mass: 7761 Da**

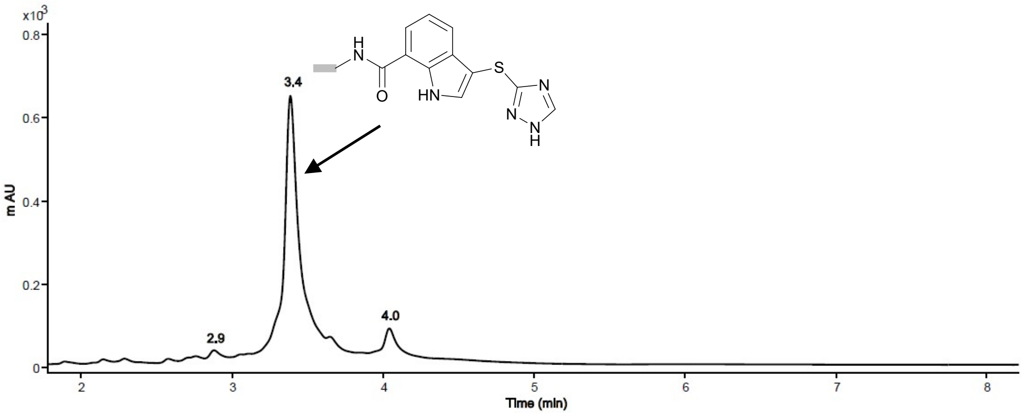


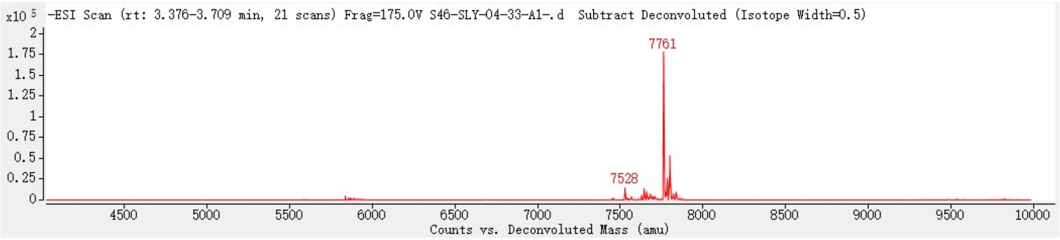


UPLC chromatograph and deconvoluted MS of **3ah**.

**Conversion: 94%**

**Calculated Mass: 7793 Da; Found Mass: 7794 Da**

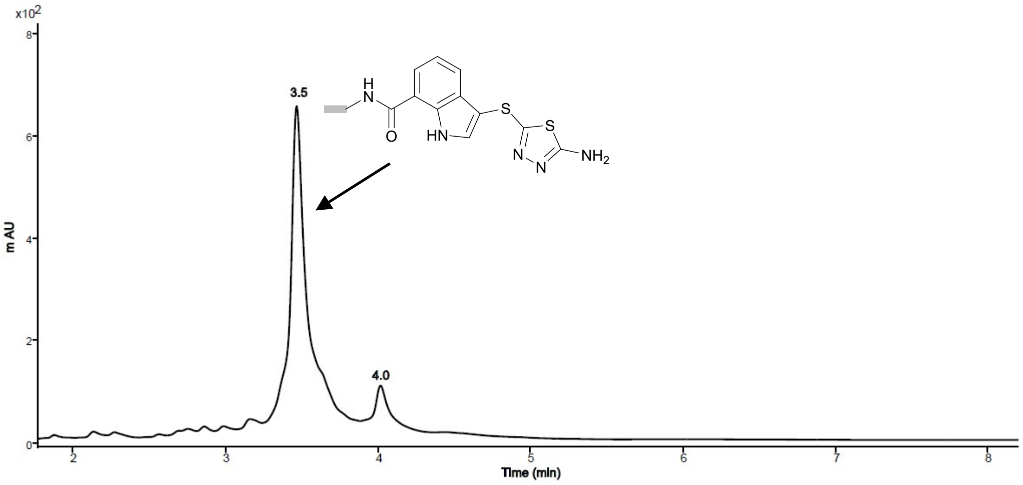


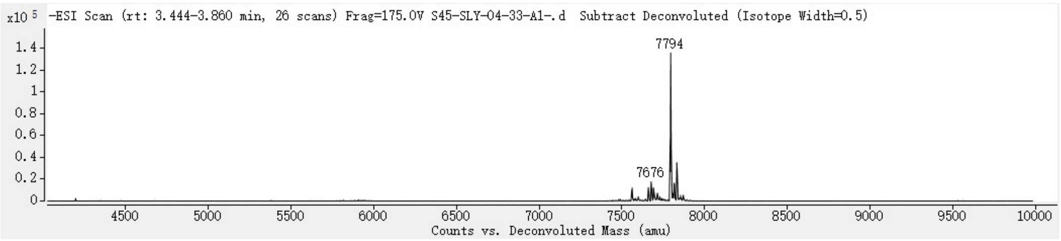


UPLC chromatograph and deconvoluted MS of **3ai**.

**Conversion: 90%**

**Calculated Mass: 7846 Da; Found Mass: 7846 Da**

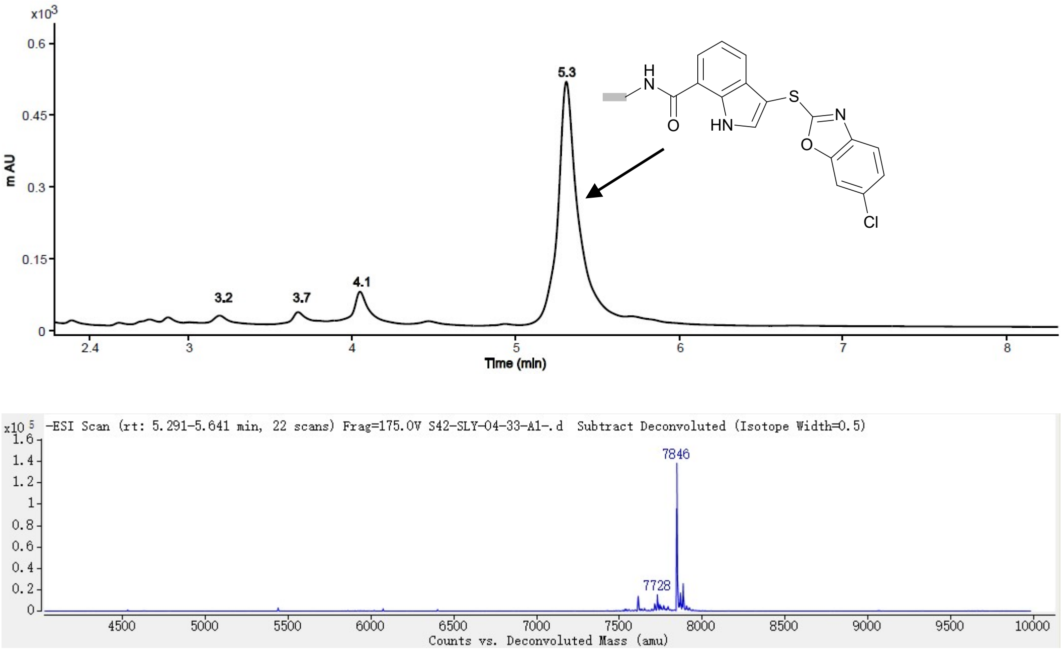


UPLC chromatograph and deconvoluted MS of **3aj**.

**Conversion: >95%**

**Calculated Mass: 7829 Da; Found Mass: 7830 Da**

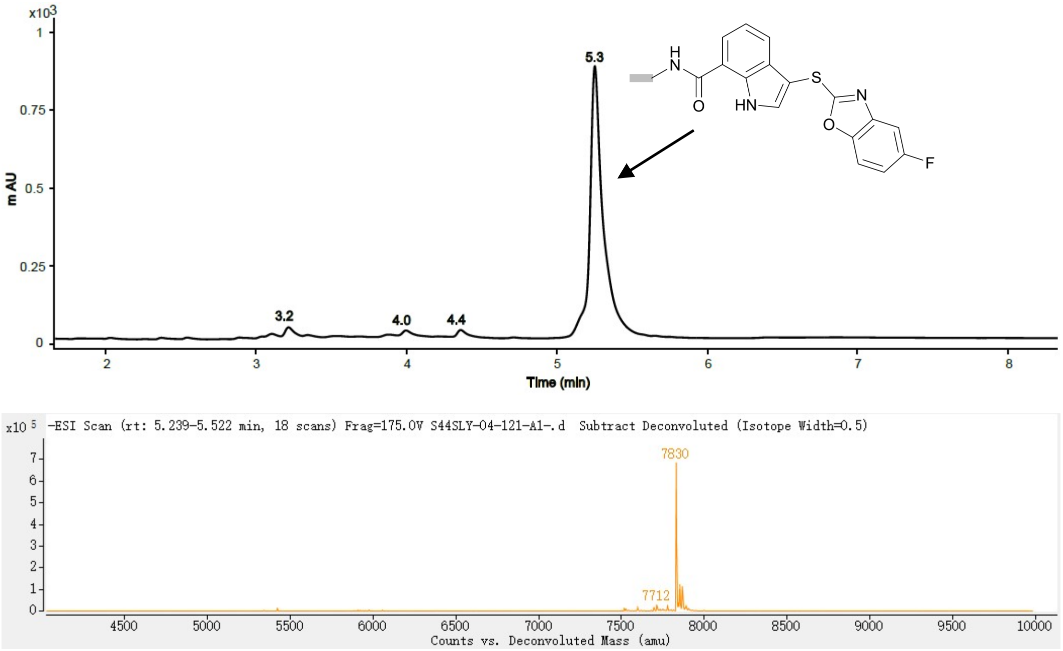


UPLC chromatograph and deconvoluted MS of **3ak**.

**Conversion: 95%**

**Calculated Mass: 7800 Da; Found Mass: 7801 Da**

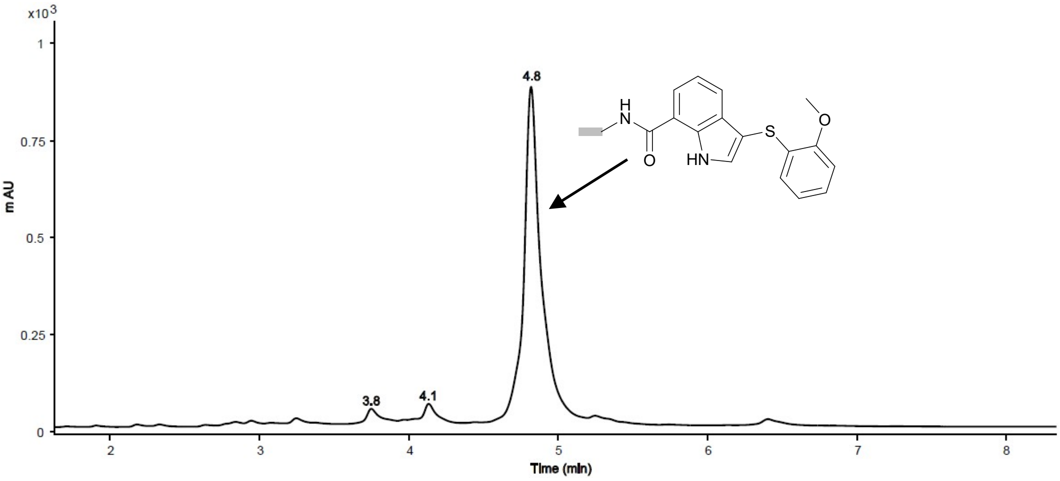


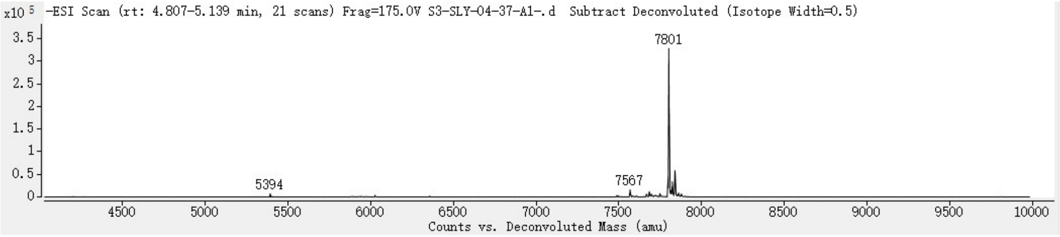


UPLC chromatograph and deconvoluted MS of **3al**.

**Conversion: 94%**

**Calculated Mass: 7828 Da; Found Mass: 7828 Da**

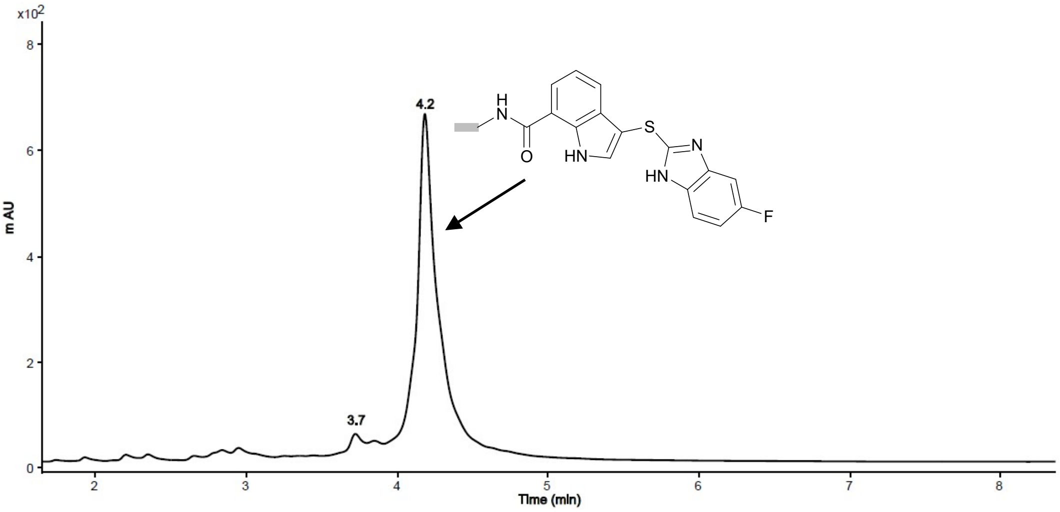


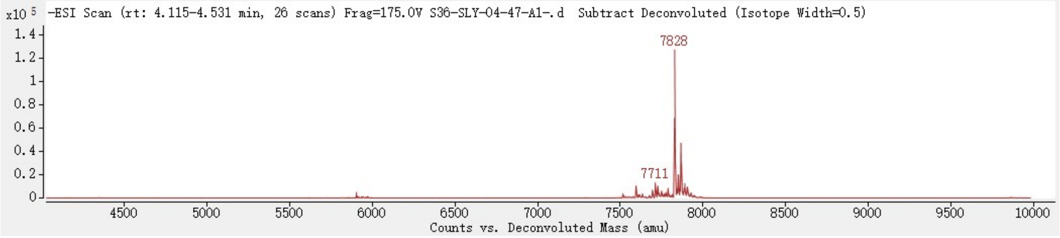


UPLC chromatograph and deconvoluted MS of **3am**.

**Conversion: 94%**

**Calculated Mass: 7786 Da; Found Mass: 7786 Da**

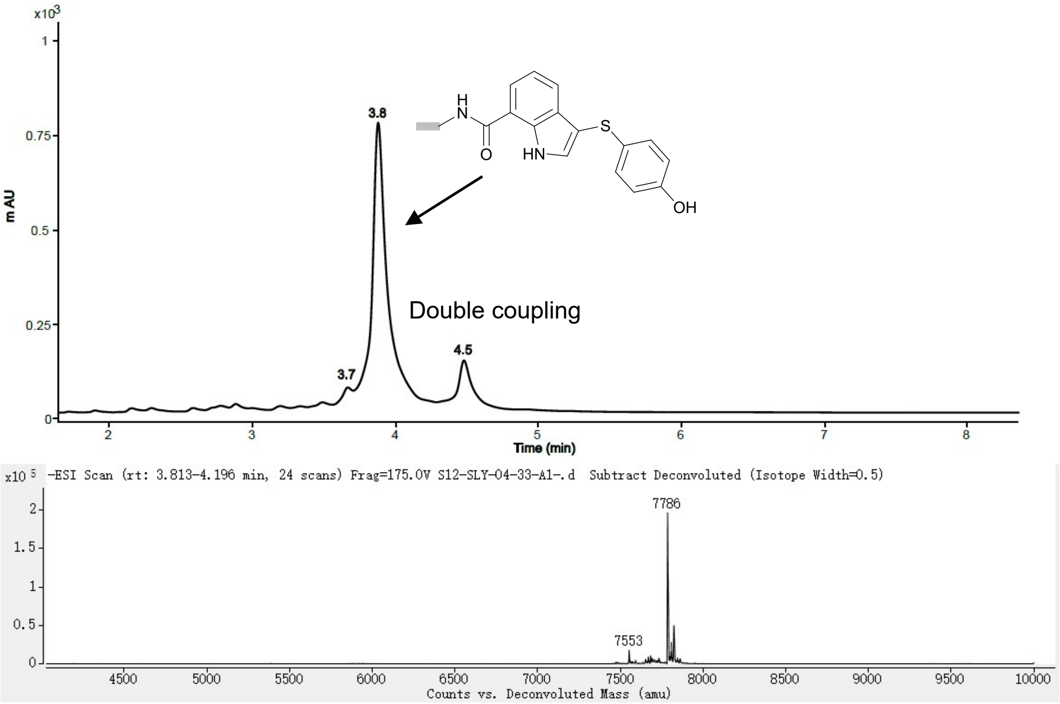


UPLC chromatograph and deconvoluted MS of **3an**.

**Conversion: 97%**

**Calculated Mass: 7788 Da; Found Mass: 7788 Da**

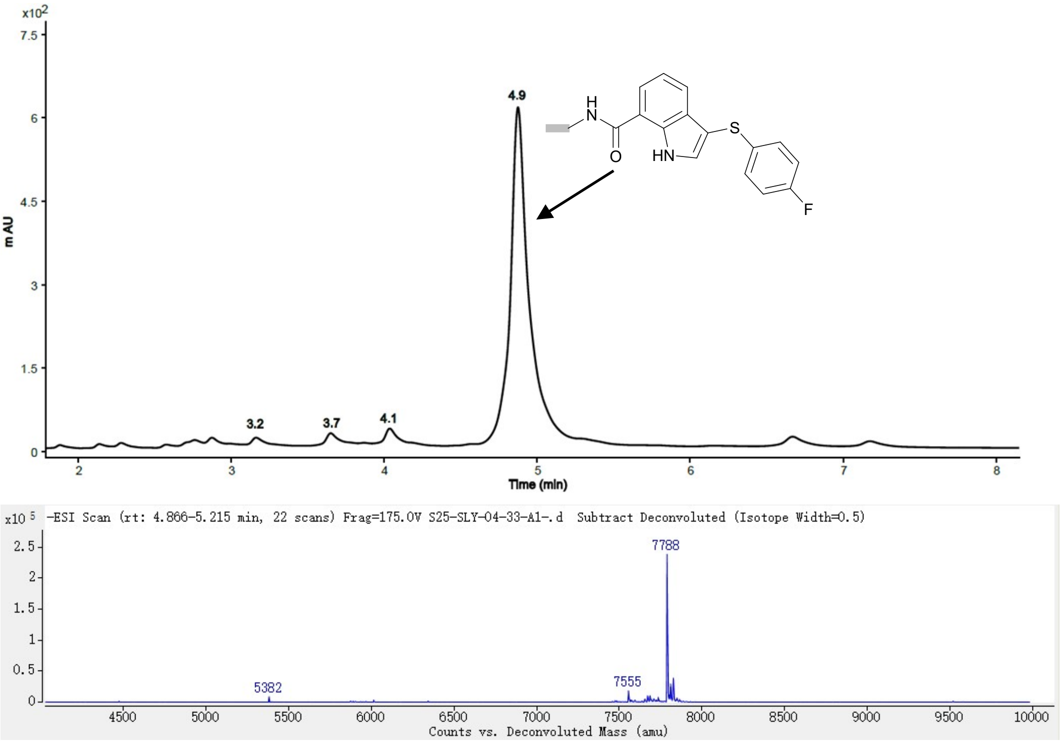


UPLC chromatograph and deconvoluted MS of **3ao**.

**Conversion: 40%**
**Calculated Mass: 7802 Da; Found Mass: 7802 Da**

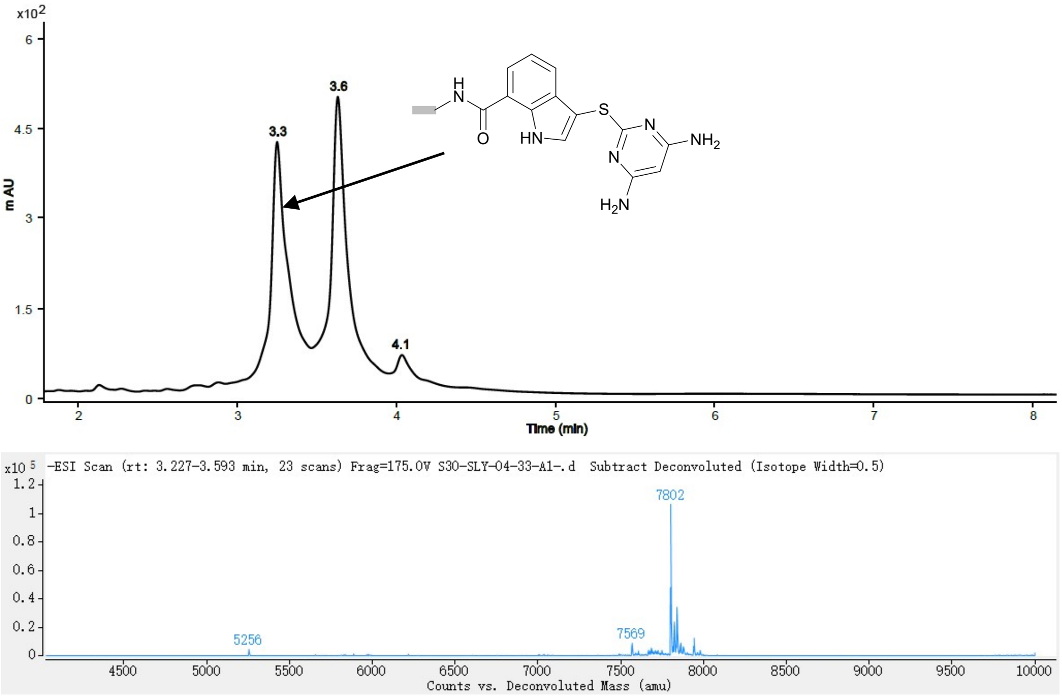


UPLC chromatograph and deconvoluted MS of **3ap**.

**Conversion: 90%**
**Calculated Mass: 7776 Da; Found Mass: 7777 Da**

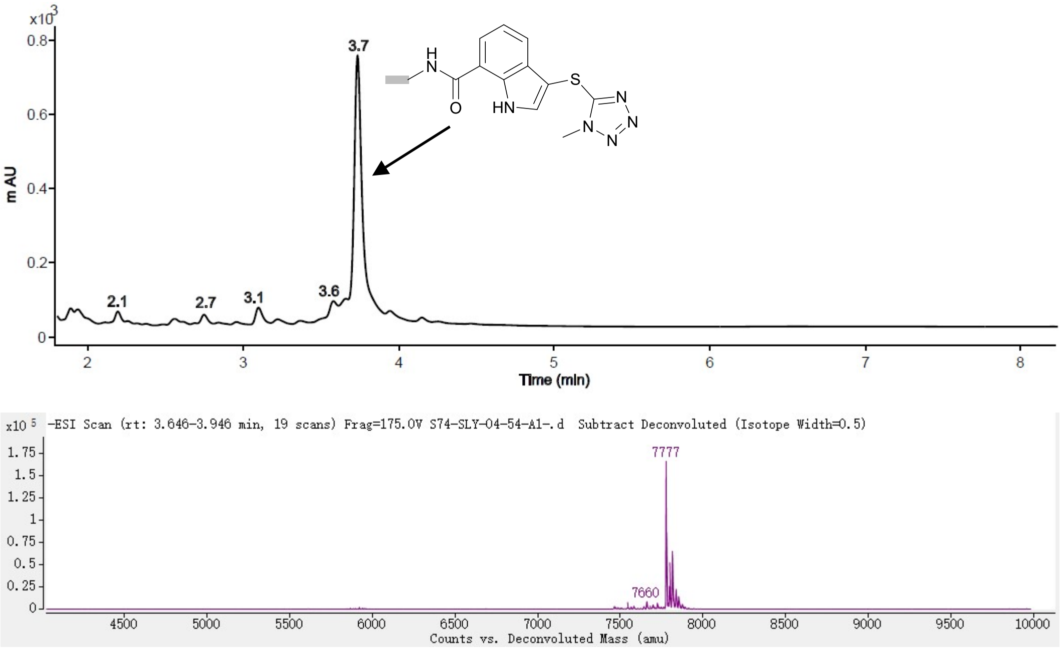


UPLC chromatograph and deconvoluted MS of **3aq**.

**Conversion: 71%**
**Calculated Mass: 7868 Da; Found Mass: 7869 Da**

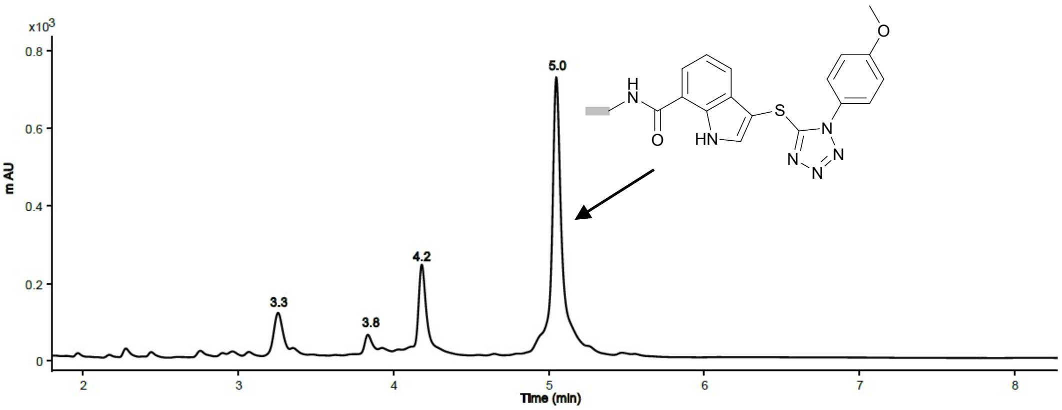


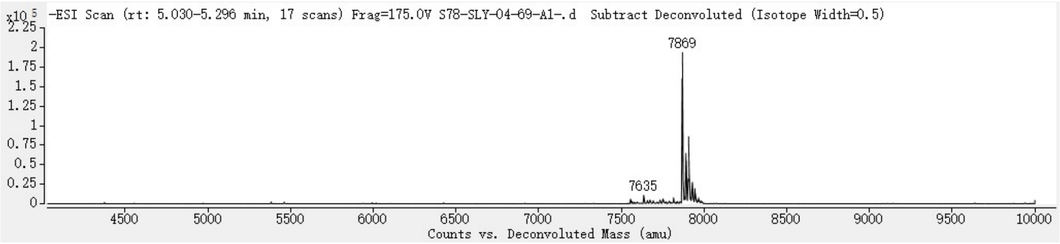


UPLC chromatograph and deconvoluted MS of **3ar**.
**Conversion: >95%**
**Calculated Mass: 7778 Da; Found Mass: 7778 Da**

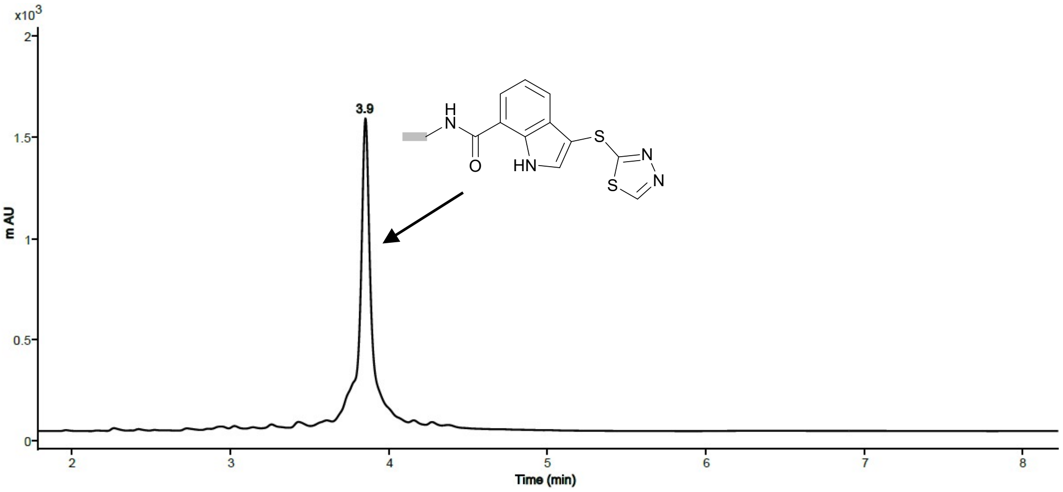


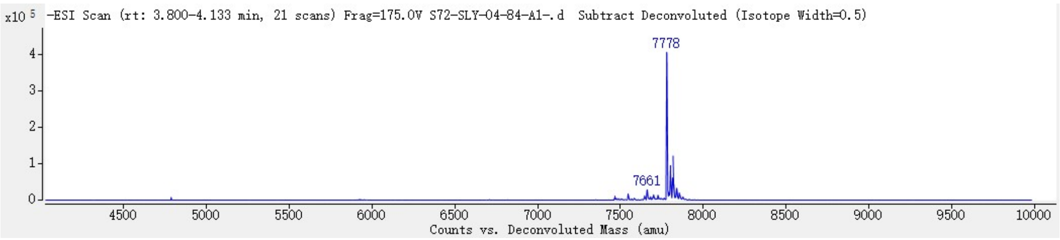


UPLC chromatograph and deconvoluted MS of **3as**.

**Conversion: 67%**

**Calculated Mass: 7826 Da; Found Mass: 7826 Da**

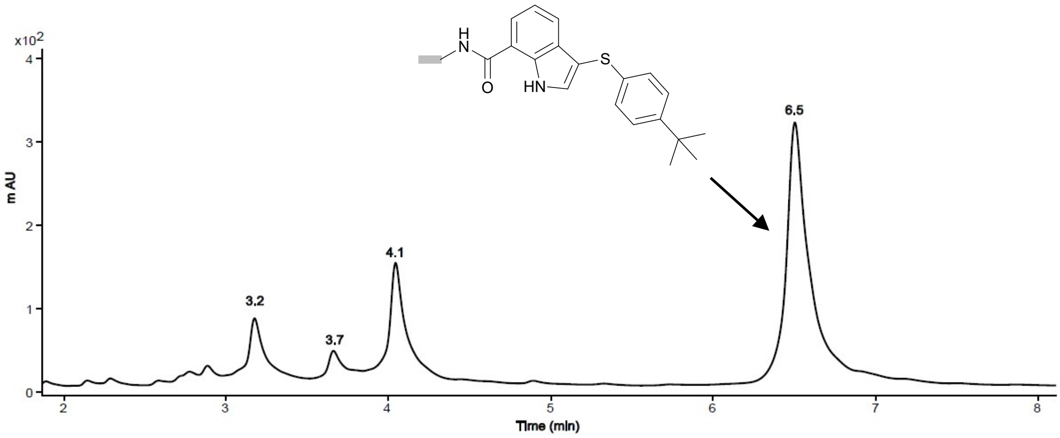


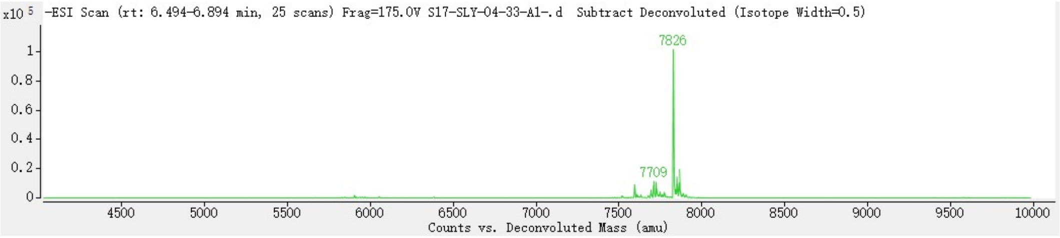


UPLC chromatograph and deconvoluted MS of **3at**.

**Conversion: >95%**

**Calculated Mass: 7841 Da; Found Mass: 7841 Da**

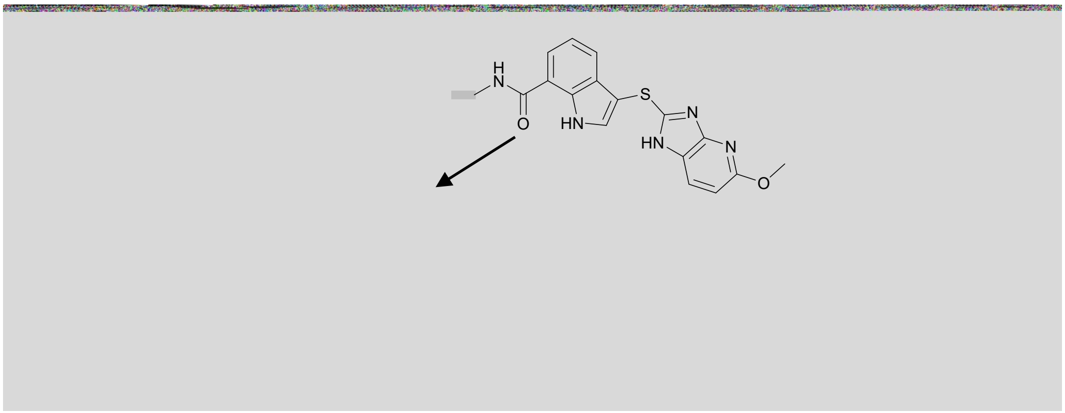

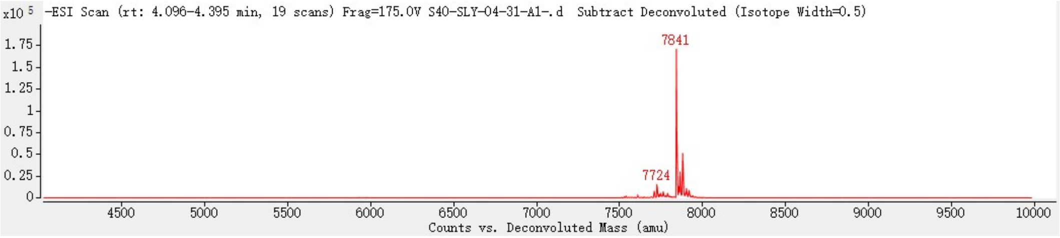

UPLC chromatograph and deconvoluted MS of **3au**.

**Conversion: 22%**

**Calculated Mass: 7827 Da; Found Mass: 7827 Da**

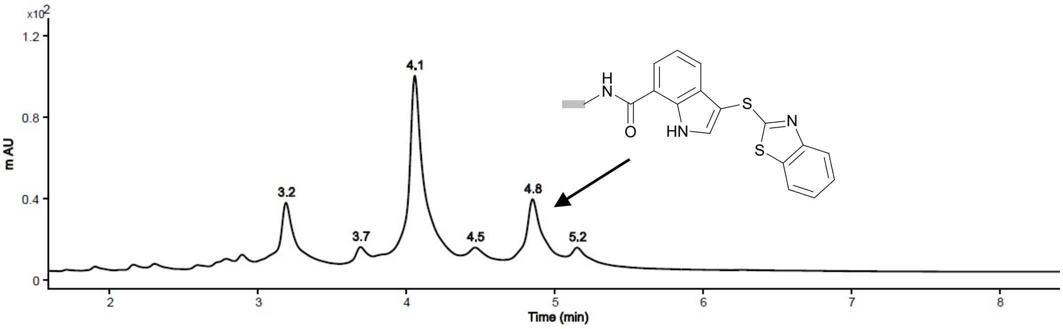


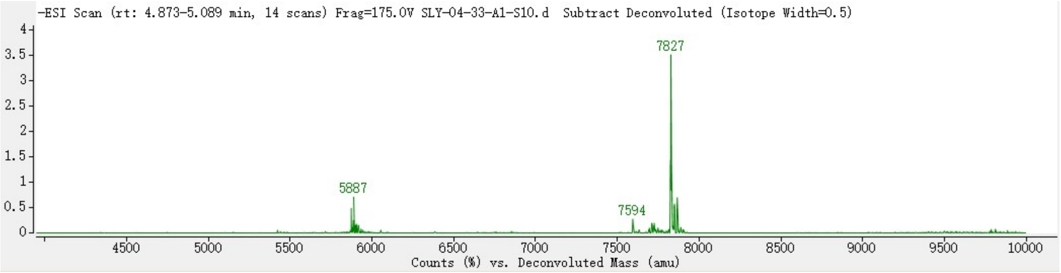


UPLC chromatograph and deconvoluted MS of **3av**.

**Conversion: 95%**

**Calculated Mass: 7833 Da; Found Mass: 7834 Da**

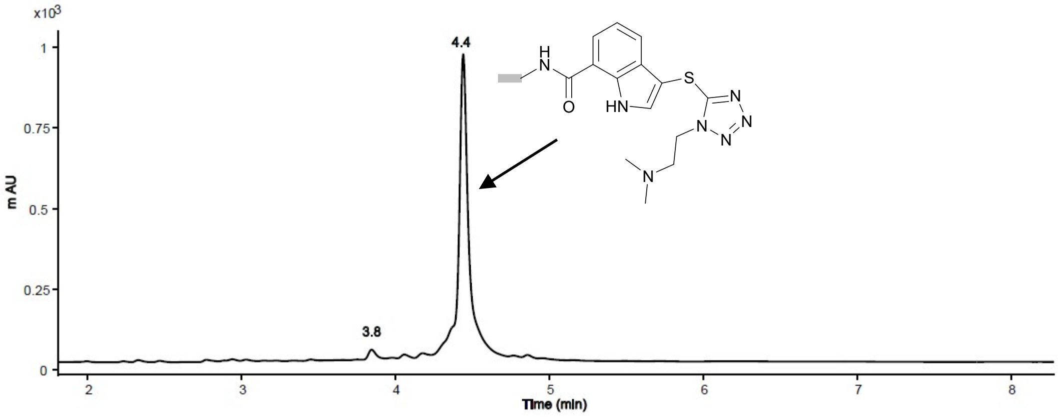


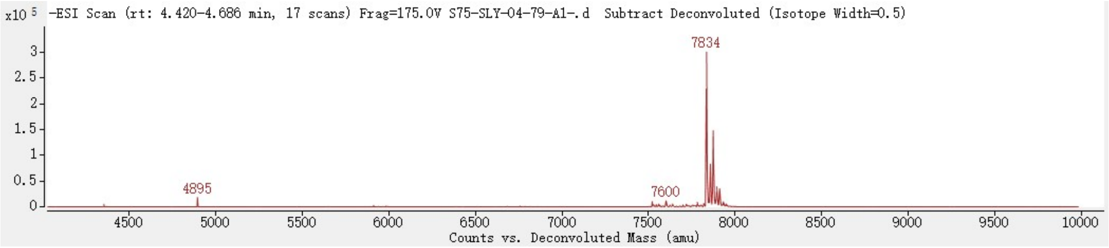

UPLC chromatograph and deconvoluted MS of **3aw**.

**Conversion: 87%**

**Calculated Mass: 7798 Da; Found Mass: 7798 Da**

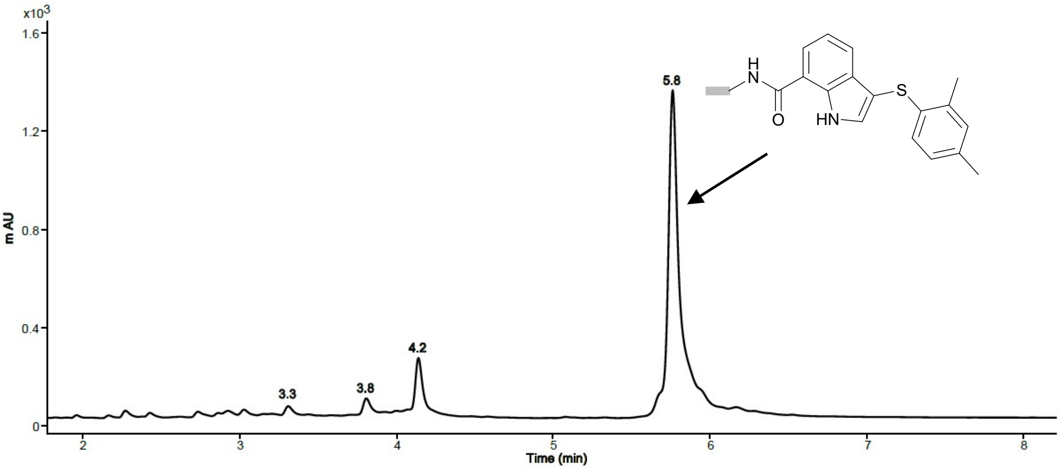


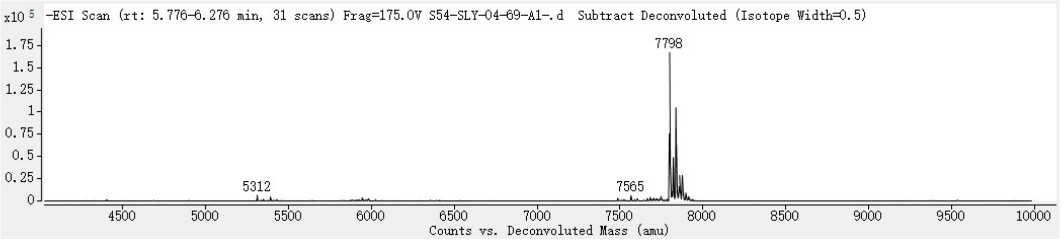


UPLC chromatograph and deconvoluted MS of **3ax**.

**Conversion: 85%**

**Calculated Mass: 7798 Da; Found Mass: 7799 Da**

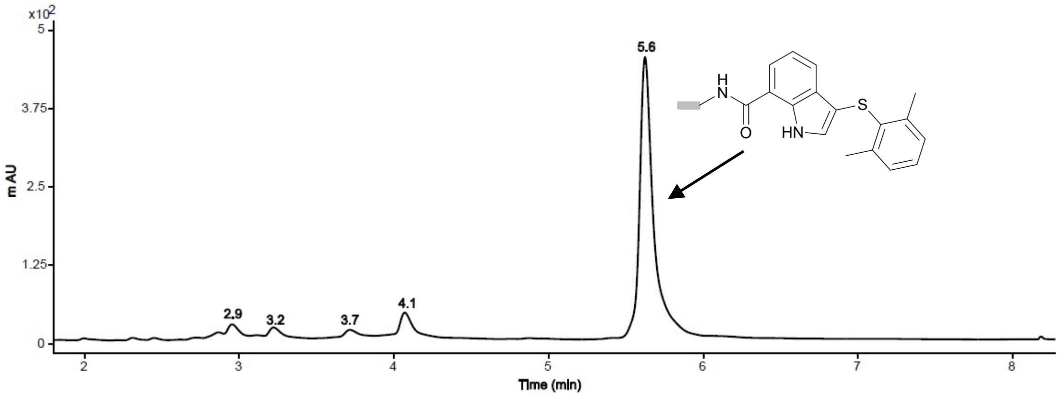


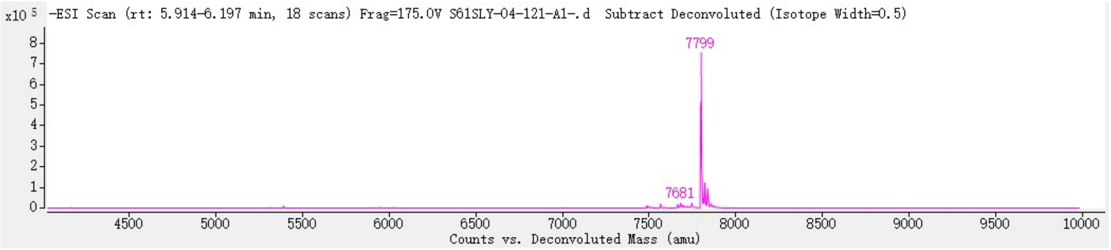

UPLC chromatograph and deconvoluted MS of **3ay**.

**Conversion: 78%**

**Calculated Mass: 7800 Da; Found Mass: 7800 Da**

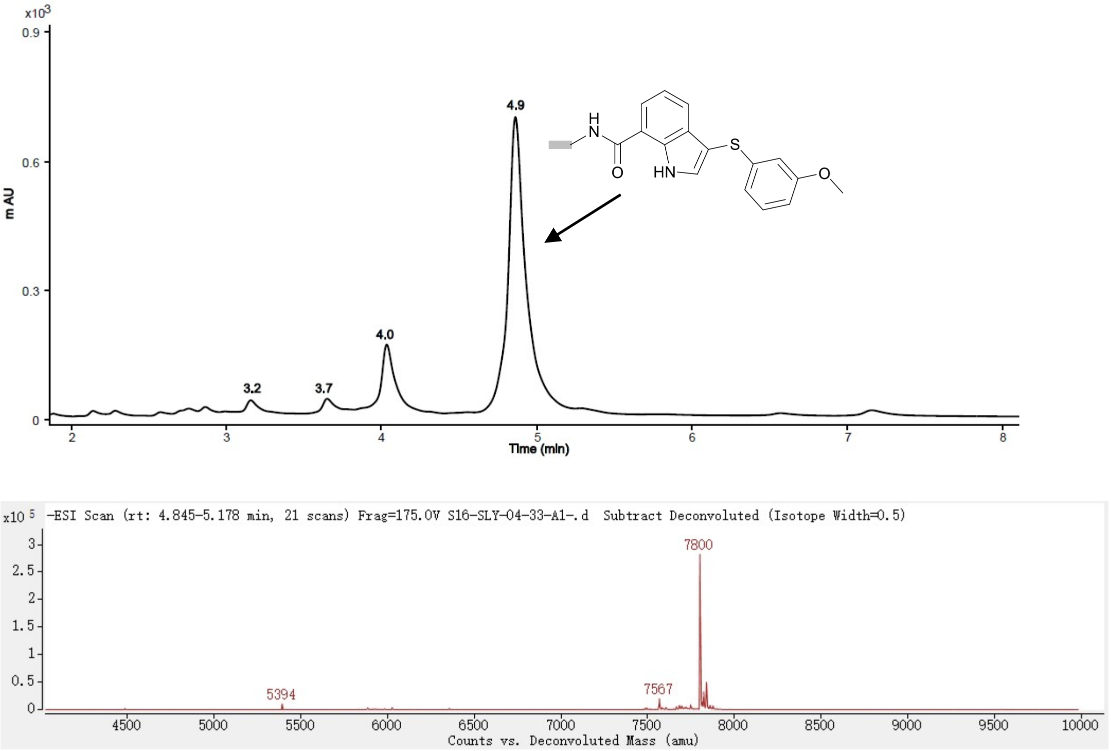


UPLC chromatograph and deconvoluted MS of **3az**

**Conversion: 30%**

**Calculated Mass: 7772 Da; Found Mass: 7772 Da**

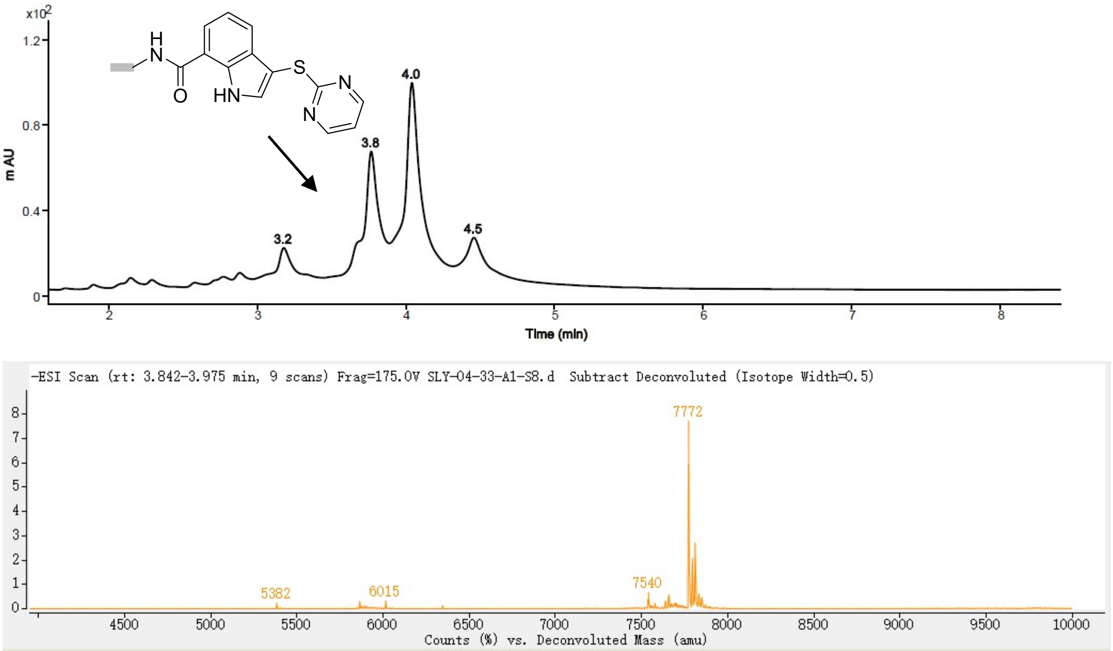

UPLC chromatograph and deconvoluted MS of **3ba**.
**Conversion: 62%**
**Calculated Mass: 7814 Da; Found Mass: 7814 Da**

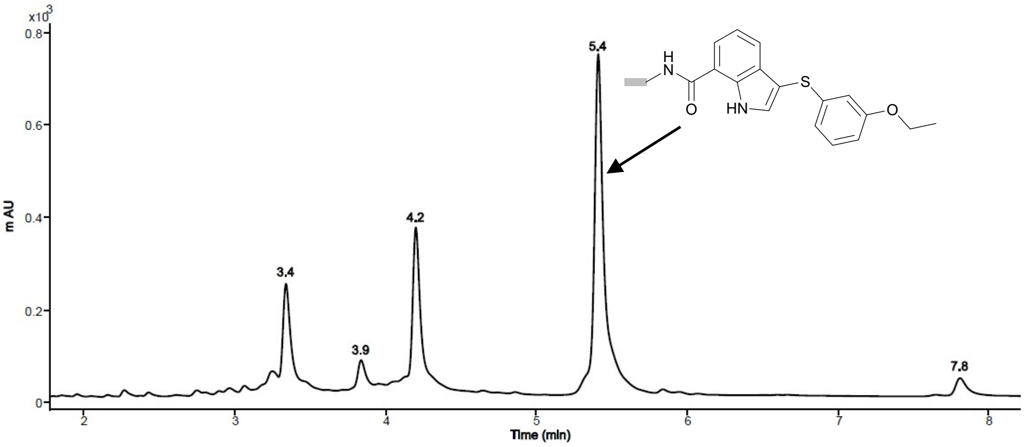


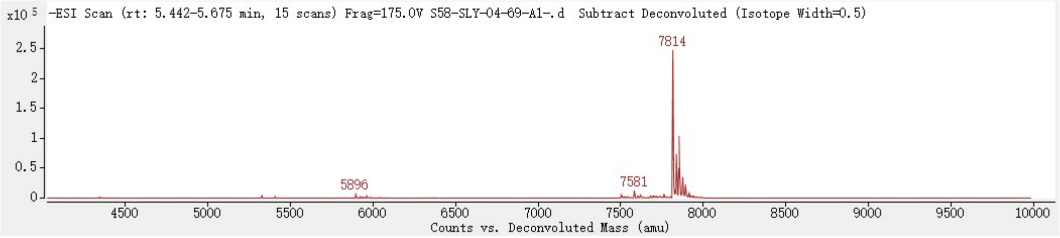


UPLC chromatograph and deconvoluted MS of **3bb**.
**Conversion: 90%**
**Calculated Mass: 7895 Da; Found Mass: 7896 Da**

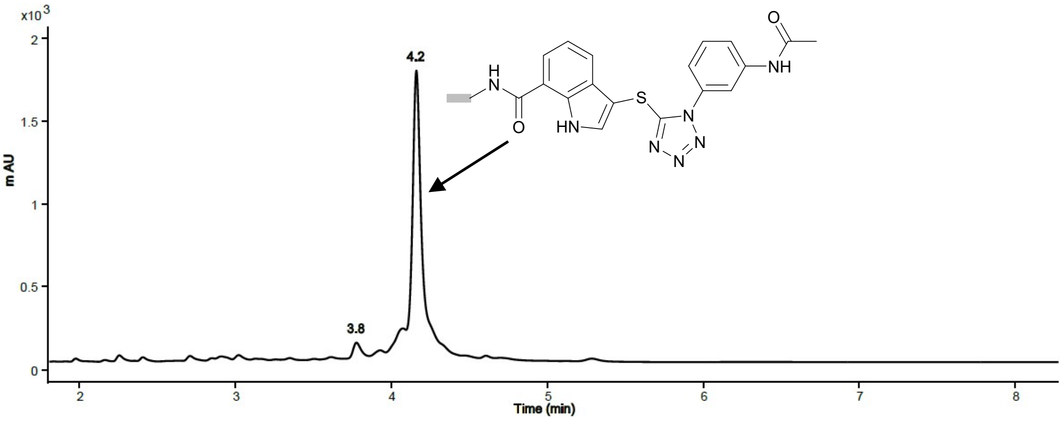


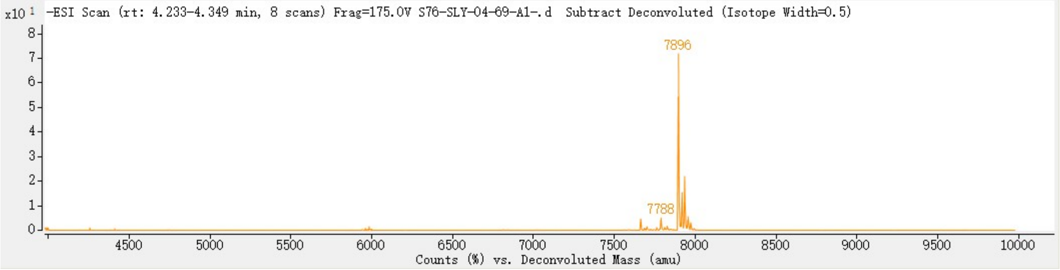

UPLC chromatograph and deconvoluted MS of **3bc**.

**Conversion: 61%**

**Calculated Mass: 7785 Da; Found Mass: 7786 Da**

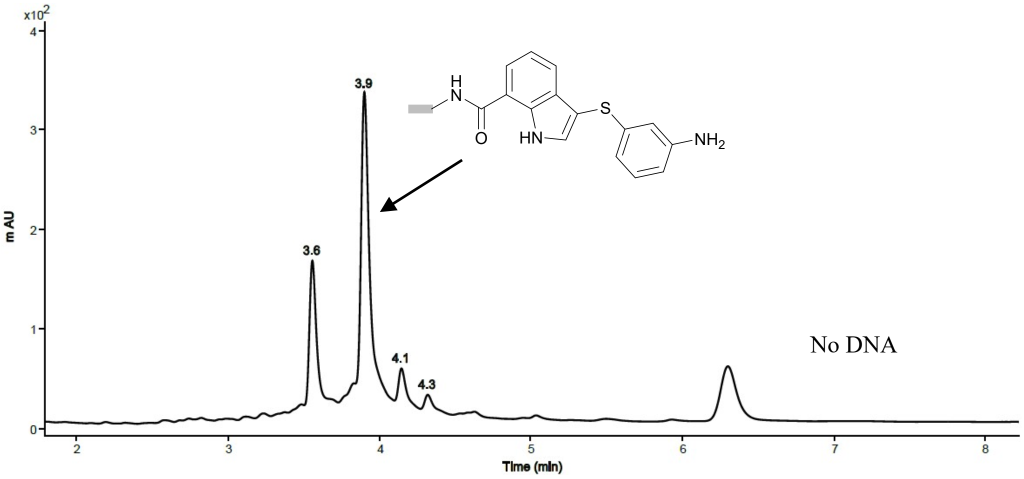


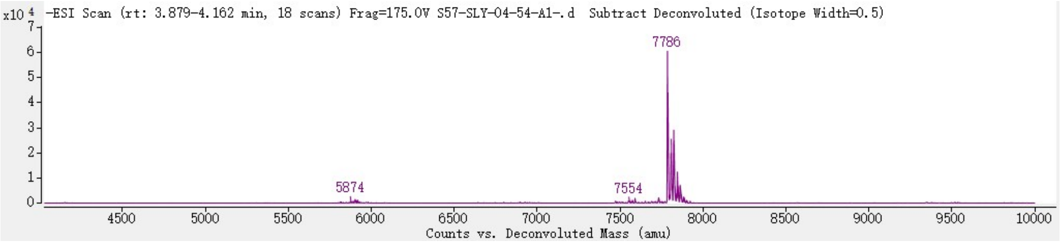


UPLC chromatograph and deconvoluted MS of **3bd**.

**Conversion: 79%**

**Calculated Mass: 7838 Da; Found Mass: 7839 Da**

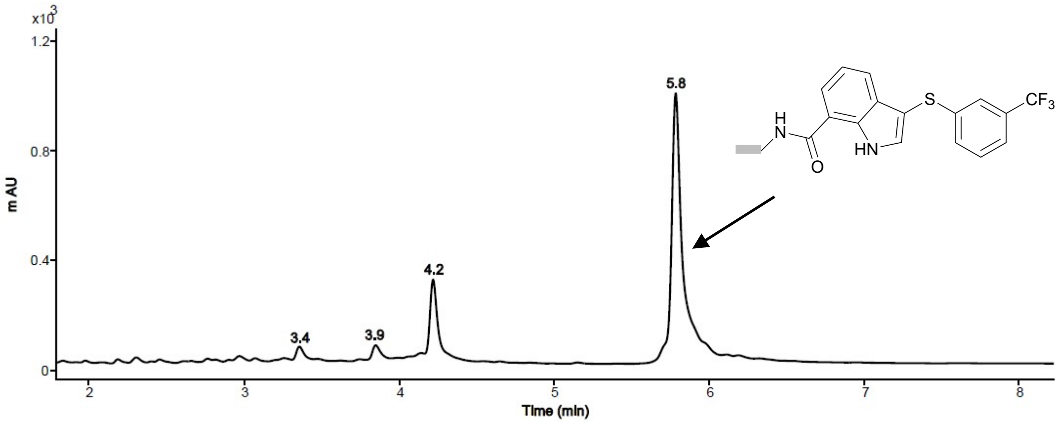


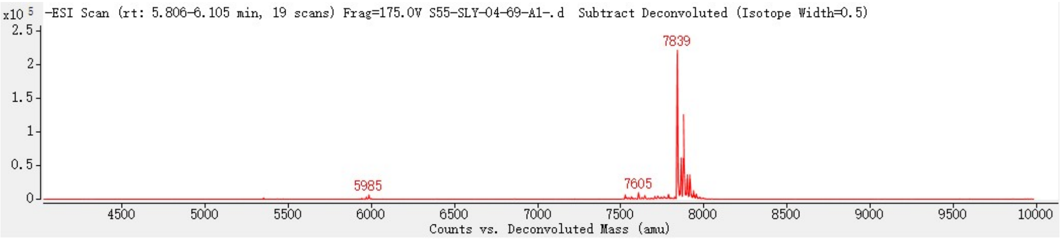

UPLC chromatograph and deconvoluted MS of **3be**.

**Conversion: 89%**

**Calculated Mass: 7788 Da; Found Mass: 7789 Da**

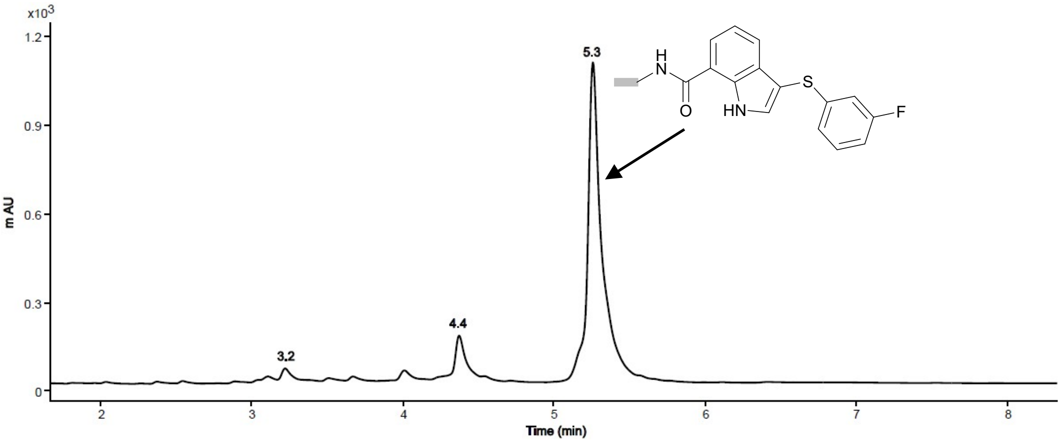


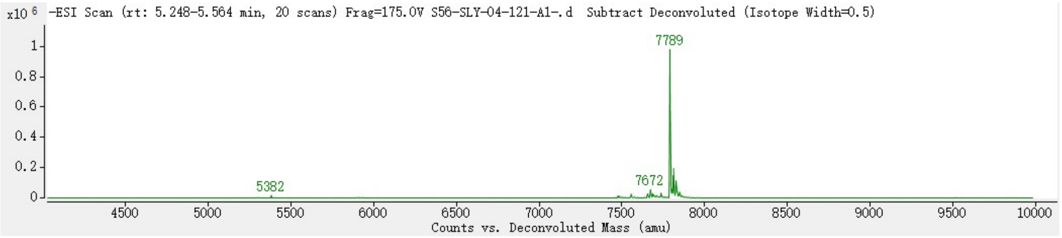


UPLC chromatograph and deconvoluted MS of **3bf**.

**Conversion: 80%**

**Calculated Mass: 7806 Da; Found Mass: 7806 Da**

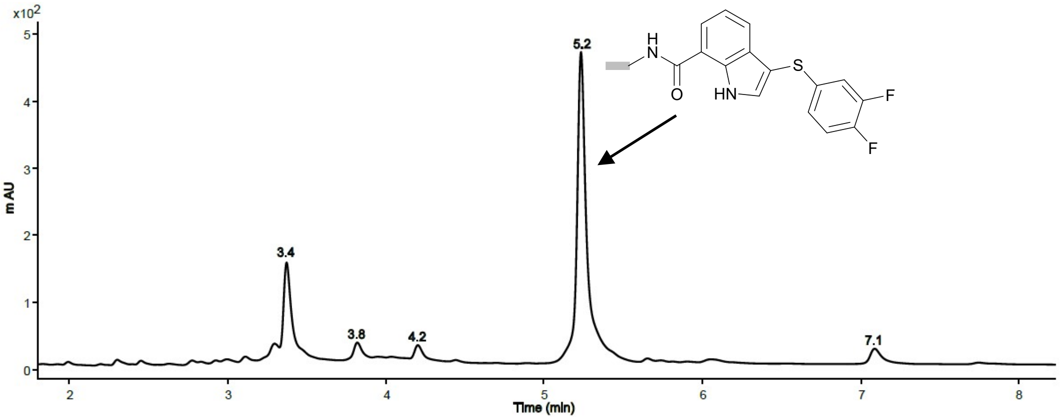


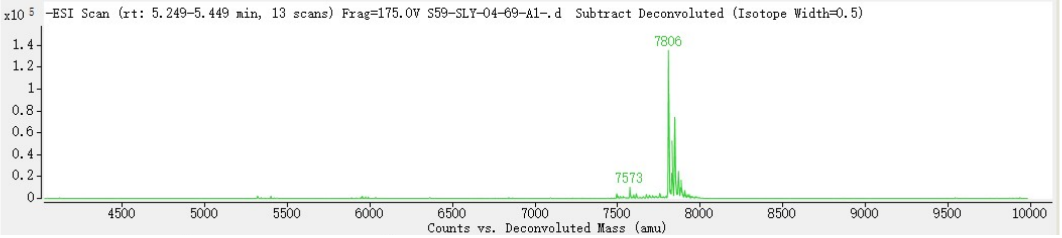

UPLC chromatograph and deconvoluted MS of **3bg**.

**Conversion: 89%**

**Calculated Mass: 7804 Da; Found Mass: 7805 Da**

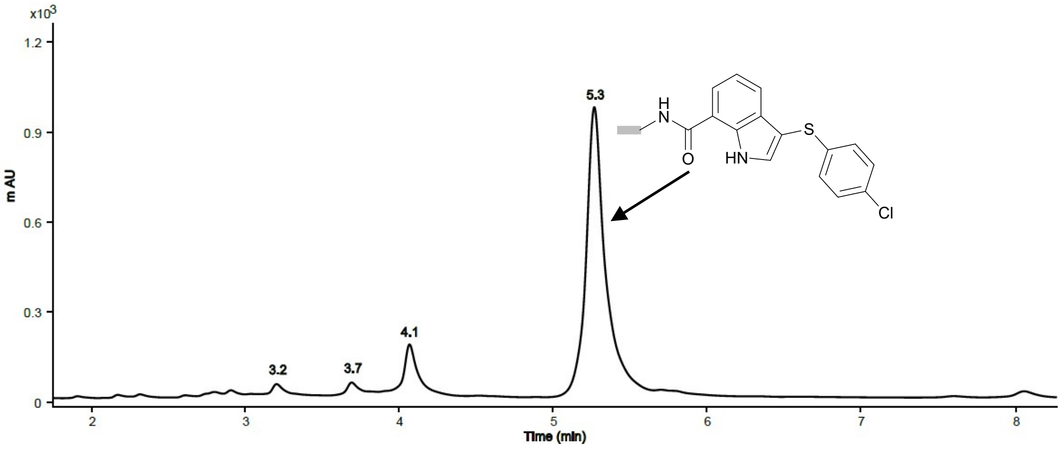


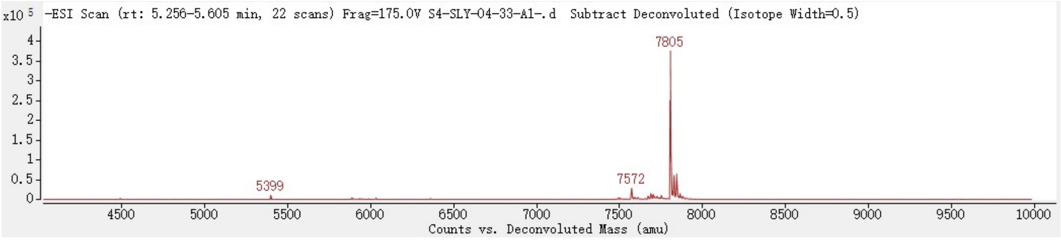


UPLC chromatograph and deconvoluted MS of **3bh**.

**Conversion: 95%**

**Calculated Mass: 7805 Da; Found Mass: 7805 Da**

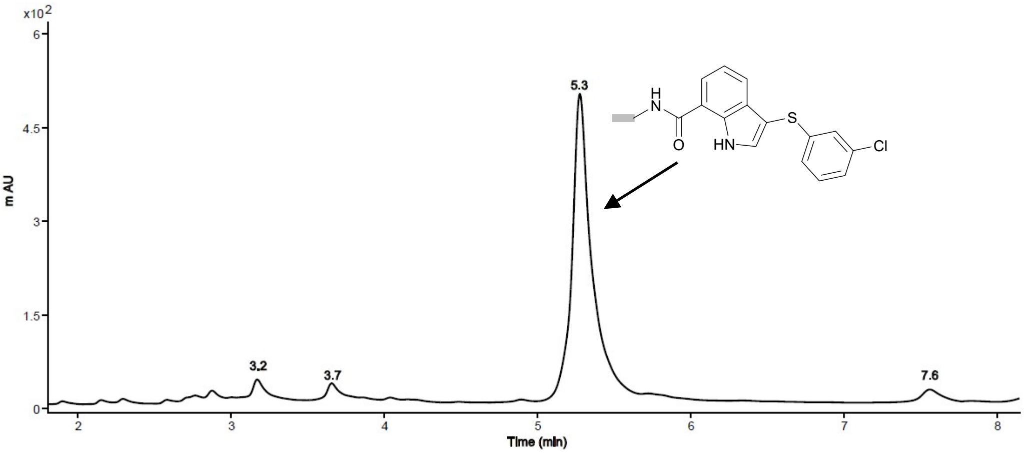


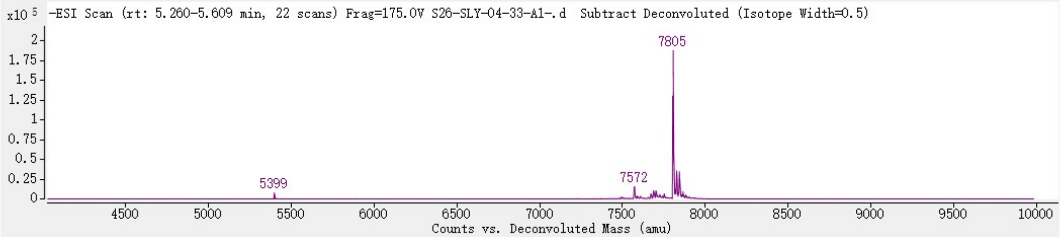

UPLC chromatograph and deconvoluted MS of **3bi**.

**Conversion: 93%**

**Calculated Mass: 7839 Da; Found Mass: 7839 Da**

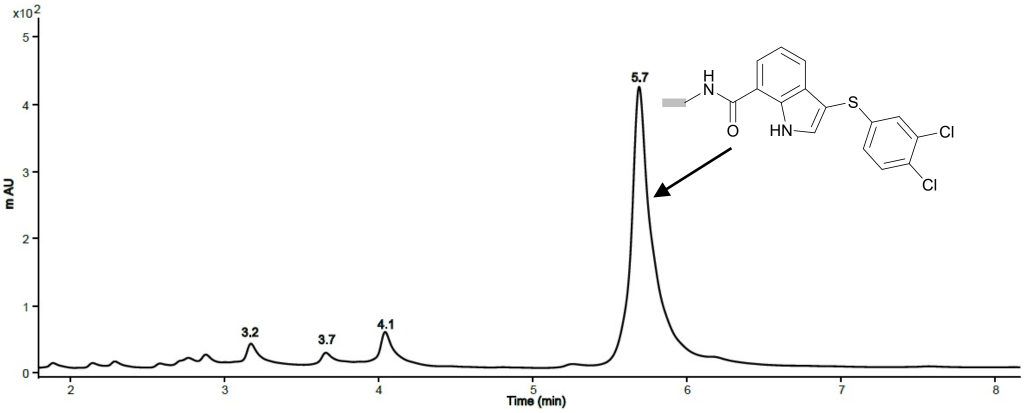


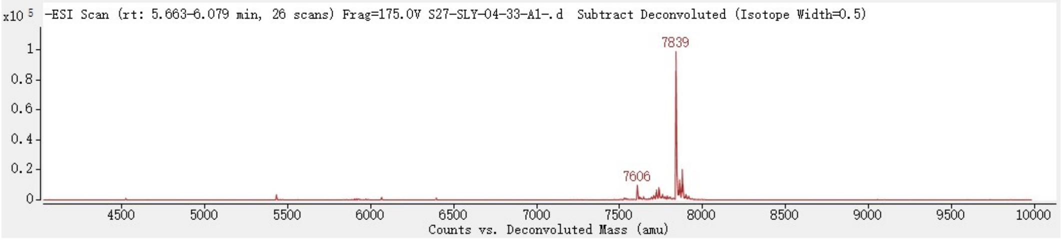


UPLC chromatograph and deconvoluted MS of 2**bj**.

**Conversion: 83%**

**Calculated Mass: 7889 Da; Found Mass: 7890 Da**

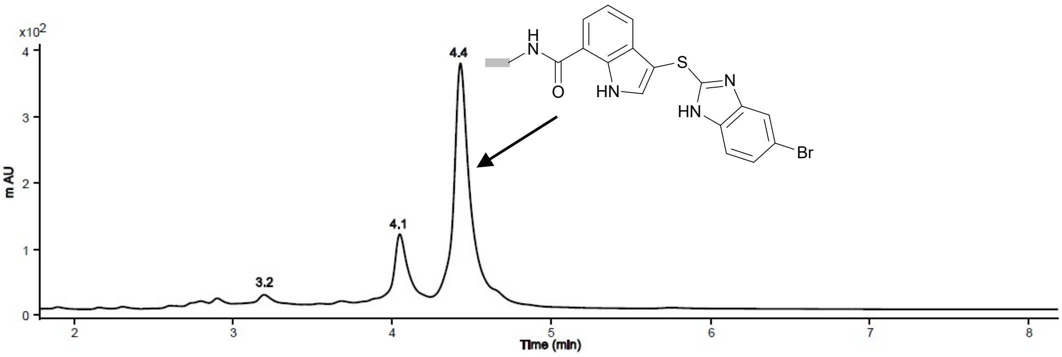


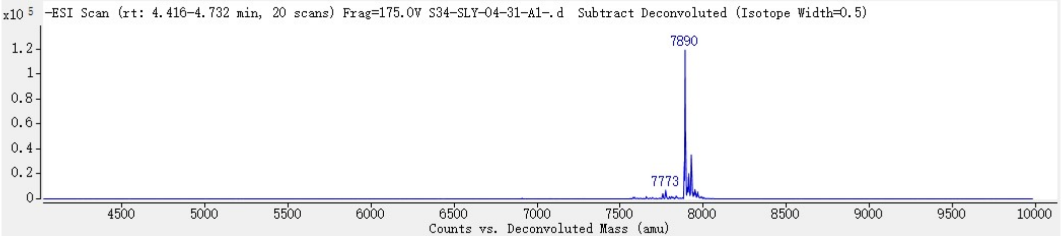

UPLC chromatograph and deconvoluted MS of **3bk**.

**Conversion: 76%**

**Calculated Mass: 7828 Da; Found Mass: 7829 Da**

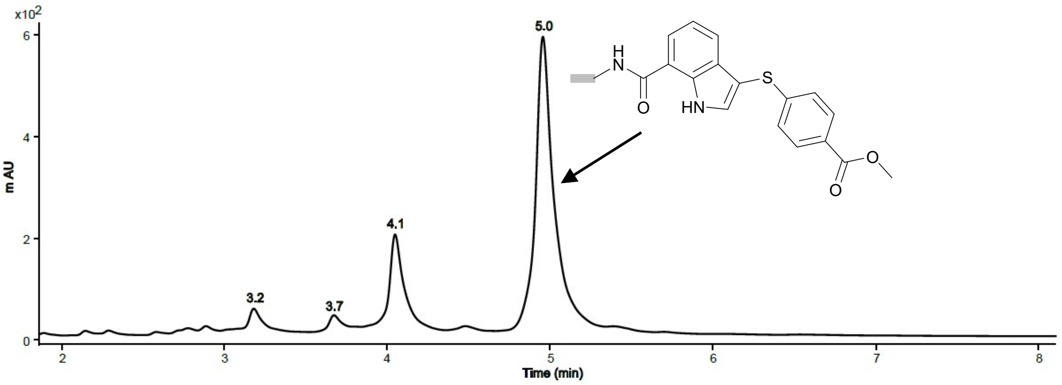


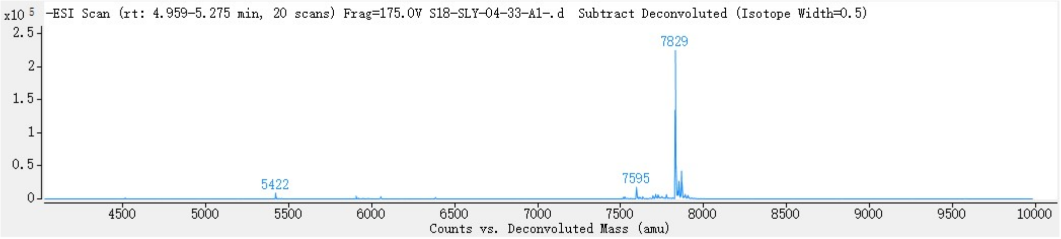


UPLC chromatograph and deconvoluted MS of **3bl**.

**Conversion: >95%**

**Calculated Mass: 7828 Da; Found Mass: 7829 Da**

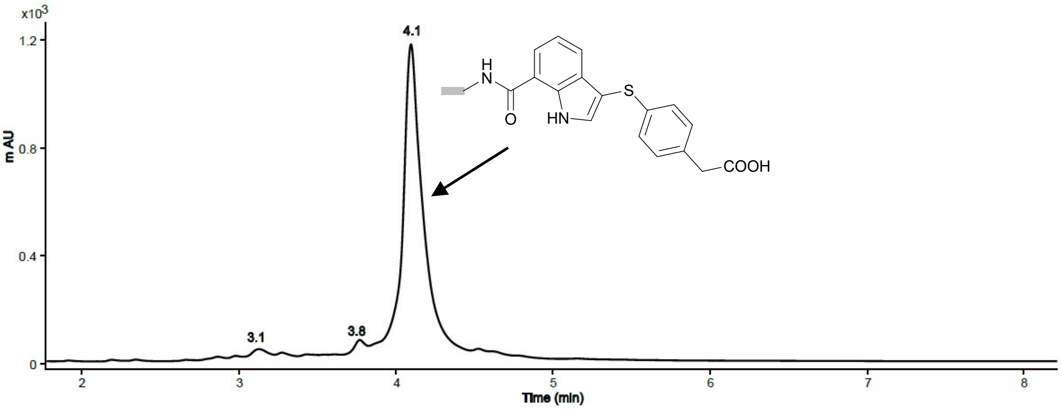


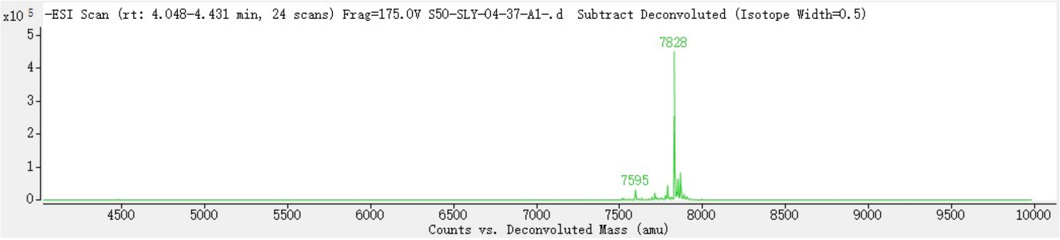

UPLC chromatograph and deconvoluted MS of **3bm**.

**Conversion: 85%**

**Calculated Mass: 7820 Da; Found Mass: 7820 Da**

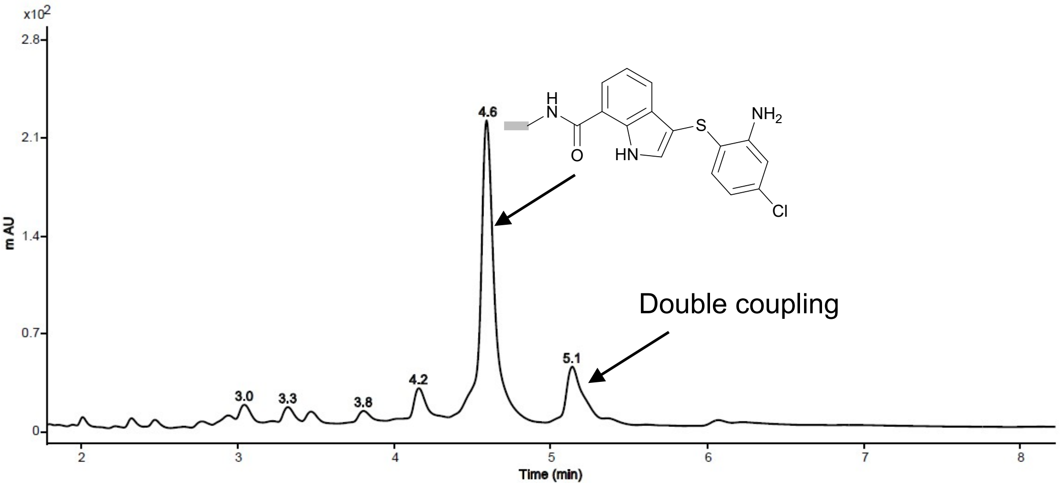


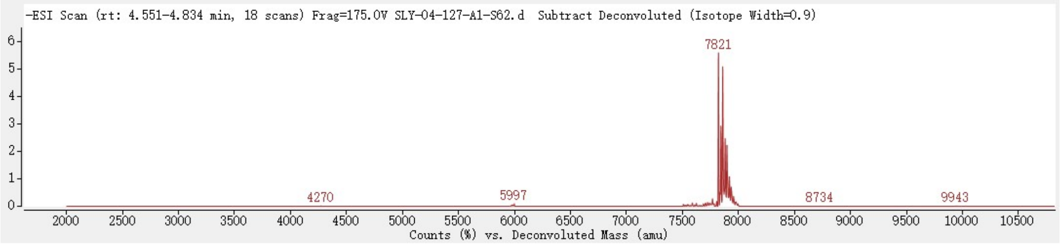


UPLC chromatograph and deconvoluted MS of **3bn**.

**Conversion: 5%**

**Calculated Mass: 7815 Da; Found Mass: 7815 Da**

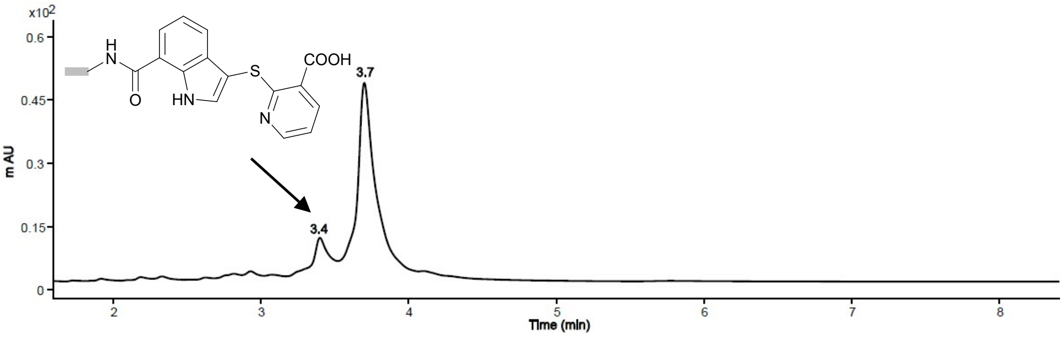


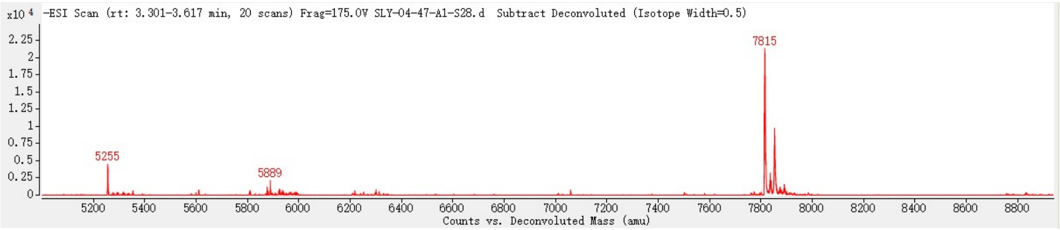

UPLC chromatograph and deconvoluted MS of **3bo**.

**Conversion: >95%**

**Calculated Mass: 7840 Da; Found Mass: 7840 Da**

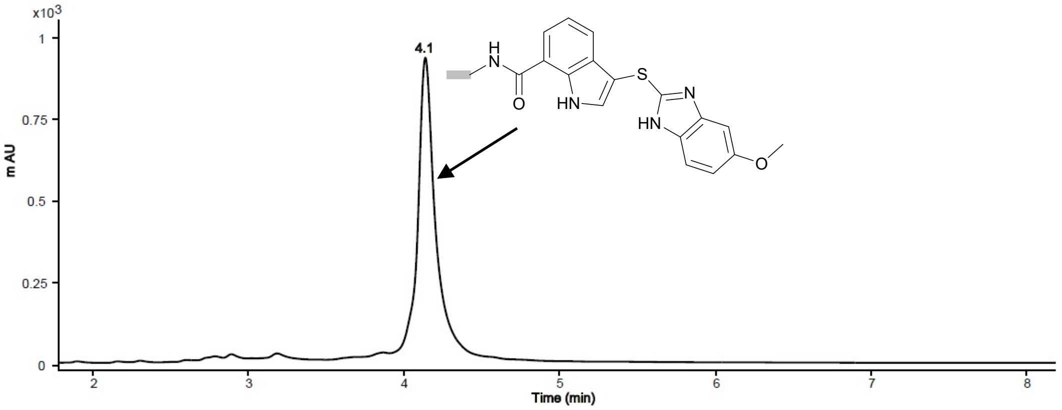


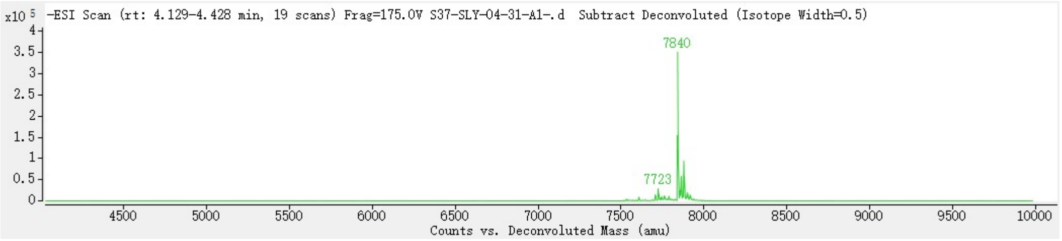


UPLC chromatograph and deconvoluted MS of **3bp**.

**Conversion: 86%**

**Calculated Mass: 7824 Da; Found Mass: 7825 Da**

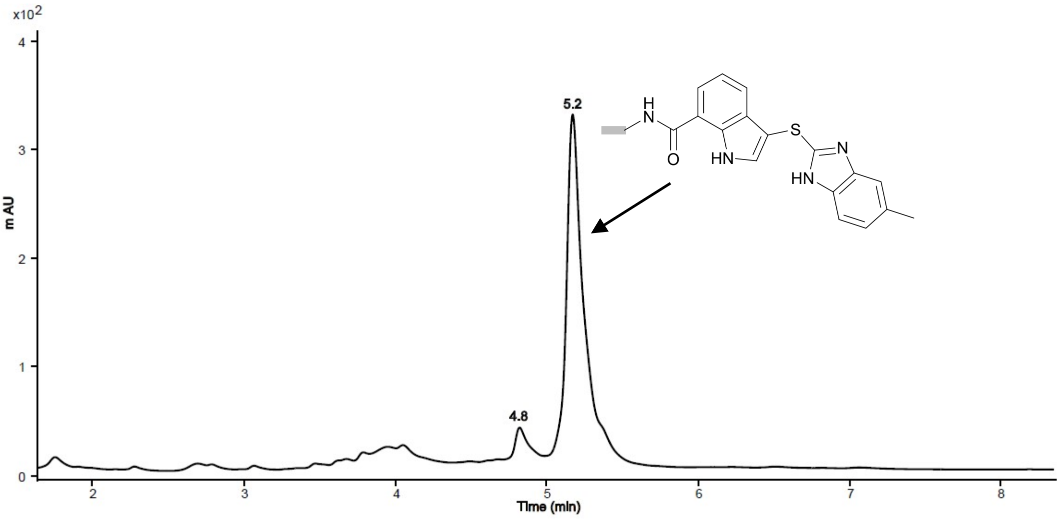


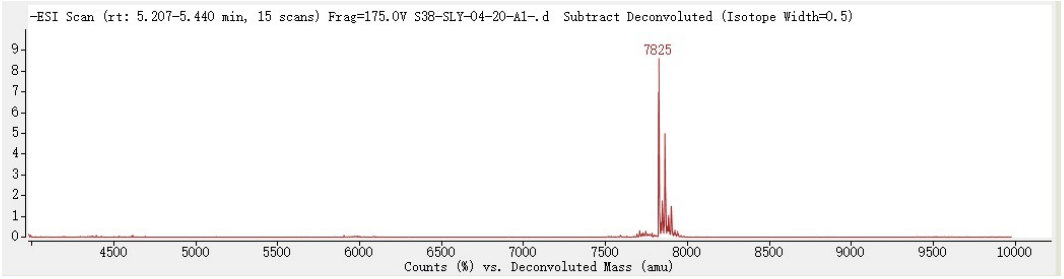

## 6.2 Substrate scope of the sulfide formation from 1b and thiols on DNA

UPLC chromatograph and deconvoluted MS of **5a.**

**Conversion: 86%**

**Calculated Mass: 7827 Da; Found Mass: 7827 Da**

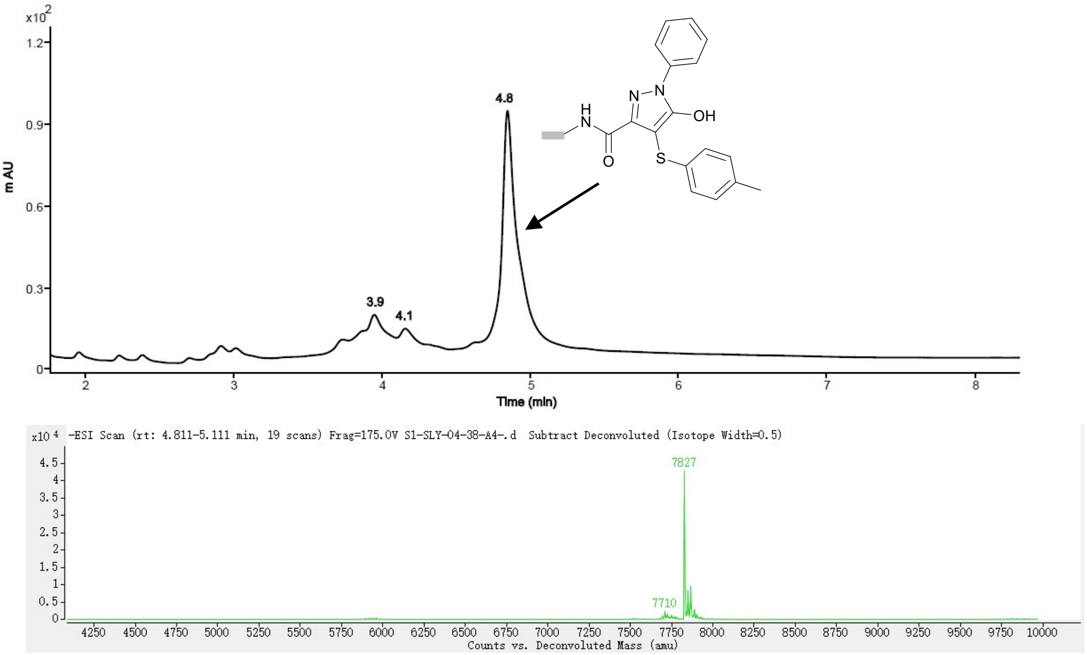


UPLC chromatograph and deconvoluted MS of **5b.**

**Conversion: 77%**

**Calculated Mass: 7827 Da; Found Mass: 7827 Da**

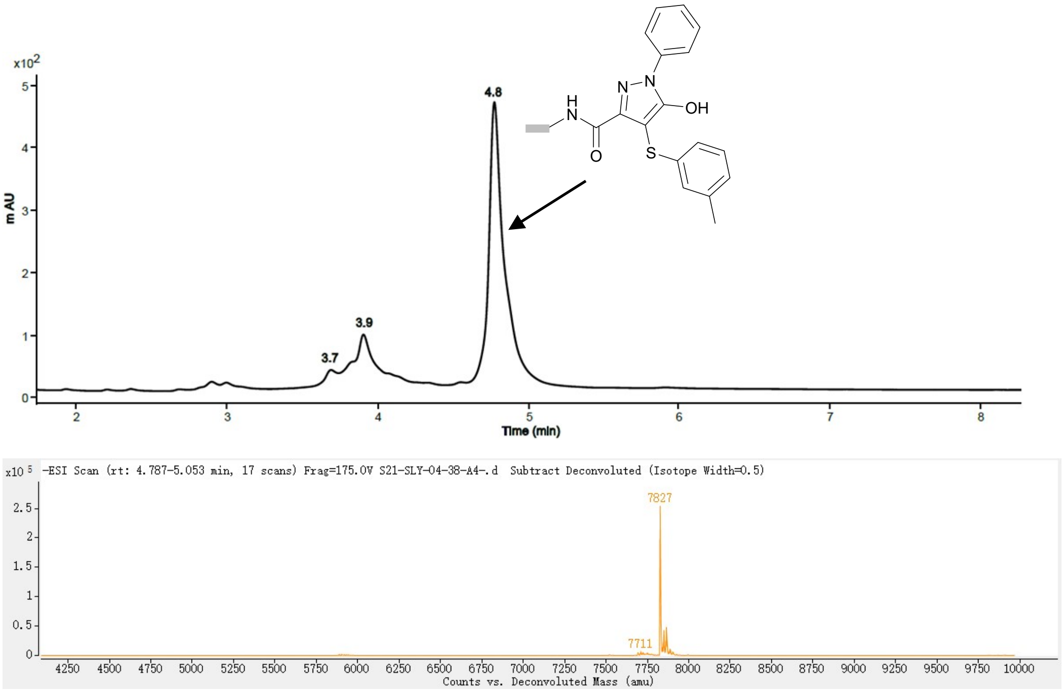

UPLC chromatograph and deconvoluted MS of **5c.**

**Conversion: 91%**

**Calculated Mass: 7827 Da; Found Mass: 7829 Da**

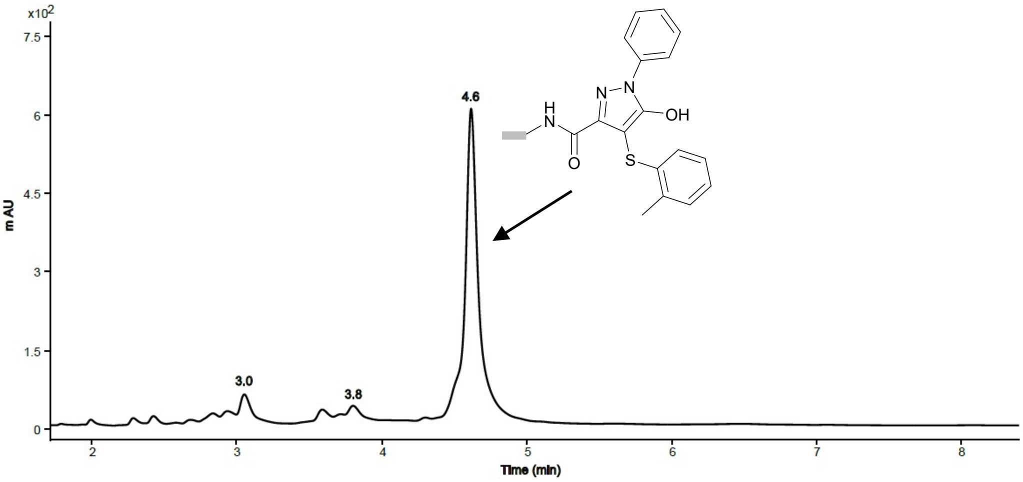


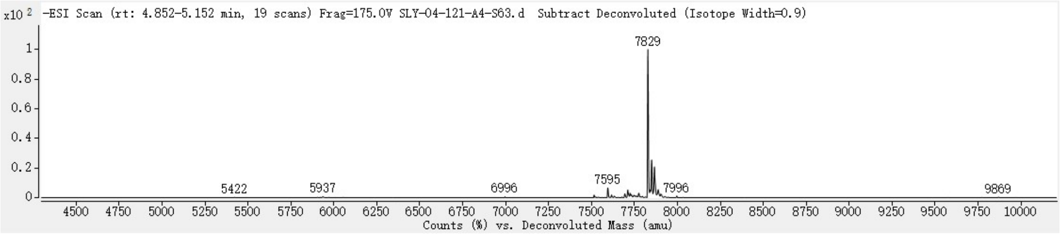


UPLC chromatograph and deconvoluted MS of **5d.**

**Conversion: 87%**

**Calculated Mass: 7829 Da; Found Mass: 7830 Da**

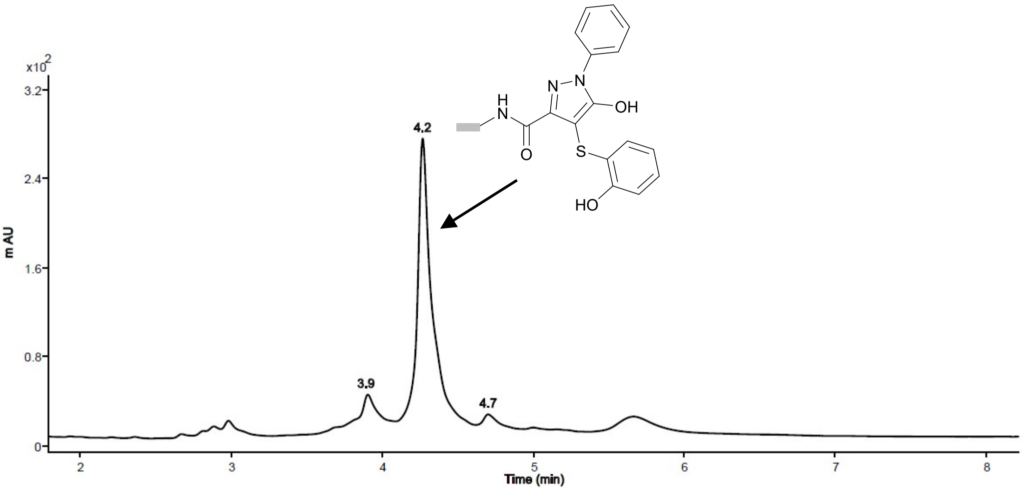


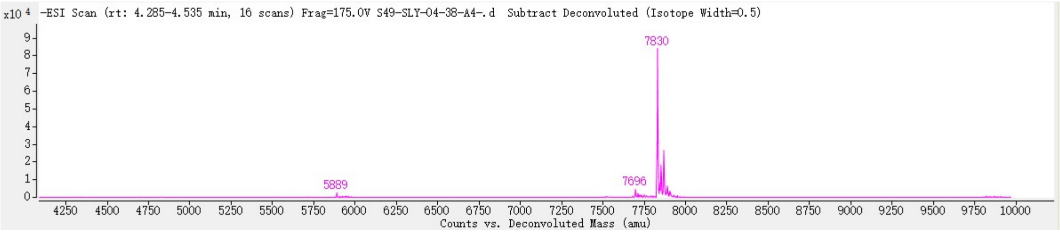

UPLC chromatograph and deconvoluted MS of **5f.**

**Conversion: 84%**

**Calculated Mass: 7891 Da; Found Mass: 7893 Da**

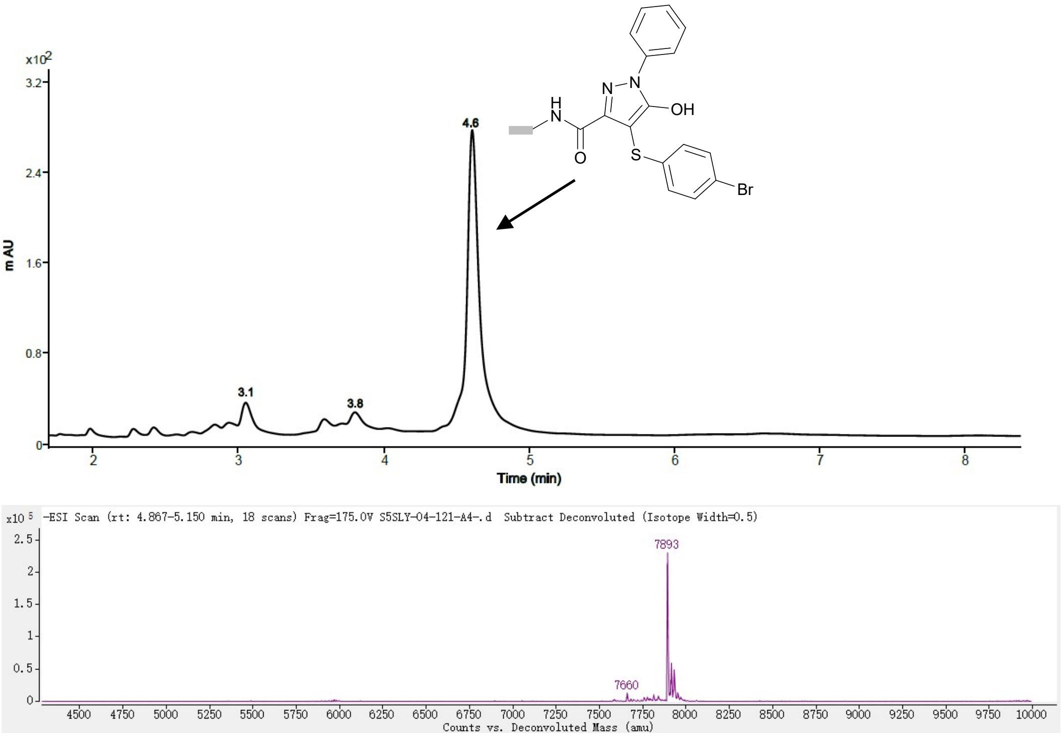


UPLC chromatograph and deconvoluted MS of **5g.**

**Conversion: 44%**

**Calculated Mass: 7858 Da; Found Mass: 7858 Da**

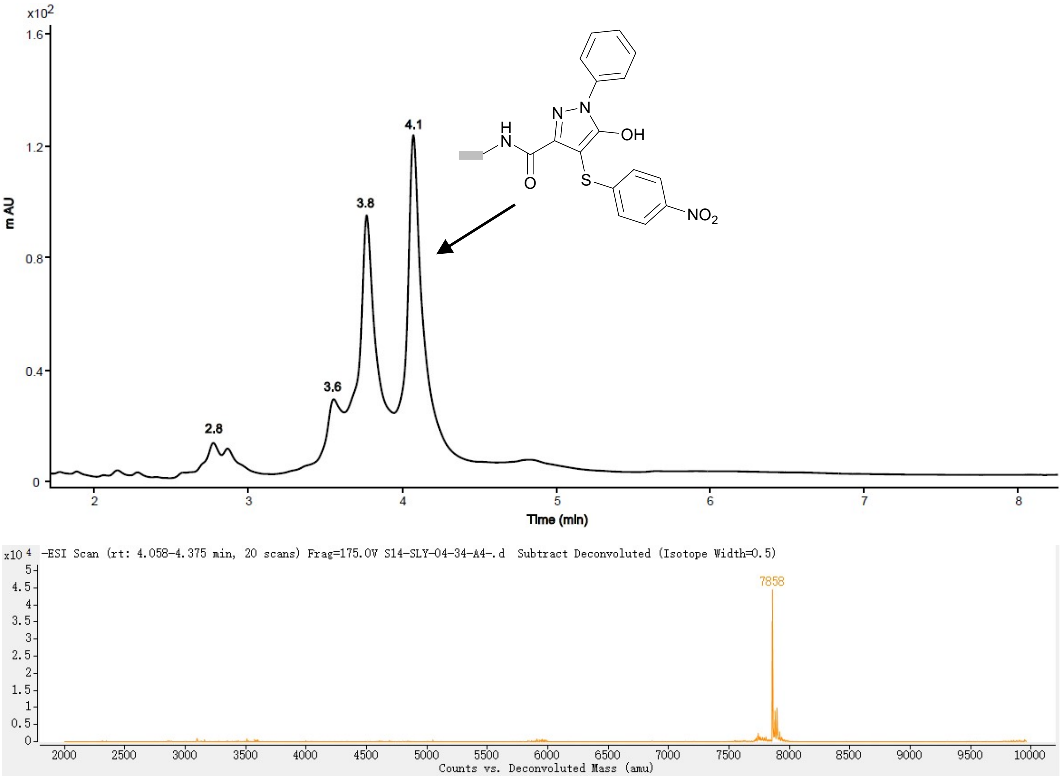

UPLC chromatograph and deconvoluted MS of **5h.**

**Conversion: 40%**

**Calculated Mass: 7863 Da; Found Mass: 7864 Da**

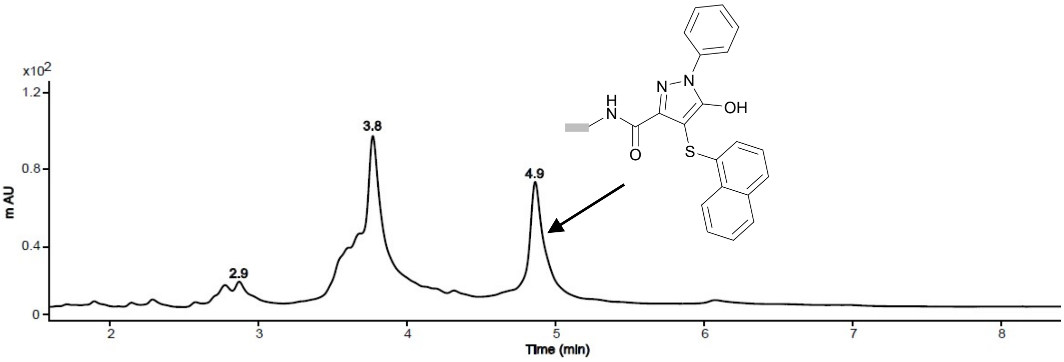


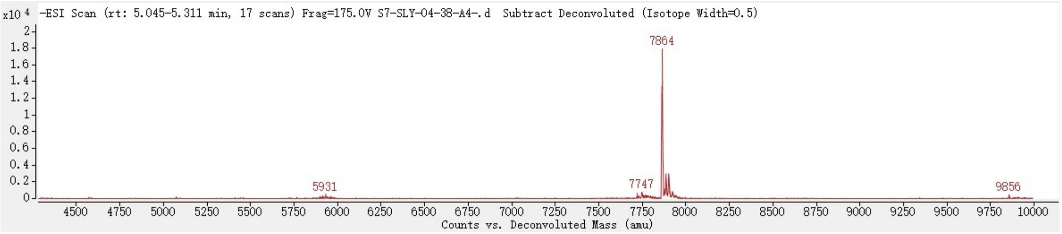


UPLC chromatograph and deconvoluted MS of **5i.**

**Conversion: 82%**

**Calculated Mass: 7815 Da; Found Mass: 7815 Da**

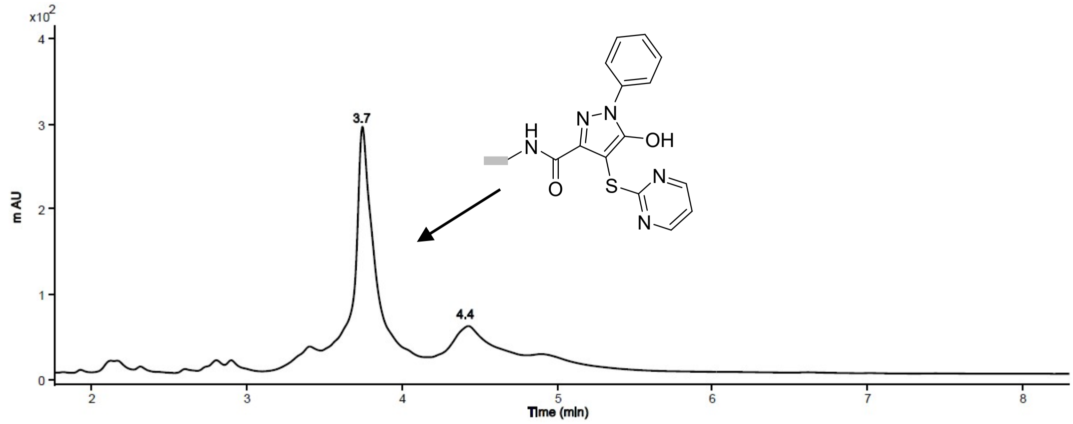


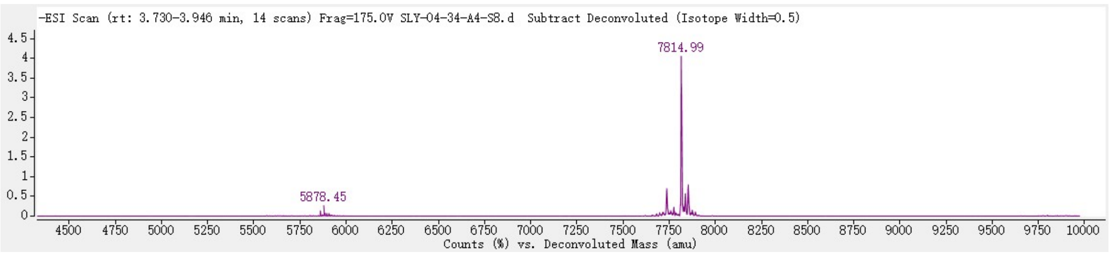

UPLC chromatograph and deconvoluted MS of **5j.**

**Conversion: >95%**

**Calculated Mass: 7835 Da; Found Mass: 7836 Da**

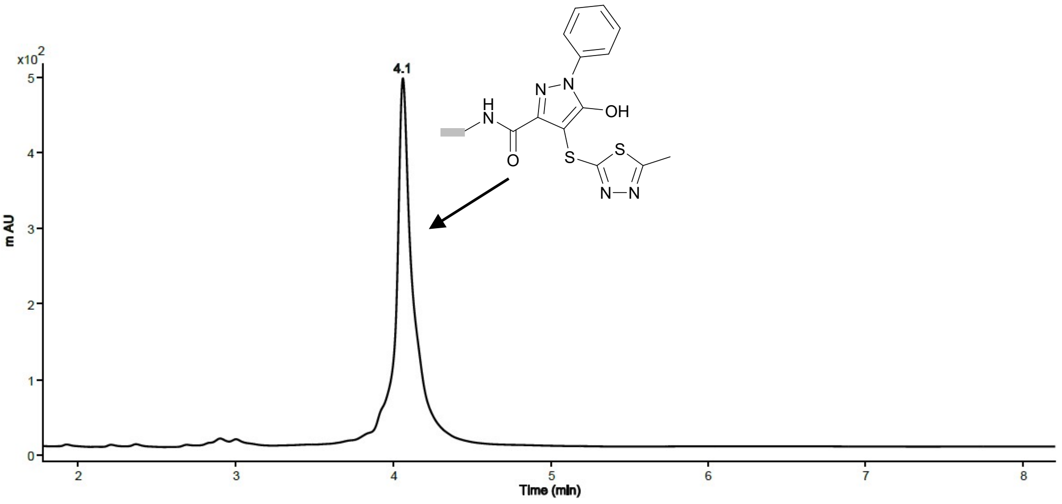


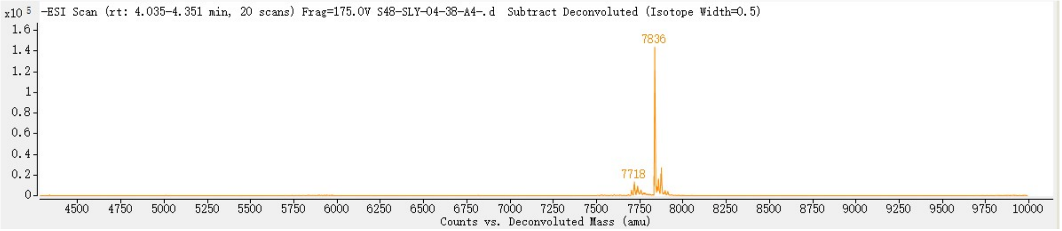


UPLC chromatograph and deconvoluted MS of **5k.**

**Conversion: 89%**

**Calculated Mass: 7819 Da; Found Mass: 7820 Da**

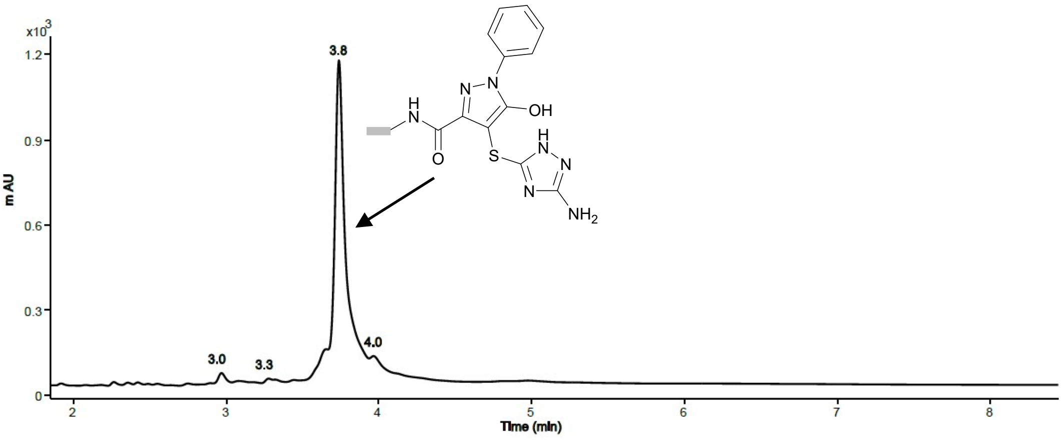


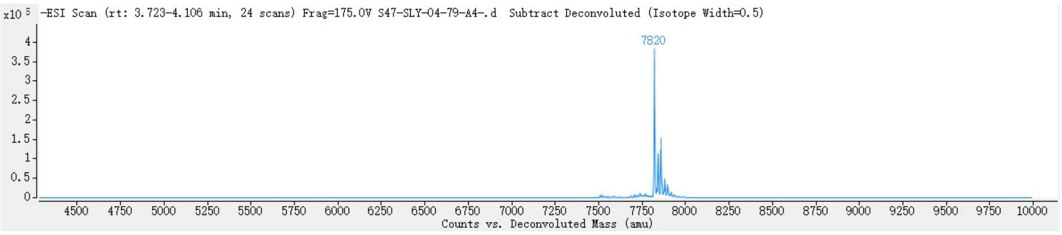

UPLC chromatograph and deconvoluted MS of **5l.**

**Conversion: 92%**

**Calculated Mass: 7854 Da; Found Mass: 7855 Da**

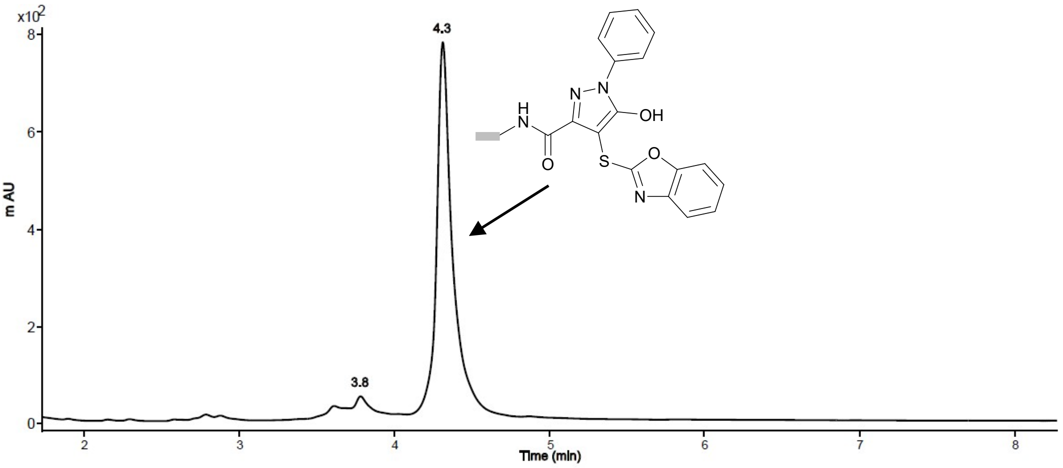


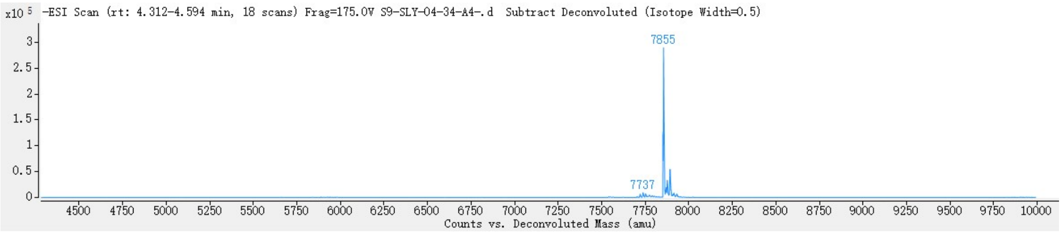


UPLC chromatograph and deconvoluted MS of **5m.**

**Conversion: 94%**

**Calculated Mass: 7899 Da; Found Mass: 7889 Da**

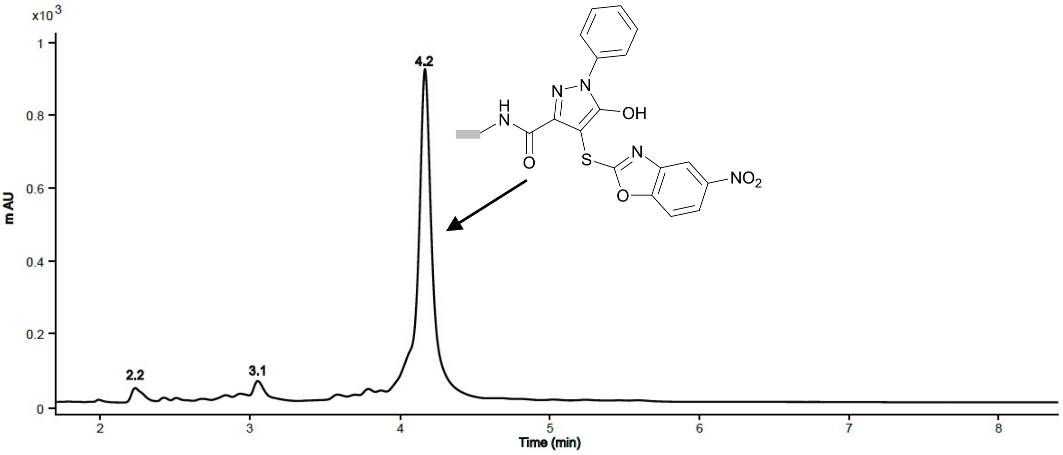


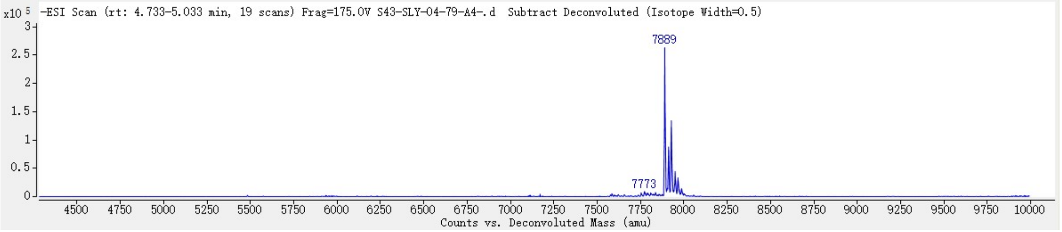

UPLC chromatograph and deconvoluted MS of **5n.**

**Conversion: 88%**

**Calculated Mass: 7814 Da; Found Mass: 7814 Da**

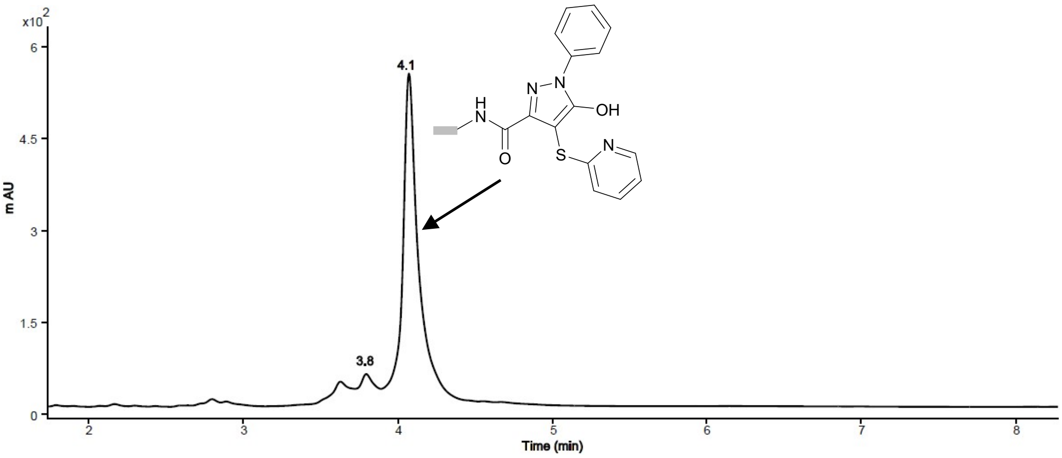


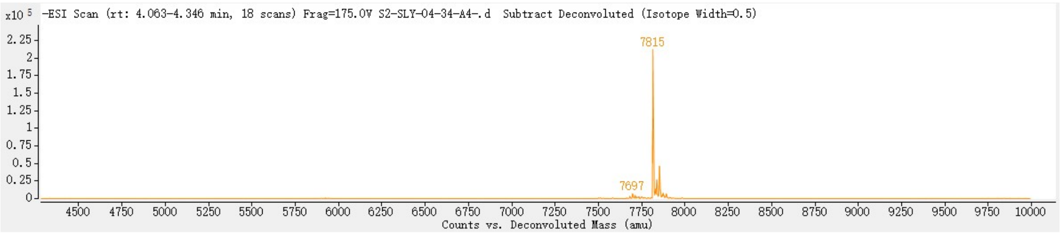


UPLC chromatograph and deconvoluted MS of **5o.**

**Conversion: >95%**

**Calculated Mass: 7820 Da; Found Mass: 7821 Da**

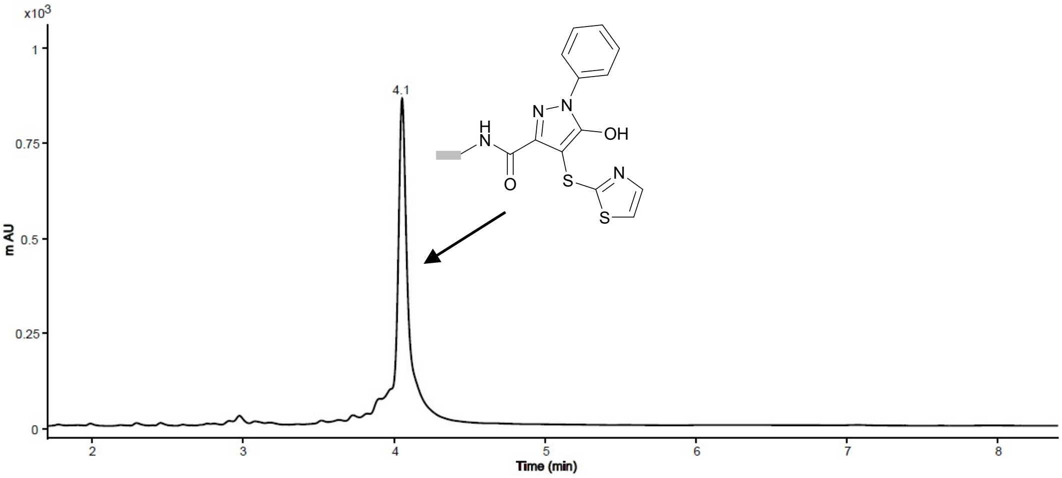


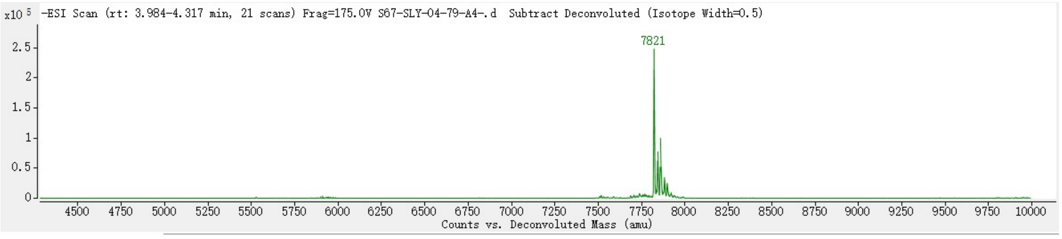

UPLC chromatograph and deconvoluted MS of **5p.**

**Conversion: >95%**

**Calculated Mass: 7803 Da; Found Mass: 7803 Da**

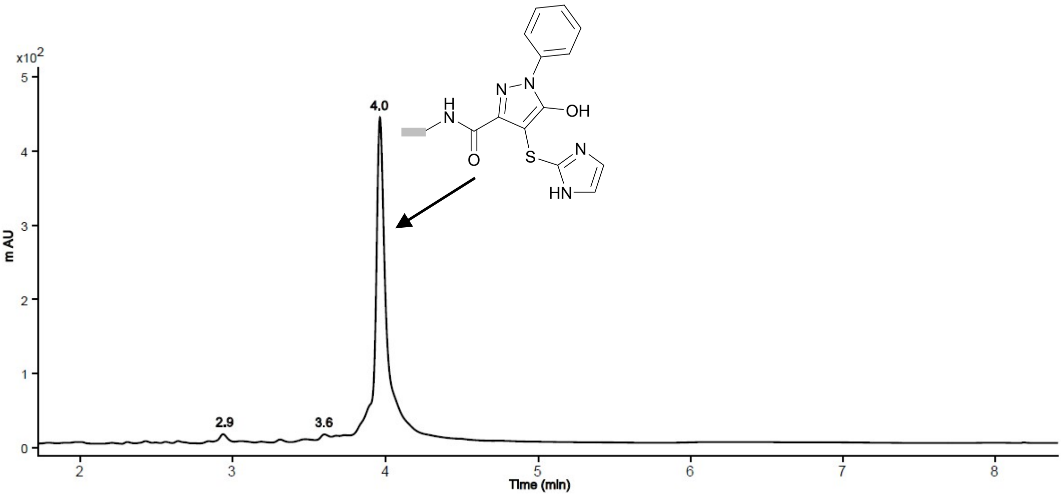


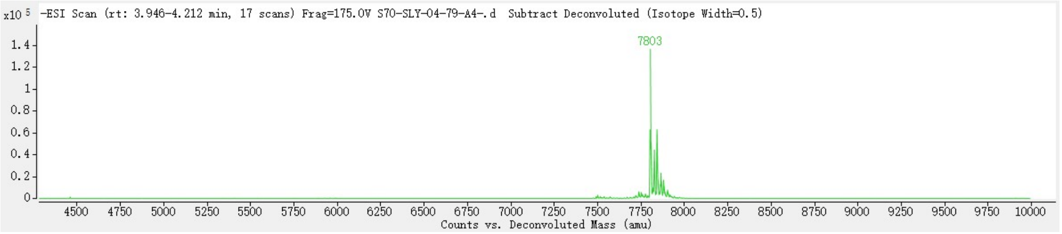


UPLC chromatograph and deconvoluted MS of **5q.**

**Conversion: 91%**

**Calculated Mass: 7884 Da; Found Mass: 7885 Da**

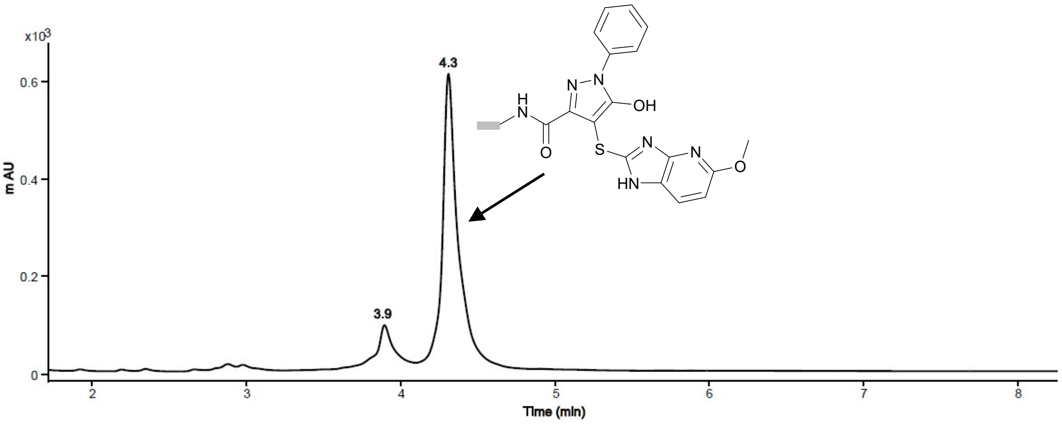


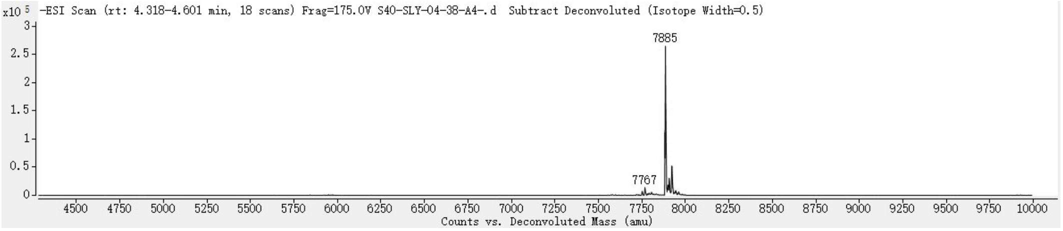

UPLC chromatograph and deconvoluted MS of **5r.**

**Conversion: 88%**

**Calculated Mass: 7853 Da; Found Mass: 7853 Da**

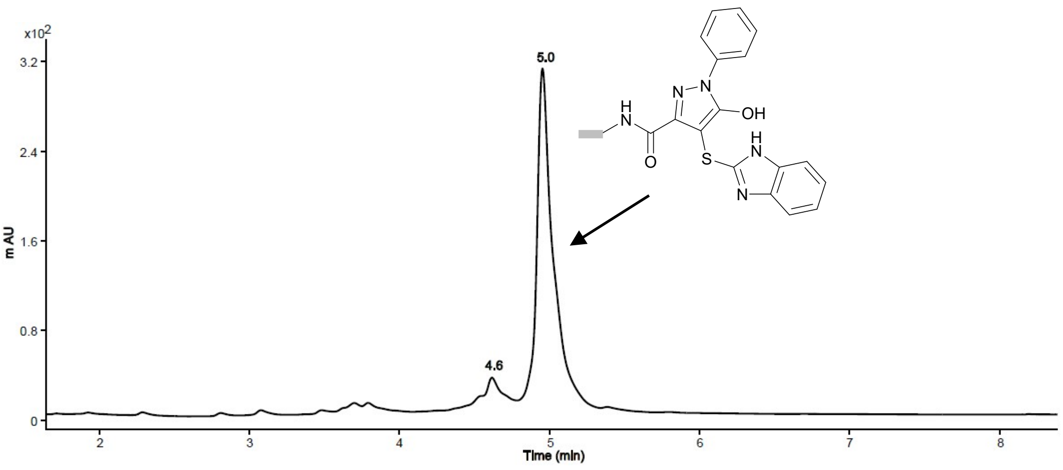


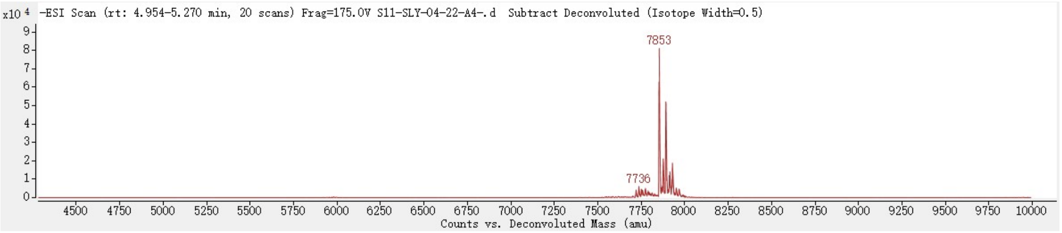


UPLC chromatograph and deconvoluted MS of **5s.**

**Conversion: >95%**

**Calculated Mass: 7868 Da; Found Mass: 7868 Da**

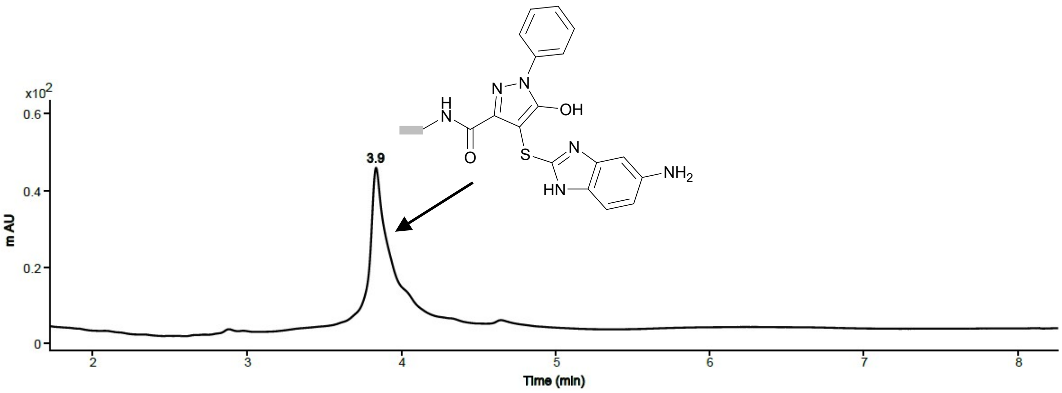


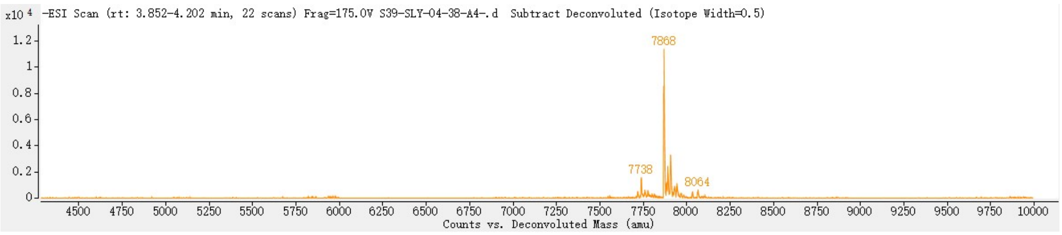

UPLC chromatograph and deconvoluted MS of **5t.**

**Conversion: >95%**

**Calculated Mass: 7919 Da; Found Mass: 7920 Da**

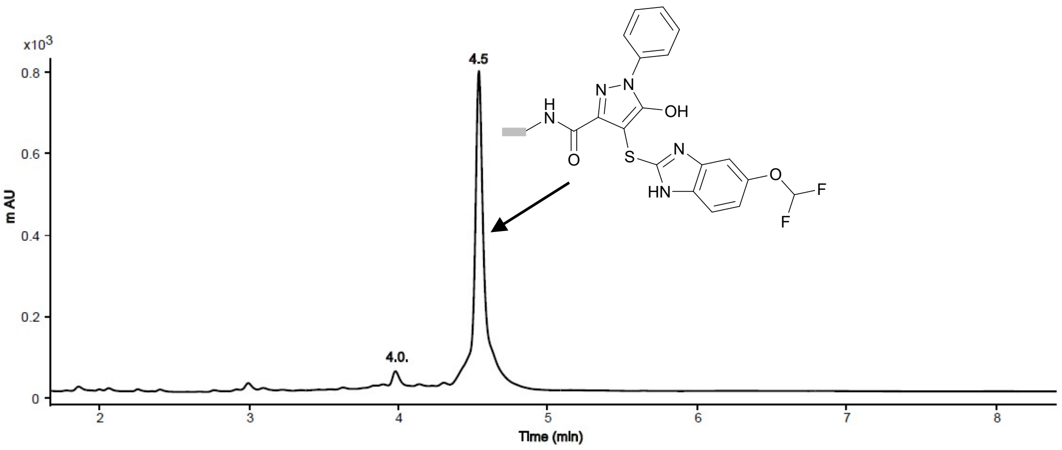


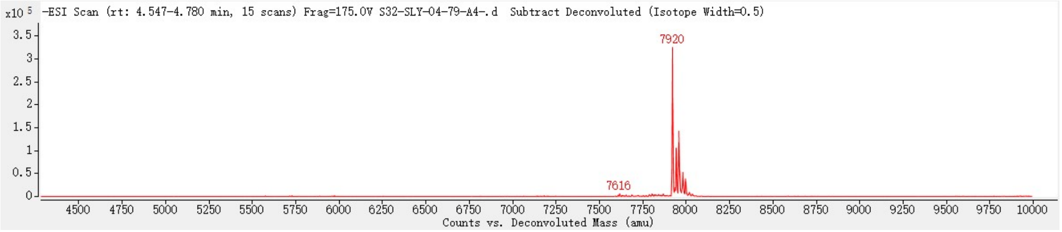


UPLC chromatograph and deconvoluted MS of **5u.**

**Conversion: >95%**

**Calculated Mass: 7897 Da; Found Mass: 7898 Da**

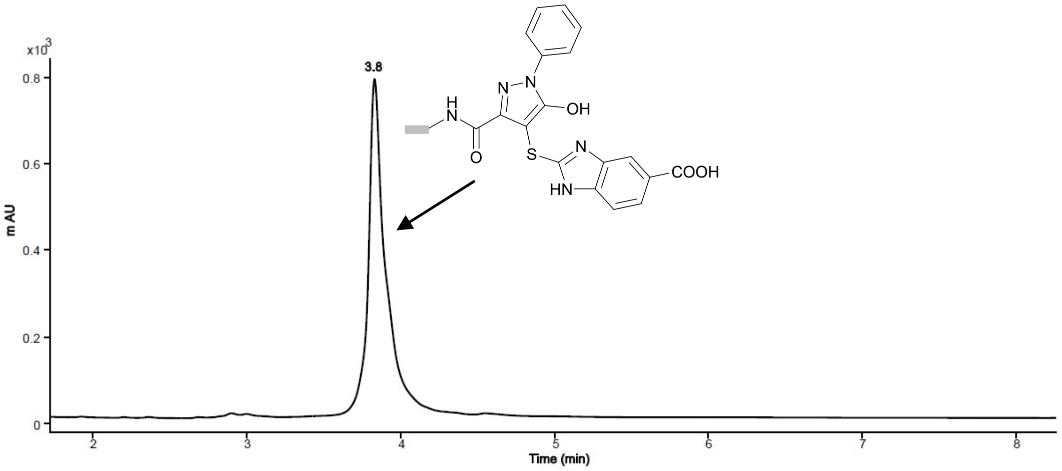


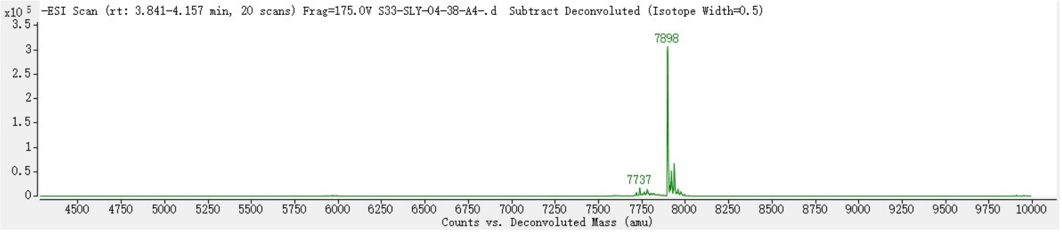

UPLC chromatograph and deconvoluted MS of **5v.**

**Conversion: 85%**

**Calculated Mass: 7898 Da; Found Mass: 7899 Da**

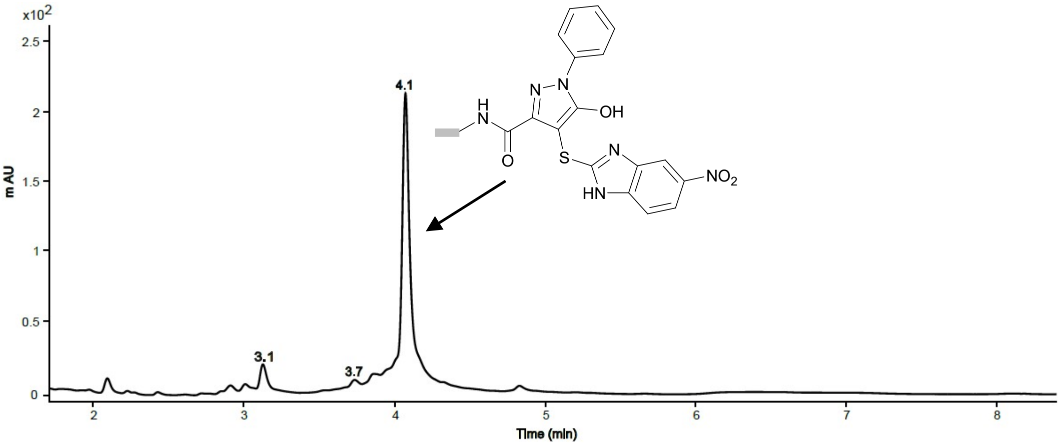


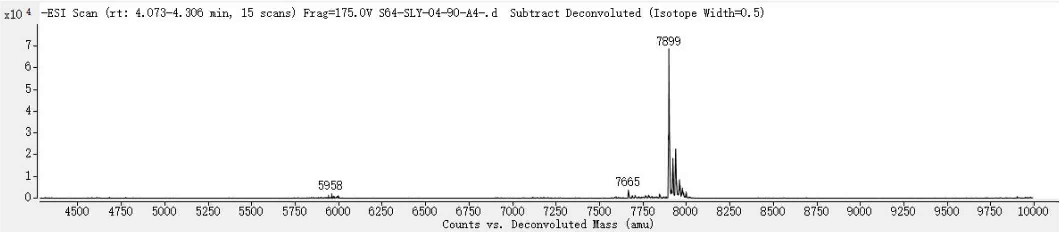


UPLC chromatograph and deconvoluted MS of **5w.**

**Conversion: 92%**

**Calculated Mass: 7888 Da; Found Mass: 7888 Da**

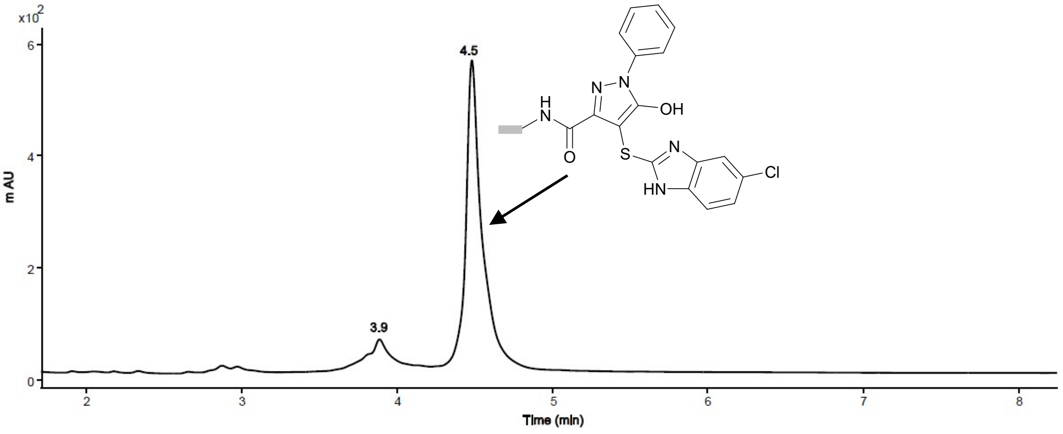


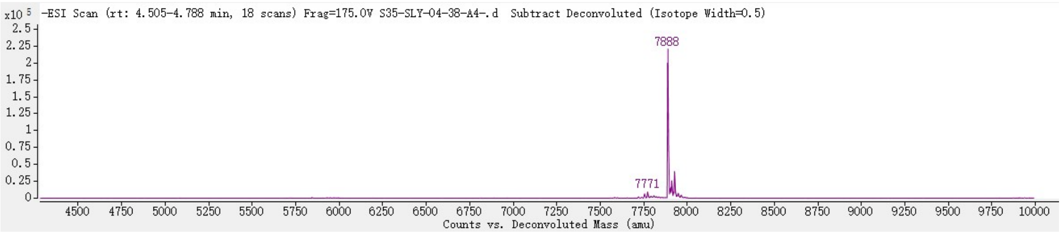

UPLC chromatograph and deconvoluted MS of **5x.**

**Conversion: 44%**

**Calculated Mass: 7827 Da; Found Mass: 7827 Da**

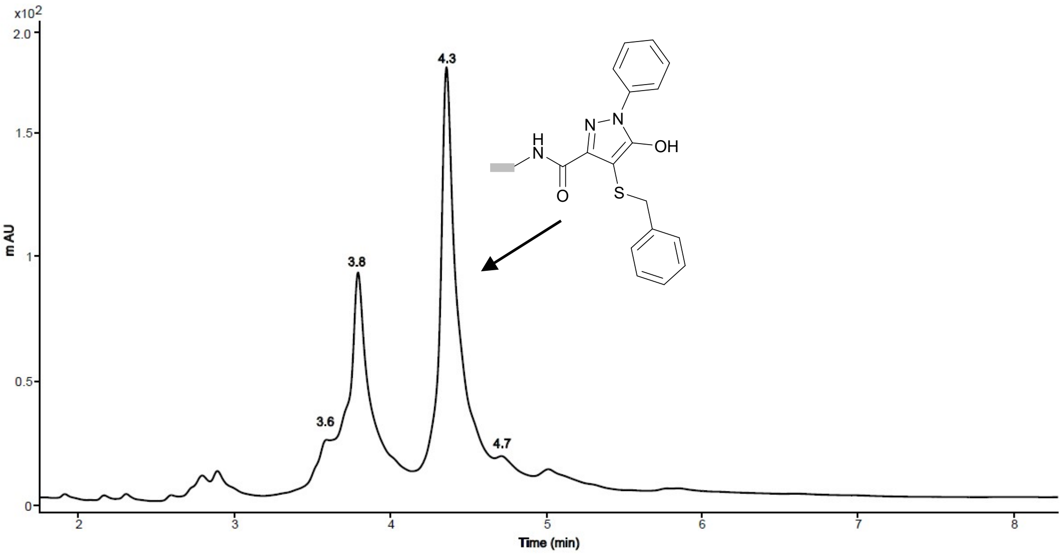


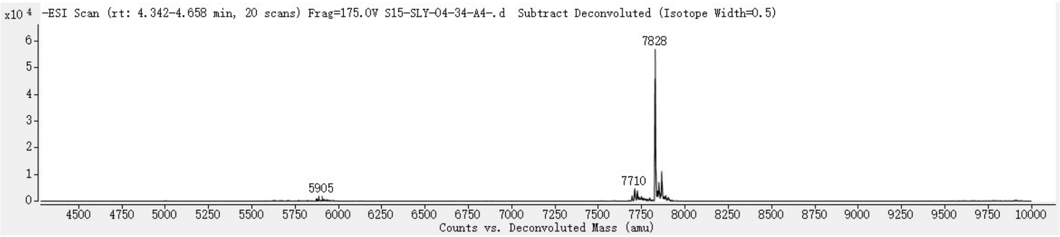


UPLC chromatograph and deconvoluted MS of **5z.**

**Conversion: 90%**

**Calculated Mass: 7818 Da; Found Mass: 7818 Da**

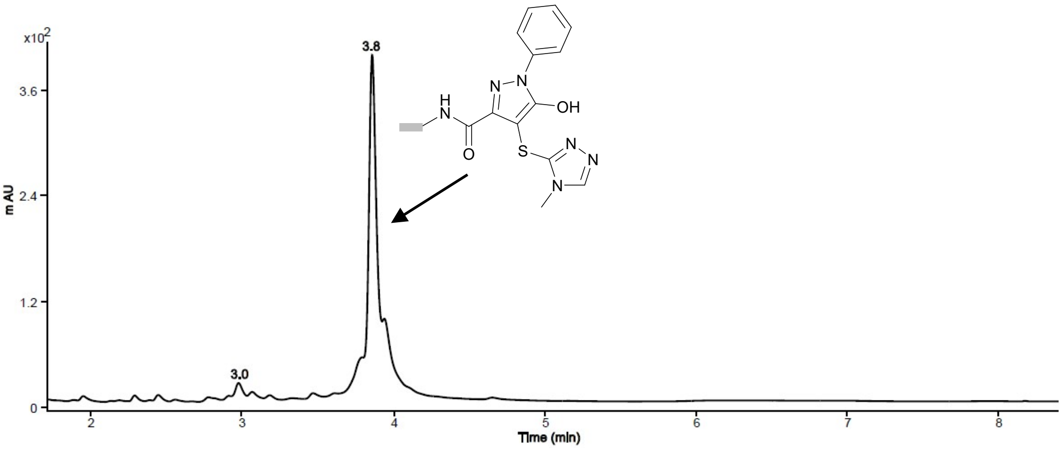


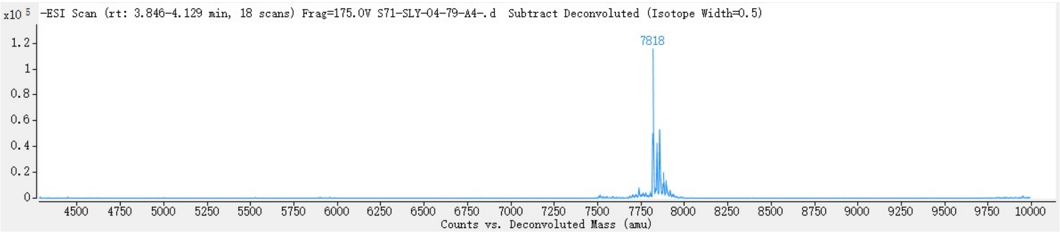

UPLC chromatograph and deconvoluted MS of **5aa.**

**Conversion: >95%**

**Calculated Mass: 7863 Da; Found Mass: 7863 Da**

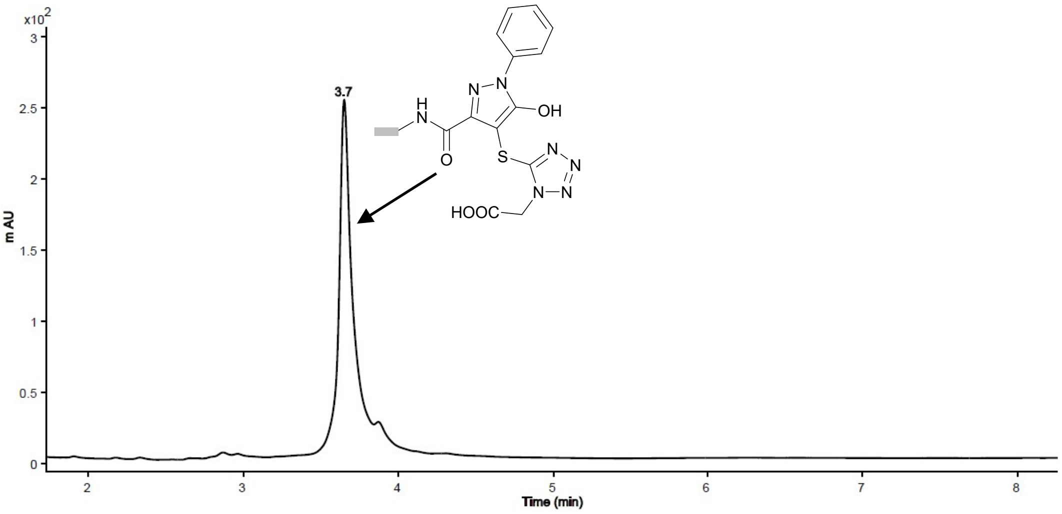


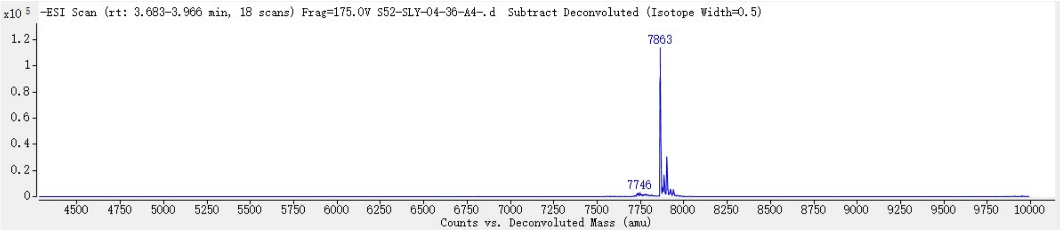


UPLC chromatograph and deconvoluted MS of **5ab.**

**Conversion: >95%**

**Calculated Mass: 7855 Da; Found Mass: 7856 Da**

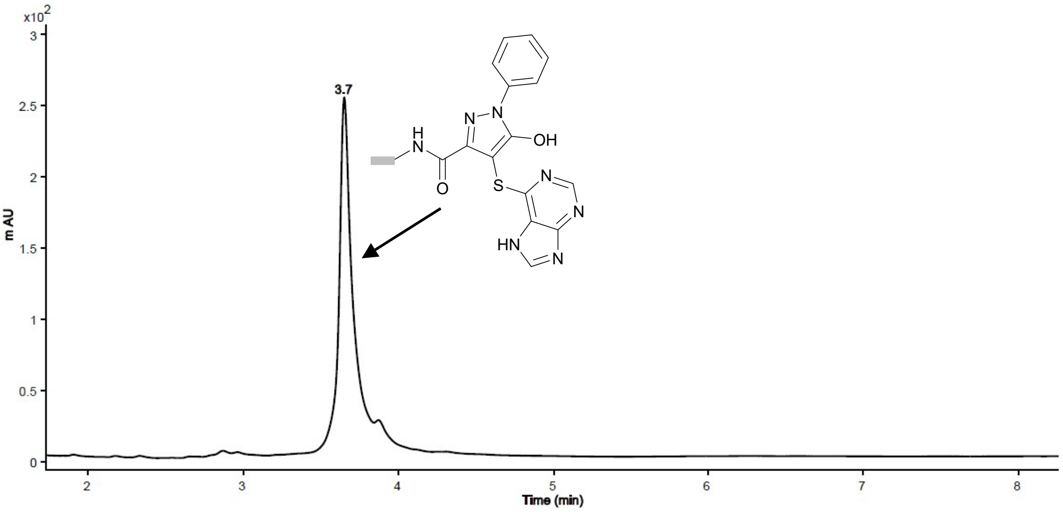


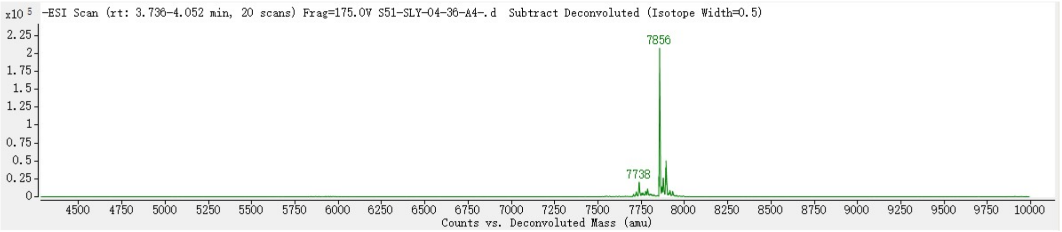

UPLC chromatograph and deconvoluted MS of **5ac.**

**Conversion: 79%**

**Calculated Mass: 7855 Da; Found Mass: 7856 Da**

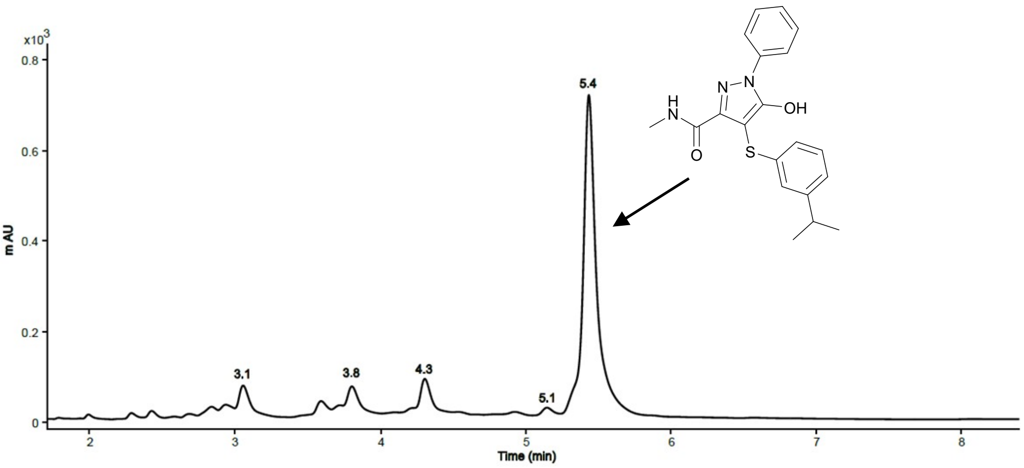


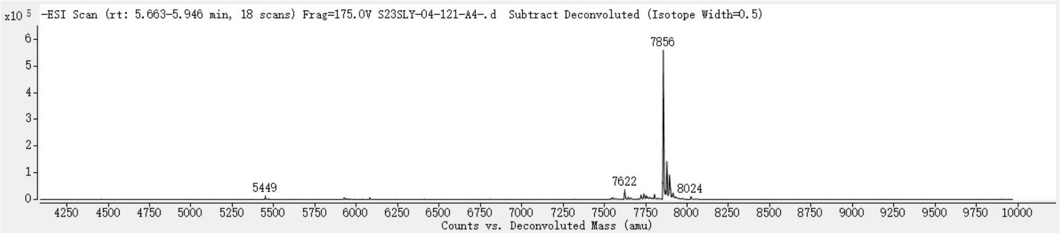


UPLC chromatograph and deconvoluted MS of **5ad.**

**Conversion: 73%**

**Calculated Mass: 7949 Da; Found Mass: 7951 Da**

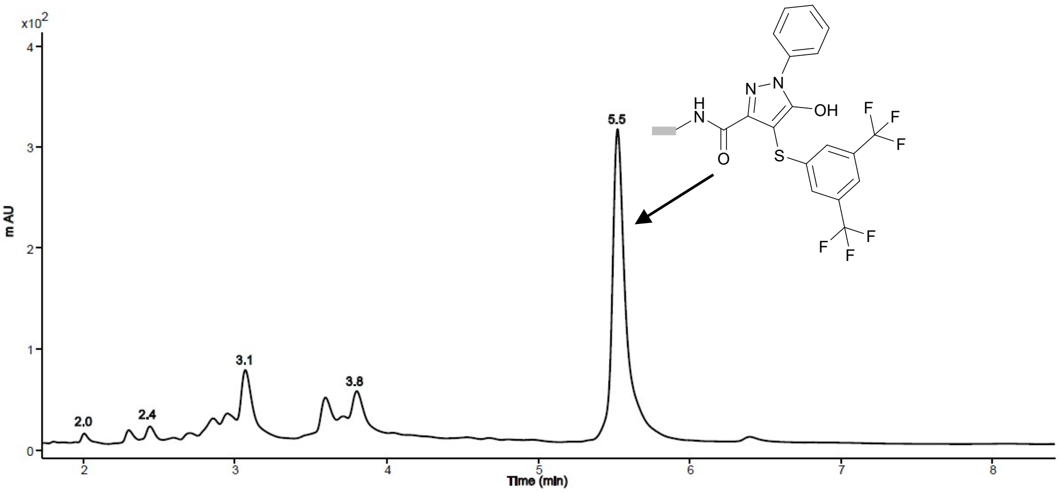


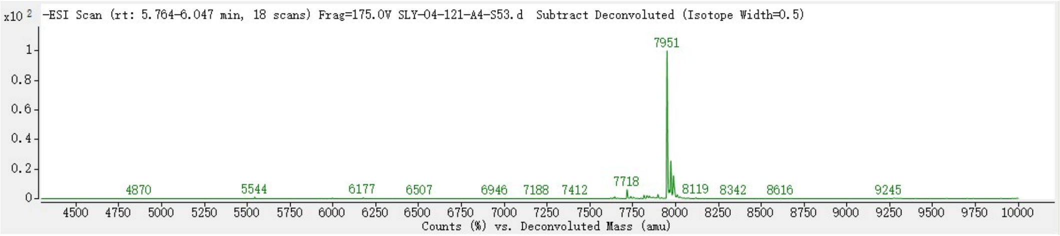

UPLC chromatograph and deconvoluted MS of **5ae.**

**Conversion: 78%**

**Calculated Mass: 7817 Da; Found Mass: 7817 Da**

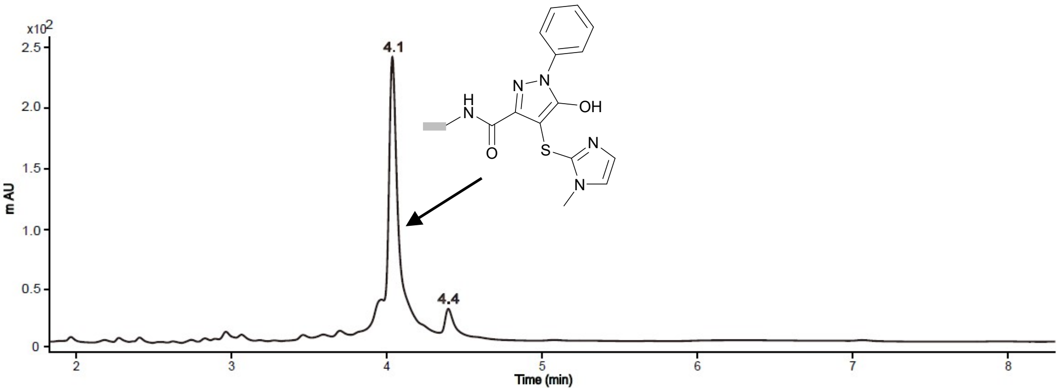


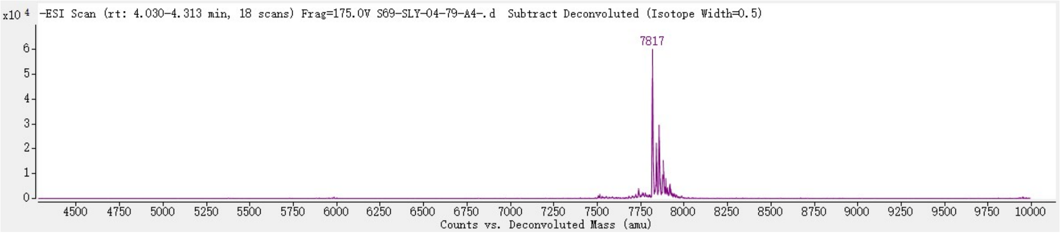


UPLC chromatograph and deconvoluted MS of **5af.**

**Conversion: >95%**

**Calculated Mass: 7897 Da; Found Mass: 7898 Da**

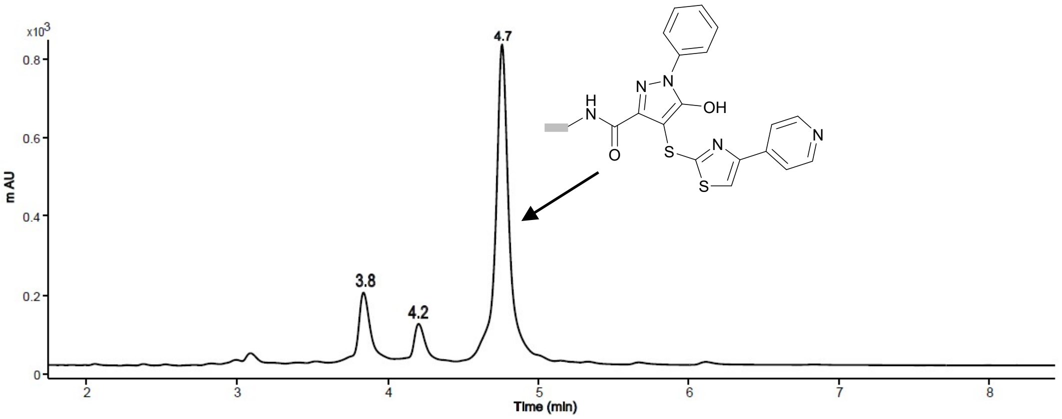


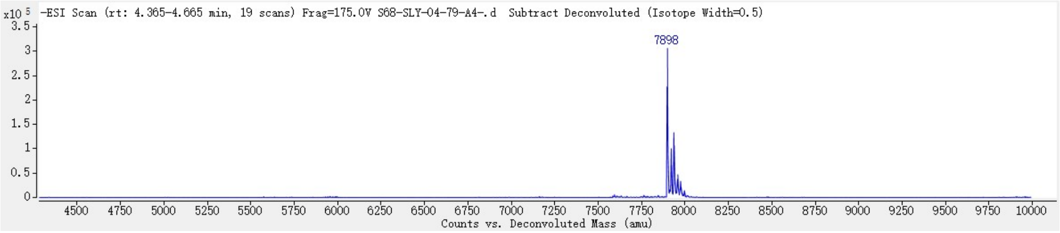

UPLC chromatograph and deconvoluted MS of **5ag.**

**Conversion: 81%**

**Calculated Mass: 7804 Da; Found Mass: 7804 Da**

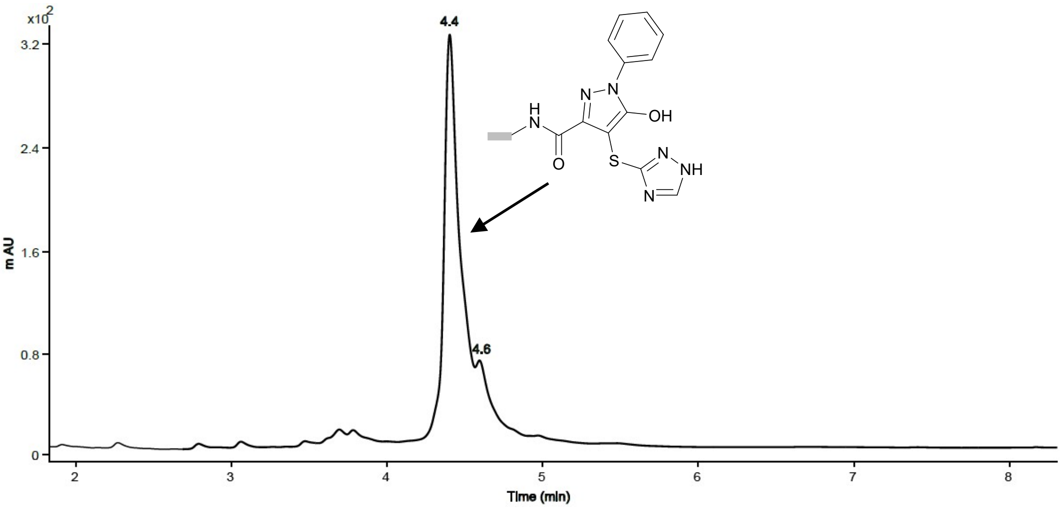


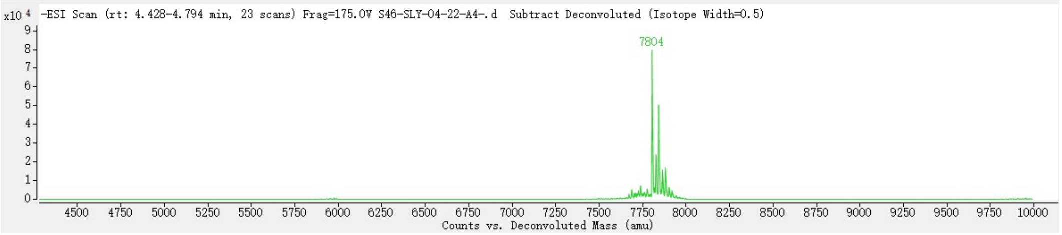


UPLC chromatograph and deconvoluted MS of **5ah.**

**Conversion: 86%**

**Calculated Mass: 7836 Da; Found Mass: 7837 Da**

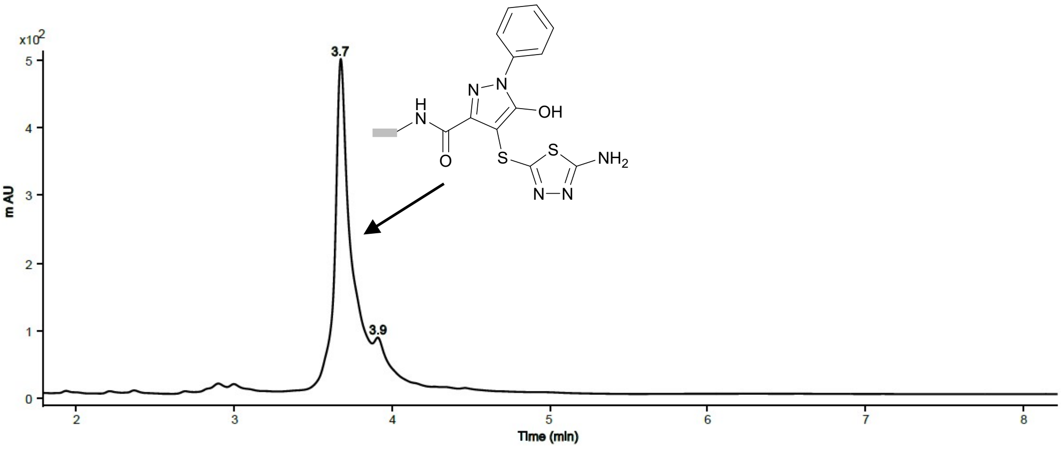


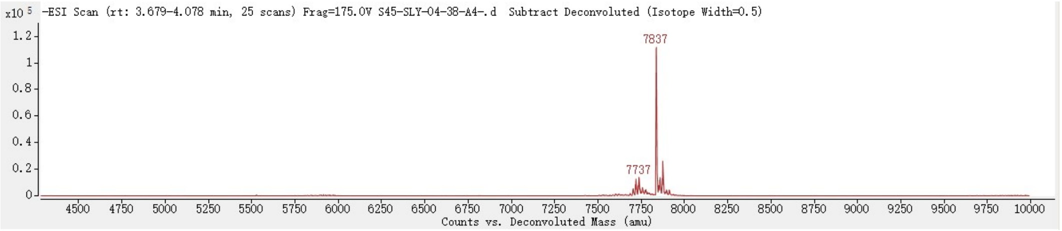

UPLC chromatograph and deconvoluted MS of **5ai.**

**Conversion: 93%**

**Calculated Mass: 7889 Da; Found Mass: 7889 Da**

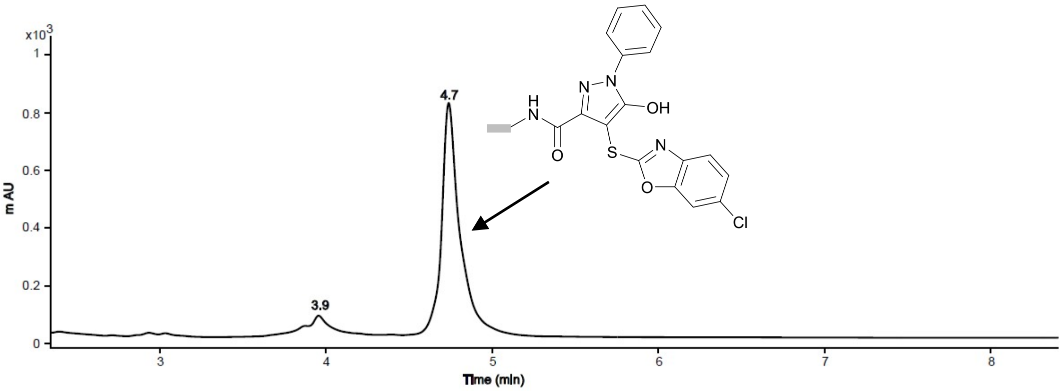


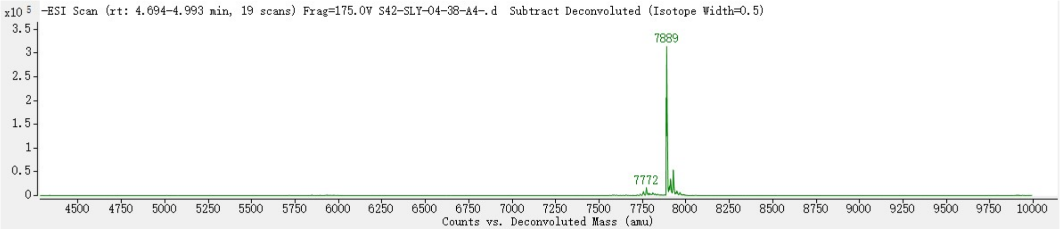


UPLC chromatograph and deconvoluted MS of **5aj.**

**Conversion: 92%**

**Calculated Mass: 7872 Da; Found Mass: 7873 Da**

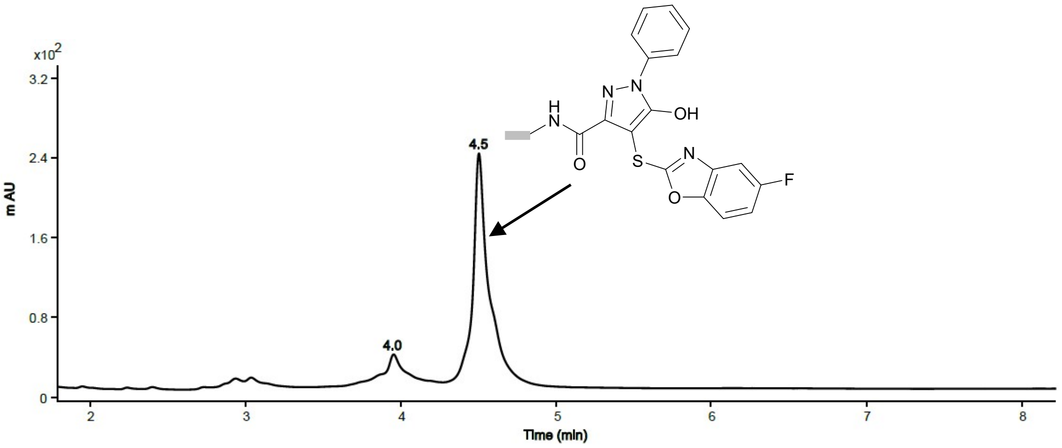


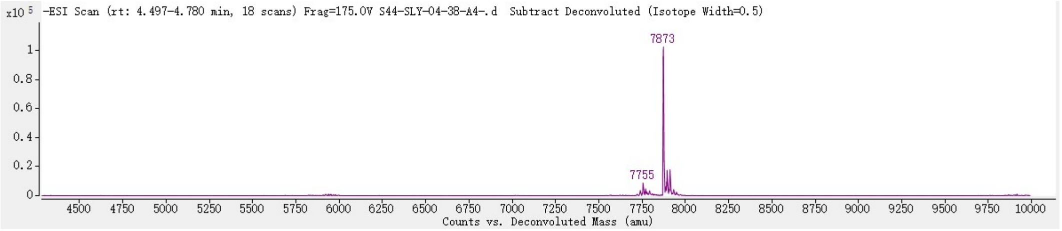

UPLC chromatograph and deconvoluted MS of **5ak.**

**Conversion: 83%**

**Calculated Mass: 7843 Da; Found Mass: 7843 Da**

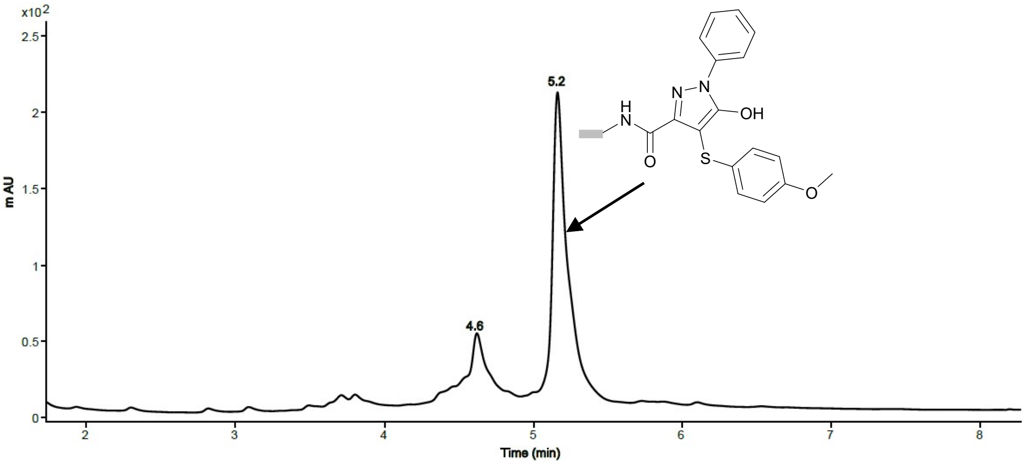


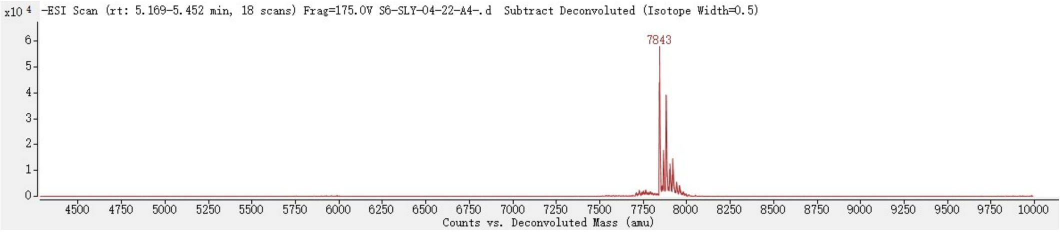


UPLC chromatograph and deconvoluted MS of **5al.**

**Conversion: 86%**

**Calculated Mass: 7871 Da; Found Mass: 7871 Da**

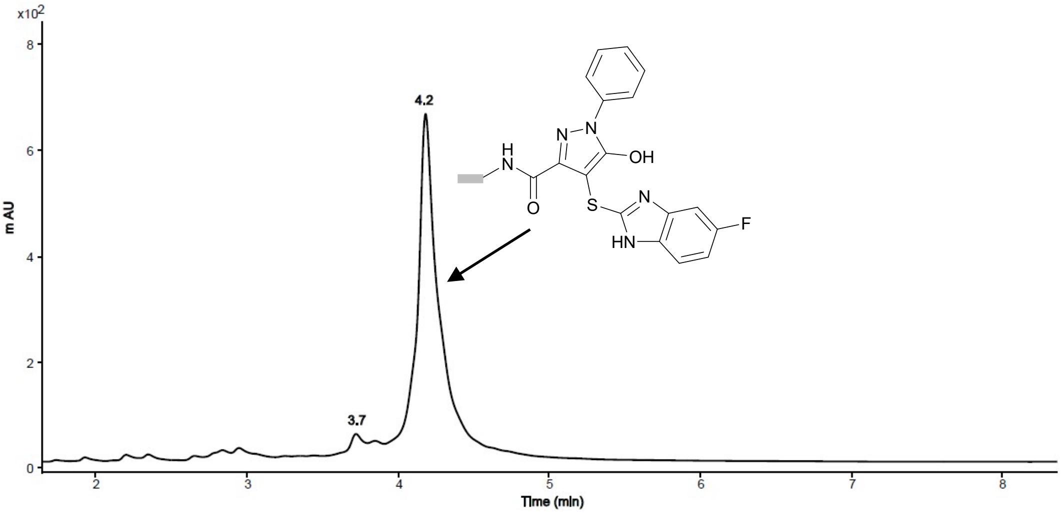


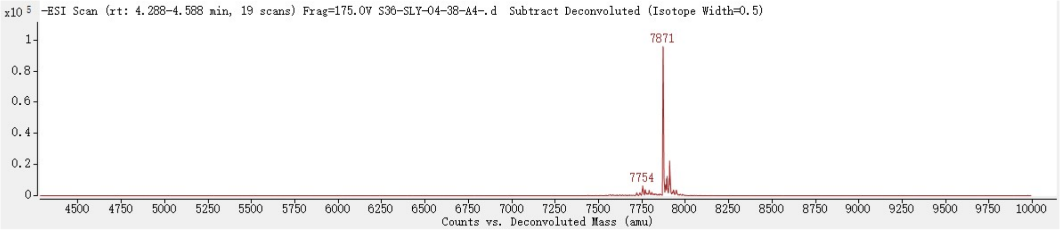

UPLC chromatograph and deconvoluted MS of **5am.**

**Conversion: >95%**

**Calculated Mass: 7829 Da; Found Mass: 7829 Da**

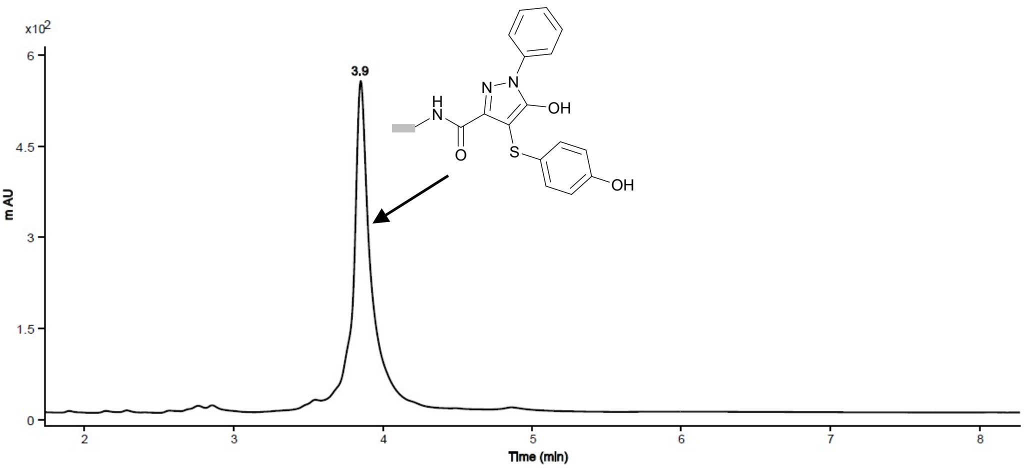


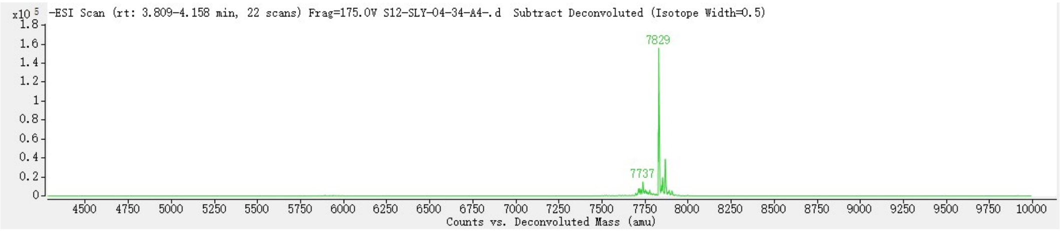


UPLC chromatograph and deconvoluted MS of **5an.**

**Conversion: 91%**

**Calculated Mass: 7831 Da; Found Mass: 7831 Da**

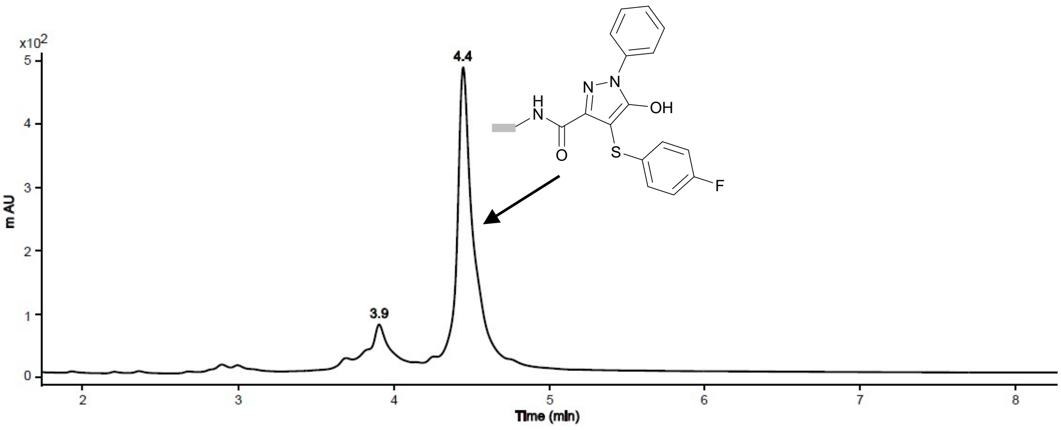


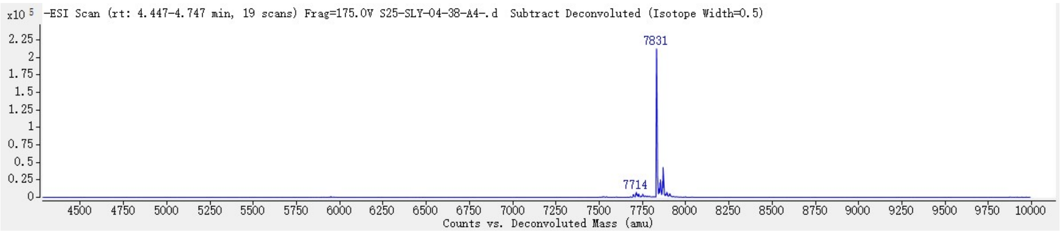

UPLC chromatograph and deconvoluted MS of **5ao.**

**Conversion: >95%**

**Calculated Mass: 7845 Da; Found Mass: 7845 Da**

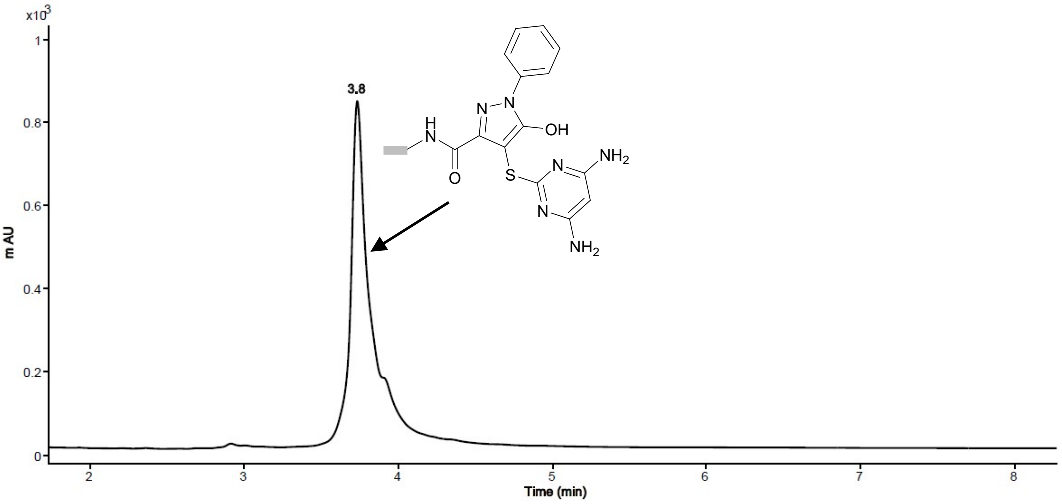


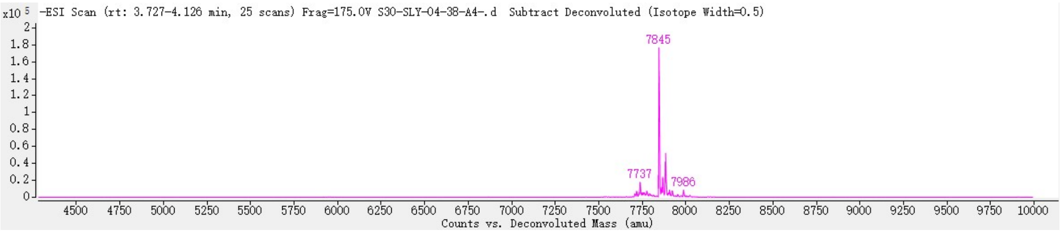


UPLC chromatograph and deconvoluted MS of **5ap.**

**Conversion: 84%**

**Calculated Mass: 7819 Da; Found Mass: 7819 Da**

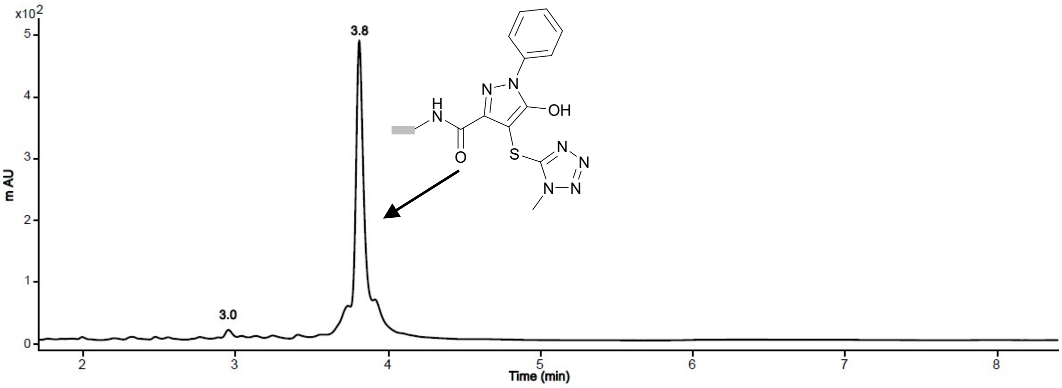


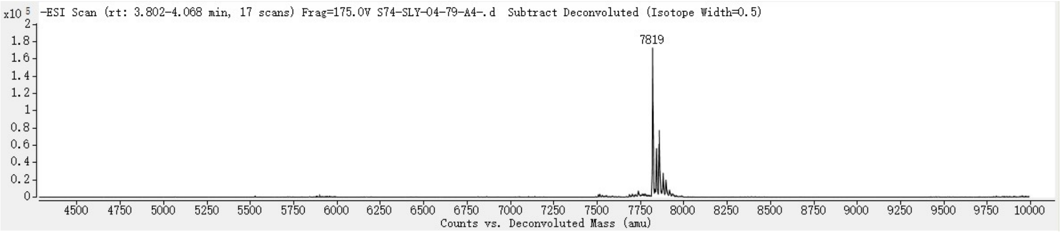

UPLC chromatograph and deconvoluted MS of **5aq.**

**Conversion: 77%**

**Calculated Mass: 7911 Da; Found Mass: 7912 Da**

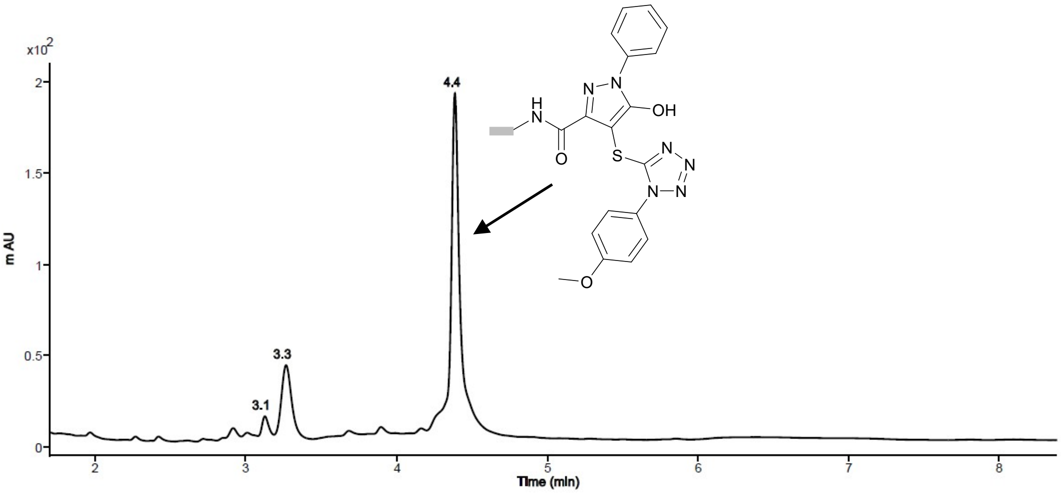


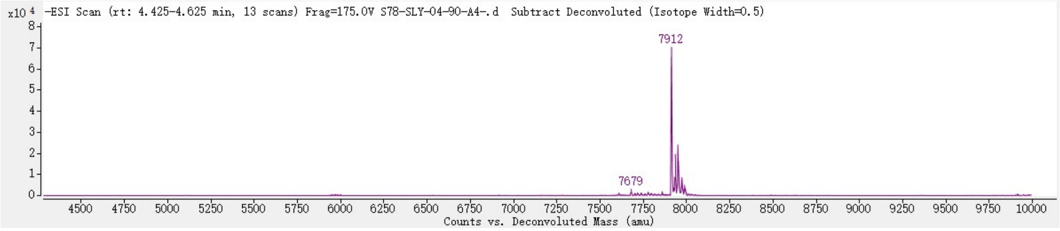


UPLC chromatograph and deconvoluted MS of **5ar.**

**Conversion: >95%**

**Calculated Mass: 7821 Da; Found Mass: 7822 Da**

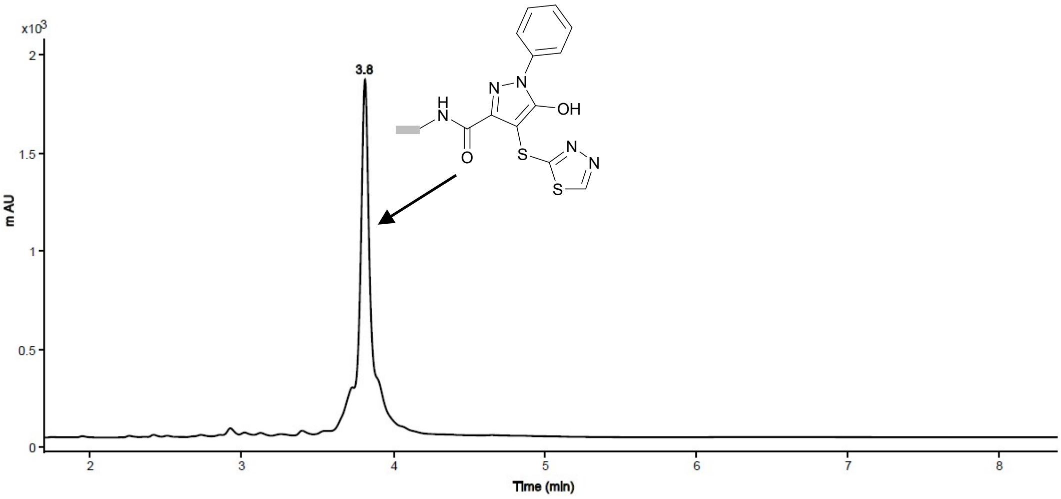


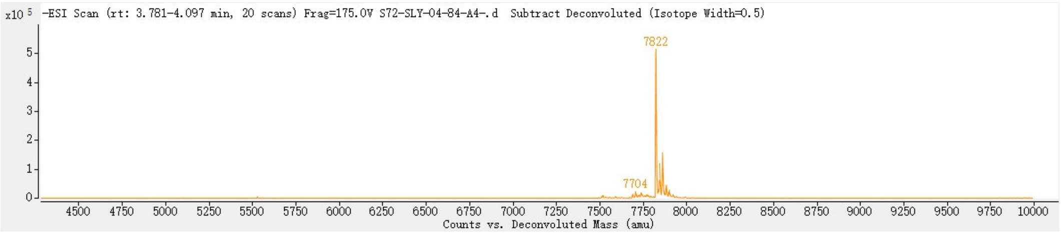

UPLC chromatograph and deconvoluted MS of **5as.**

**Conversion: 77%**

**Calculated Mass: 7869 Da; Found Mass: 7870 Da**

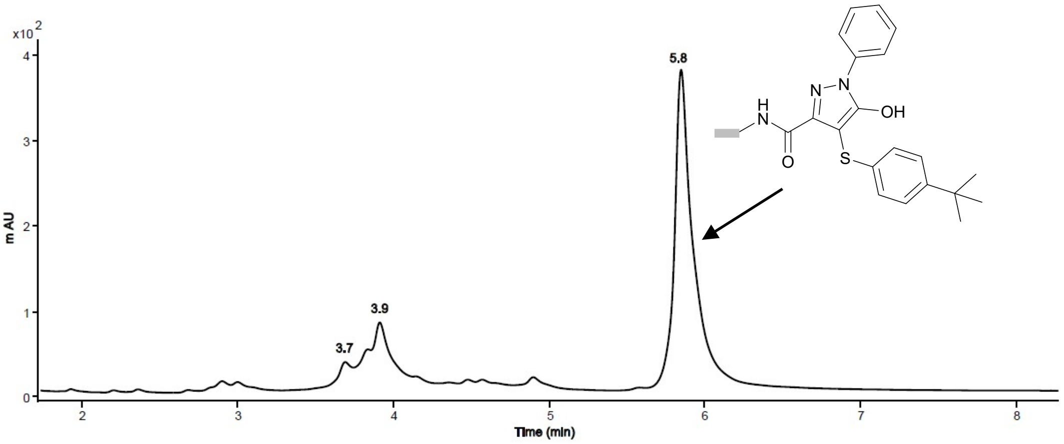


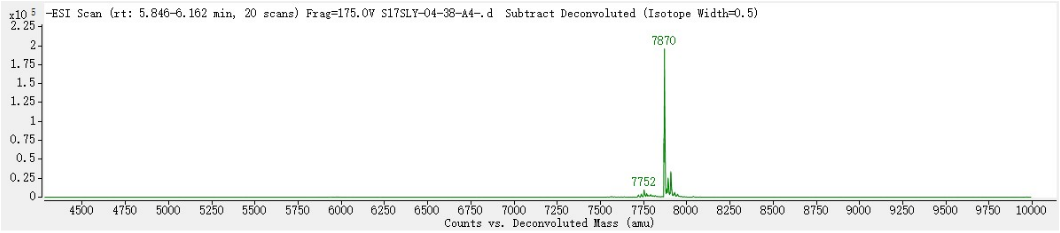


UPLC chromatograph and deconvoluted MS of **5au.**

**Conversion: 91%**

**Calculated Mass: 7870 Da; Found Mass: 7871 Da**

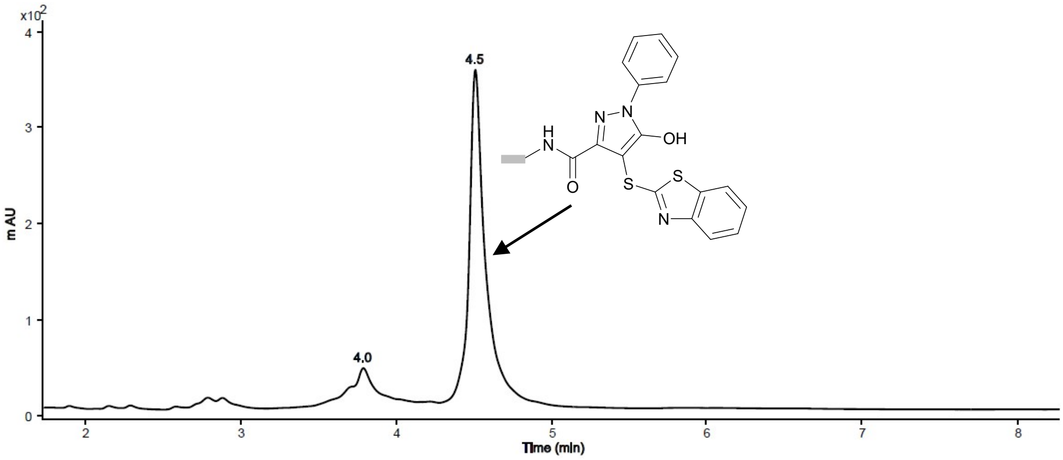


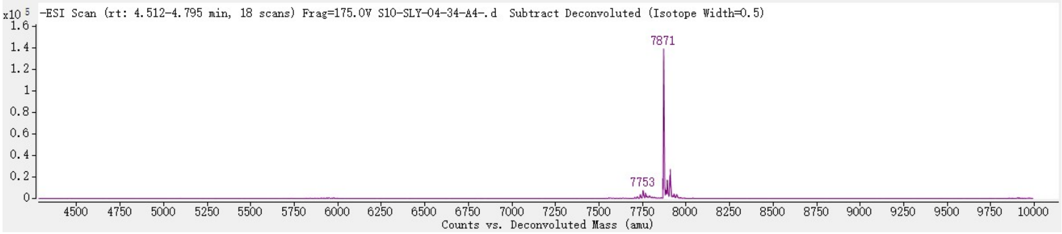

UPLC chromatograph and deconvoluted MS of **5av.**

**Conversion: 94%**

**Calculated Mass: 7876 Da; Found Mass: 7877 Da**

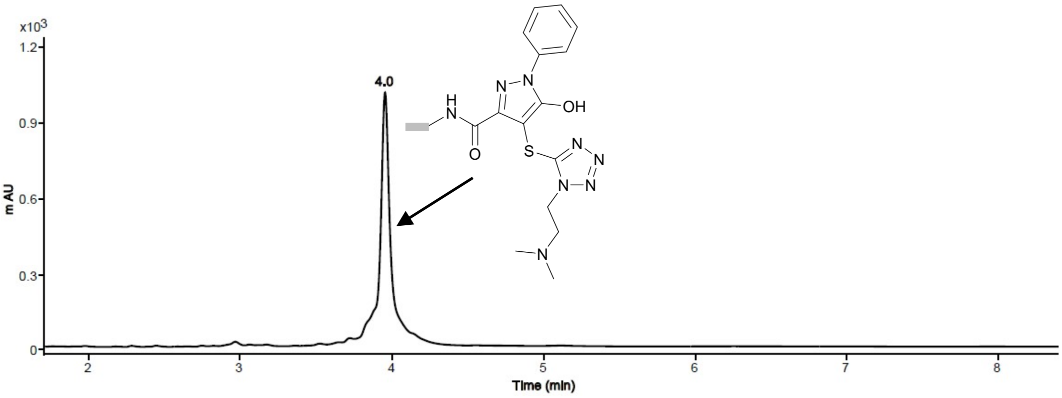


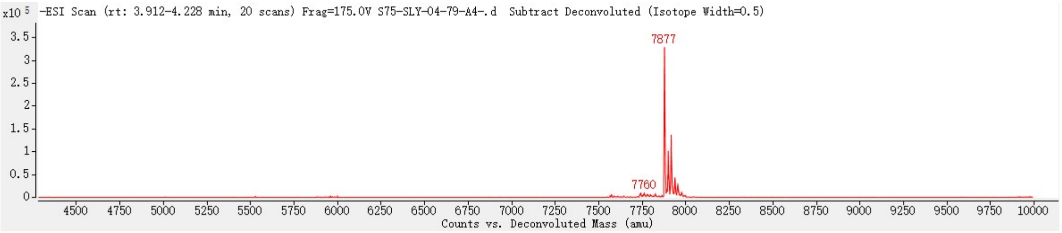


UPLC chromatograph and deconvoluted MS of **5aw.**

**Conversion: 80%**

**Calculated Mass: 7841 Da; Found Mass: 7842 Da**

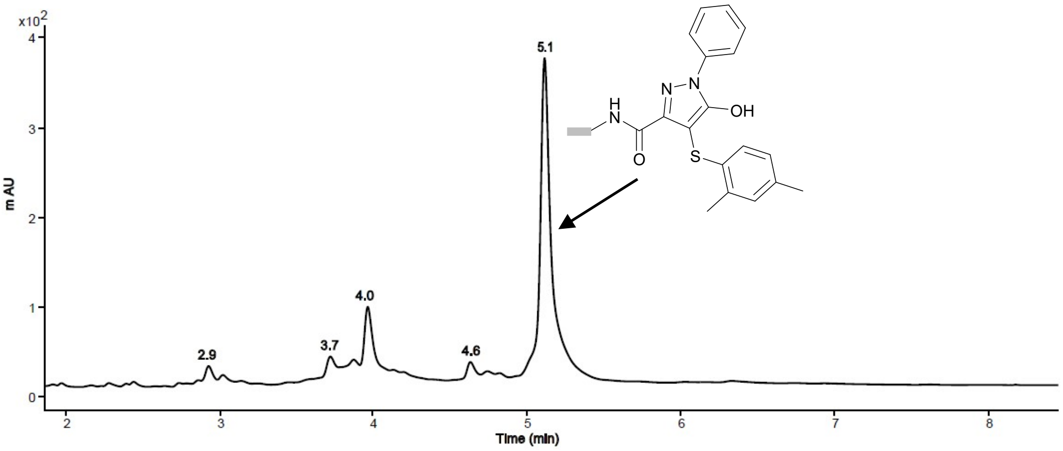


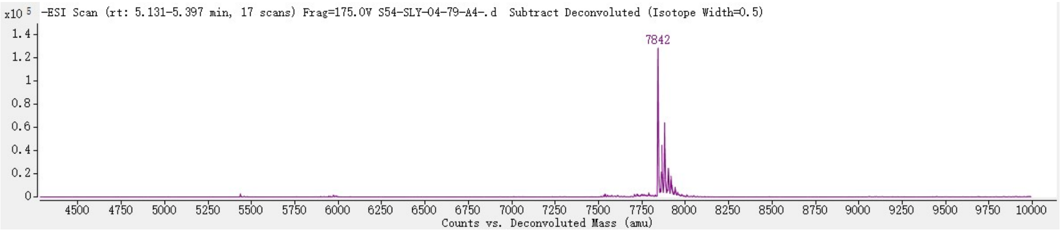

UPLC chromatograph and deconvoluted MS of **5bb.**

**Conversion: 93%**

**Calculated Mass: 7938 Da; Found Mass: 7939 Da**

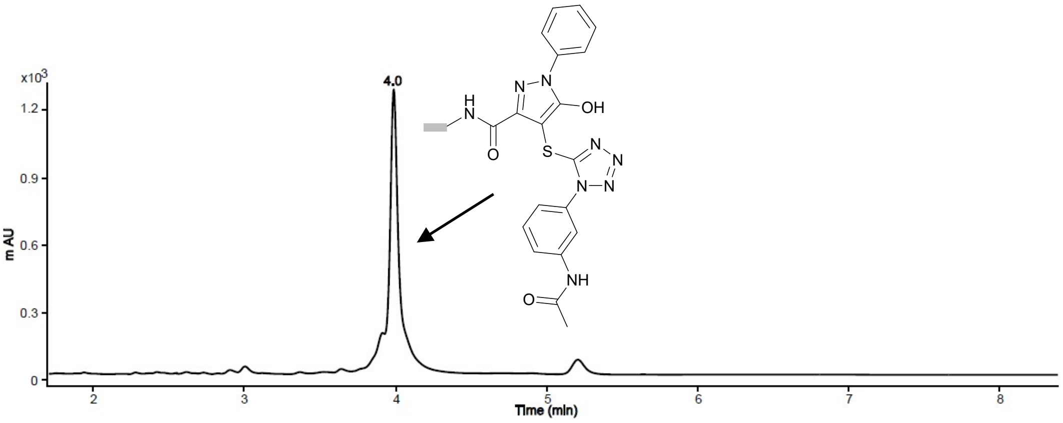


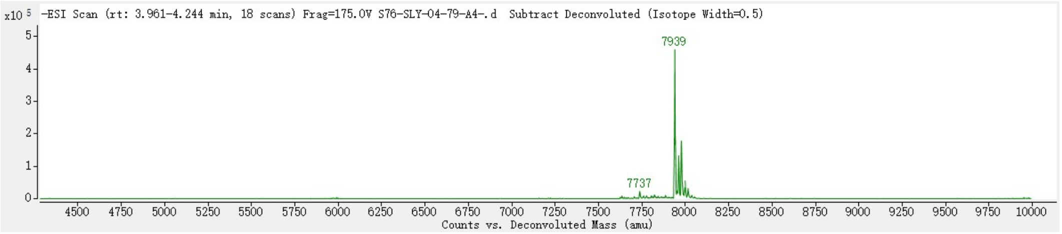


UPLC chromatograph and deconvoluted MS of **5bc.**

**Conversion: 70%**

**Calculated Mass: 7828 Da; Found Mass: 7828 Da**

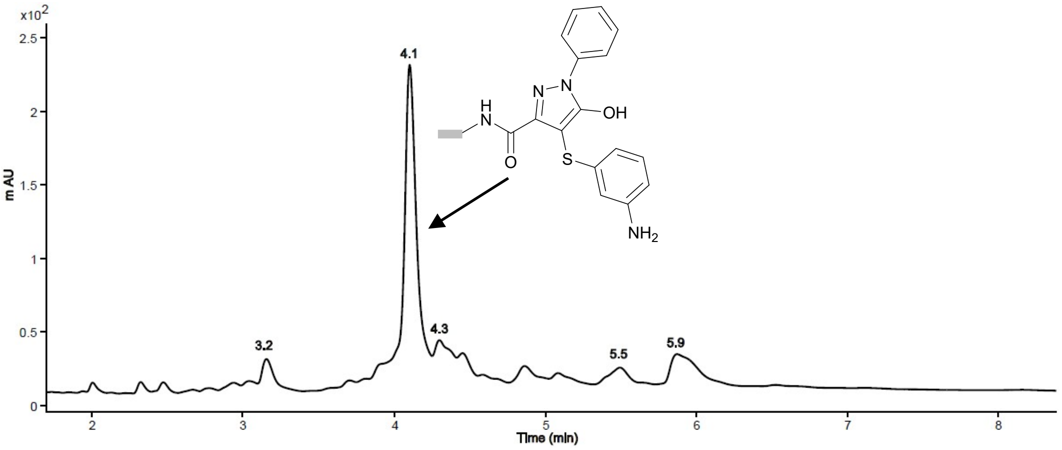


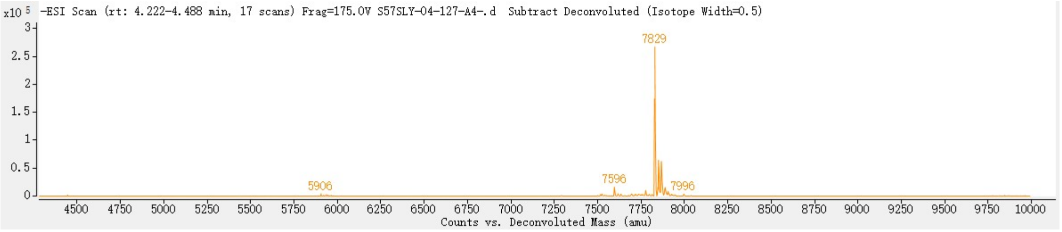

UPLC chromatograph and deconvoluted MS of **5bd.**

**Conversion: 55%**

**Calculated Mass: 7881 Da; Found Mass: 7881 Da**

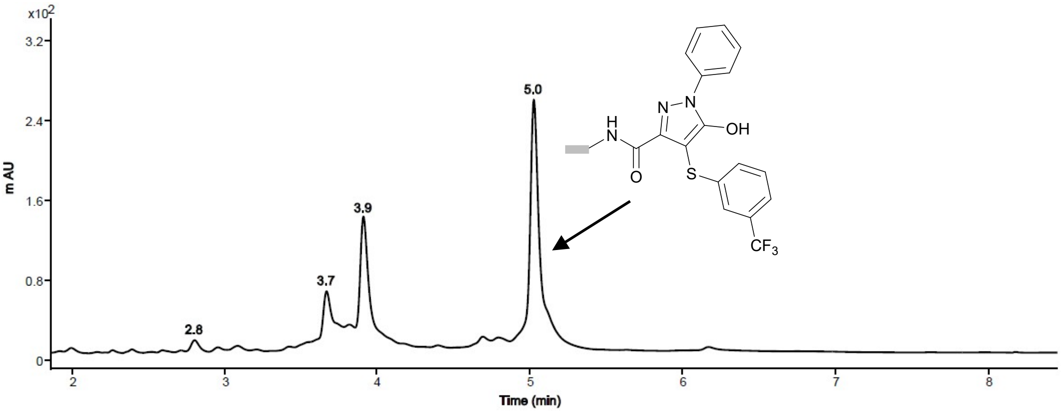


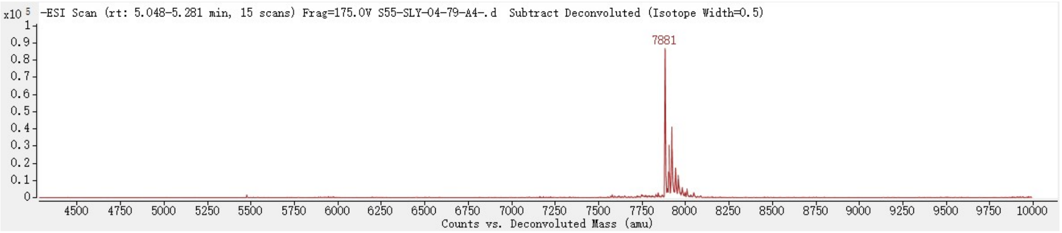


UPLC chromatograph and deconvoluted MS of **5be.**

**Conversion: 83%**

**Calculated Mass: 7831 Da; Found Mass: 7832 Da**

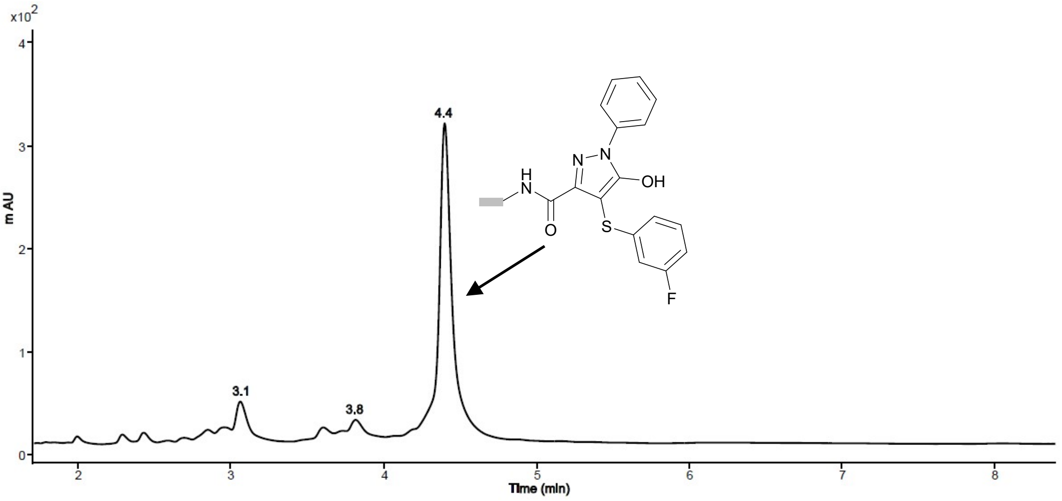


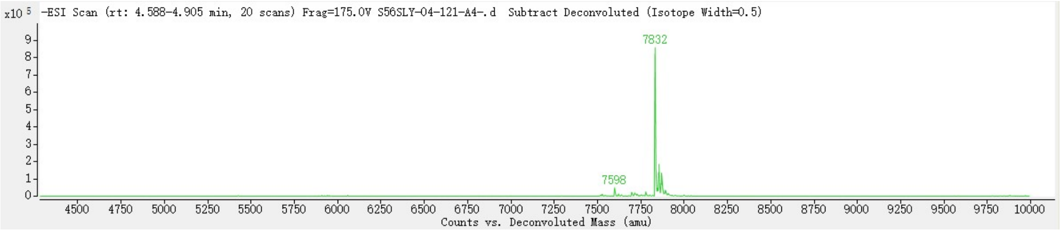


UPLC chromatograph and deconvoluted MS of **5bf.**

**Conversion: 84%**

**Calculated Mass: 7849 Da; Found Mass: 7850 Da**

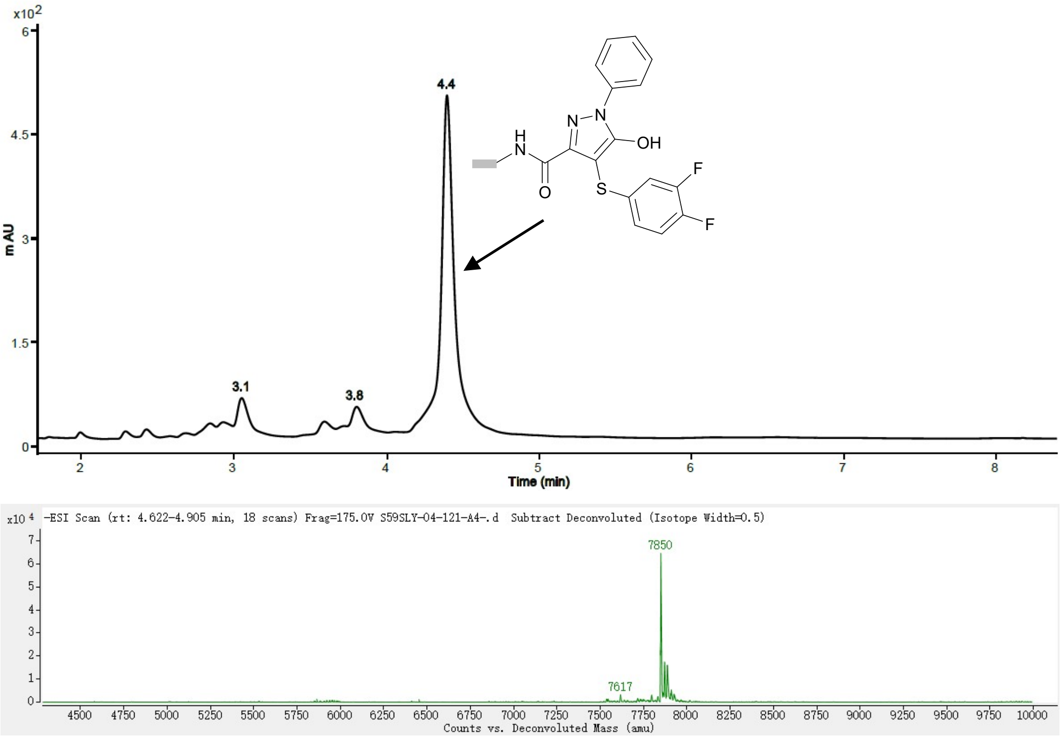


UPLC chromatograph and deconvoluted MS of **5bg.**

**Conversion: 85%**

**Calculated Mass: 7847 Da; Found Mass: 7848 Da**

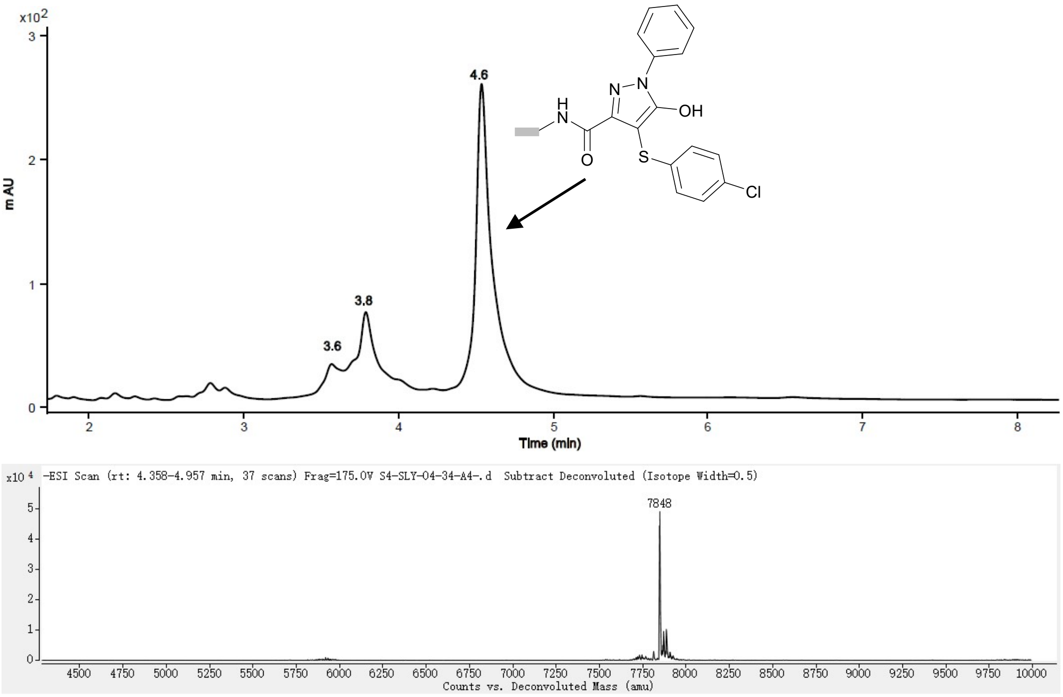


UPLC chromatograph and deconvoluted MS of **5bh.**

**Conversion: 59%**
**Calculated Mass: 7848 Da; Found Mass: 7849 Da**

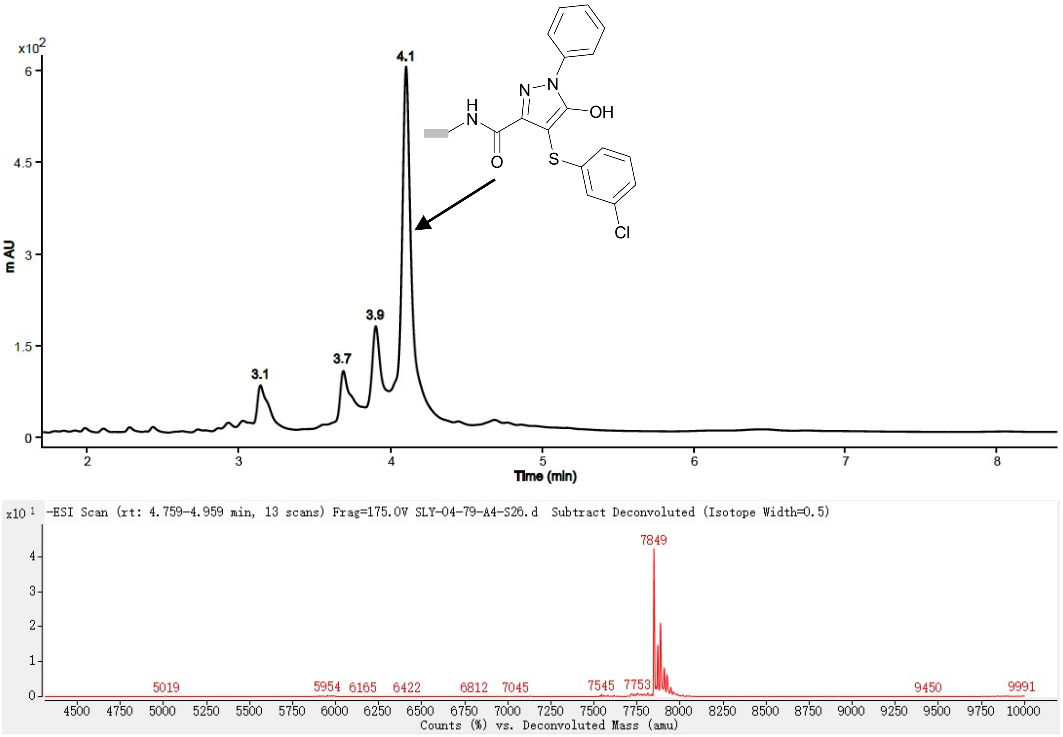


UPLC chromatograph and deconvoluted MS of **5bi.**

**Conversion: 75%**
**Calculated Mass: 7882 Da; Found Mass: 7883 Da**

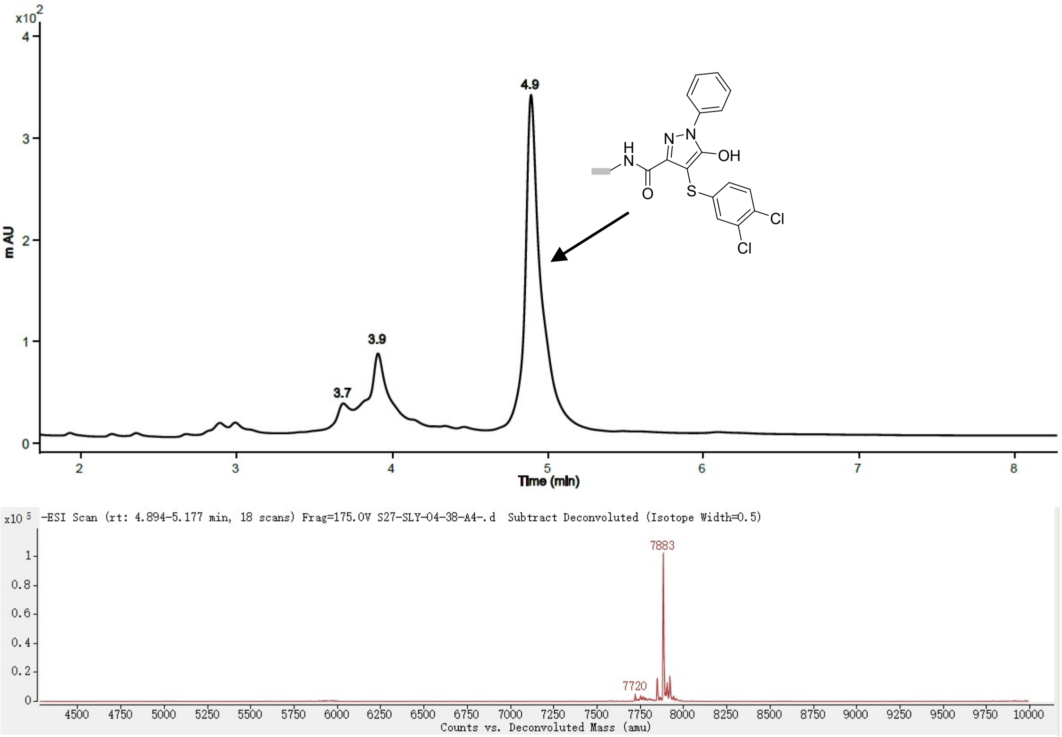


UPLC chromatograph and deconvoluted MS of **5bk.**

**Conversion: 81%**
**Calculated Mass: 7871 Da; Found Mass: 7872 Da**

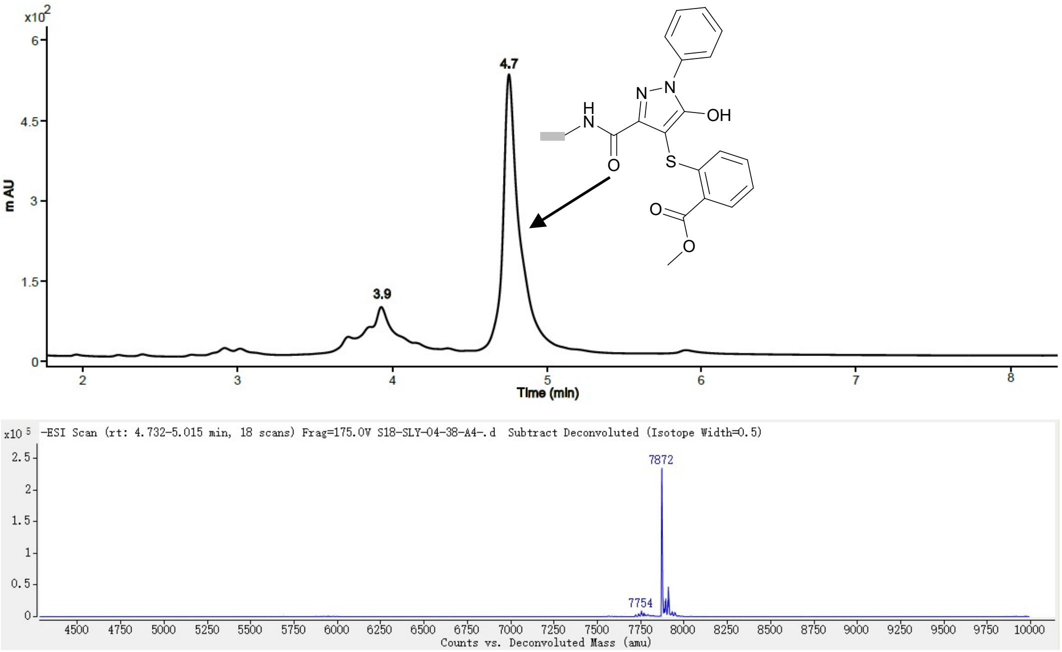


UPLC chromatograph and deconvoluted MS of **5bl.**
**Conversion: 67%**
**Calculated Mass: 7871 Da; Found Mass: 7871 Da**

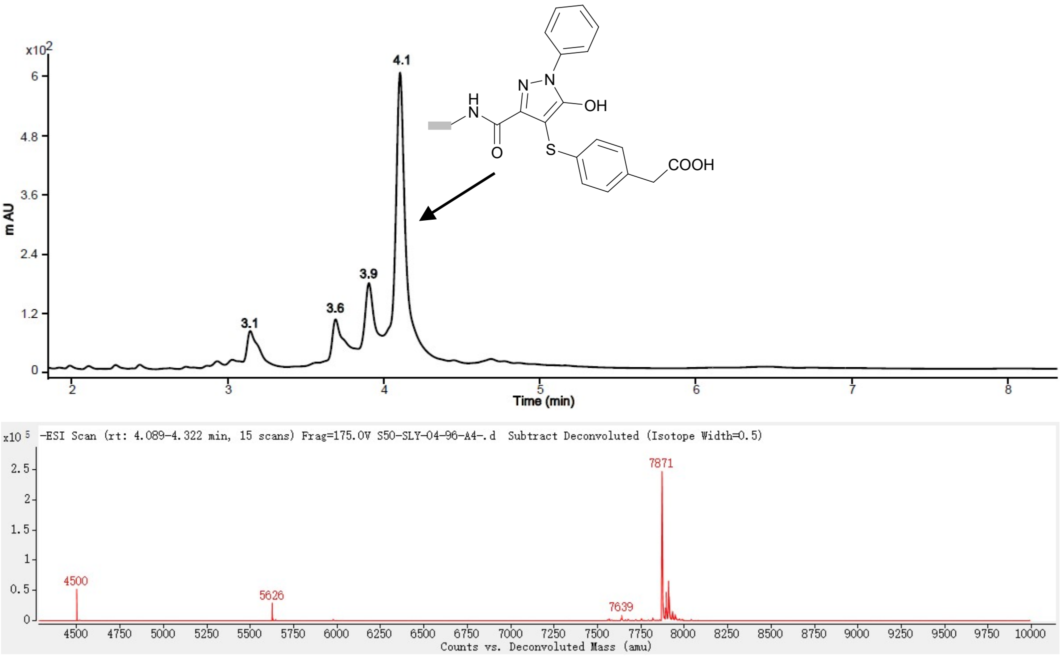


UPLC chromatograph and deconvoluted MS of **5bm.**

**Conversion: 92%**

**Calculated Mass: 7863 Da; Found Mass: 7863 Da**

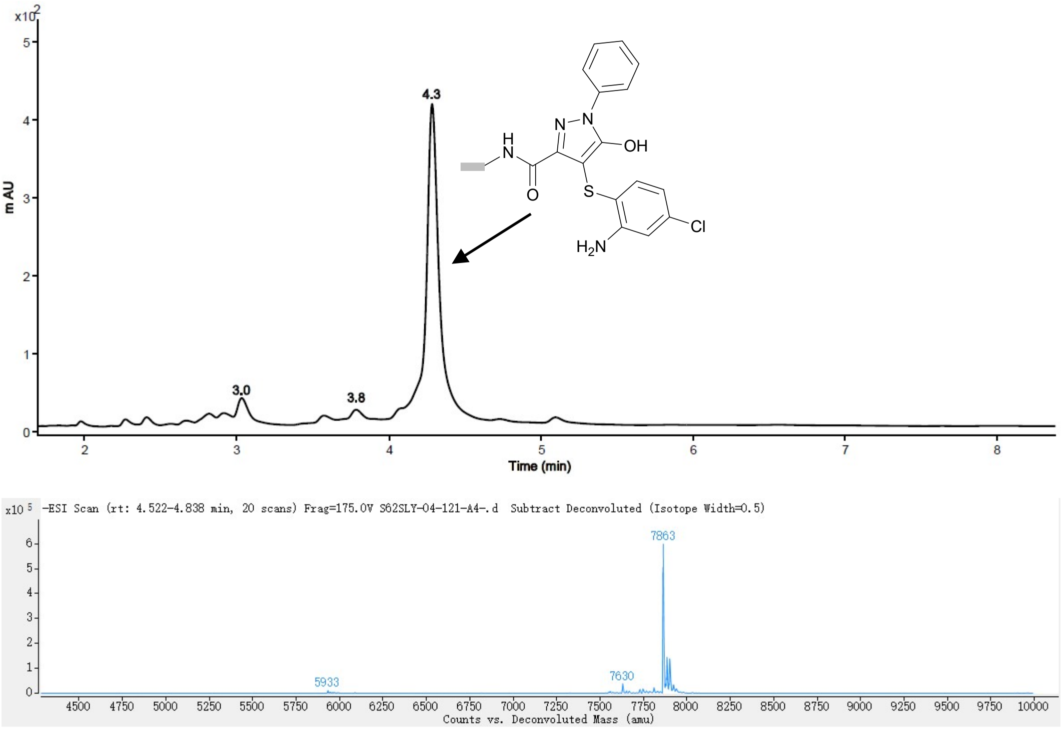


UPLC chromatograph and deconvoluted MS of **5bp.**

**Conversion: 96%**

**Calculated Mass: 7867 Da; Found Mass: 7868 Da**

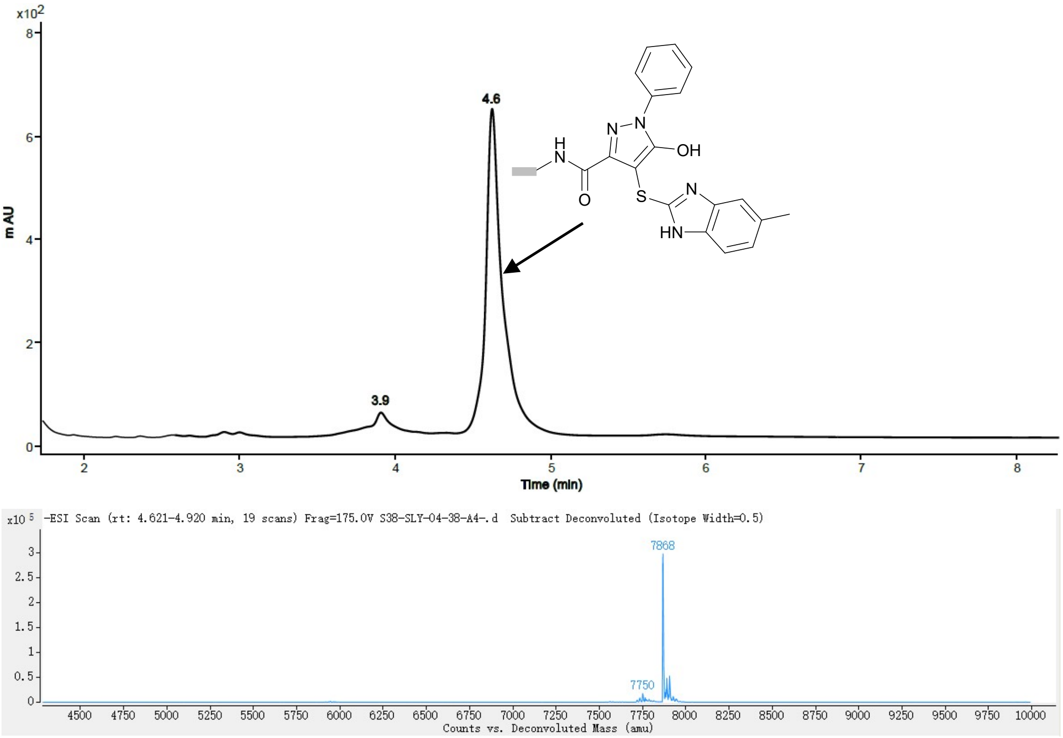


UPLC chromatograph and deconvoluted MS of **5br.**

**Conversion: 77%**
**Calculated Mass: 7843 Da; Found Mass: 7844 Da**

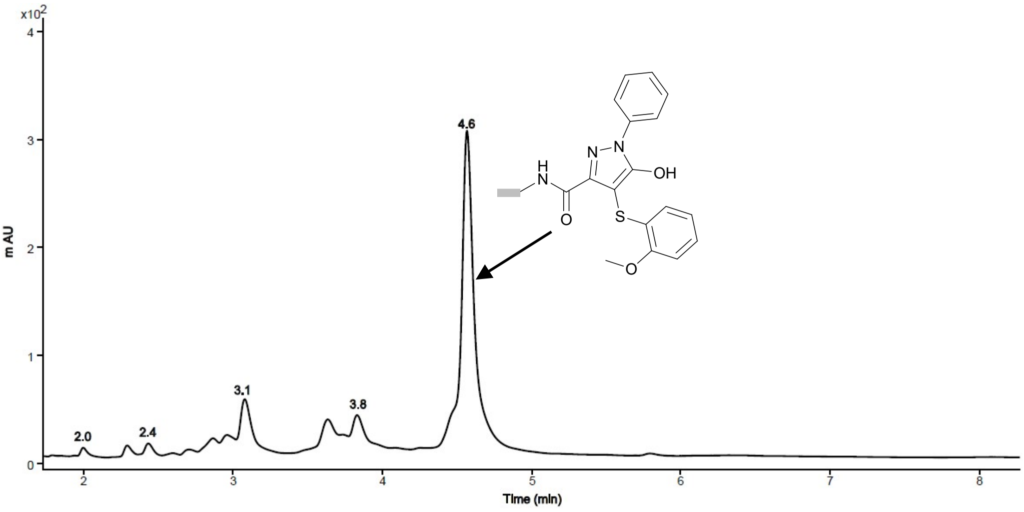


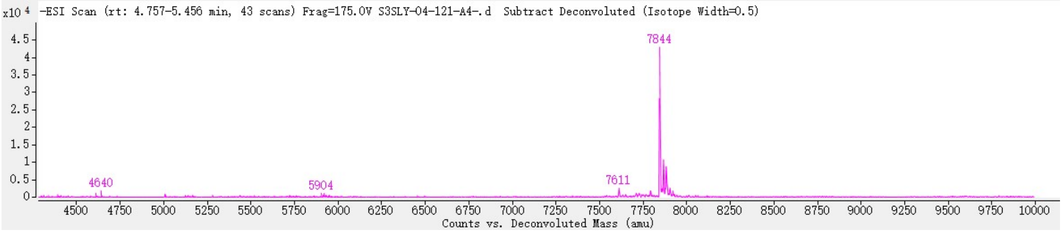


UPLC chromatograph and deconvoluted MS of **5bs.**
**Conversion: 86%**
**Calculated Mass: 7897 Da; Found Mass: 7897 Da**

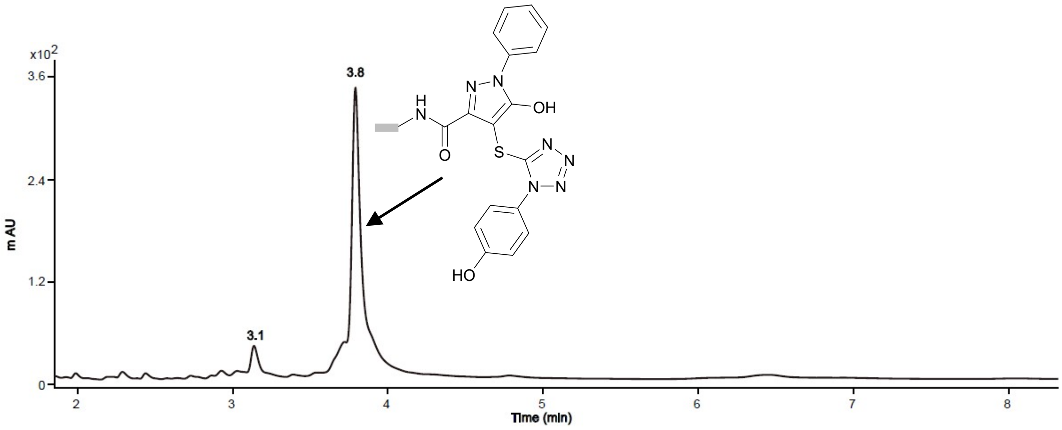


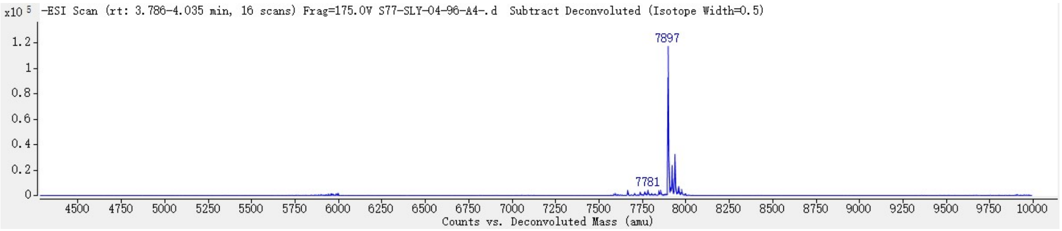


UPLC chromatograph and deconvoluted MS of **5bt.**

**Conversion: 78%**
**Calculated Mass: 7892 Da; Found Mass: 7893 Da**

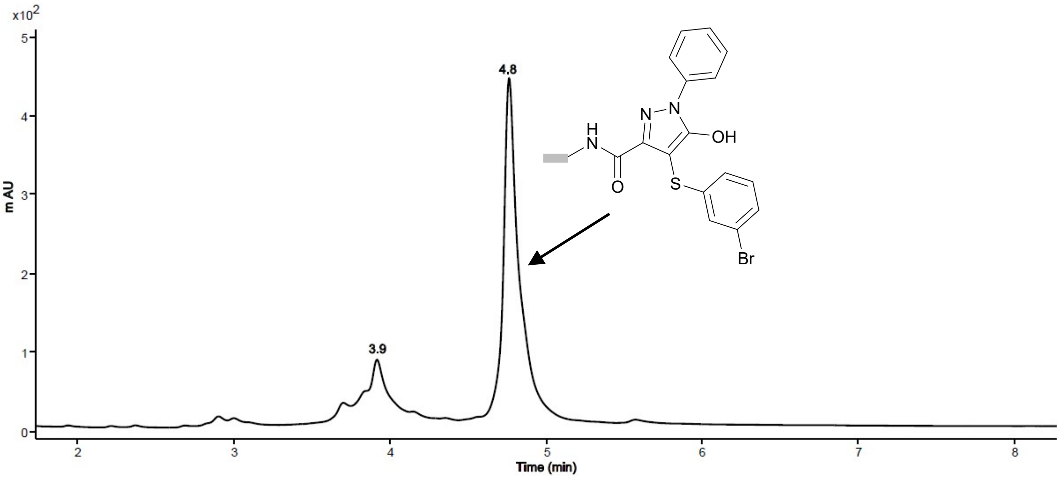


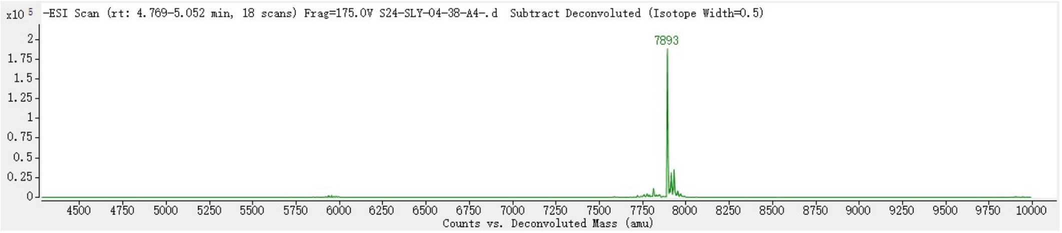


UPLC chromatograph and deconvoluted MS of **5bu.**
**Conversion: 94%**
**Calculated Mass: 7827 Da; Found Mass: 7829 Da**

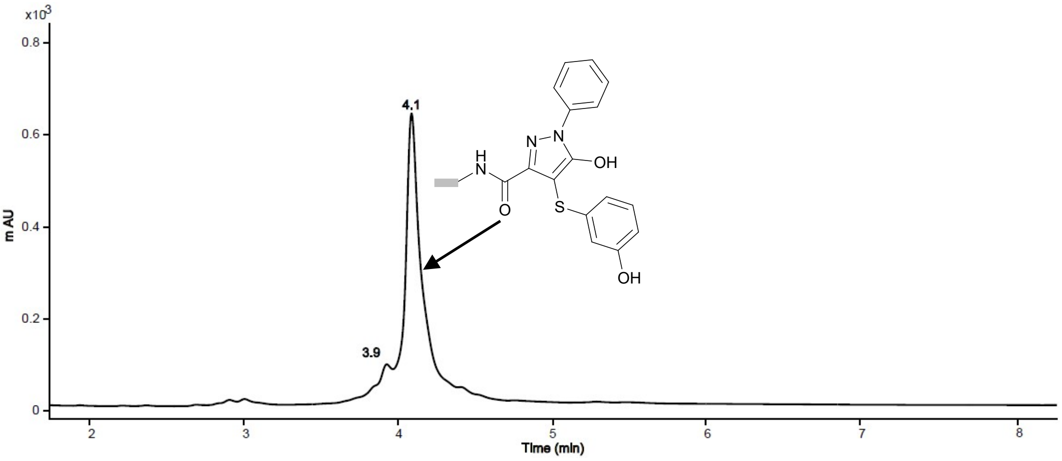


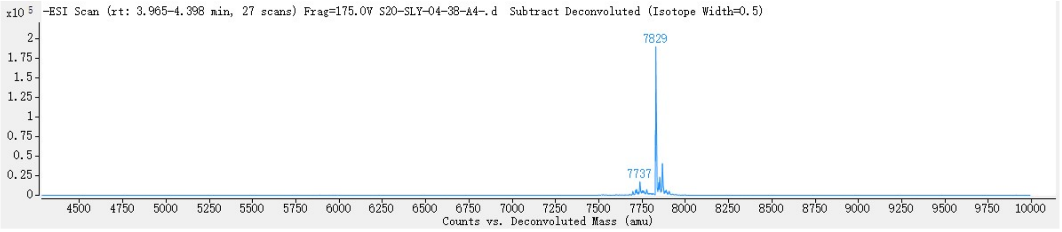

UPLC chromatograph and deconvoluted MS of **5bv.**

**Conversion: 82%**

**Calculated Mass: 7853 Da; Found Mass: 7853 Da**

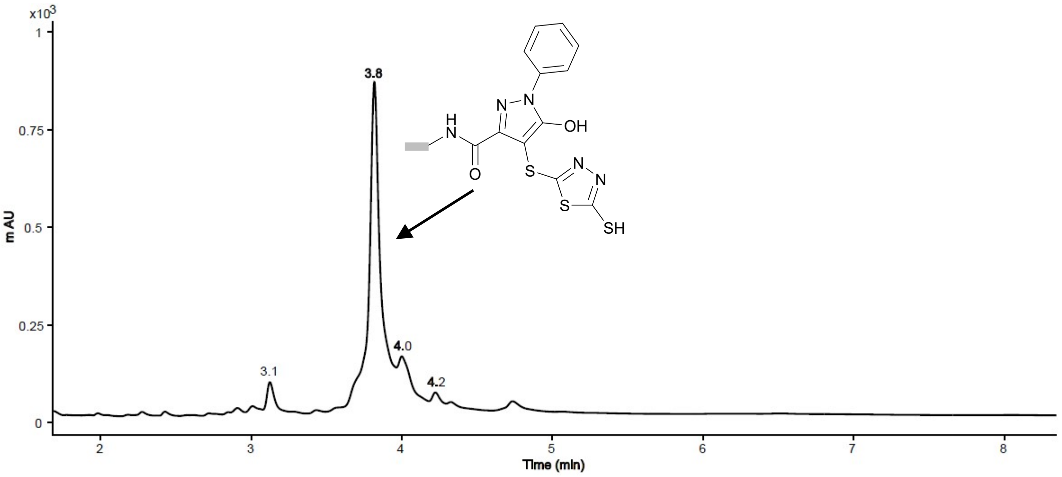


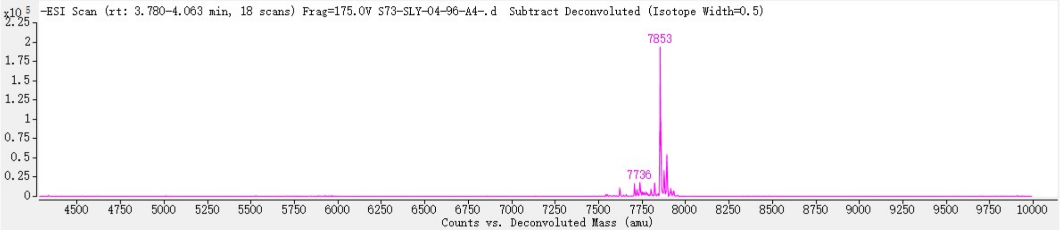


UPLC chromatograph and deconvoluted MS of **5bw.**

**Conversion: 89%**

**Calculated Mass: 7873 Da; Found Mass: 7874 Da**

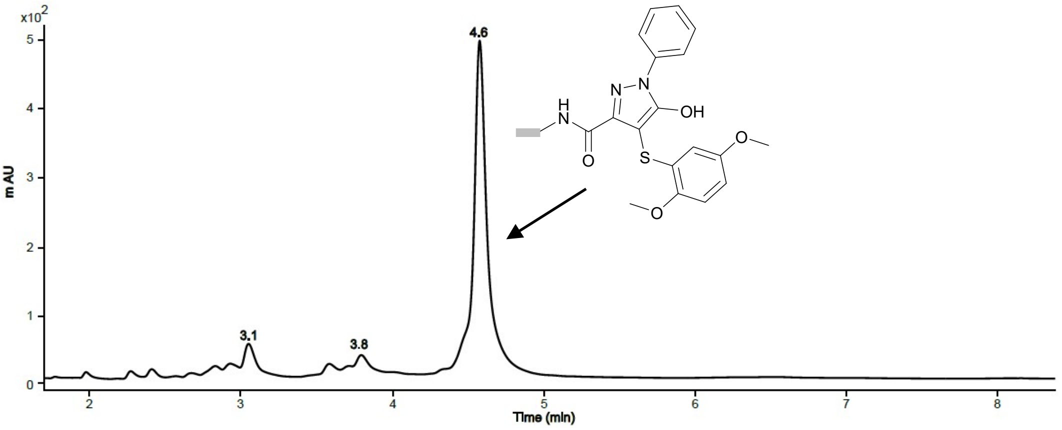


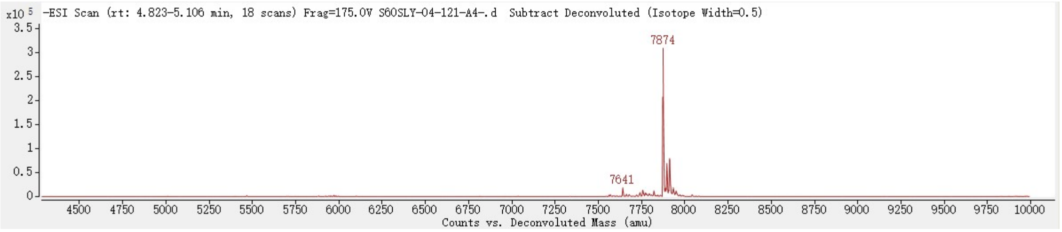

UPLC chromatograph and deconvoluted MS of **5bx.**

**Conversion: 93%**

**Calculated Mass: 7857 Da; Found Mass: 7858 Da**

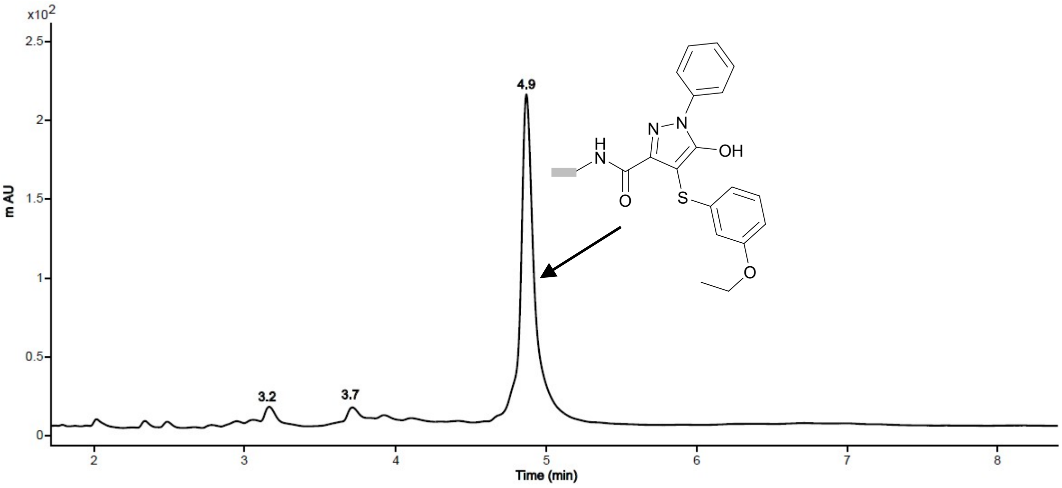


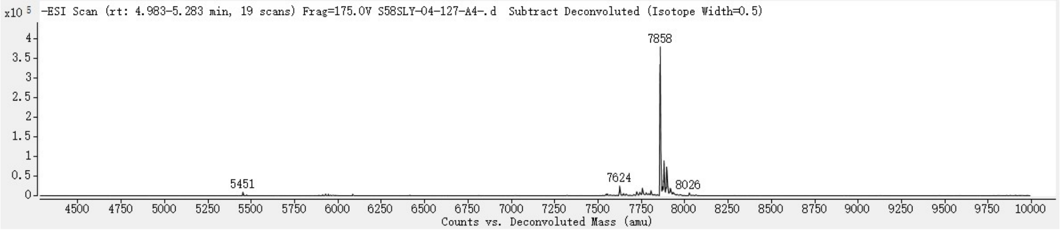


UPLC chromatograph and deconvoluted MS of **5by.**

**Conversion: 87%**

**Calculated Mass: 7828 Da; Found Mass: 7829 Da**

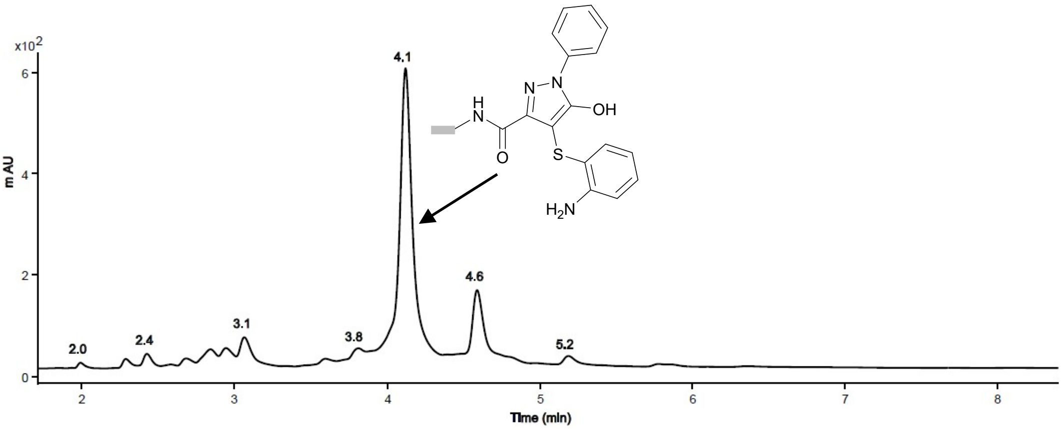


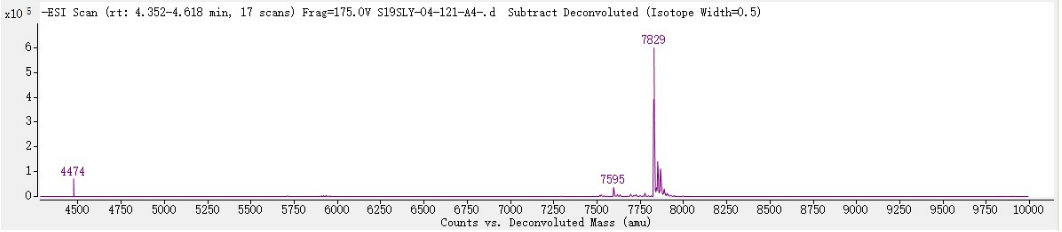

UPLC chromatograph and deconvoluted MS of **5bz.**

**Conversion: 81%**

**Calculated Mass: 7889 Da; Found Mass: 7889 Da**

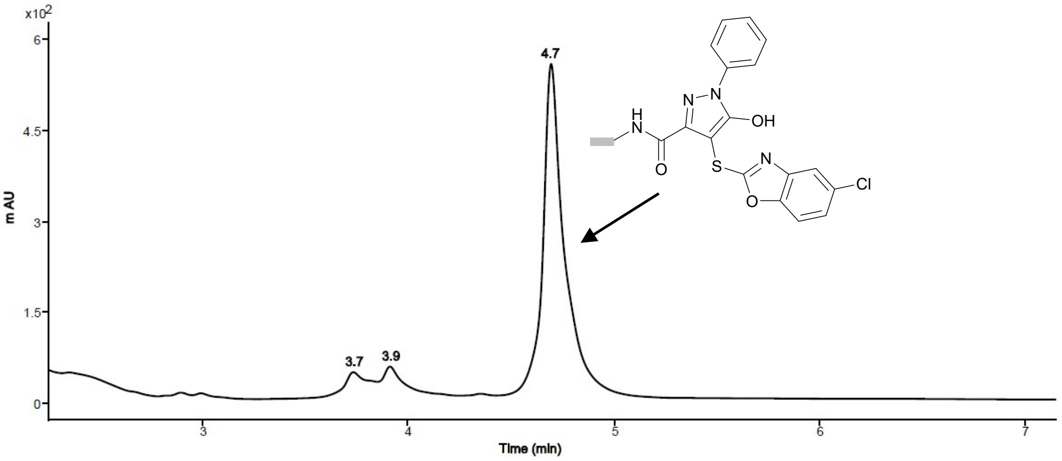


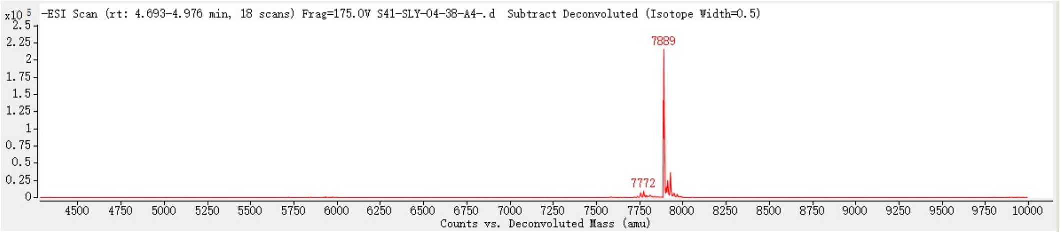


UPLC chromatograph and deconvoluted MS of **5cb.**

**Conversion: 86%**

**Calculated Mass: 7843 Da; Found Mass: 7844 Da**

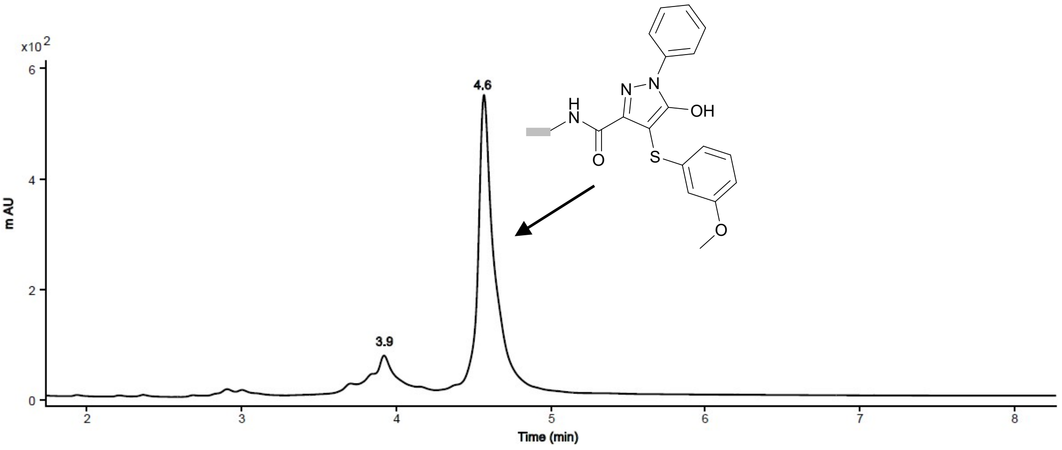


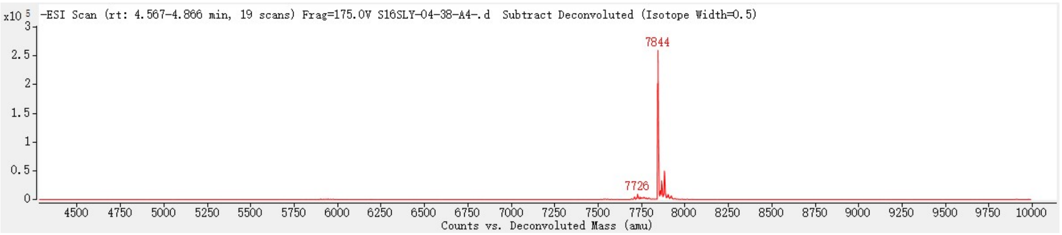

## 6.3 Substrate scope of DNA conjugates for sulfenylation

UPLC chromatograph and deconvoluted MS of **6c**.

**Conversion: 86%**

**Calculated Mass: 7784 Da; Found Mass: 7786 Da**

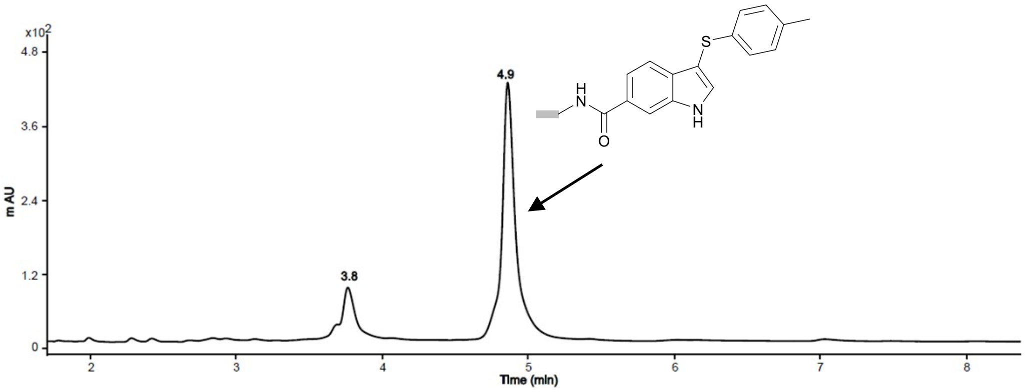


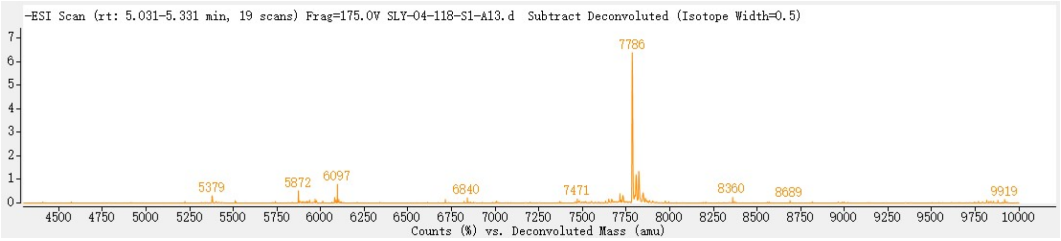


UPLC chromatograph and deconvoluted MS of **6d**.

**Conversion: 52%**

**Calculated Mass: 7784 Da; Found Mass: 7785 Da**

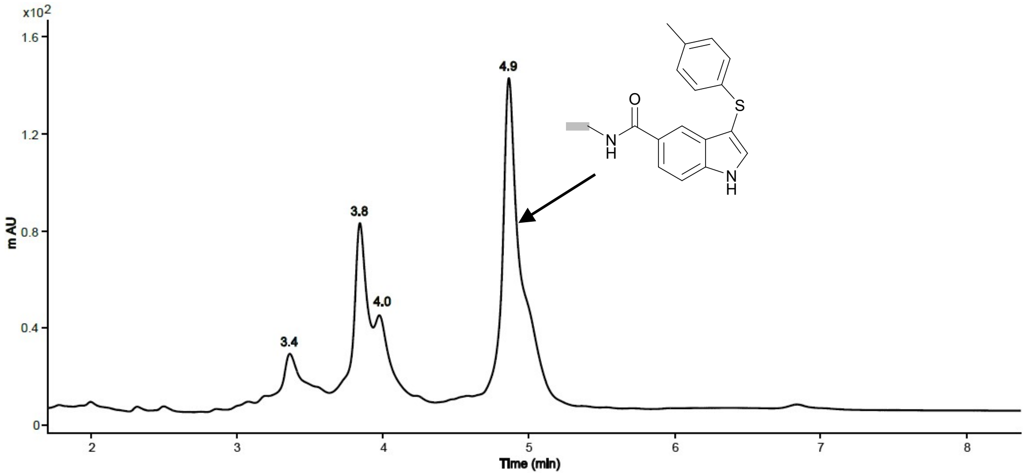


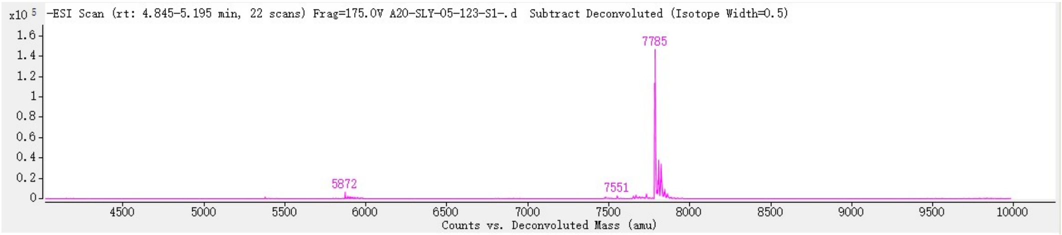

UPLC chromatograph and deconvoluted MS of **6e**.

**Conversion: 93%**

**Calculated Mass: 7798 Da; Found Mass: 7800 Da**

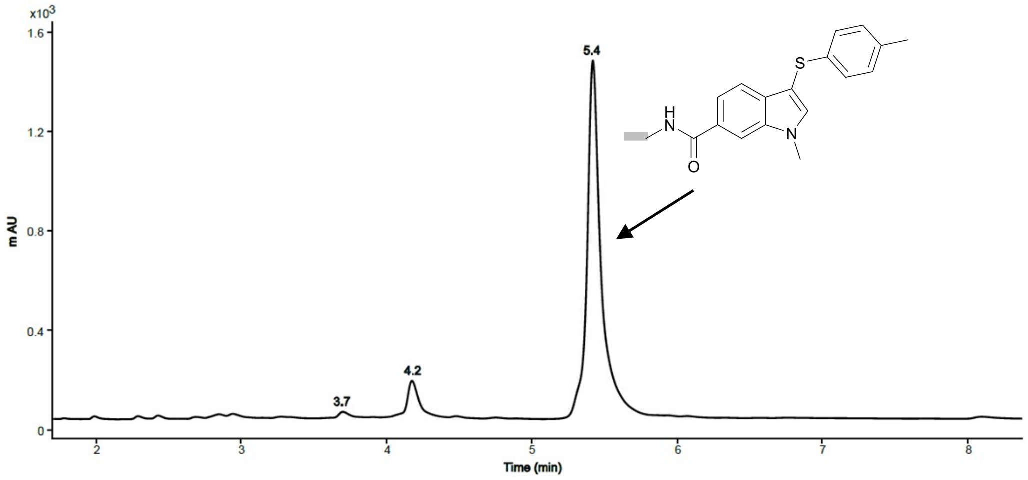


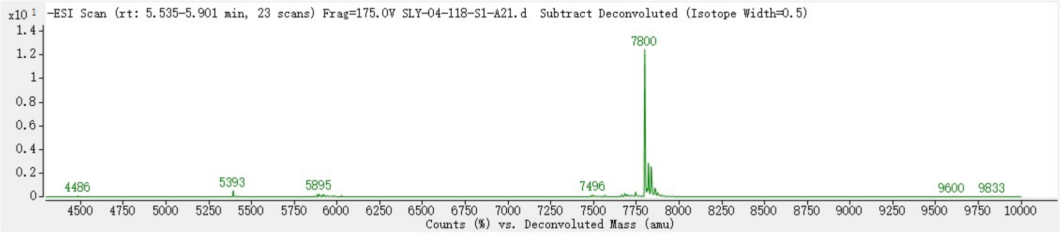


UPLC chromatograph and deconvoluted MS of **6f**.

**Conversion: 87%**

**Calculated Mass: 7798 Da; Found Mass: 7799 Da**

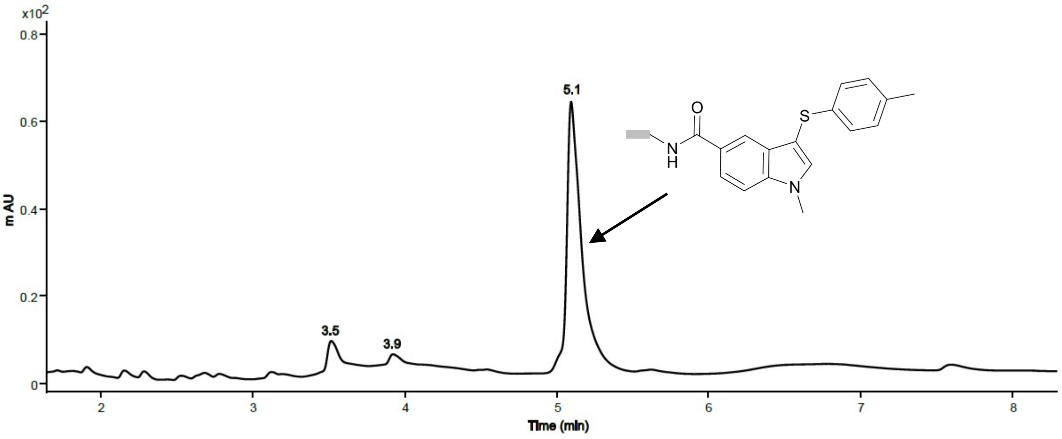


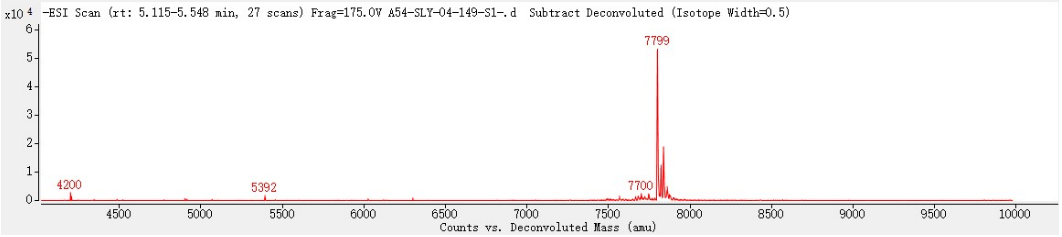

UPLC chromatograph and deconvoluted MS of **6g**.

**Conversion: 92%**

**Calculated Mass: 7798 Da; Found Mass: 7839 (7798+K$^+$) Da**

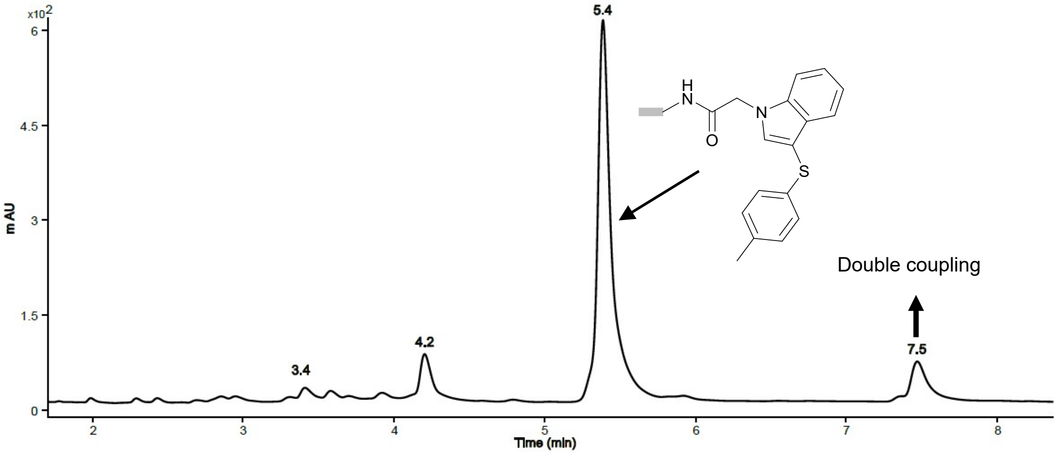


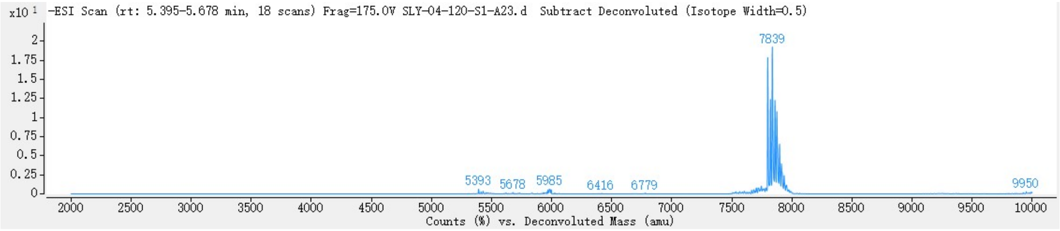


UPLC chromatograph and deconvoluted MS of **6h**.

**Conversion: 91%**

**Calculated Mass: 7814 Da; Found Mass: 7815 Da**

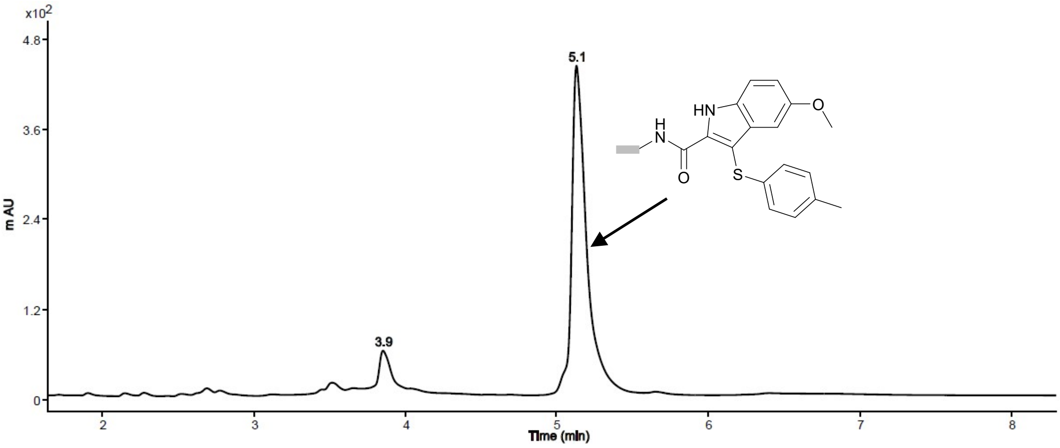


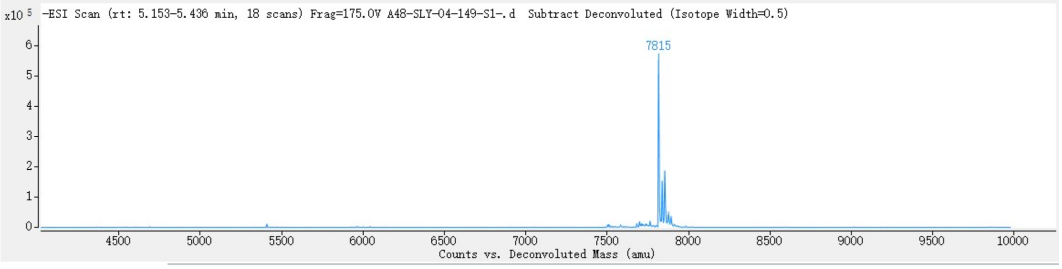

UPLC chromatograph and deconvoluted MS of **6i**.

**Conversion: >95%**

**Calculated Mass: 7784 Da; Found Mass: 7786 Da**

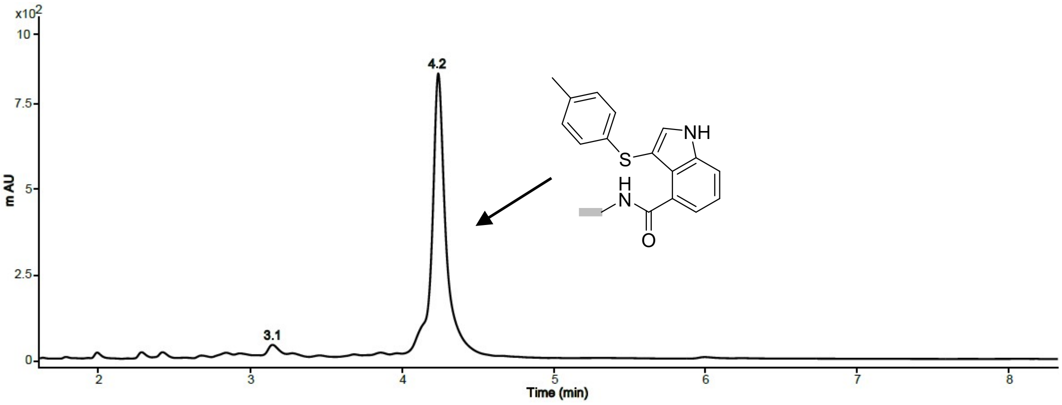


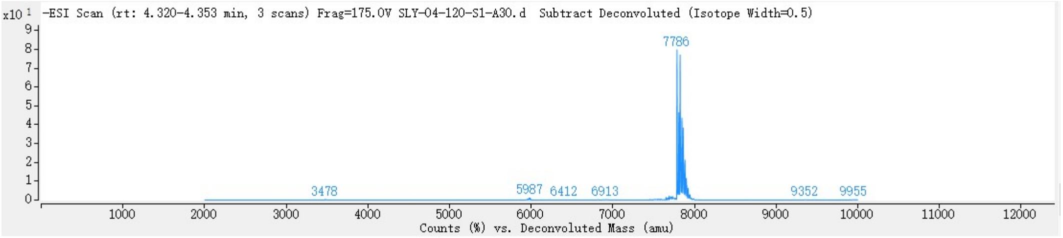


UPLC chromatograph and deconvoluted MS of **6j**.

**Conversion: >95%**

**Calculated Mass: 7826 Da; Found Mass: 7827 Da**

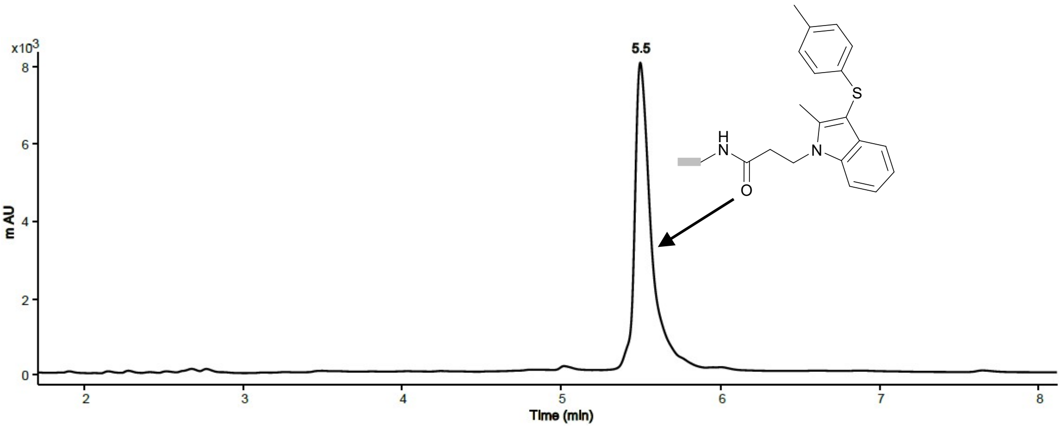


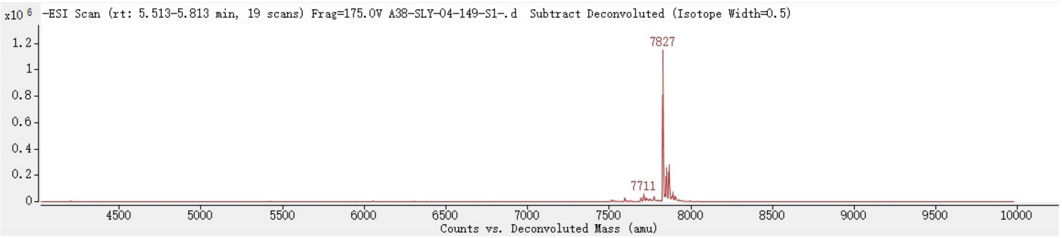

UPLC chromatograph and deconvoluted MS of **6k**.

**Conversion: 81%**

**Calculated Mass: 7812 Da; Found Mass: 7812 Da**

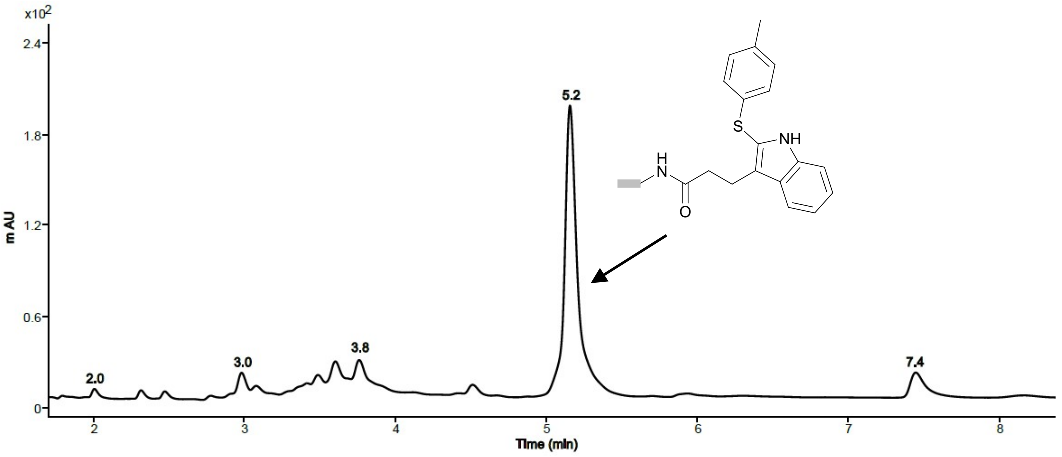


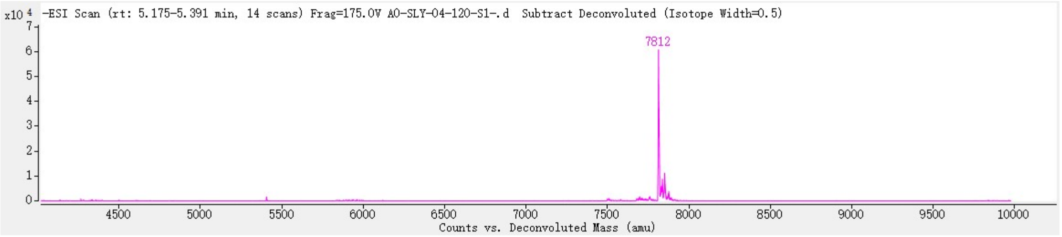


UPLC chromatograph and deconvoluted MS of **6l**.

**Conversion: 76%**

**Calculated Mass: 7869 Da; Found Mass: 7869 Da**

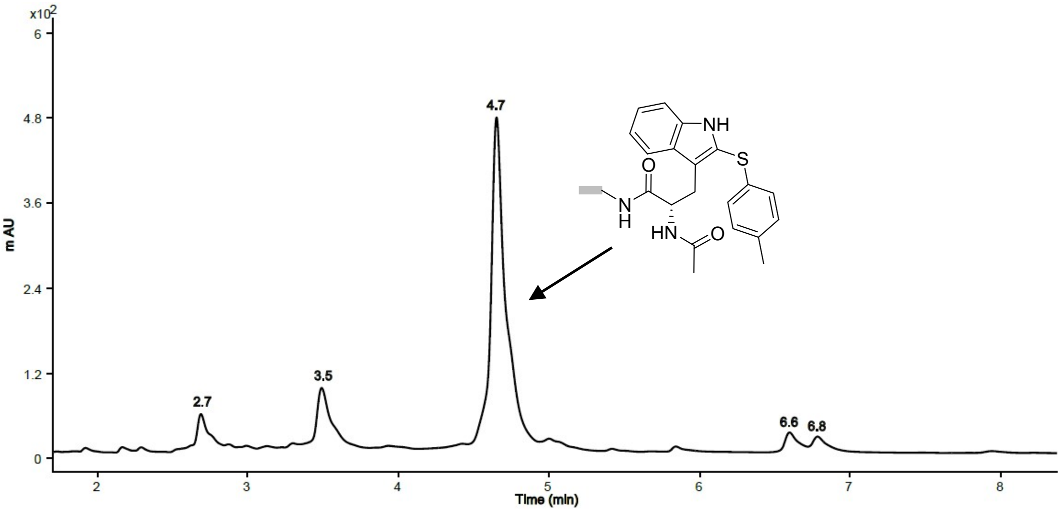


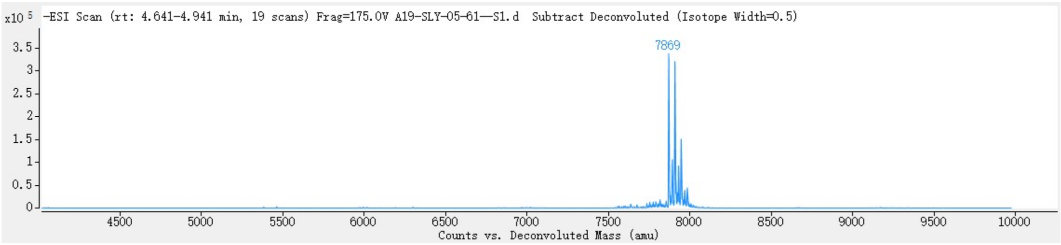

UPLC chromatograph and deconvoluted MS of **6m**.

**Conversion: 82%**

**Calculated Mass: 7867 Da; Found Mass: 7868 Da**

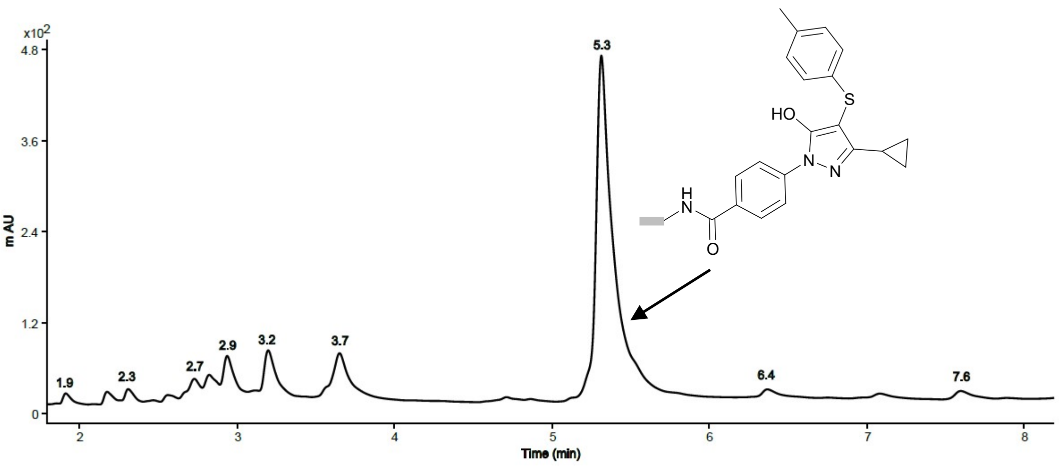


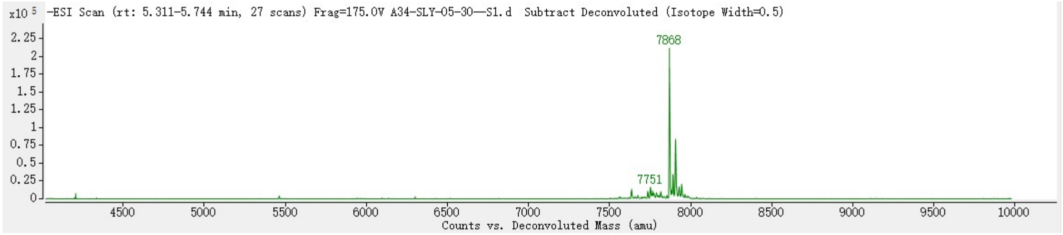


UPLC chromatograph and deconvoluted MS of **6n**.

**Conversion: 91%**

**Calculated Mass: 7788 Da; Found Mass: 7789 Da**

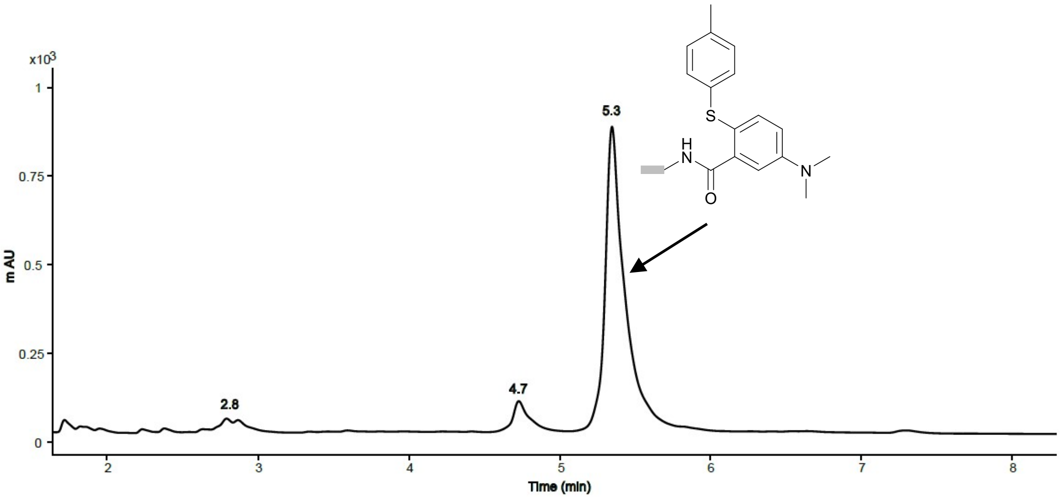


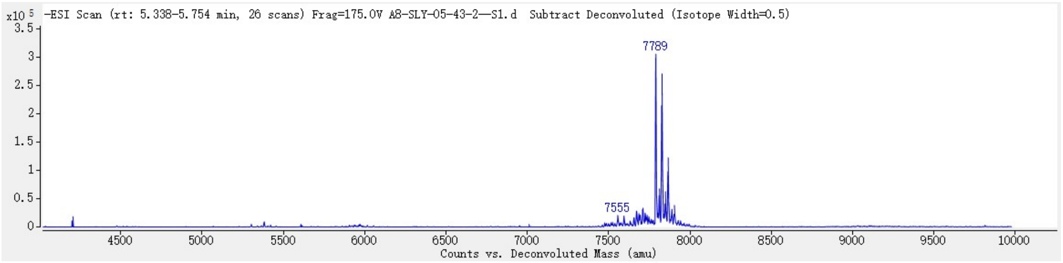

UPLC chromatograph and deconvoluted MS of **6o**.

**Conversion: 82%**

**Calculated Mass: 7830 Da; Found Mass: 7831 Da**

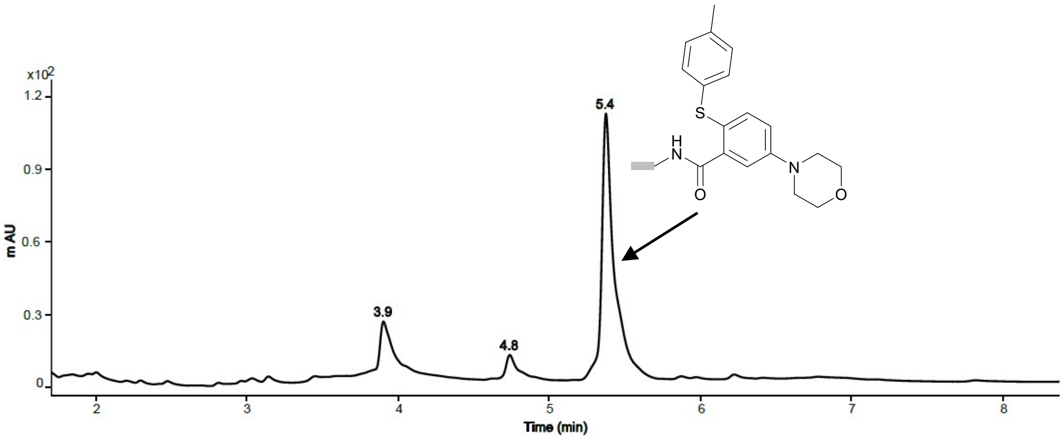


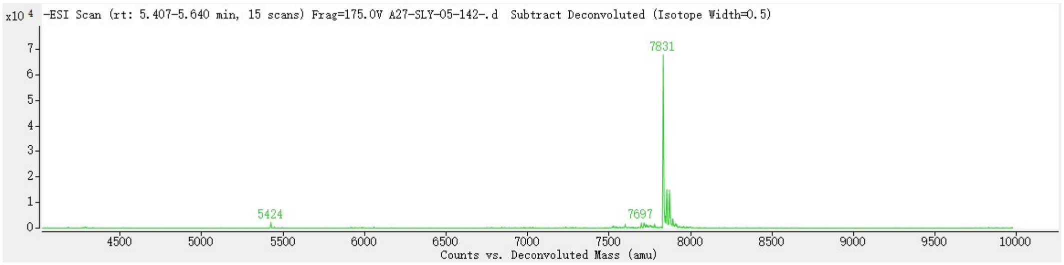


UPLC chromatograph and deconvoluted MS of **6p**.

**Conversion: 95%**

**Calculated Mass: 7833 Da; Found Mass: 7834 Da**

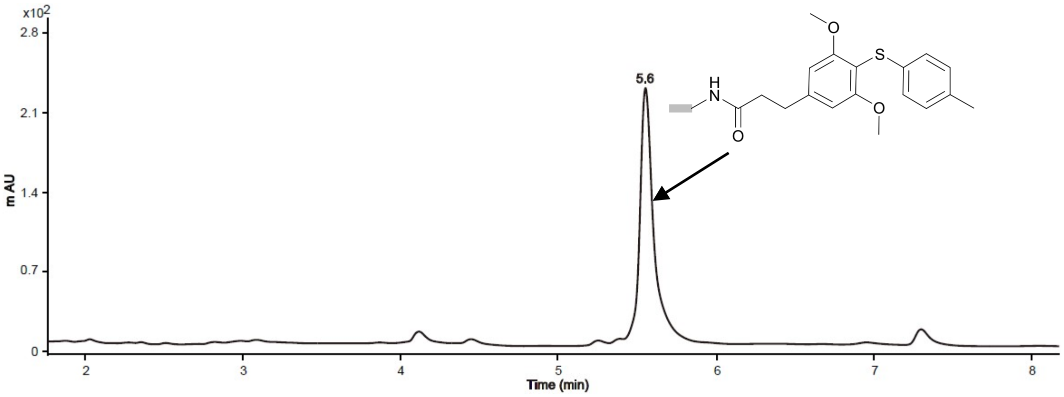


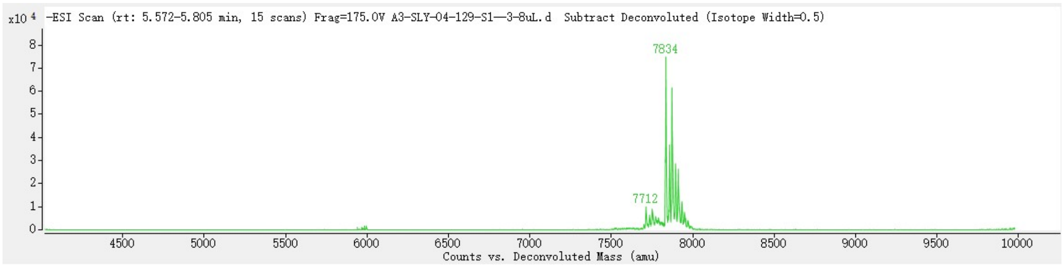

UPLC chromatograph and deconvoluted MS of **6q**.

**Conversion: 95%**

**Calculated Mass: 7849 Da; Found Mass: 7850 Da**

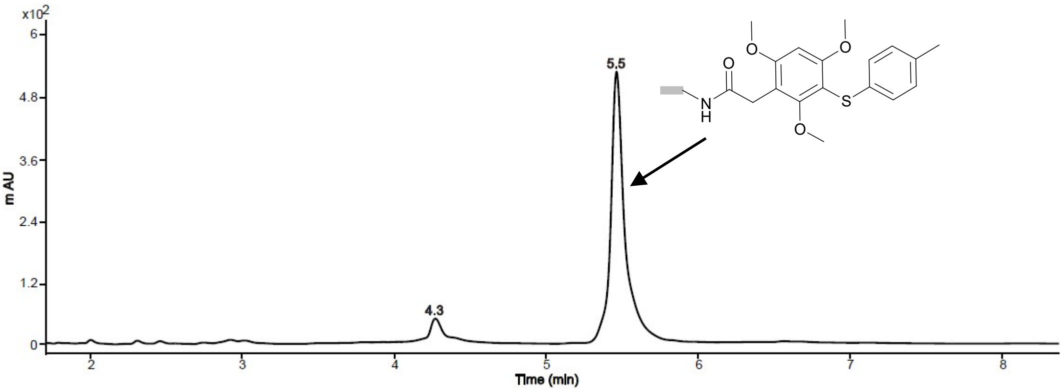


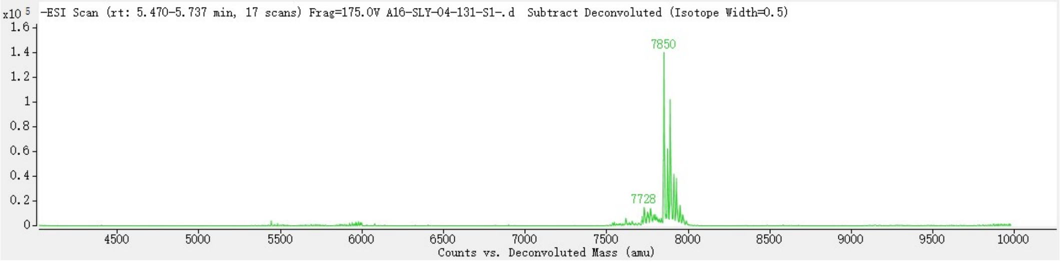


UPLC chromatograph and deconvoluted MS of **6r**.

**Conversion: 82%**

**Calculated Mass: 7853 Da; Found Mass: 7853 Da**

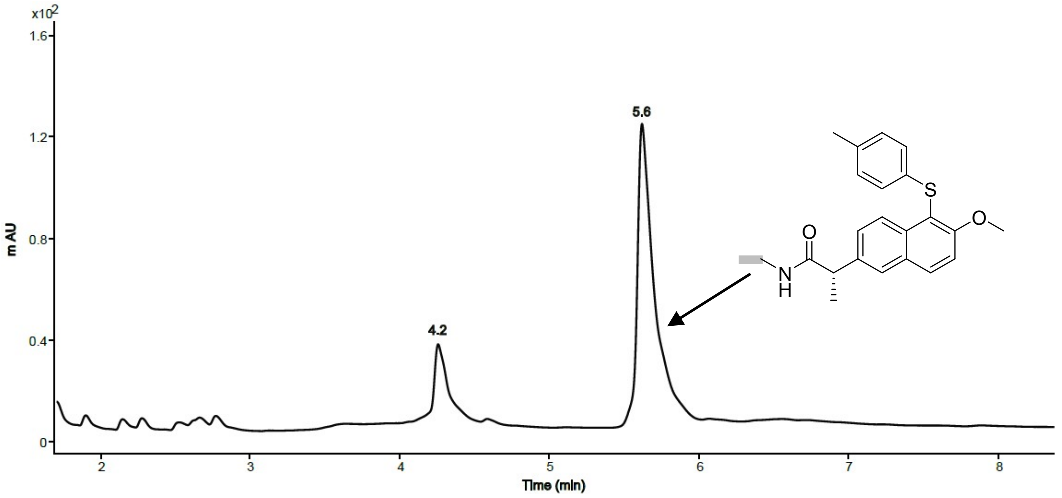


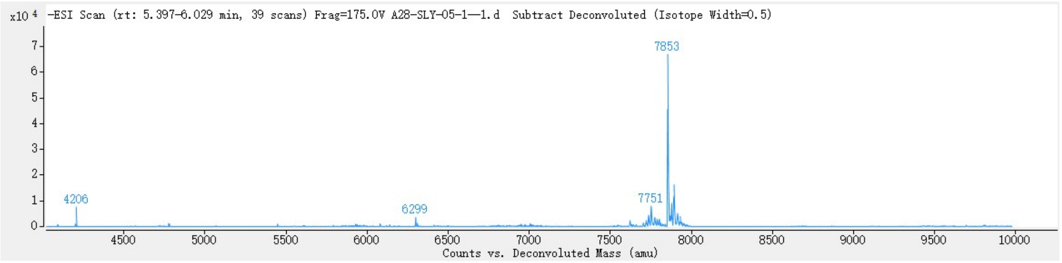

UPLC chromatograph and deconvoluted MS of **6s**.

**Conversion: 35%**

**Calculated Mass: 7785 Da; Found Mass: 7786 Da**

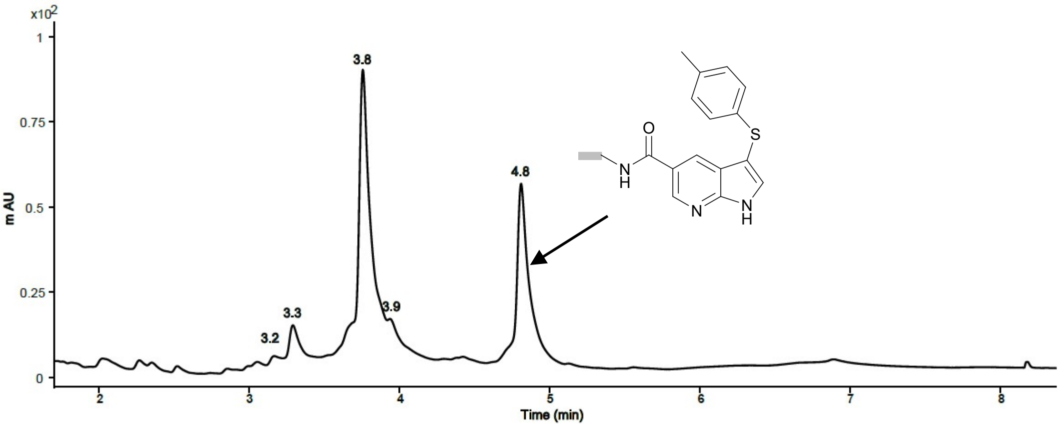


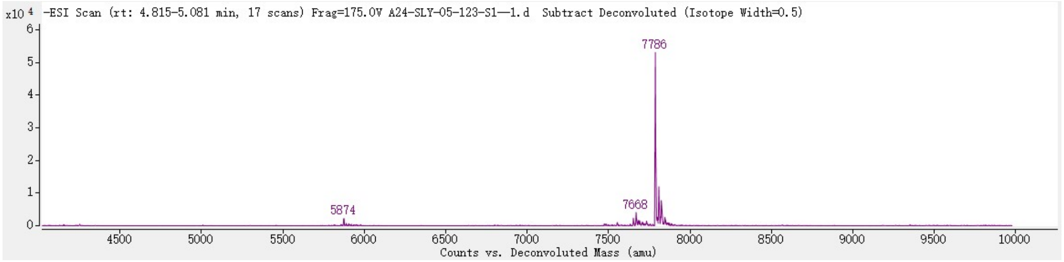


UPLC chromatograph and deconvoluted MS of **6t**.

**Conversion: 56%**

**Calculated Mass: 7791 Da; Found Mass: 7792 Da**

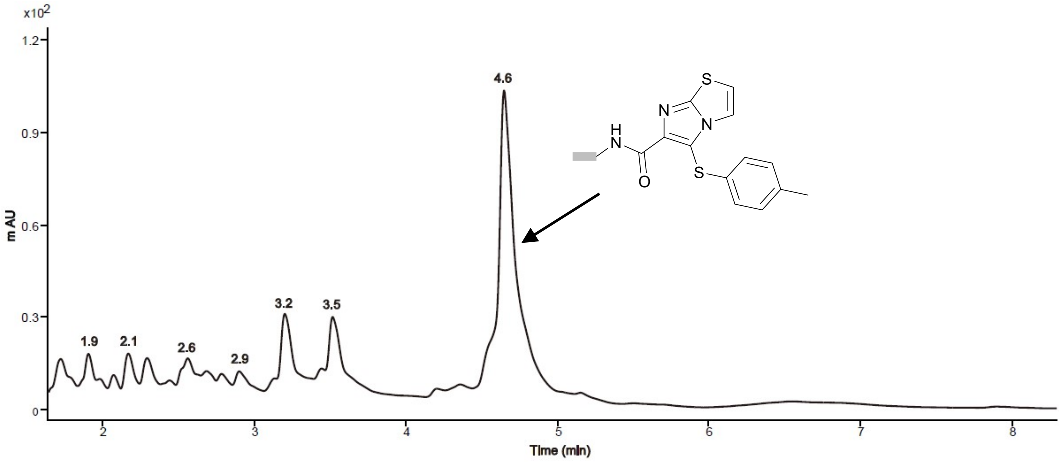


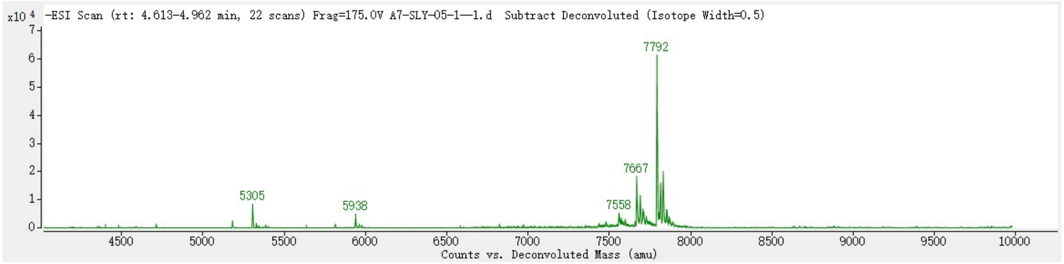

UPLC chromatograph and deconvoluted MS of **6u**.

**Conversion: 98%**

**Calculated Mass: 7855 Da; Found Mass: 7856 Da**

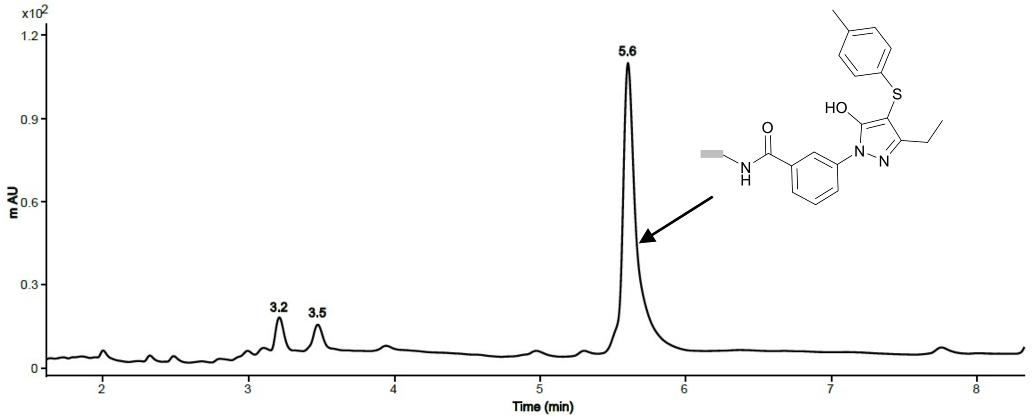


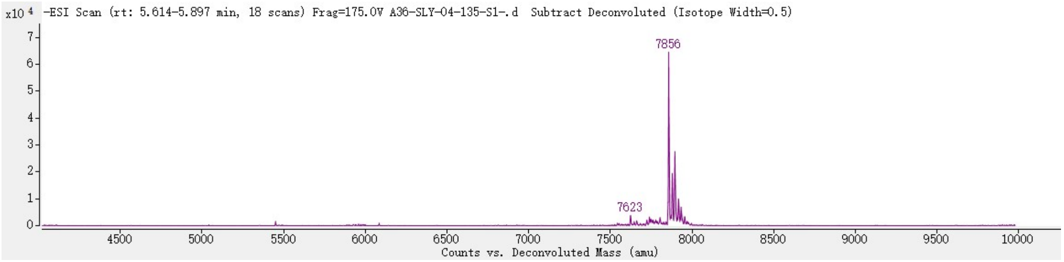


UPLC chromatograph and deconvoluted MS of **6v**.

**Conversion: 86%**

**Calculated Mass: 7819 Da; Found Mass: 7820 Da**

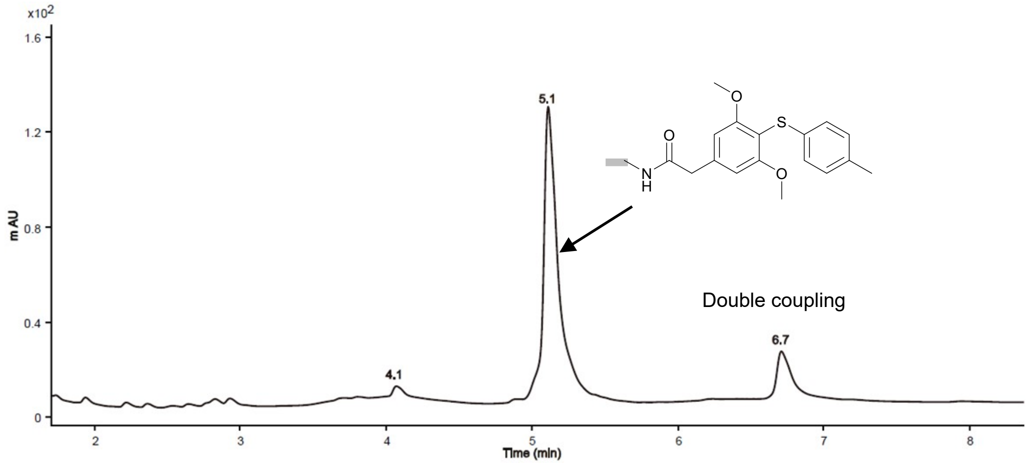


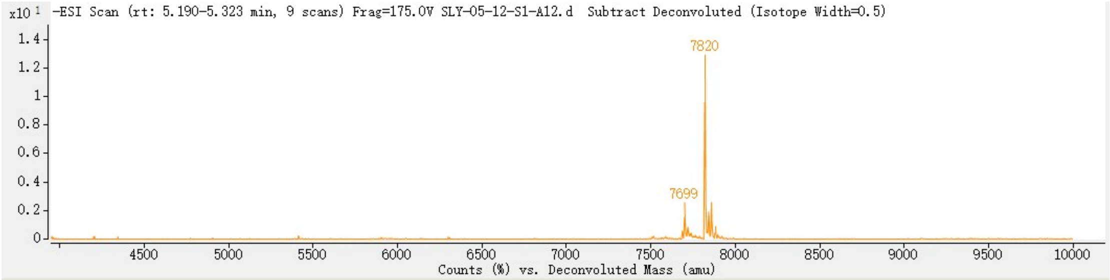


UPLC chromatograph and deconvoluted MS of **6w**.

**Conversion: 83%**

**Calculated Mass: 7798 Da; Found Mass: 7799 Da**

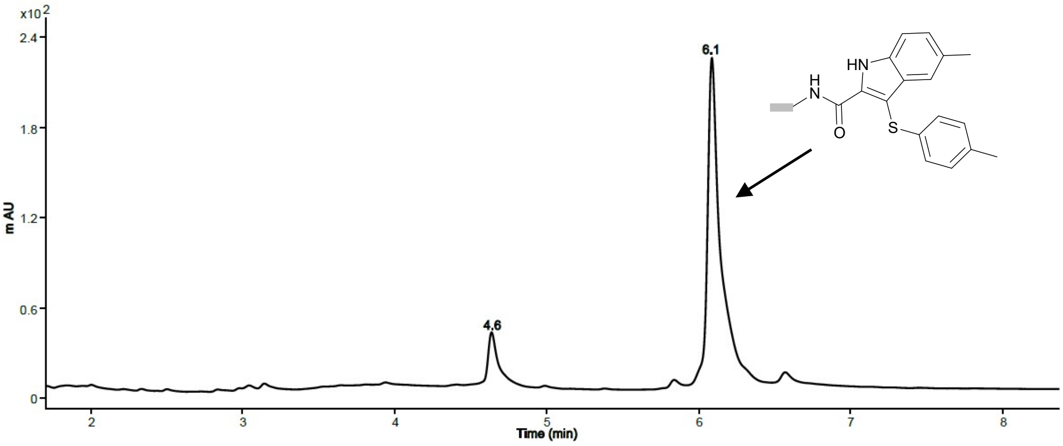


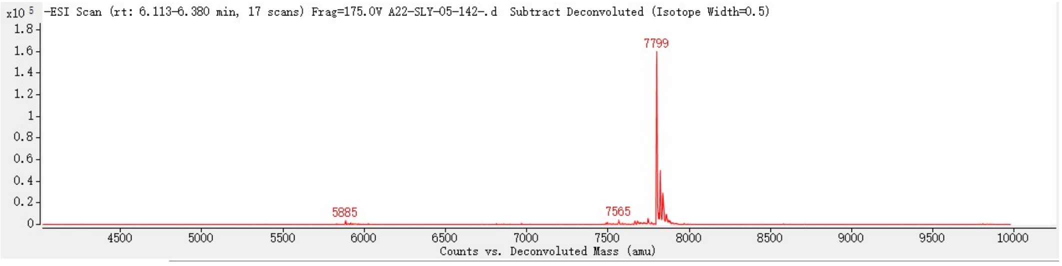


UPLC chromatograph and deconvoluted MS of **6x**.

**Conversion: 23%**

**Calculated Mass: 7788 Da; Found Mass: 7789 Da**

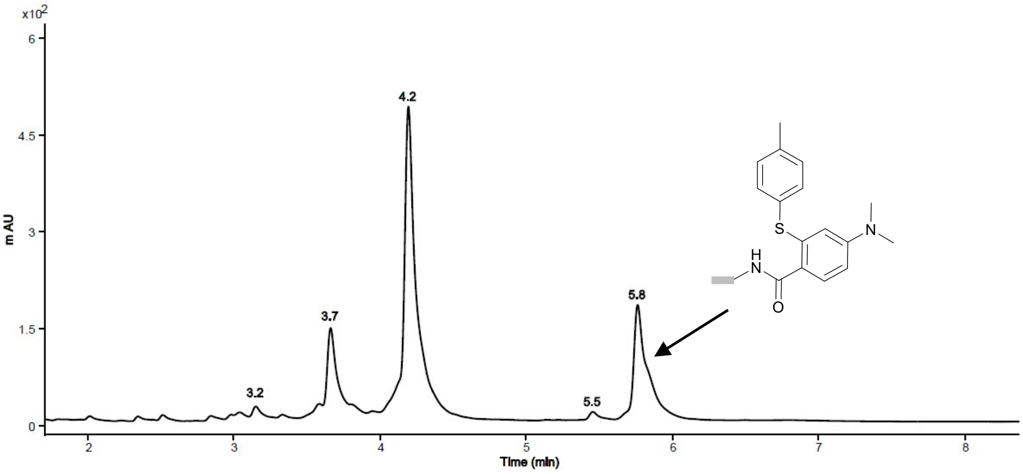


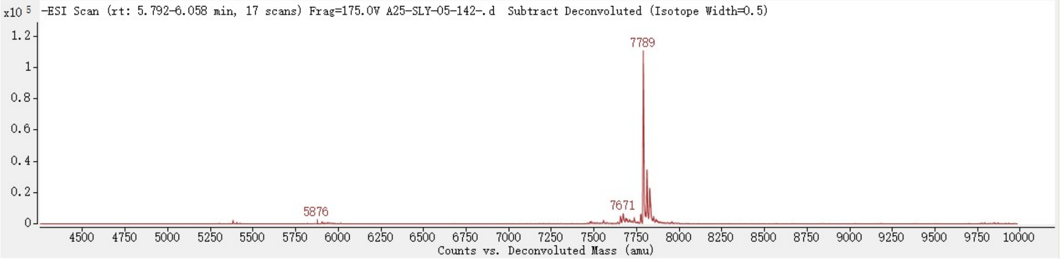


UPLC chromatograph and deconvoluted MS of **6y**.

**Conversion: 92%**
**Calculated Mass: 7812 Da; Found Mass: 7813 Da**

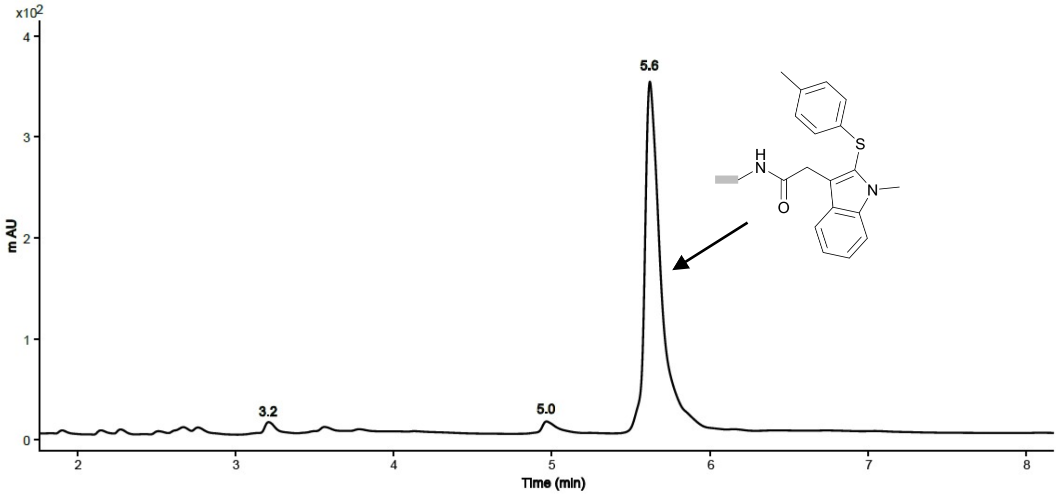


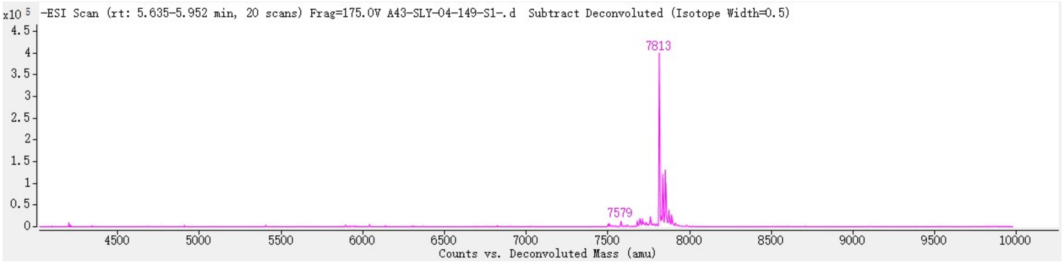


UPLC chromatograph and deconvoluted MS of **6z**.

**Conversion: 85%**
**Calculated Mass: 7828 Da; Found Mass: 7829 Da**

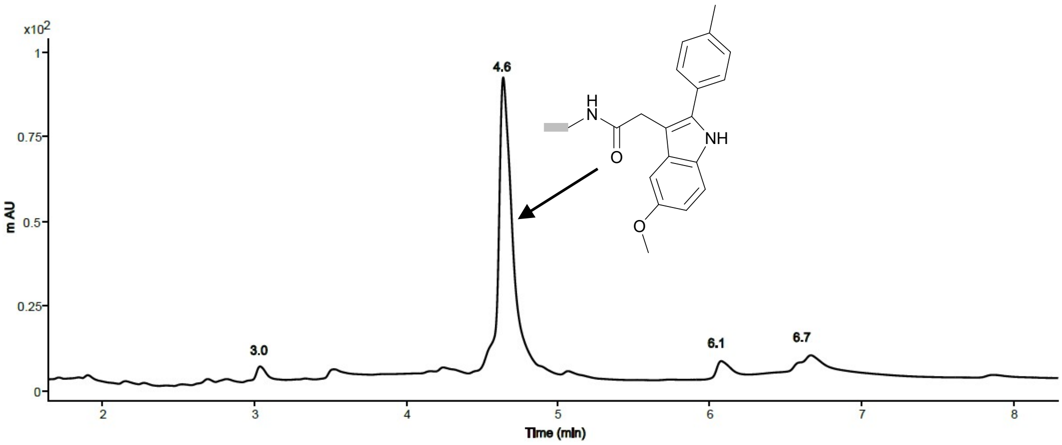


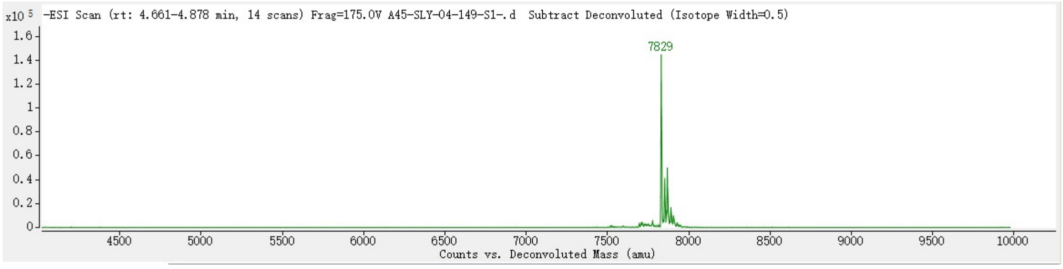


UPLC chromatograph and deconvoluted MS of **6aa**.

**Conversion: 90%**

**Calculated Mass: 7841 Da; Found Mass: 7842 Da**

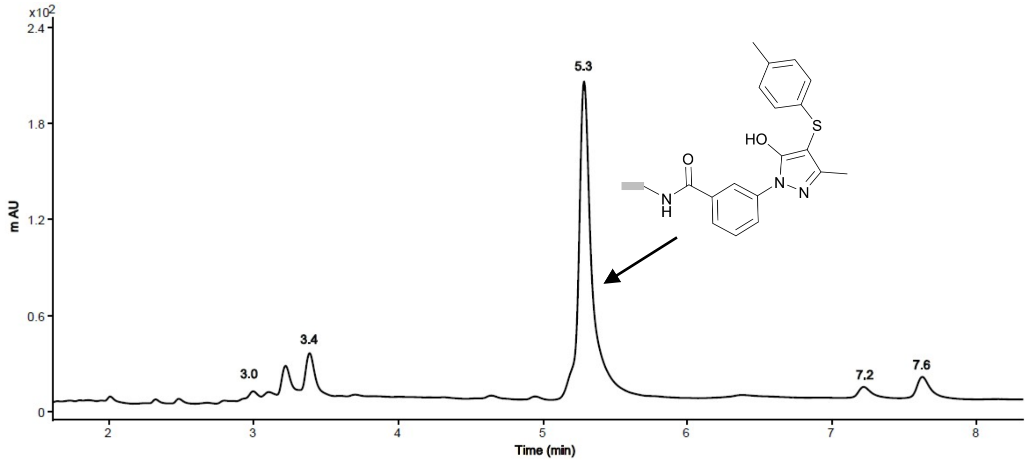


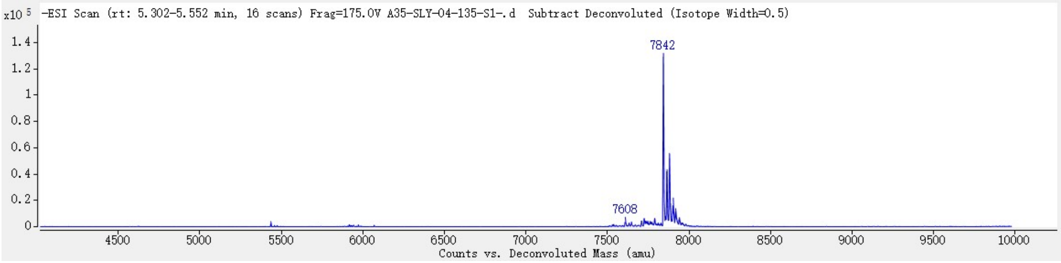


UPLC chromatograph and deconvoluted MS of **6ab**.

**Conversion: 65%**

**Calculated Mass: 7784 Da; Found Mass: 7784 Da**

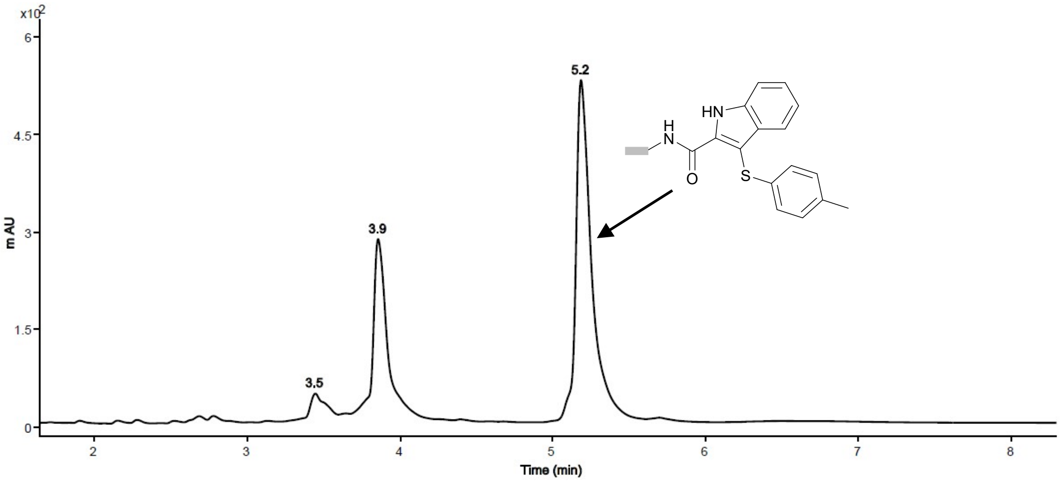


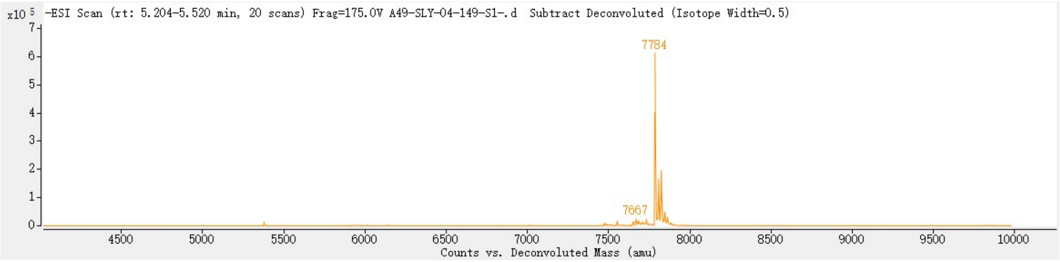


UPLC chromatograph and deconvoluted MS of **6ac**.

**Conversion: 6%**

**Calculated Mass: 7876 Da; Found Mass: 7878 Da**

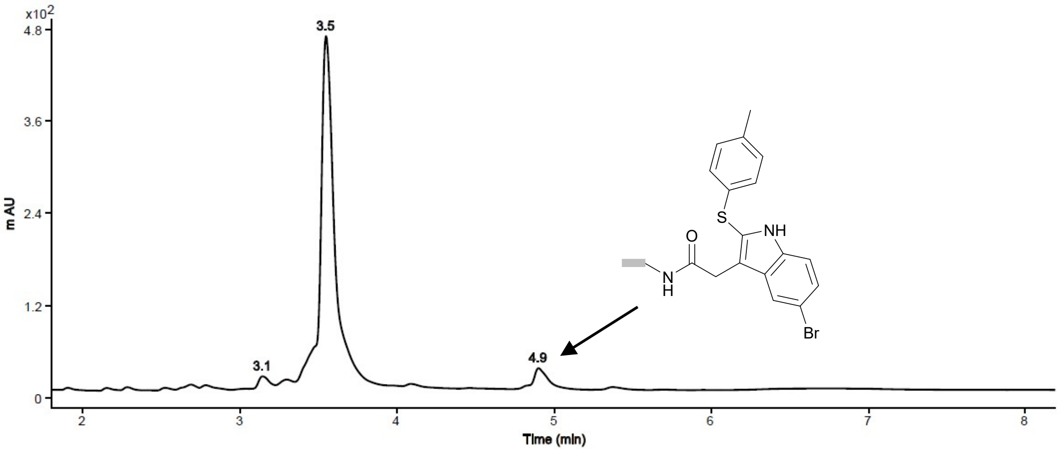


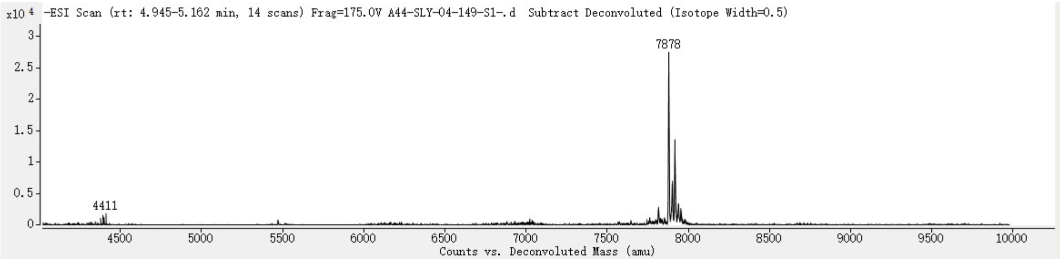


UPLC chromatograph and deconvoluted MS of **6ad**.

**Conversion: 14%**

**Calculated Mass: 7802 Da; Found Mass: 7803 Da**

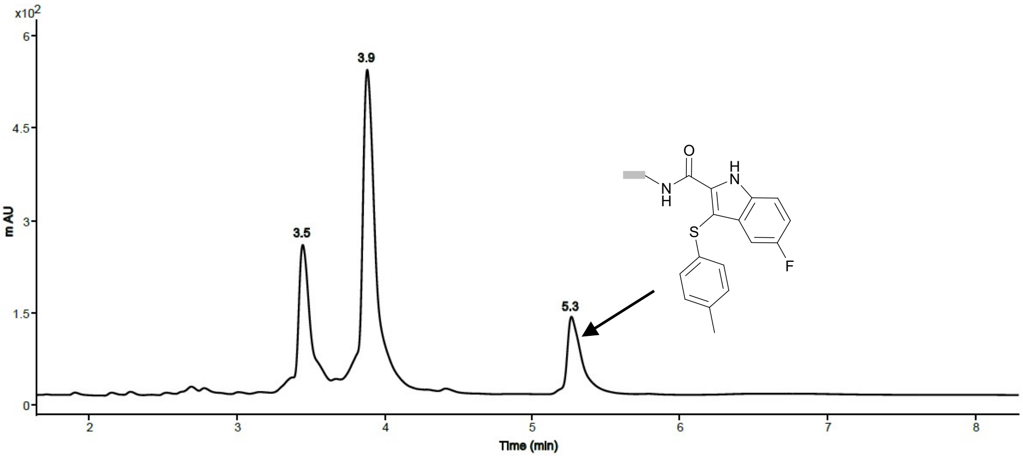


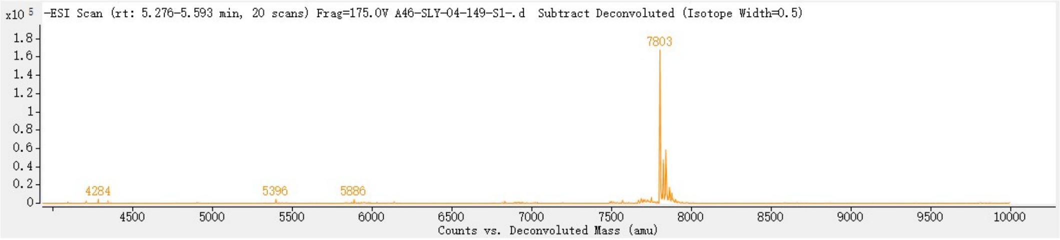


UPLC chromatograph and deconvoluted MS of **6ae**.

**Conversion: 88%**

**Calculated Mass: 7855 Da; Found Mass: 7856 Da**

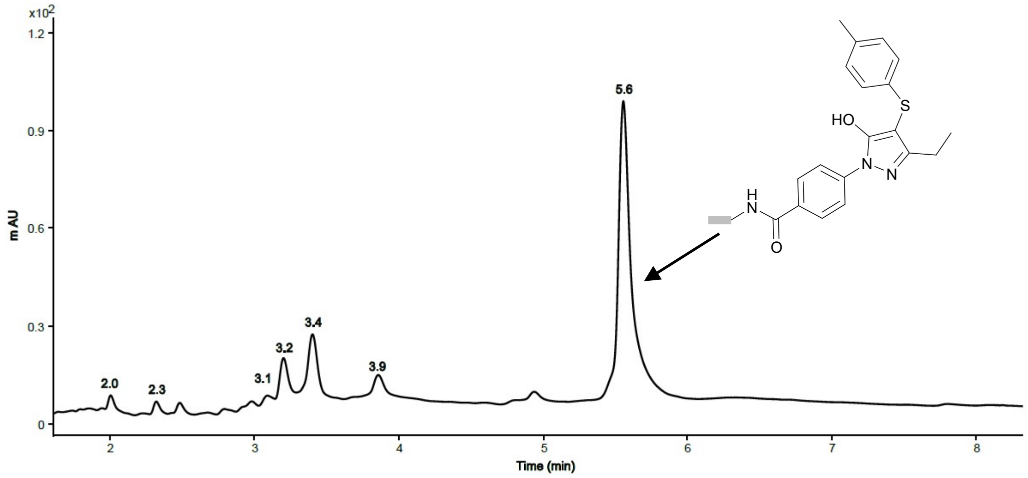


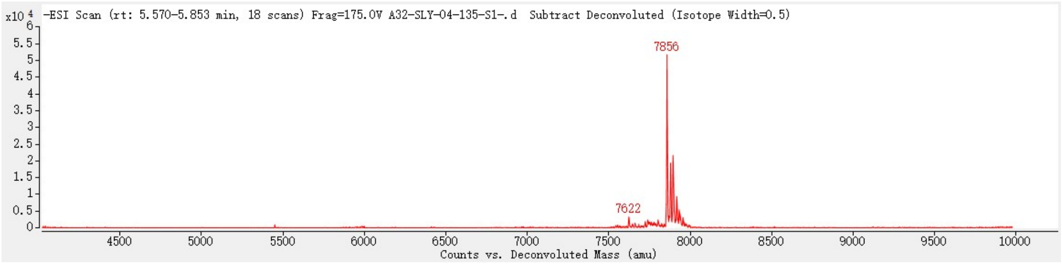

## 6.4 Substrate scope of DNA conjugates for selenylation

UPLC chromatograph and deconvoluted MS of **10c.**

**Conversion: 82%**

**Calculated Mass: 7818 Da; Found Mass: 7819 Da**

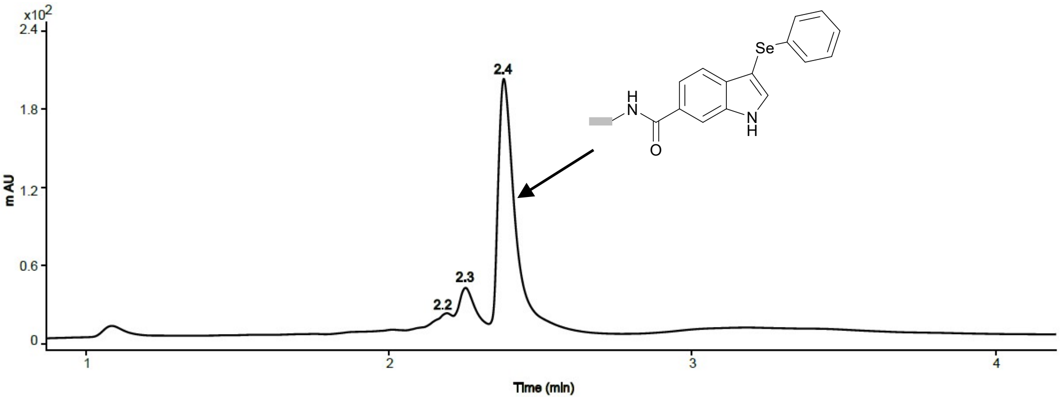


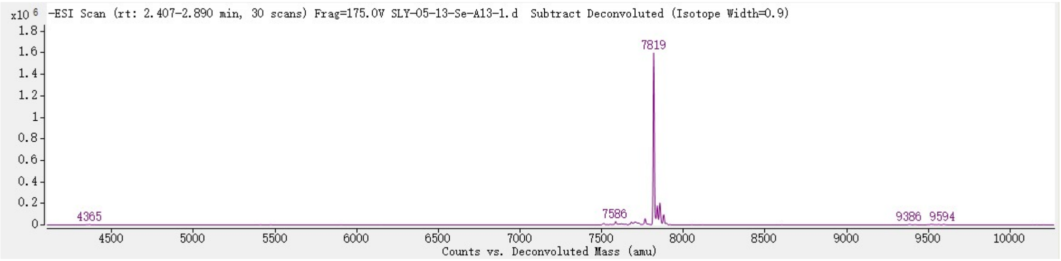


UPLC chromatograph and deconvoluted MS of **10d.**

**Conversion: 77%**

**Calculated Mass: 7818 Da; Found Mass: 7819 Da**

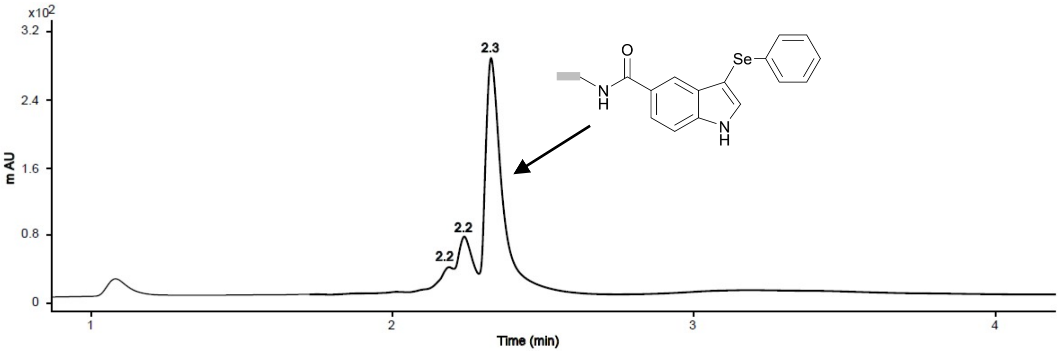


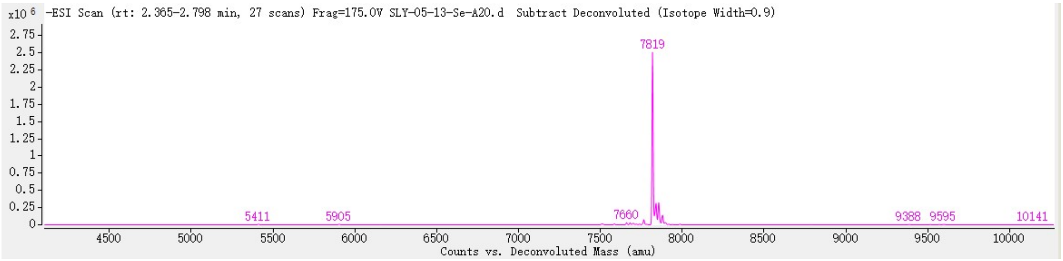

UPLC chromatograph and deconvoluted MS of **10e.**

**Conversion: 85%**

**Calculated Mass: 7832 Da; Found Mass: 7833 Da**

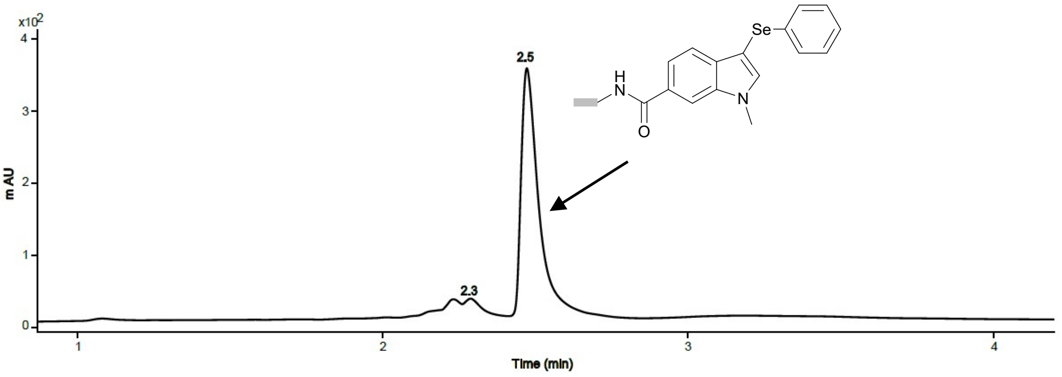


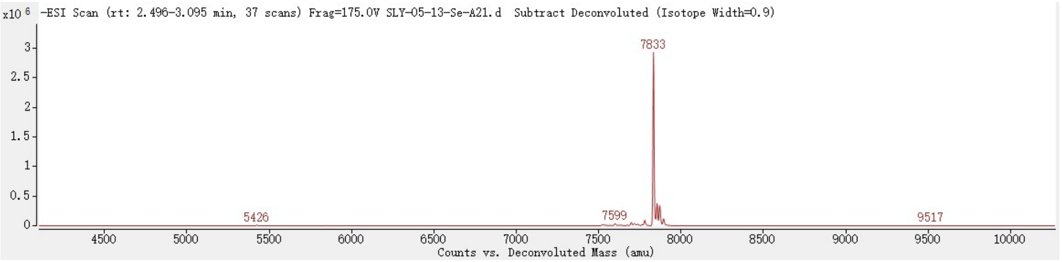


UPLC chromatograph and deconvoluted MS of **10f.**

**Conversion: 88%**

**Calculated Mass: 7832 Da; Found Mass: 7833 Da**

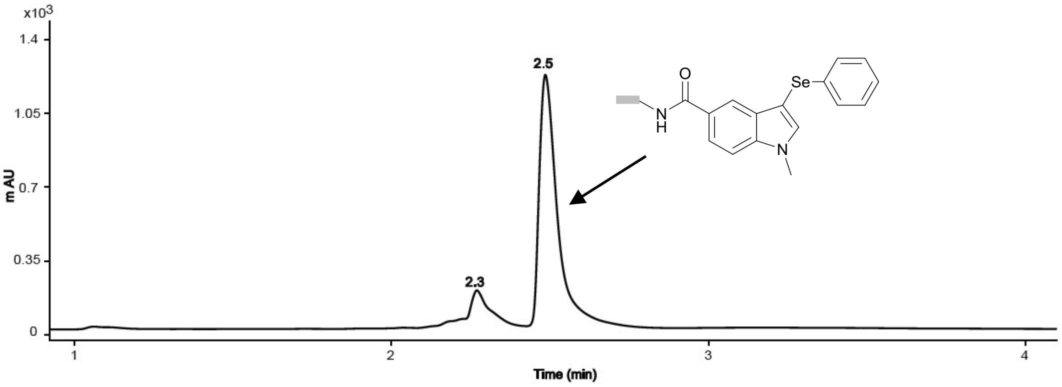


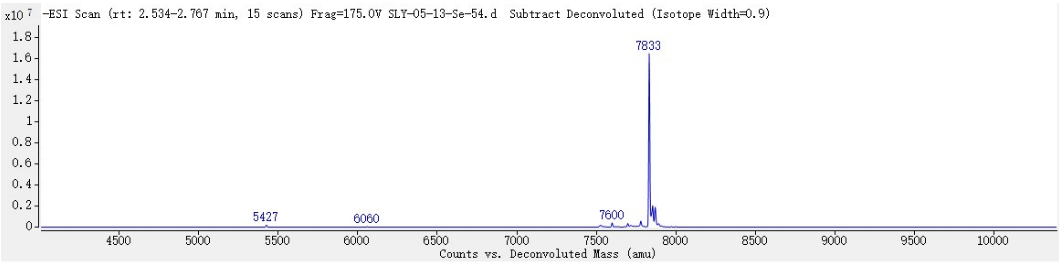

UPLC chromatograph and deconvoluted MS of **10g.**

**Conversion: 78%**

**Calculated Mass: 7832 Da; Found Mass: 7833 Da**

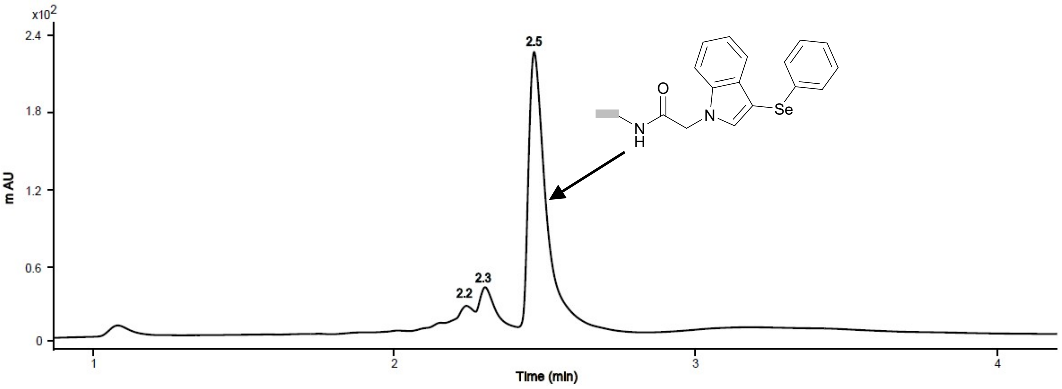


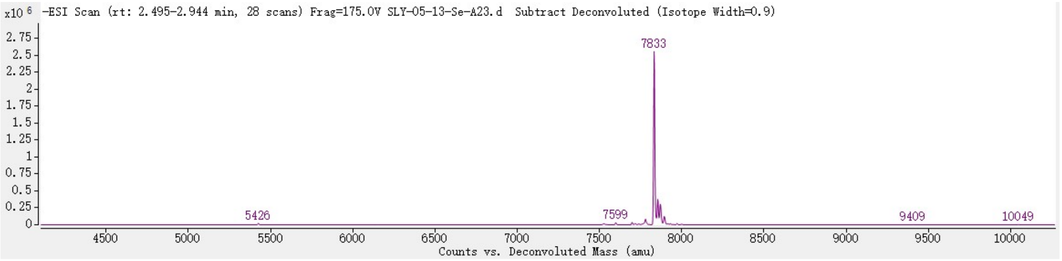


UPLC chromatograph and deconvoluted MS of **10h.**

**Conversion: 88%**

**Calculated Mass: 7848 Da; Found Mass: 7849 Da**

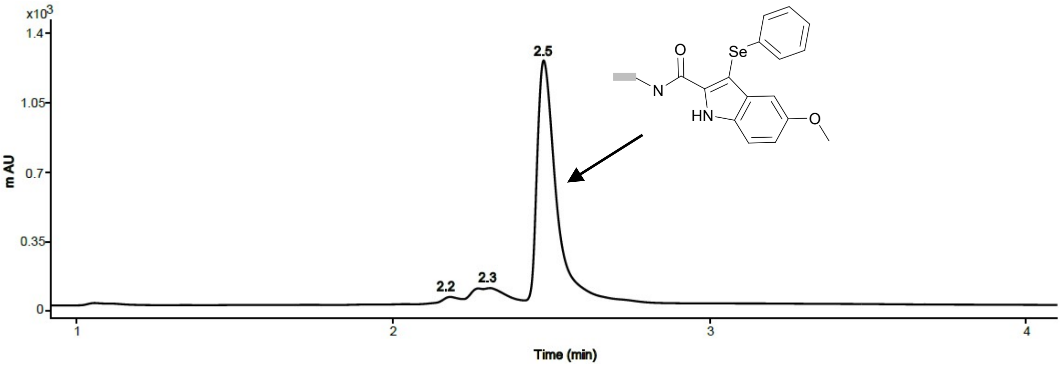


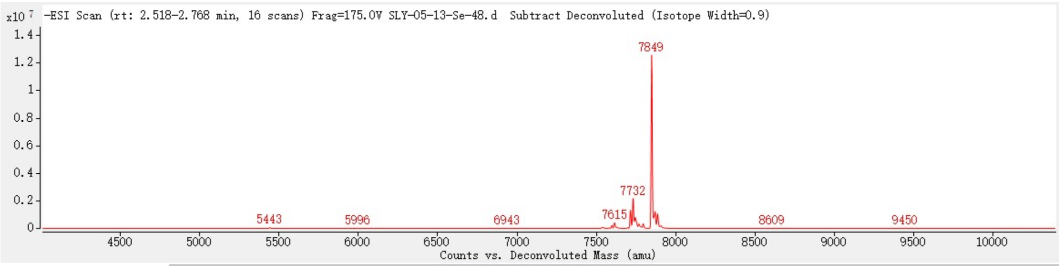

UPLC chromatograph and deconvoluted MS of **10p.**

**Conversion: 50%**

**Calculated Mass: 7867 Da; Found Mass: 7868 Da**

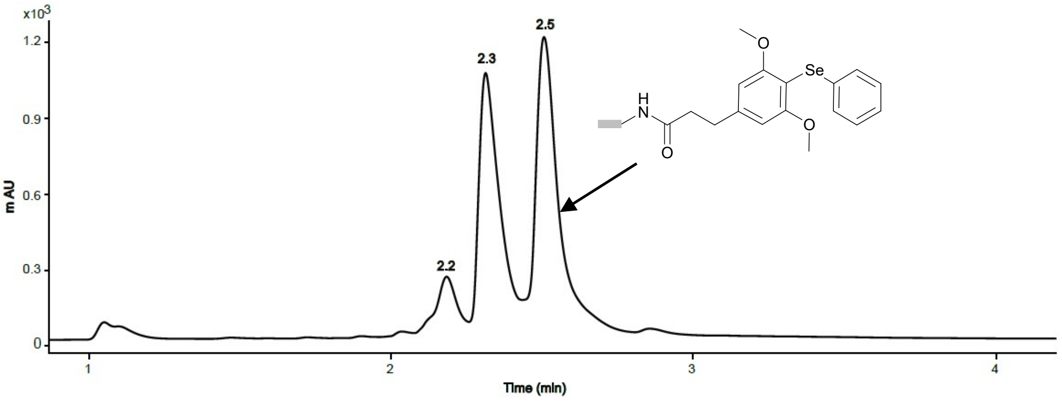


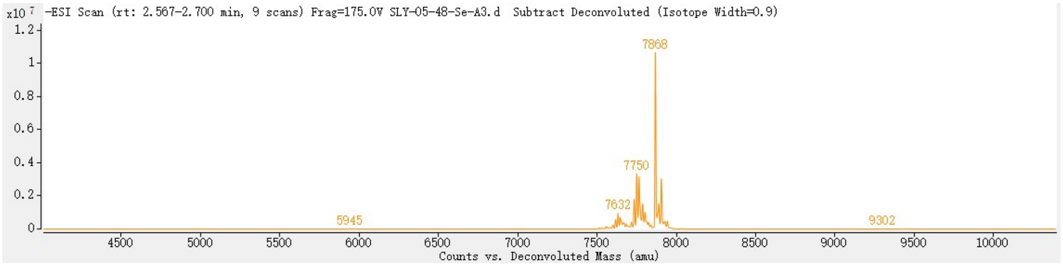


UPLC chromatograph and deconvoluted MS of **10s.**

**Conversion: 36%**

**Calculated Mass: 7819 Da; Found Mass: 7819 Da**

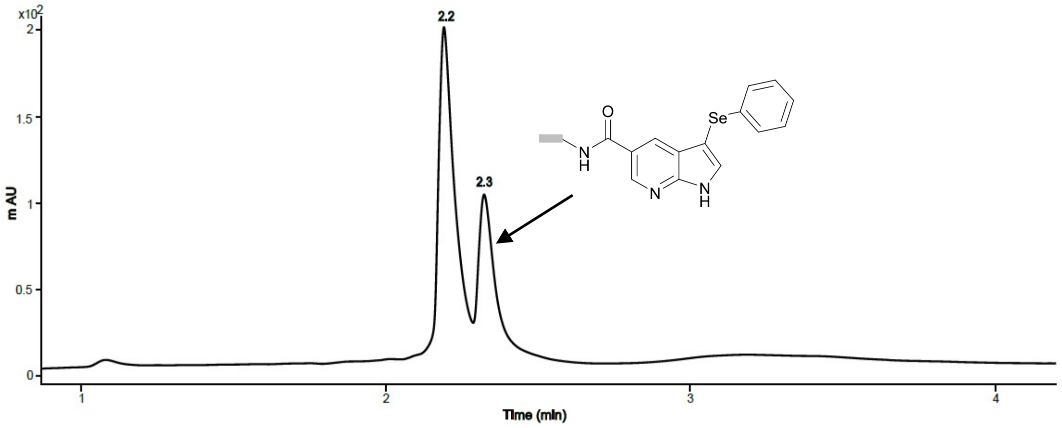


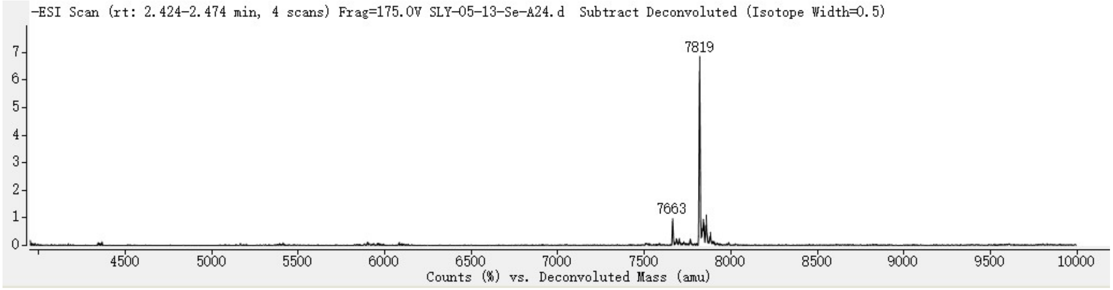

UPLC chromatograph and deconvoluted MS of **10t.**

**Conversion: 10%**

**Calculated Mass: 7825 Da; Found Mass: 7825 Da**

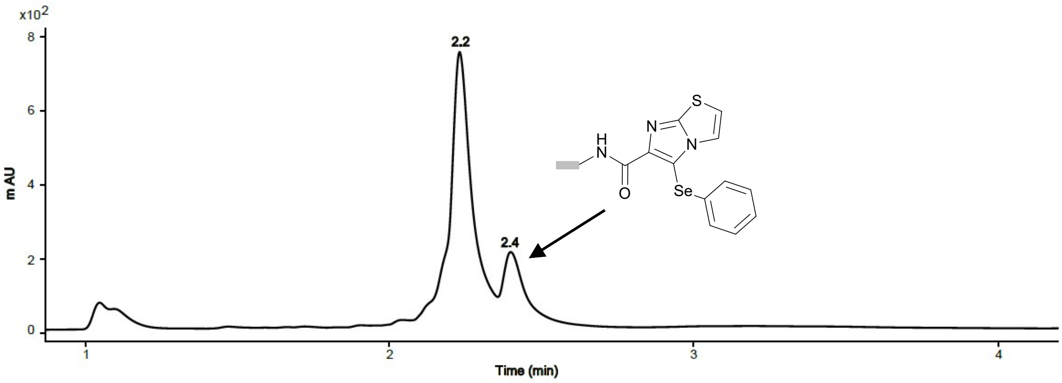


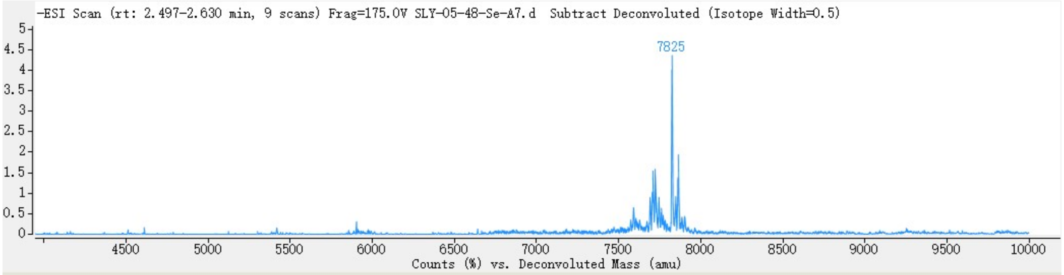


UPLC chromatograph and deconvoluted MS of **10w.**

**Conversion: 52%**

**Calculated Mass: 7832 Da; Found Mass: 7833 Da**

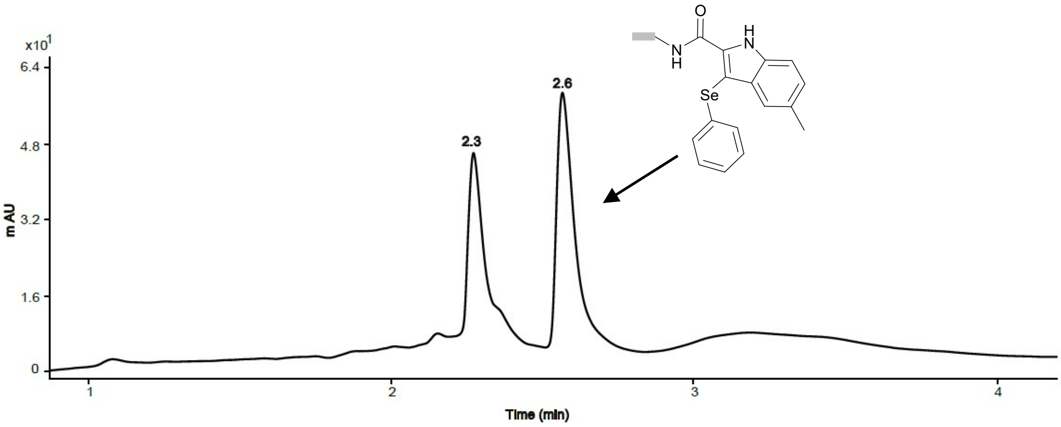


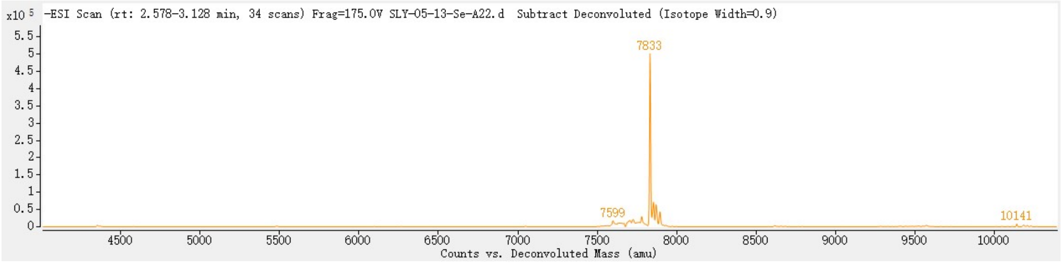

UPLC chromatograph and deconvoluted MS of **10ab.**

**Conversion: 61%**

**Calculated Mass: 7818 Da; Found Mass: 7819 Da**

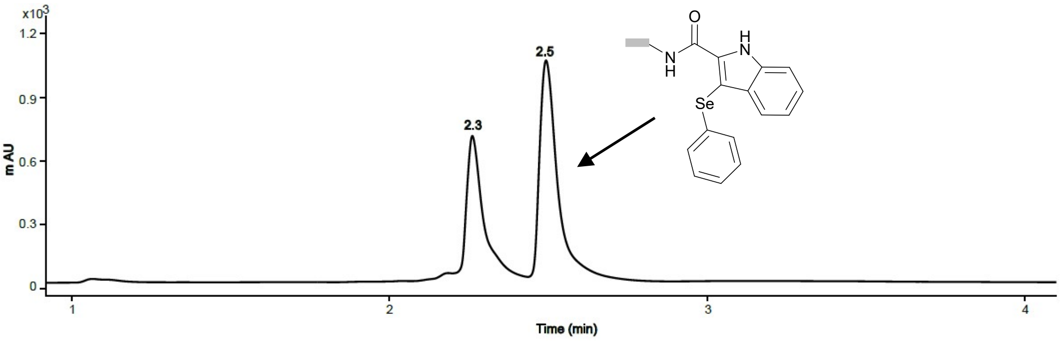


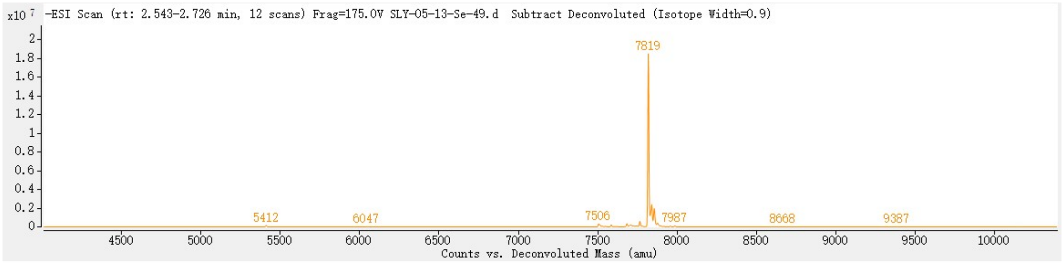


UPLC chromatograph and deconvoluted MS of **10ad.**

**Conversion: 60%**

**Calculated Mass: 7836 Da; Found Mass: 7837 Da**

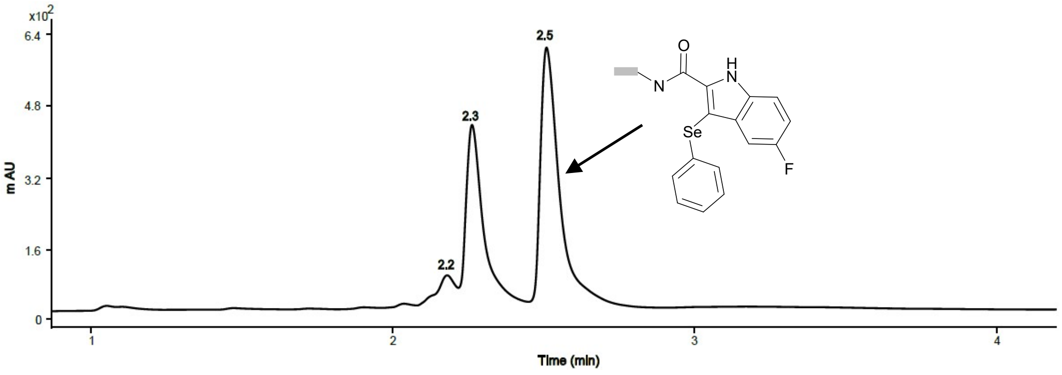


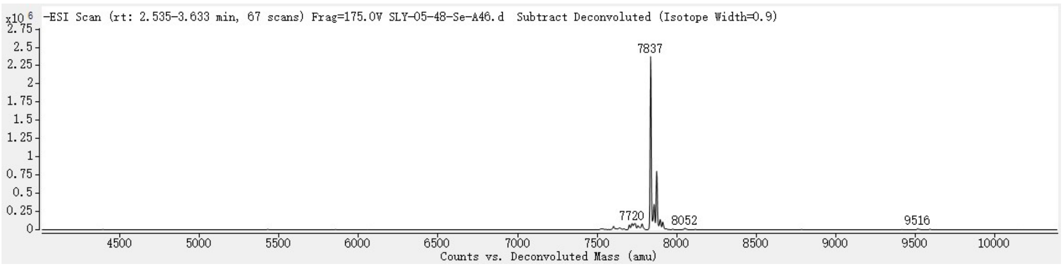

UPLC chromatograph and deconvoluted MS of **10af.**

**Conversion: 50%**

**Calculated Mass: 7860 Da; Found Mass: 7861 Da**

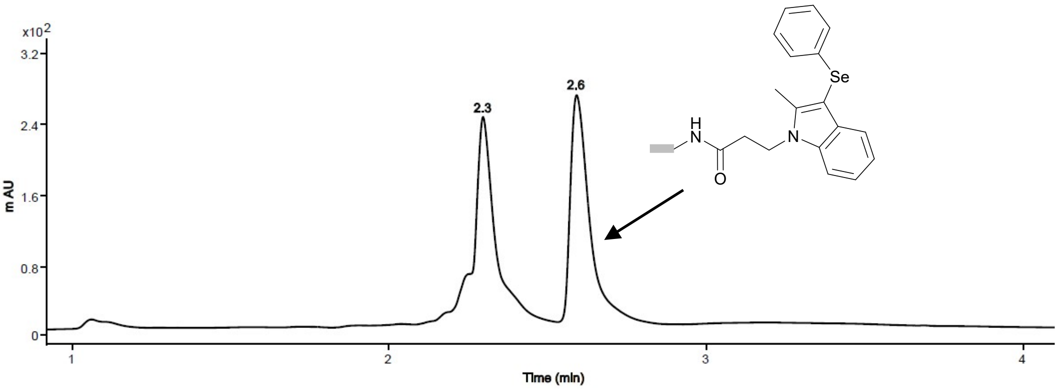


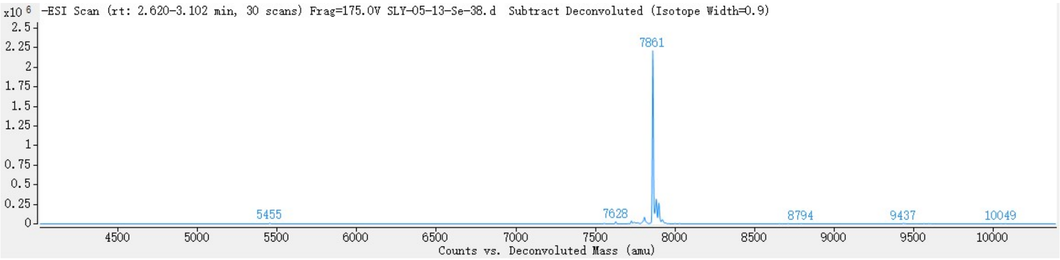


UPLC chromatograph and deconvoluted MS of **10ag.**

**Conversion: 20%**

**Calculated Mass: 7860 Da; Found Mass: 7861 Da**

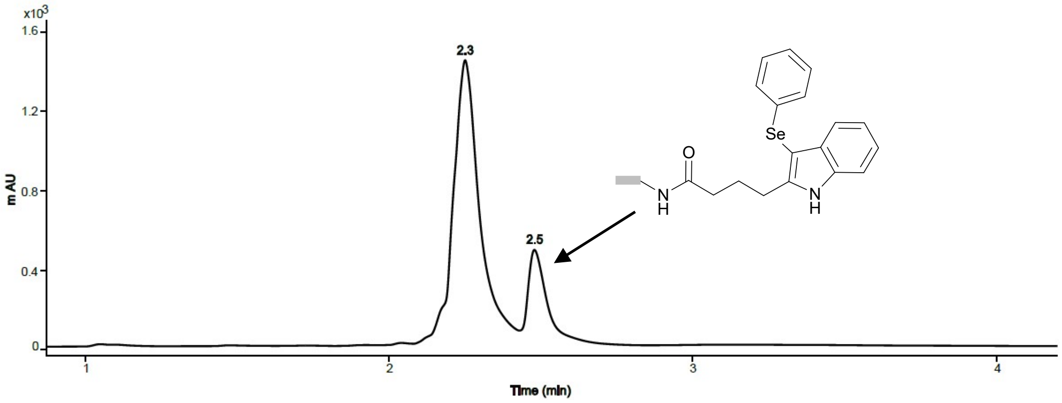


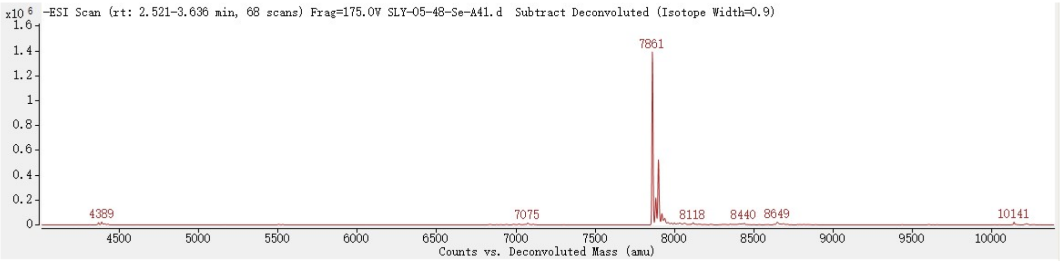

UPLC chromatograph and deconvoluted MS of **10ah.**

**Conversion: 17%**

**Calculated Mass: 7814 Da; Found Mass: 7815 Da**

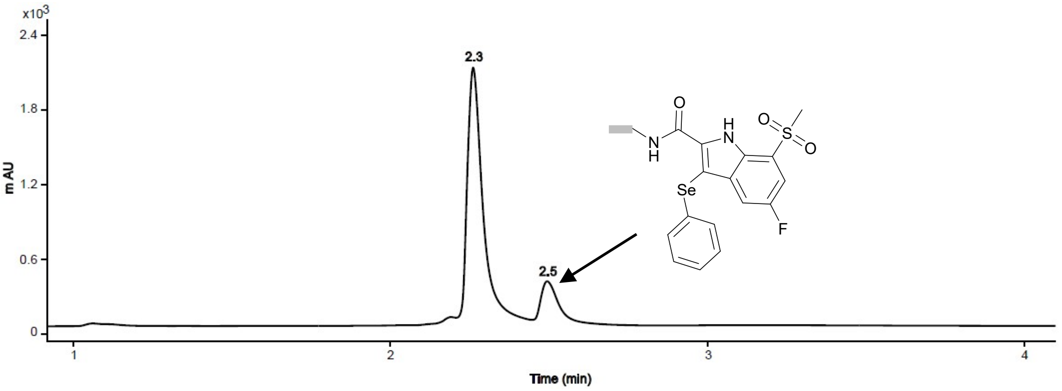


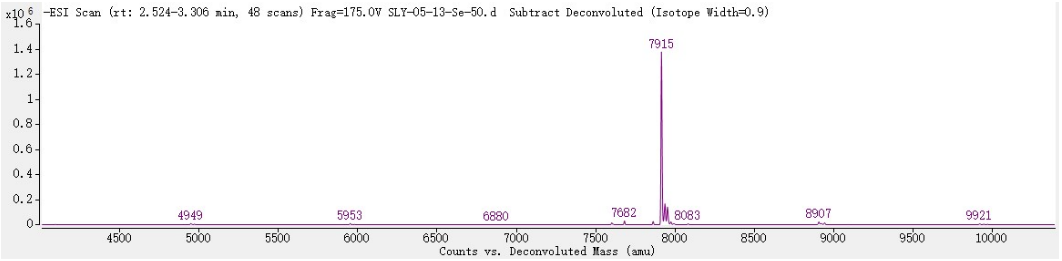


UPLC chromatograph and deconvoluted MS of **10ai.**

**Conversion: 60%**

**Calculated Mass: 7896 Da; Found Mass: 7898 Da**

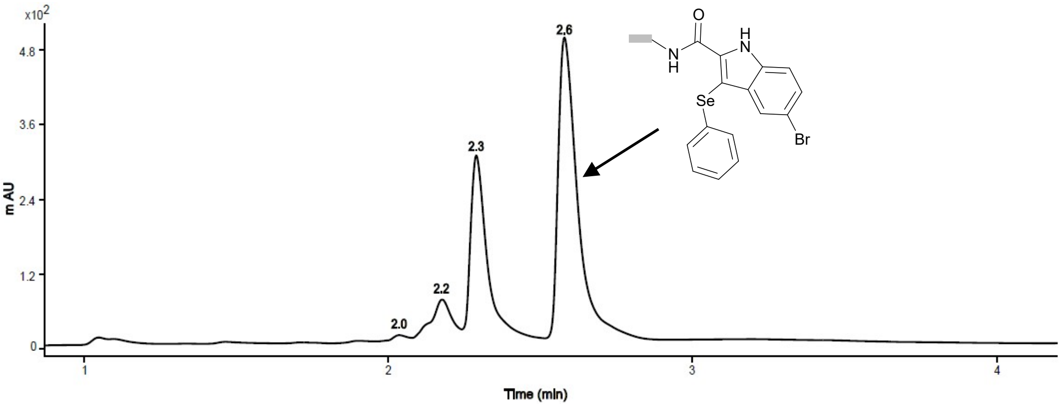


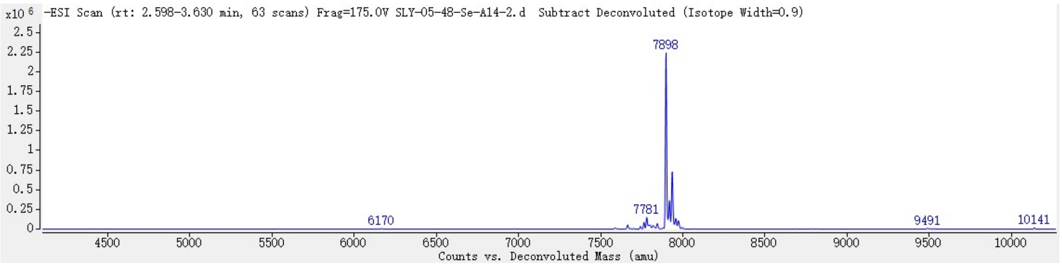

## 7. Application of on-DNA Selenylation in Mock Peptide-like DEL Synthesis

### 7.1 General procedure for DNA ligation

To the DNA conjugate HP-P (5 nmol, 5.0 μL, 1.0 equiv.) was added DNA code 1 (5'-phosphate-TAGTTCTCGGAAG-3' -OH, 7 nmol, 3.5 μL, 1.4 equiv.), DNA code 1' (5'-phosphate-TCCGAGAACTACA-3' -OH, 7 nmol, 3.5 μL, 1.4 equiv.) and nuclease-free water (5.0 μL), followed by the addition of 10× ligation buffer (2.0 μL, HitGen Inc.) and T4 DNA ligase (1 μL, 2000 U, HitGen Inc.).[3] The reaction mixture was incubated at 20 ℃ for 8 h. The conversions were all over 80% based on denaturing PAGE analysis.

### 7.2 Regioselectivity validation

**(a)**

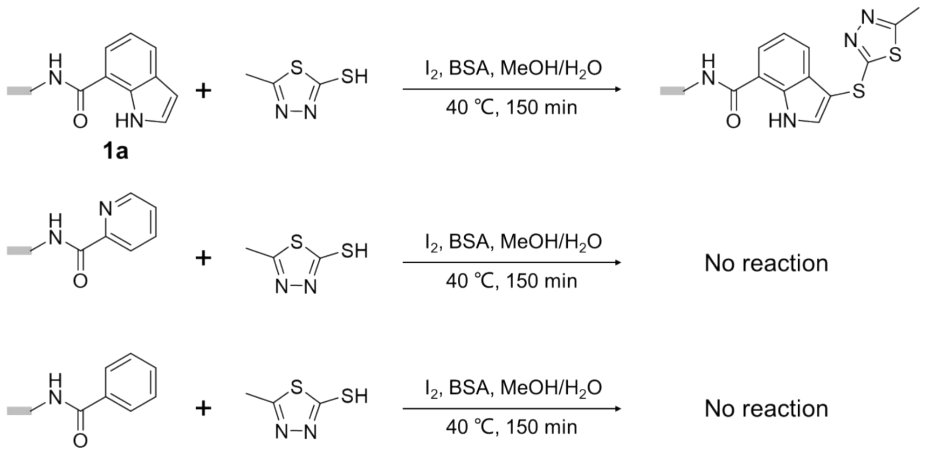

(b)

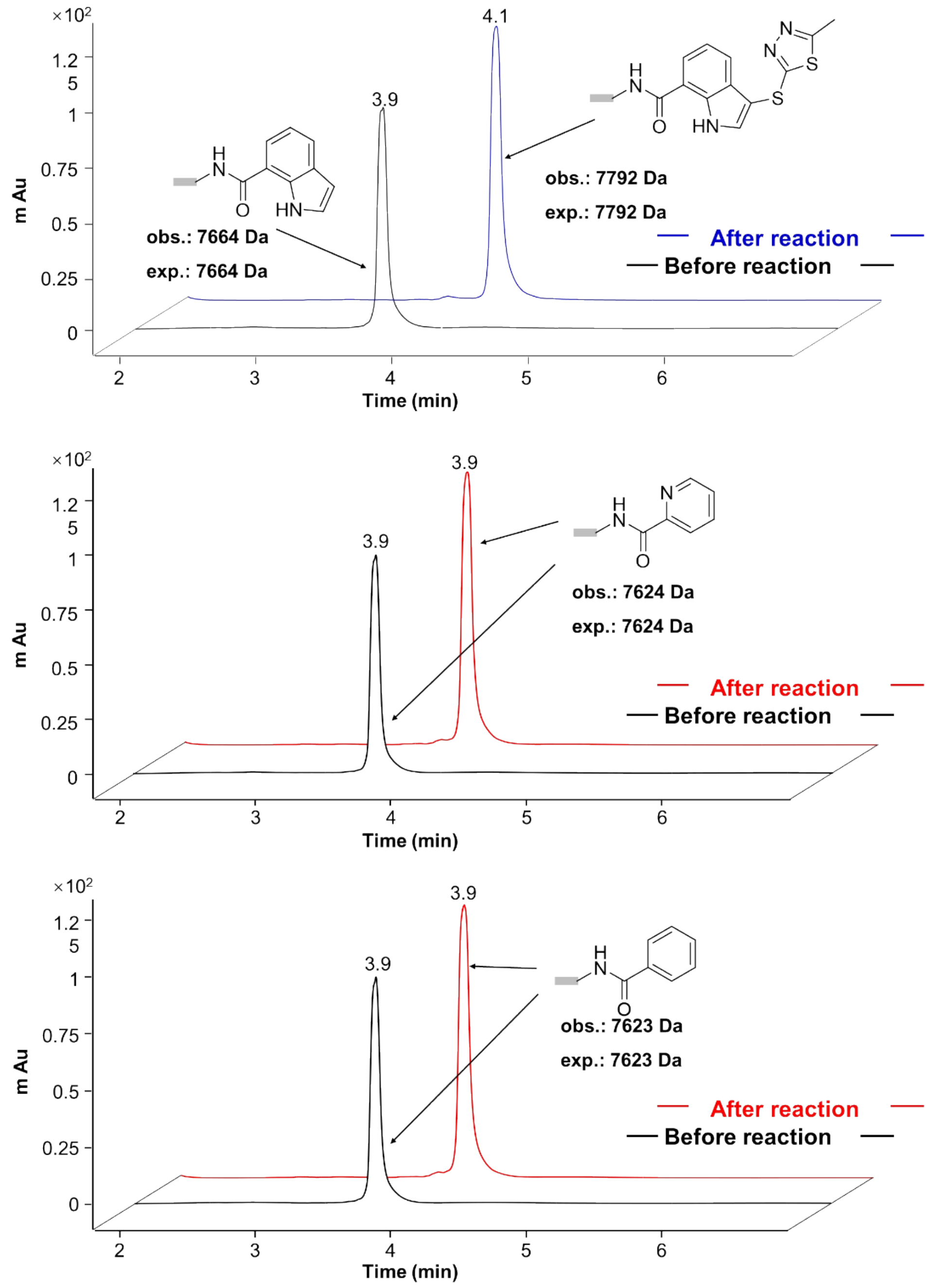


**Fig. S10.** (a) Three DNA-conjugated aryl compounds were tested with 5-methyl thiadiazole under the optimized condition. Compound **1a** afforded the corresponding product as the positive control while the other two aryl rings derivatives gave no product, indicating that no sulfenylation occurred under the reaction condition. (b) HPLC chromatograph of the three reactions.

## 7.3 General procedure for mock peptide-like DEL synthesis

**Scheme S10.** Synthetic protocol of the indole-based focused DELs.

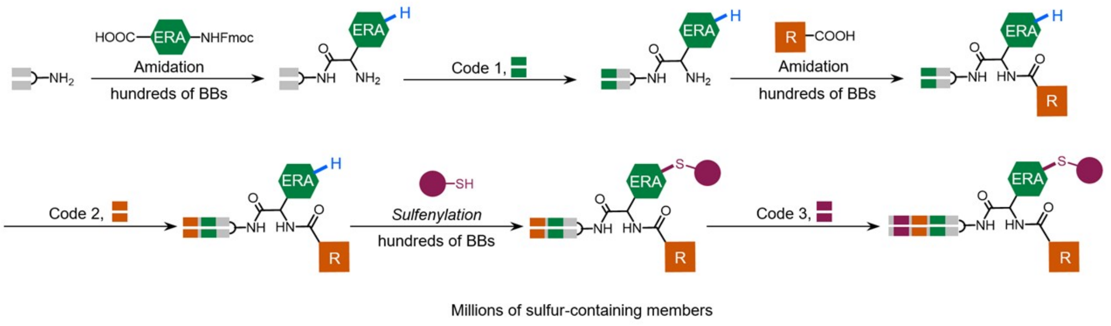


HP-P was initially coupled with ERA-containing Fmoc-protected amino acid BBs via amide formation reaction and then tagged with bar-coding DNA. The product was then coupled with aryl carboxylic acid BBs, followed by enzymatic ligation to achieve DNA conjugate. Round 3 included the sulfenylation onto the ERAs under the optimized condition, and then the products were coded with DNA tags.

## 7.4 Mock peptide-like DEL synthesis

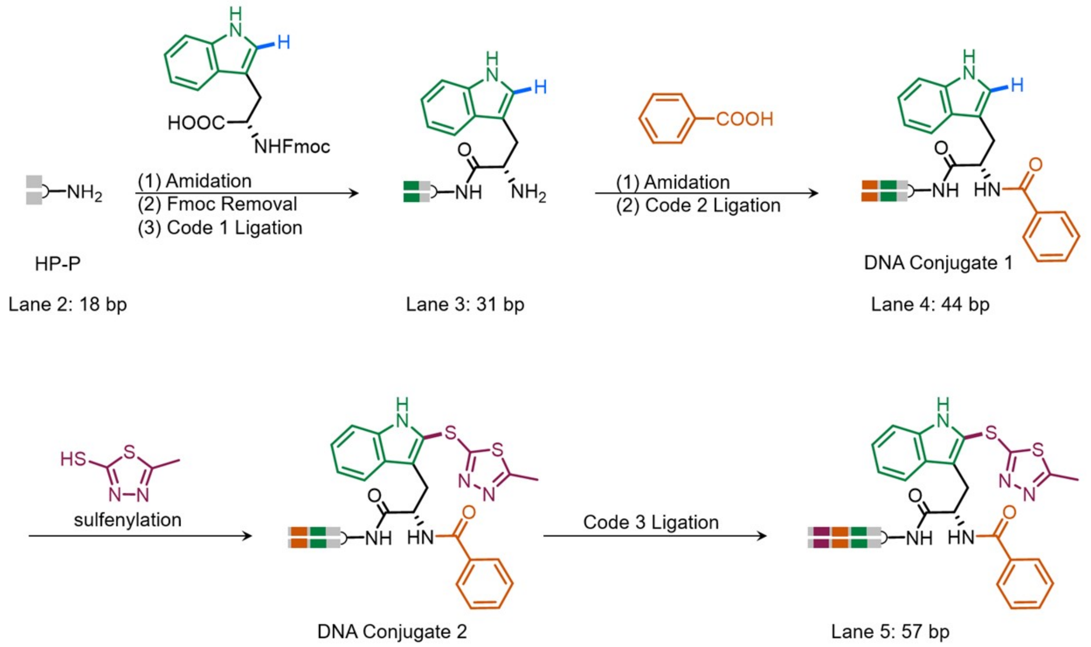

(b)

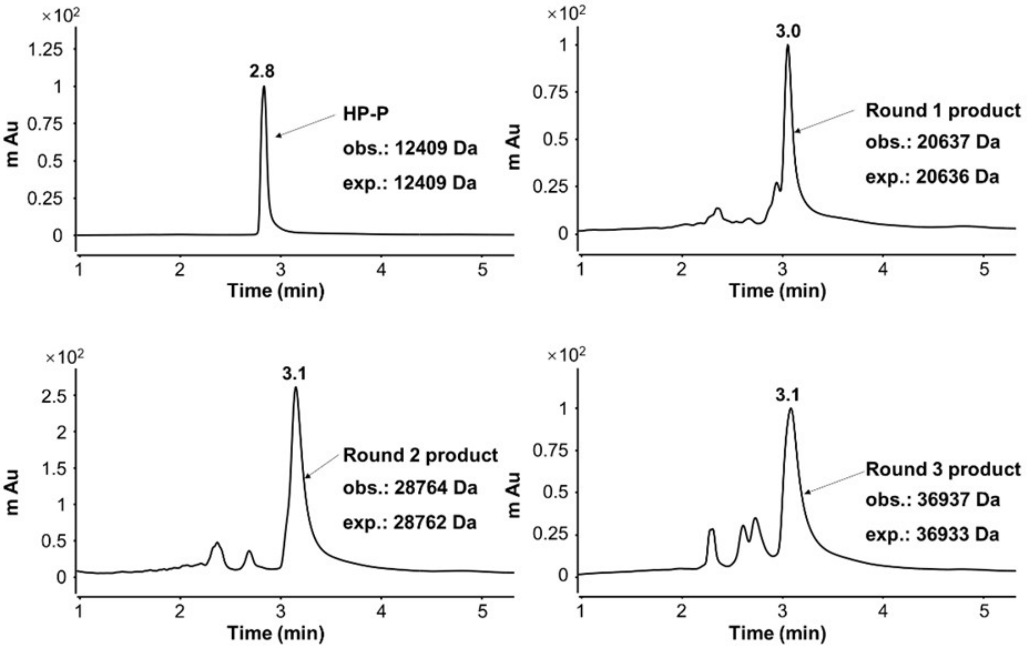


(c)

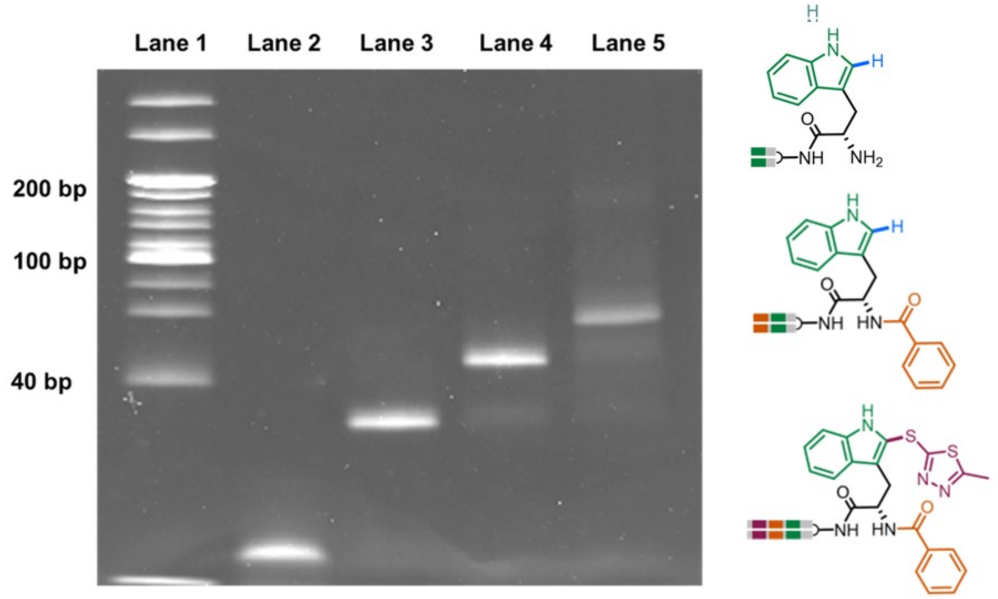


**Fig. S11.** Mock peptide-like DEL synthesis. (a) General procedure for DEL synthesis. (b) UPLC chromatogram of DNA conjugate in each round. (c) 20% PAGE characterization of DNA ligation in each round.

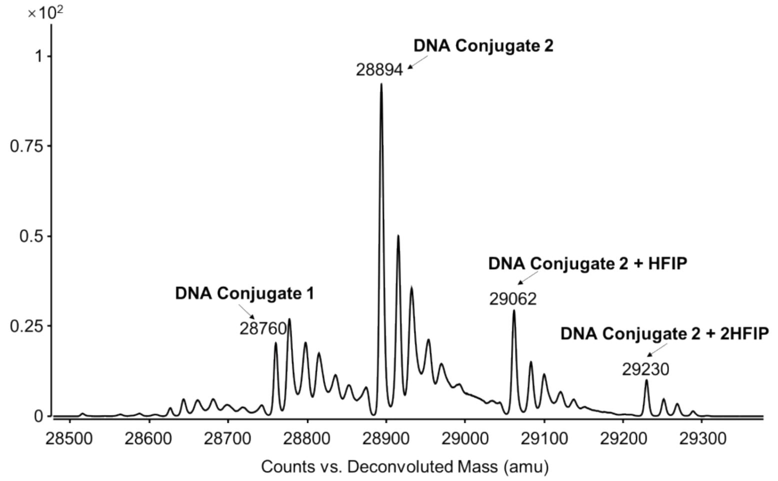


**Fig. S12.** Mass spectrum of DNA conjugate 2.

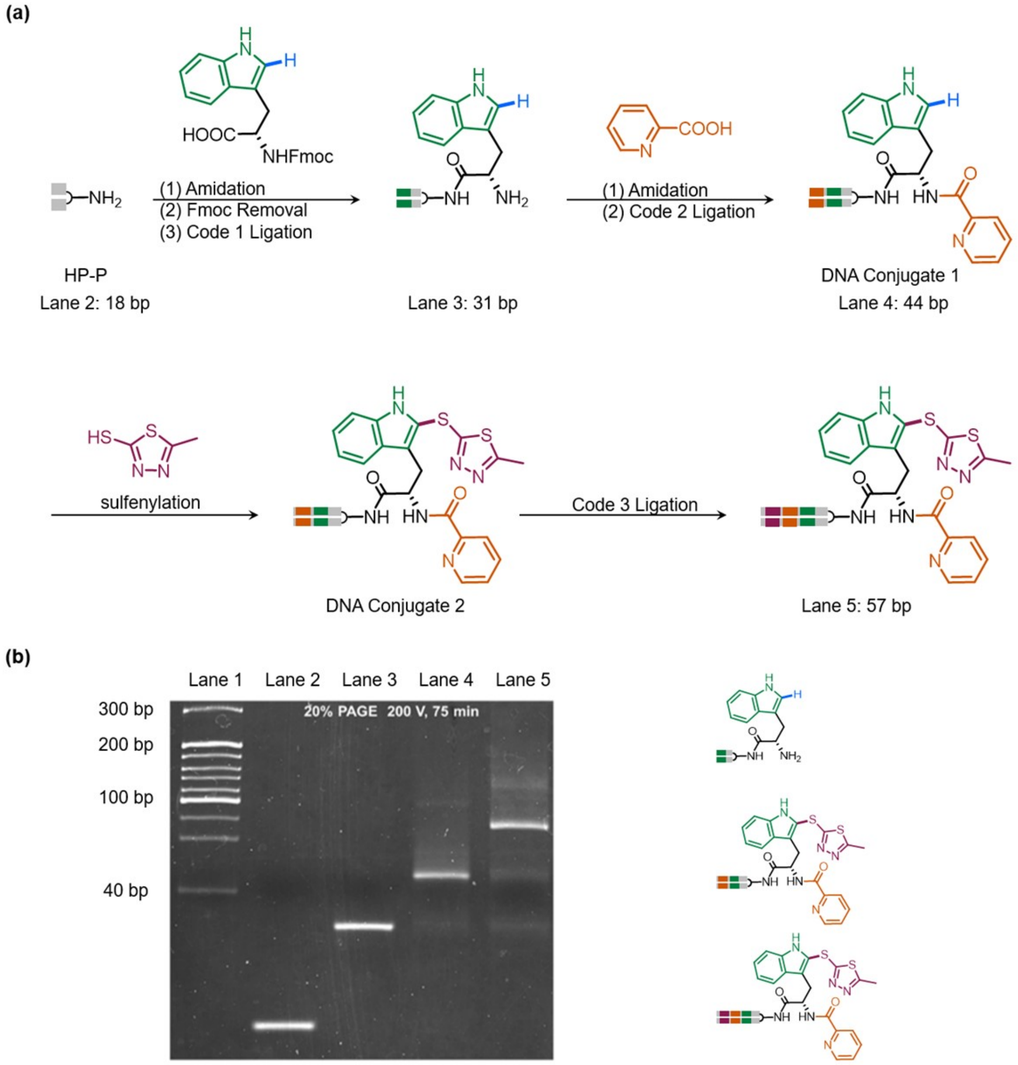


**Fig. S13.** Another mock peptide-like DEL synthesis. (a) General procedure for DEL synthesis. (b) 20% PAGE characterization of DNA ligation in each round.

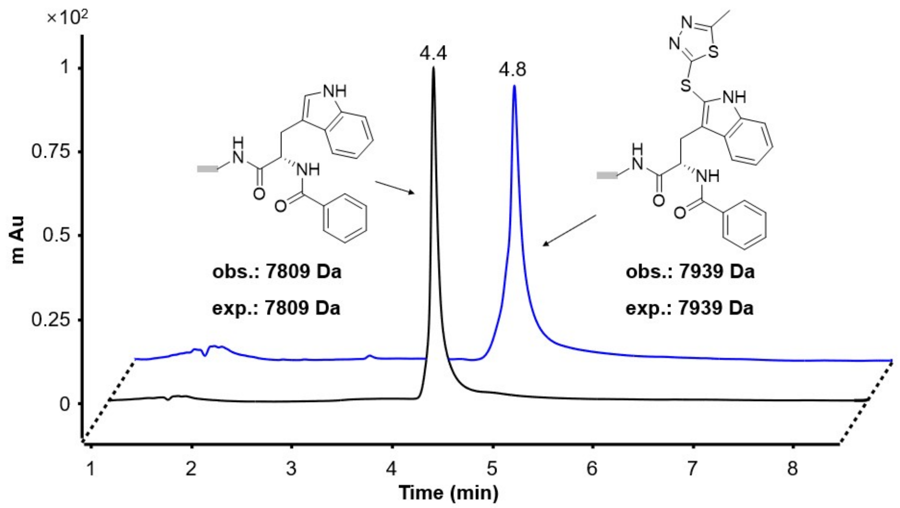


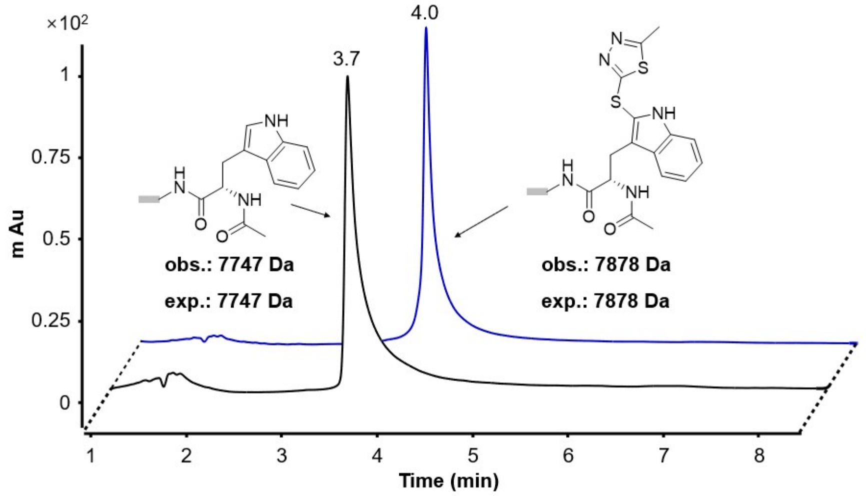


**Fig. S14.** Two DNA-conjugated protected-tryptophans were reacted with 2-mercapto-5-methyl-1,3,4-thiadiazole to afford corresponding 2-indole sulfenylated products, respectively.

### 7.5 Stability of the sulfur/selenium-containing DNA-conjugates under the standard affinity selection condition.

**Protocol 1[4]:**

Sulfur/selenium-containing DNA-conjugates working solution: Mix the 500 pmol sulfur/selenium-containing DNA-conjugates in $H_2O$ (5 µL) with PBST-HS (5 µL) and PBST (90 µL) in order to obtain the DECL working solution.

PBS solution is 50 mM sodium phosphate and 100 mM NaCl, pH 7.4. Dissolve sodium phosphate and sodium chloride in water under constant stirring. Adjust the pH with 5 M NaOH. The buffer should be freshly prepared.

PBST is PBS, to which Tween 20 is added under constant stirring, to yield a Tween 20 concentration of 0.05% (vol/vol).

PBST-HS is PBST, containing 0.2 mg/ml herring sperm DNA. Prepare by adding

10mg/ml herring sperm DNA solution to PBST.

A 100 pmol biotinylated CA-II was immobilized on 10 µL streptavidin magnetic beads (Promrga, Z5418). Sulfur/selenium-containing DNA-conjugates working solution (100 µL) was added and incubated with the immobilized CA-II for 1 h at room temperature. The magnetic beads were then separated and the supernatant recovered by ethanol precipitation (Figure S15c, S16c).

**Protocol 2[5]:**

A 100 pmol biotinylated CA-II was immobilized on 10 µL streptavidin magnetic beads (Promrga, Z5418). Sulfur/selenium-containing DNA conjugates (500 pmol) in 100 uL selection buffer (50mM Tris-HCl, pH 7.5, 150mM NaCl, 0.1% tween-20, 1mg/mL herring sperm DNA (Invitrogen, cat: 15634-017), 1mg/mL BSA, and 1mM βME) was incubated with the immobilized CA-II for 1 h at room temperature. The magnetic beads were then separated and the supernatant recovered by ethanol precipitation(Figure S15d, S16d).

**Protocol 3[6]:**

Sulfur/selenium-containing DNA-conjugates (5 µL, 500 pmol) was added to 95 µL working buffer containing 1.0 µM biotinylated carbonic anhydrase II (CA-II) in 0.1 M sodium phosphate, pH 8.0, 0.25 M NaCl, 0.02% (V/V) tween-20, 0.2 mg/mL herring sperm DNA (Invitrogen, cat: 15634-017). And the CA-II to the mixture and incubated 2 hours at 25 °C. Then incubated at 0 °C for 30 min before light irradiation at 365 nm by a UVP CL-1000 Ultraviolet crosslinker. The mixture was incubated with pre-washed streptavidin magnetic beads (Promrga, Z5418) for 2 hours. The magnetic beads were then separated and the supernatant recovered by ethanol precipitation(Figure S15e, S16e).

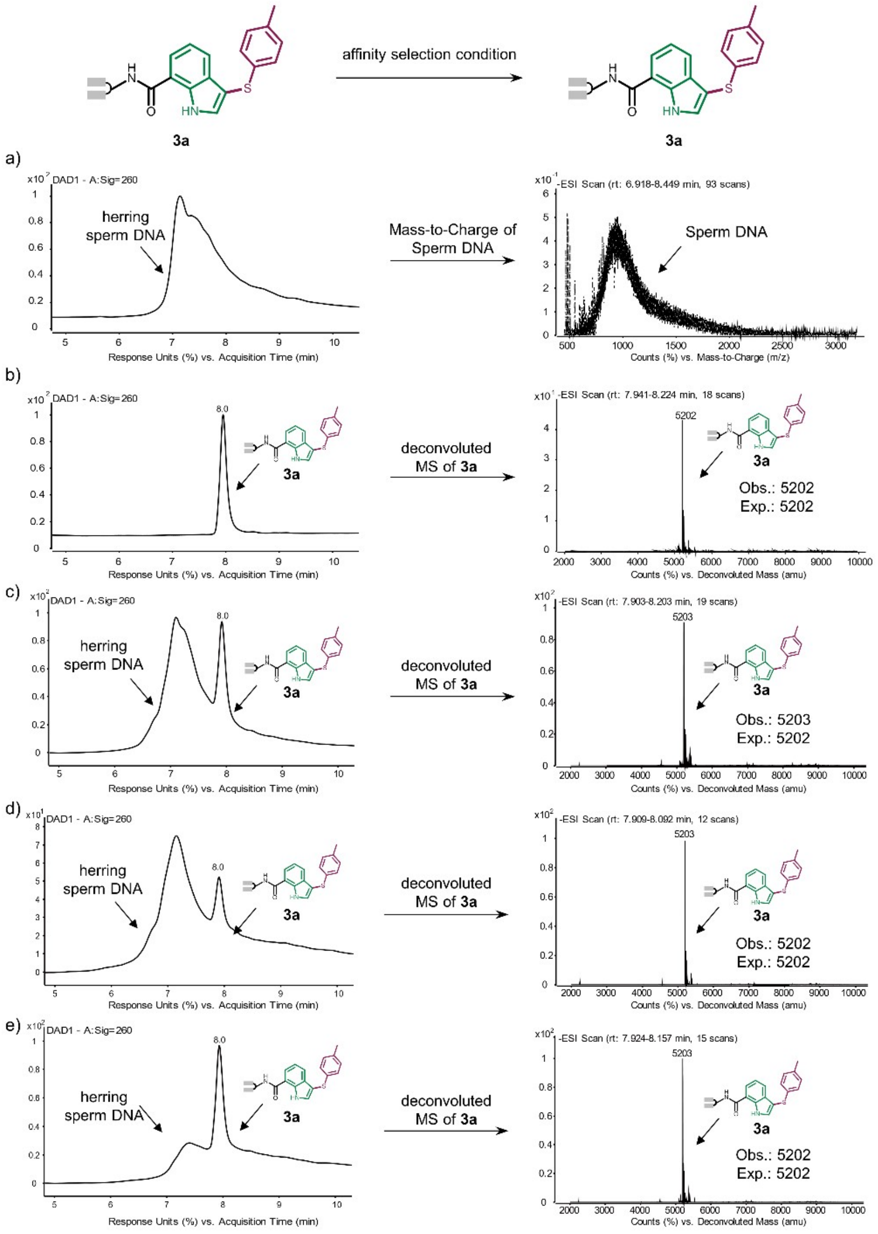


**Fig. S15.** Stability of the sulfur-containing DNA-conjugates (**3a**) under affinity selection conditions. a) herring sperm DNA; b) **3a** before selection; c) Stability of **3a** under the protocol 1 selection condition; d) Stability of **3a** under the protocol 2 selection condition; e) Stability of **3a** under the protocol 3 selection condition.

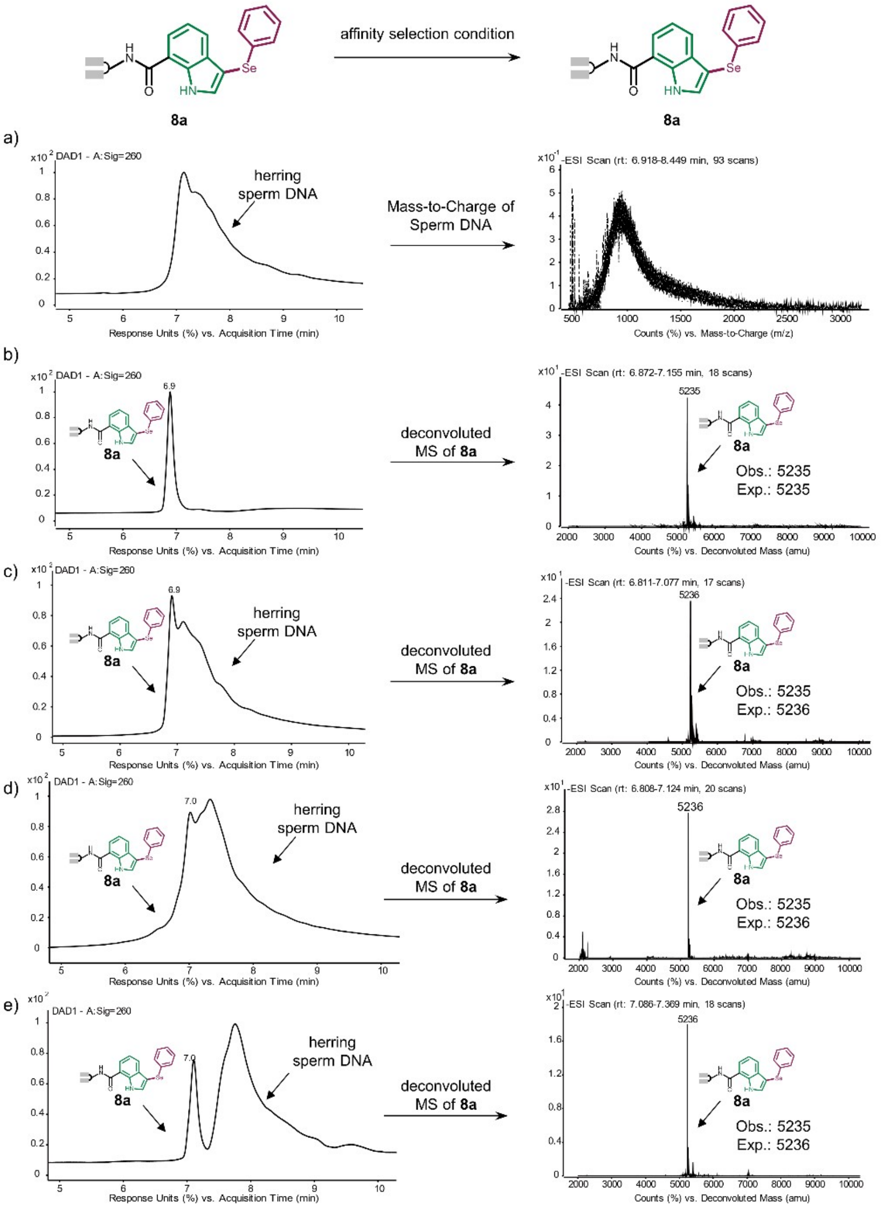


**Fig. S16.** Stability of the sulfur-containing DNA-conjugates (**8a**) under affinity selection conditions. a) herring sperm DNA; b) **8a** before selection; c) Stability of **8a** under the protocol 1 selection condition; d) Stability of **8a** under the protocol 2 selection condition; e) Stability of **8a** under the protocol 3 selection condition.

Moreover, we treated DNA-conjugated molecules sulfenylated product or selenylated product with incubation process stated above first and then processed at high temperature (95 °C) for ten minutes.

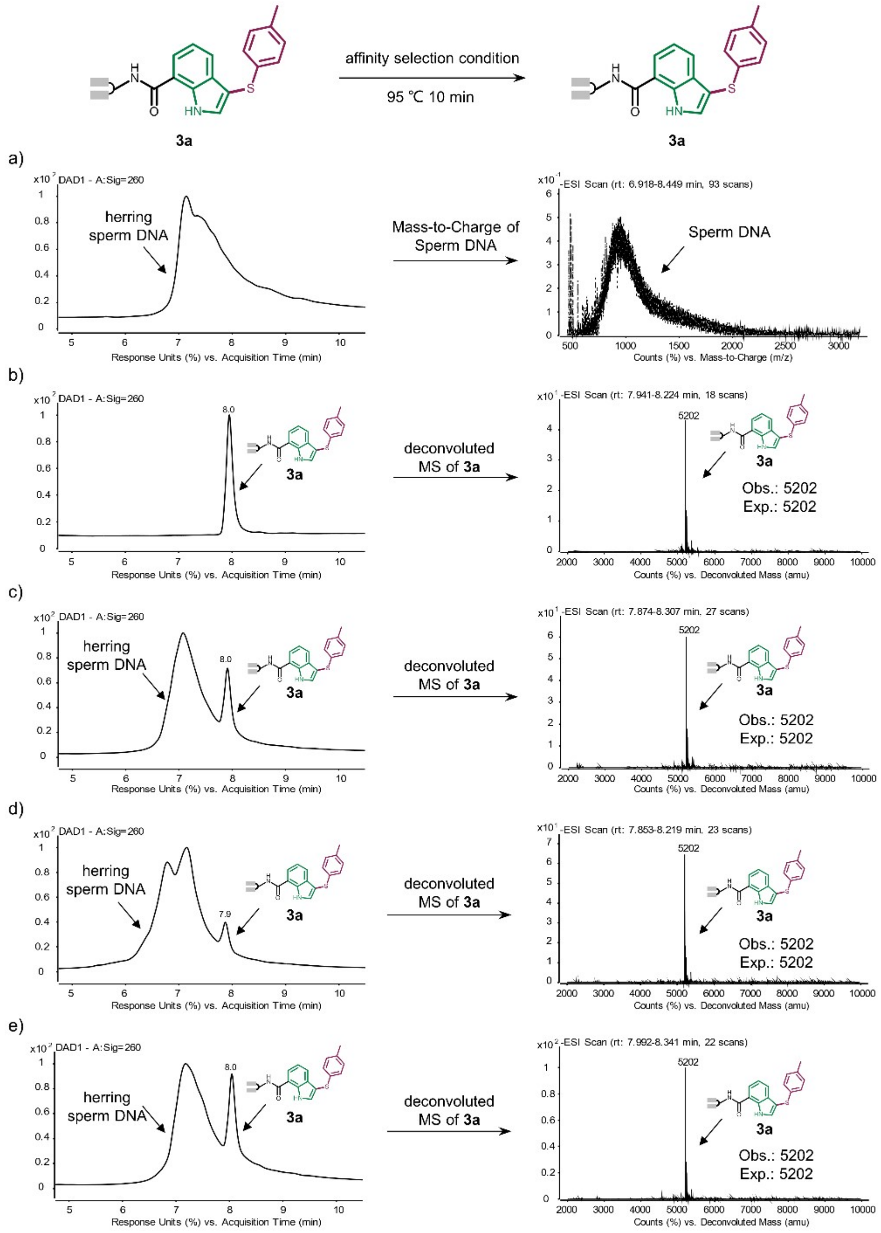


**Fig. S17.** Stability of the sulfur-containing DNA-conjugates (**3a**) under affinity selection conditions after processed at 95 °C for ten minutes. a) herring sperm DNA; b) **3a** before selection; c) Stability of **3a** under the protocol 1 selection condition; d) Stability of **8a** under the protocol 2 selection condition; e) Stability of **3a** under the protocol 3 selection condition.

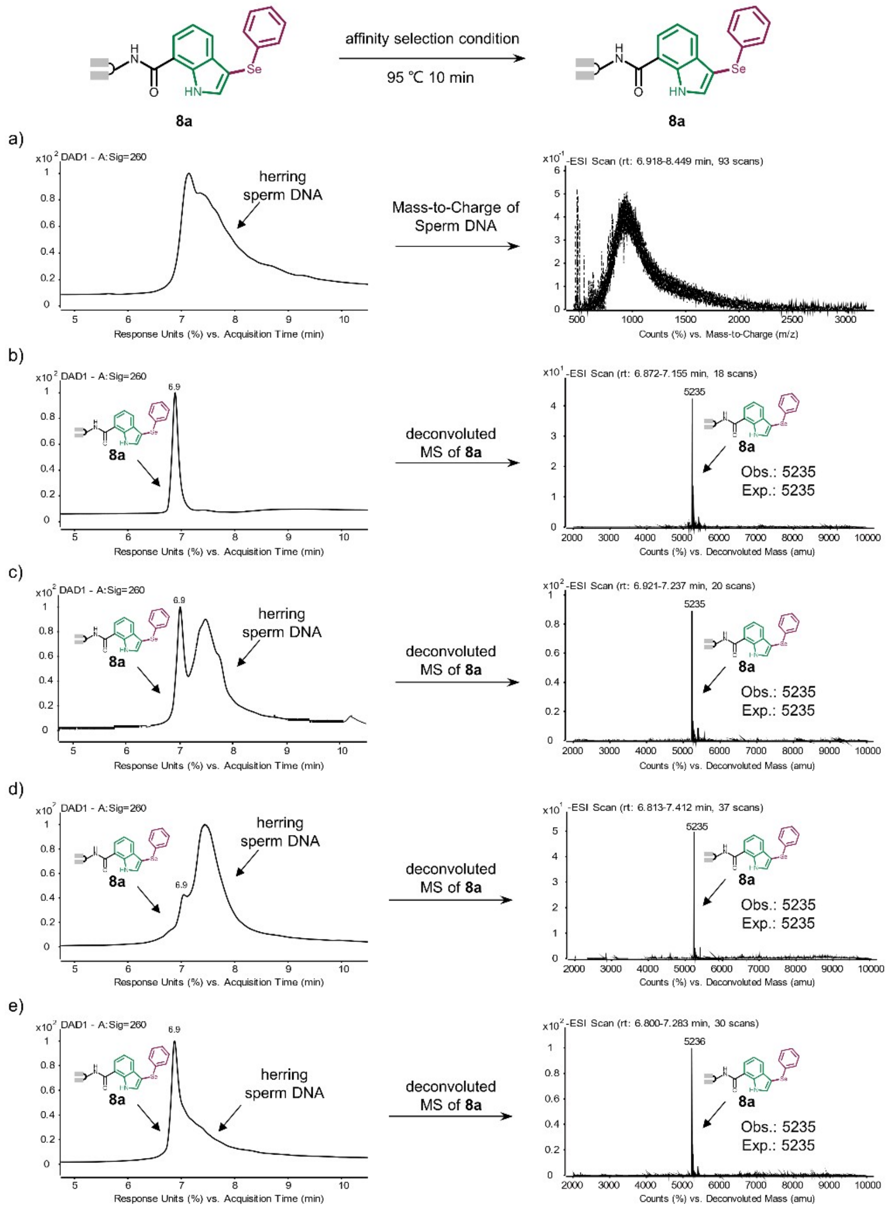


**Fig. S18.** Stability of the sulfur-containing DNA-conjugates (**8a**) under affinity selection conditions after processed at 95 °C for ten minutes. a) herring sperm DNA; b) **8a** before selection; c) Stability of **8a** under the protocol 1 selection condition; d) Stability of **8a** under the protocol 2 selection condition; e) Stability of **8a** under the protocol 3 selection condition.

No detectable DNA damage was observed under these protocols, which demonstrated the stability of the sulfur/selenium-containing compounds under the standard affinity selection condition.

## 8. References


1. D. T. Flood, S. Asai, X. Zhang, J. Wang, L. Yoon, Z. C. Adams, B. C. Dillingham, B. B. Sanchez, J. C. Vantourout, M. E. Flanagan, D. W. Piotrowski, P. Richardson, S. A. Green, R. A. Shenvi, J. S. Chen, P. S. Baran and P. E. Dawson, *J. Am. Chem. Soc.*, 2019, **141**, 9998-10006.
2. Saima, D. Equbal, A. G. Lavekar and A. K. Sinha, *Org. Biomol. Chem.*, 2016, **14**, 6111-6118.
3. S. Dawadi, N. Simmons, G. Miklossy, K. M. Bohren, J. C. Faver, M. N. Ucisik, P. Nyshadham, Z. Yu and M. M. Matzuk, *Proc. Natl. Acad. Sci. U. S. A.*, 2020, **117**, 16782.
4. W. Decurtins, M. Wichert, R. M. Franzini, F. Buller, M. A. Stravs, Y. Zhang, D. Neri and J. Scheuermann, *Nat. Protoc.*, 2016, **11**, 764-780.
5. M. A. Clark, R. A. Acharya, C. C. Arico-Muendel, S. L. Belyanskaya, D. R. Benjamin, N. R. Carlson, P. A. Centrella, C. H. Chiu, S. P. Creaser, J. W. Cuozzo, C. P. Davie, Y. Ding, G. J. Franklin, K. D. Franzen, M. L. Gefter, S. P. Hale, N. J. Hansen, D. I. Israel, J. Jiang, M. J. Kavarana, M. S. Kelley, C. S. Kollmann, F. Li, K. Lind, S. Mataruse, P. F. Medeiros, J. A. Messer, P. Myers, H. O'Keefe, M. C. Oliff, C. E. Rise, A. L. Satz, S. R. Skinner, J. L. Svendsen, L. Tang, K. van Vloten, R. W. Wagner, G. Yao, B. Zhao and B. A. Morgan, *Nat. Chem. Biol.*, 2009, **5**, 647-654.
6. G. Zhao, S. Zhong, G. Zhang, Y. Li and Y. Li, *Angew. Chem. Int. Ed*, 2021, DOI: 10.1002/anie.202115157, e202115157.
